# Divergent Enzymatic Synthesis of a Comprehensive Type‑1 Glycan Determinant Library

**DOI:** 10.1021/acscatal.5c06702

**Published:** 2025-12-01

**Authors:** Guitao Bai, Tangliang Shen, MohammadHossein Shabahang, Shumin Bao, Shuquan Fan, Hongshuai Lv, Lei Li

**Affiliations:** Department of Chemistry and Center for Diagnostics & Therapeutics, 1373 Georgia State University, Atlanta, Georgia 30303, United States

**Keywords:** divergent enzymatic synthesis, glycan determinant, glycan microarray, Lewis antigen, type-1 unit

## Abstract

Type-1 glycan unit, Galβ1-3GlcNAc, is one of the two major backbones from which the large glycan determinant repertoire is derived. Numerous type-1 glycan determinants, including Lewis-series antigens, have been identified, often featuring fucosylation and sialylation. In this study, we achieved efficient synthesis of 30 type-1 determinants using a divergent enzymatic strategy. The synthetic efficiency of eight fucosyltransferases (FucTs) was evaluated for the installation of fucose at every step of the type-1 determinant synthesis. Using selected FucTs and a few additional robust glycosyltransferases, 22 basic type-1 determinants were constructed at preparative scales in no more than three steps, starting from the type-1 unit. Additionally, 8 extended type-1 Lewis antigens were successfully synthesized in no more than five steps. Subsequent glycan microarray assays of these glycan determinants revealed intriguing recognitions by glycan-binding proteins and anti-type-1 Lewis antigen monoclonal antibodies.

## Graphical abstract

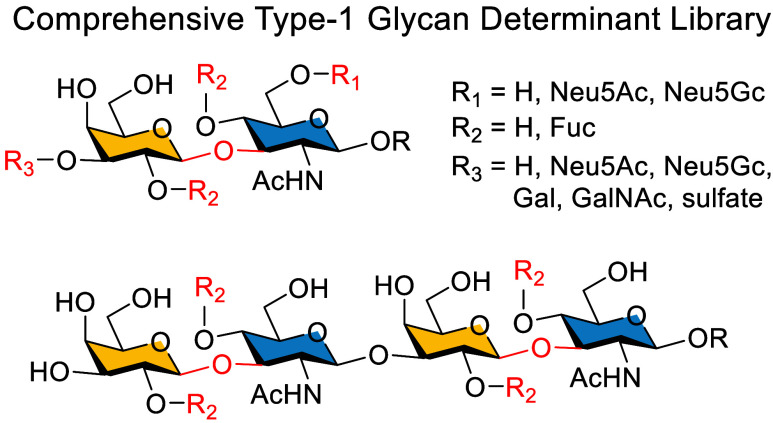

## Introduction

Type-1 glycan unit (Galβ1-3GlcNAc, also known as type-1 LacNAc, type-1 LN, or Lewis C) stands as one of the two fundamental backbones of terminal epitopes in diverse structures of eukaryotic glycans and glycoconjugates.^1^ These epitopes, also referred to as glycan determinants,^2^ directly engage glycan-binding proteins (GBPs), mediating a broad range of physiological and pathological processes, including cell adhesion,^3^ immune recognition,^4^ pathogen–host interactions,^5,6^ and tumor progression.^7^ Type-1 glycan determinants often feature additional fucosylation and/or sialylation, leading to structurally and functionally distinct antigens such as various type-1 Lewis blood group antigens (Figure 1).^2^ Among these, sialyl-Lewis A (SLe^A^), also known as carbohydrate antigen 19-9 (CA19-9), is a tumor-associated carbohydrate antigen (TACA) whose elevated levels are closely associated with diverse carcinomas, including pancreatic, gastric, and colorectal cancers.^8–10^ Consequently, SLe^A^ is routinely employed as a tumor marker in clinical diagnostics and biomedical research.^8^ Nonsialylated Lewis A (Le^A^) and Lewis B (Le^B^) are extensively expressed in the gastrointestinal tract. Particularly, Le^B^, along with its blood group A/B derivatives (A-Le^B^ and B-Le^B^, Figure 1), acts as a receptor for *Helicobacter pylori*,^11,12^ a bacterium that causes chronic gastritis and peptic ulcers. In addition, 3′-O-sulfated Lewis A/C antigens are often aberrantly expressed in numerous precancerous lesions and malignancies.^13^

**Figure 1:**
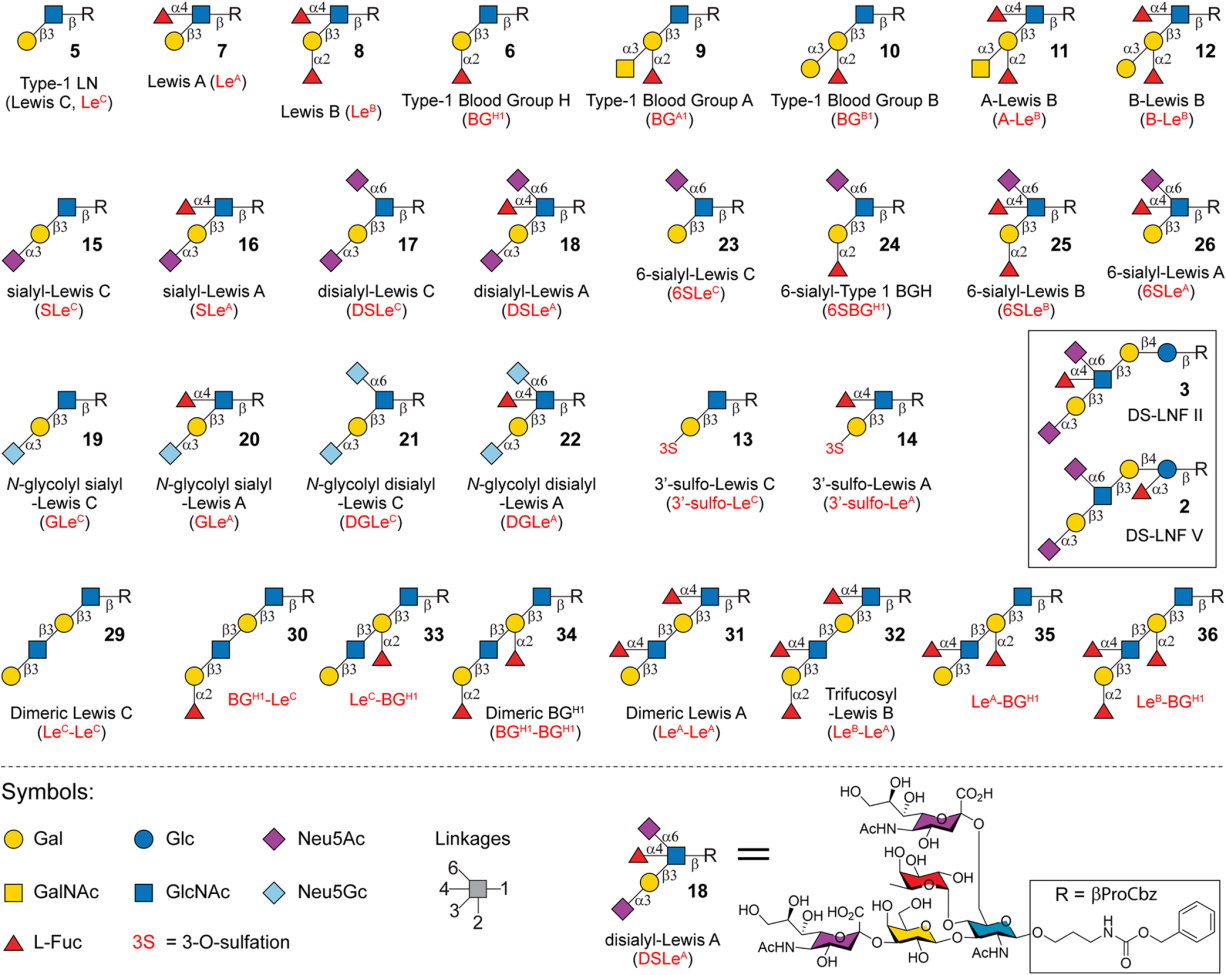
Structures of all 30 type-1 glycan determinants and two HMOs (DS-LNF II and DS-LNF V) prepared in this work. Gal, galactose; GalNAc, *N*-acetylgalactosamine; Glc, glucose; GlcNAc, *N*-acetylglucosamine; Neu5Ac, *N*-acetylneuraminic acid; Neu5Gc, *N*-glycolylneuraminic acid; Fuc, l-fucose.

Interestingly, several type-1 glycan determinants feature an atypical α2-6 sialylation at the C6 position of the GlcNAc residue, producing disialyl-Lewis A/C (DSLe^A/C^) and related epitopes (Figure 1).^14^ Both DSLe^A^ and DSLe^C^ were initially identified in human colonic adenocarcinoma.^15,16^ Subsequent investigations showed that they primarily occur on normal epithelial cells and generally exhibit an inverse correlation with tumor progression.^17^ Other type-1 determinants include 6-sialyl-Lewis C (6SLe^C^), found in the mouse cerebral cortex,^18^ and *N*-glycolyl (di)­sialyl-Lewis A (GLe^A^ and DGLe^A^), found in both normal epithelial and cancer cells.^19^ Notably, many type-1 determinants, including Le^A/B^, SLe^A^, DSLe^A/C^, 6SLe^C^, and 6-sialyl-type-1 blood group H-antigen (6SBG^H1^) (Figure 1), have been identified on various structures of human milk oligosaccharides (HMOs).^20^ Beyond these basic type-1 determinants, extended glycan chains featuring tandem repeats of type-1 LN have been identified, exemplified by TACAs such as dimeric Le^A^ (Le^A^–Le^A^) and trifucosyl-Le^B^ (Le^B^–Le^A^) (Figure 1) that were observed in human colorectal carcinoma cell lines.^21–23^ These findings underscore the structural diversity of type-1 glycan determinants and highlight their significance in both normal physiology and disease pathology.

Despite their high prevalence and extensive functional studies, the synthesis of type-1 glycan determinants remains scattered. While Le^A/B/C^, SLe^A/C^, and type-1 blood group ABH antigens have been extensively synthesized chemically^24^ and enzymatically^25–40^ (often as terminal epitopes of more complex glycans such as HMOs), efforts to access other determinants are scarce. For example, A-Le^B^ and B-Le^B^ on poly-LacNAc chains were only synthesized by Ye et al. via α1-4 fucosylation of BG^A1^ and BG^B1^ antigens.^32^ We and others recently synthesized HMOs bearing DSLe^C^, 6SLe^C^, and 6SBG^H1^ antigens;^36,39^ however, attempts to synthesize HMOs containing DSLe^A^ failed in both cases due to the absence of a capable α1-4 fucosyltransferase (α1-4FucT). The chemical synthesis of glycolipids containing DSLe^A/C^ was achieved two decades ago but with low overall yields.^41^ Similarly, attempts to produce extended type-1 determinants are limited, with only Le^A^ tandem repeats^42^ and a few segments of the Le^B^–Le^A^ epitope having been prepared.^43^ Overall, most type-1 determinants have been prepared as terminal epitopes of various complex glycans, lacking a consistent and defined format for functional studies as underlying substructures can significantly influence their recognition by GBPs. Meanwhile, many type-1 determinants remain largely inaccessible (e.g., DSLe^A^, 3′-sulfo-Le^A/C^, Neu5Gc-containing epitopes, and extended type-1 structures), highlighting a pressing need for an efficient and practical strategy to prepare a comprehensive suite of type-1 determinants. To address this, we systematically reevaluated the synthetic efficiency and substrate scope of eight fucosyltransferases (FucTs), enabling a divergent enzymatic approach for the streamlined synthesis of a 30-member library of type-1 glycan determinants (Figure 1).

## Materials and Methods

### Materials

All chemicals were purchased from Fisher Scientific (Hampton, NH), unless otherwise stated. Monosaccharides and nucleotides used for the synthesis of sugar nucleotides were purchased from Biosynth (Gardner, MA). The XBridge peptide BEH C18 column (130Å, 5 μm, 4.6 × 250 mm) was obtained from Waters Corp (Milford, MA). Bio-Gel P2 and P4 resins were purchased from Bio-Rad Laboratories (Hercules, CA). Silica gel plates 60 F254 for thin-layer chromatography (TLC) were acquired from MilliporeSigma (Burlington, MA).

Expression vectors for N-terminal GFP-tagged human enzymes FUT1, FUT3, FUT5, and Gal3ST2 were purchased from Glyco Expression Technologies, Inc. (Athens, GA). Enzymes including β1-3 galactosyltransferase from *Chromobacterium violaceum* (Cv3GalT),^44^ β1,3-*N*-acetylglucosaminyltransferase from *H. pylori* (HpLgtA),^44^ α1-2 fucosyltransferase from *Helicobacter mustelae* (Hm2FT),^31^ α1-3/4 fucosyltransferase from *H. pylori* (Hp3/4FT),^45^ α2-3 sialyltransferase 1 mutant M144D from *Pasteurella multocida* (PmST1m),^46^ sialic acid Aldolase,^47^ CMP-sialic acid synthetase from *Neisseria meningitides* (NmCSS),^47^ *P. multocida* pyrophosphatase (PmPpA),^48^ human *N*-acetylgalactosaminide α2,6-sialyltransferase 6 (ST6GalNAc6),^36^ α1,2 colitosyltransferase from *Escherichia coli* O55 (WbgN),^25^ α1,2 fucosyltransferase from *E. coli* O128 (WbsJ)^49^ and *Thermosynechococcus elongatus* BP-1 (Te2FT),^28^ α1-3-*N*-acetylgalactosaminyltransferase from *H. mustelae* (BgtA),^50^ and α1-3 galactosyltransferase from *E. coli* O86 (WbnI)^51^ were expressed and purified as previously reported.

Sugar nucleotide donors including uridine-5′-diphosphate-galactose (UDP-Gal), uridine-5′-diphosphate-*N*-acetylglucosamine (UDP-GlcNAc), guanosine 5′-diphospho-l-fucose (GDP-Fuc), cytidine 5′-monophospho-*N*-acetylneuraminic acid (CMP-Neu5Ac), and cytidine 5′-monophospho-*N*-glycolylneuraminic acid (CMP-Neu5Gc) were prepared as reported previously^29,52^ with brief P2-chromatography to >70% purity, freeze-dried, and stored at −20 °C for long-term use. The sulfate donor 3′-phosphoadenosine-5′-phosphosulfate (PAPS) was purchased from Glycan Therapeutics (Raleigh, NC).

### Expression and Purification of Human Enzymes

Human FUT1, FUT3, FUT5, and Gal3ST2 with N-terminal GFP tag were expressed and purified as previously reported.^53,54^ Expression plasmids pGEn2-FUT1, pGEn2-FUT3, pGEn2-FUT5, and pGEn2-Gal3ST2 were extracted and transfected into the HEK 293-F (Thermo Fisher) cell line for expression for 4 days. Proteins expressed contain an N-terminal 8× His tag and a GFP tag purified by Ni-NTA chromatography at 4 °C. Briefly, the culture medium was collected with brief centrifugation to remove cells, mixed with equal volumes of binding buffer (50 mM Tris-HCl, 300 mM NaCl, and 20 mM imidazole at pH 7.5), and applied directly onto a gravity column with 3 mL of HisPur Ni-NTA Resin (Thermo Fisher Scientific) that is preequilibrated with the binding buffer. The flowthrough was collected and loaded one more time. The resin was washed with 50 mL of binding buffer and then 50 mL of washing buffer (50 mM Tris-HCl, 300 mM NaCl, and 50 mM imidazole at pH 7.5). Finally, the protein was eluted with 10 mL of elution buffer (50 mM Tris-HCl, 300 mM NaCl, and 250 mM imidazole at pH 7.5) and desalted against storage buffer (50 mM HEPES, pH 7.0, 150 mM NaCl, 20% glycerol) for long-term storage at −80 °C. Eluted proteins were analyzed by SDS-PAGE (Figure S1) and their concentrations were determined using a Nanodrop spectrophotometer.

### General Procedures of Synthesis Using Glycosyltransferases

#### β1-3 Galactosylation by Cv3GalT

The reaction system contains Tris-HCl buffer (50 mM, pH 8.0), an acceptor (5–10 mM), 2 equiv of sugar donor UDP-Gal, MgCl_2_ (20 mM), and appropriate amounts of purified Cv3GalT (0.2 mg/mL).

#### β1-3GlcNAcylation by HpLgtA

The reaction system contains Tris-HCl buffer (50 mM, pH 8.0), an acceptor (5–10 mM), 2 equiv of sugar donor UDP-GlcNAc, MgCl_2_ (20 mM), and appropriate amounts of purified HpLgtA (0.3 mg/mL).

#### α1-2 Fucosylation by Hm2FT/WbsJ

The reaction system contains Tris-HCl buffer (50 mM, pH 8.0), an acceptor (5–10 mM), 2 equiv of sugar donor GDP-Fuc, MgCl_2_ (20 mM), and appropriate amounts of purified Hm2FT (0.5 mg/mL)/WbsJ (0.5 mg/mL).

#### α1-3 Fucosylation by FUT3/FUT5/Hp3/4FT

The reaction system contains Tris-HCl buffer (50 mM, pH 8.0), an acceptor (5–10 mM), 2 or 4 (for fucosylation of extended structures) equivalents of sugar donor GDP-Fuc, MgCl_2_ (20 mM), and appropriate amounts of purified FUT3 (0.3 mg/mL)/FUT5 (0.5 mg/mL)/Hp3/4FT (0.5 mg/mL).

#### α2-3 Sialylation by PmST1m

The reaction system contains Tris-HCl buffer (100 mM, pH 8.0), an acceptor (5–10 mM), 2 equiv of sugar donor CMP-Neu5Ac/Neu5Gc, MgCl_2_ (20 mM), and appropriate amounts of purified PmST1m (0.2 mg/mL).

#### α2-6 Sialylation by Human ST6GalNAc6

The reaction system contains Tris-HCl buffer (100 mM, pH 8.0), an acceptor (5–10 mM), 2 equiv of sugar donor CMP-Neu5Ac/Neu5Gc, and appropriate amounts of purified ST6GalNAc6 (0.05 mg/mL).

#### Galactose-3-*O*-sulfation by Gal3ST2

The reaction system contains MES buffer (100 mM, pH 6.5), PAPS (20 mM), ATP (20 mM), MgCl_2_ (20 mM), an acceptor (10 mM), and appropriate amounts of purified Gal3ST2 (0.2 mg/mL).

All reactions were incubated for 4 h to overnight and monitored by HPLC until the conversion rate exceeded 95%. The reactions were then quenched by adding an equal volume of ethanol and stored at 4 °C for 15 min. The mixtures were subsequently centrifuged to remove any precipitates, and supernatants were concentrated using a rotary evaporator and subjected to C18 and P2/P4 column chromatography for purification. Product-containing fractions were collected and lyophilized for HRMS and NMR characterization. Detailed procedures for reaction monitoring, product purification, and characterization are provided in the Supporting Information.

### Glycan Microarray Fabrication and Assay

All glycans with a Cbz tag were reduced overnight by Pd/C hydrogenation at room temperature to expose the amine group. MALDI-TOF was used to monitor the reactions. Pd/C was removed by centrifugation, and the toluene produced during hydrogenation was removed by a vacufuge concentrator. The glycan microarray was fabricated according to the guidelines of MIRAGE as summarized in Table S1. Glycans were prepared at a concentration of 100 μM in the printing buffer (300 mM phosphate, pH 8.5) and printed on Z-biotech Multivalent Microarray Slides (Z Biotech), each in six replicates. Noncontact printing was performed at room temperature with a humidity of 65% using a sciFLEXARRAYER S3 ultralow volume dispensing system (Scienion) with a PDC 70 Piezo Dispense Capillary, and 16 subarrays were printed on each slide. After overnight dehumidification at room temperature, the slides were blocked with 50 mM ethanolamine in 100 mM Tris buffer (pH 9.0) for 1 h. Blocked slides were then washed with Milli-Q water twice, dried, and stored desiccated at −20 °C until use. The printing buffer was printed as a negative control; Biotin-PEG3-Amine (CAS: 138529-46-1) was printed as a positive control.

All biotinylated lectins were purchased from Vector Laboratories, Inc. (Newark, CA), Lab (Burlingame, CA). Details of all tested antibodies are given in Table S2. Biotinylated lectins AAL, UEA-I, and STL were detected by Cy5-streptavidin (0.5 μg/mL). Assays for HE-193 and HEB-29 were detected by goat anti-mouse IgM­(H+L) second antibody conjugated with Alexa Fluor 555 in a concentration of 5 μg/mL. Assays for MVT-5873, Siglec-7, Siglec-9, P-Selectin, and E-Selectin were detected by goat anti-human IgG (H+L) secondary antibody conjugated with Alexa Fluor 647 in a concentration of 5 μg/mL. Assays for 7LE, 2-25LE, FH7, 17-206, and 1116-NS-19-9 were detected by goat anti-mouse IgG­(H+L) second antibody conjugated with Alexa Fluor 647 in a concentration of 5 μg/mL. Assay for LecB was detected by Anti-His Tag Alexa Fluor 647-conjugated Antibody in 10 μg/mL.

## Results and Discussion

### FUT3 Catalyzes the Construction of DSLe^A^

Despite recent progress in synthesizing type-1 determinant-bearing glycans, particularly HMOs,^35–40^ the enzymatic synthesis of DSLe^A^ and related epitopes, including 6-sialyl-Lewis A (6SLe^A^), 6-sialyl-Lewis B (6SLe^B^), and *N*-glycolyl disialyl-Lewis A (DGLe^A^) (Figure 1), remains elusive. These determinants share three distinct glycosidic linkages on GlcNAc: β1-3 galactosylation, α1-4 fucosylation, and α2-6 sialylation. The galactosylation step clearly occurs first in the biosynthetic pathway to generate the type-1 LN backbone, and it was proposed that FUT3-catalyzed α1-4 fucosylation occurs after α2-6 sialylation catalyzed by the sialyltransferase ST6GalNAc6.^14^ Indeed, our recent work has demonstrated that neither ST6GalNAc6 nor enzymes with the same activity (ST6GalNAc4, ST6GalNAc5) could sialylate SLe^A^ to generate DSLe^A^.^36^ Unfortunately, attempts to prepare DSLe^A^-containing HMO disialyl-lacto-*N*-fucopentaose II (DS-LNF II or named FDS-LNT I, Figure 1) from disialyl-lacto-*N*-tetraose (DSLNT) using a promiscuous *H. pylori* α1-3/4FucT (Hp3/4FT)^29^ was unsuccessful.^36,39^

To tackle this challenge, we expressed and purified two human α1-3/4FucTs (FUT3 and FUT5, Figure S1) and evaluated their activity toward DSLNT (**1**), with Hp3/4FT as a negative control (Figure 2A). As expected, no product formation was detected in the Hp3/4FT-catalyzed reaction. Conversely, new peaks were observed in the HPLC profiles of both FUT5- and FUT3-catalyzed reactions, albeit with different retention times (Figure 2B). LC-MS analysis of these peaks revealed the same *m*/*z* value of 1625.66 [M – H^+^]^−^, corresponding to monofucosylated DSLNT. The product of FUT5-catalyzed reaction (**2**) had the same retention time (*T*_R_ = 13.14 min) as DS-LNF V^36^ (Figure 2B), indicating that FUT5 catalyzes the α1-3 fucosylation of Glc to generate DS-LNF V. Differently, product **3** yielded from FUT3-catalyzed reaction eluted at *T*_R_ = 13.85 min, suggesting a distinct fucosylation, presumably α1-4 fucosylation on GlcNAc, which generates DS-LNF II. To confirm this, **3** was synthesized from DSLNT by using FUT3 (85% yield, 7.2 mg) and analyzed by NMR (Supporting Information). Merged HMBC and HSQC-TOCSY spectra revealed proton signals for Glc, Gal1, GlcNAc, Gal2, and Fuc, and the correlation of C1 on Fuc with H4 on GlcNAc (δ 3.79, 72.05) demonstrated that Fuc is attached to C4 of GlcNAc rather than to the Glc (Figure 2C). Notably, prolonged incubation with FUT3 or FUT5 did not produce difucosylated byproducts, highlighting their regioselectivity for GlcNAc and Glc on DSLNT, respectively.

**Figure 2:**
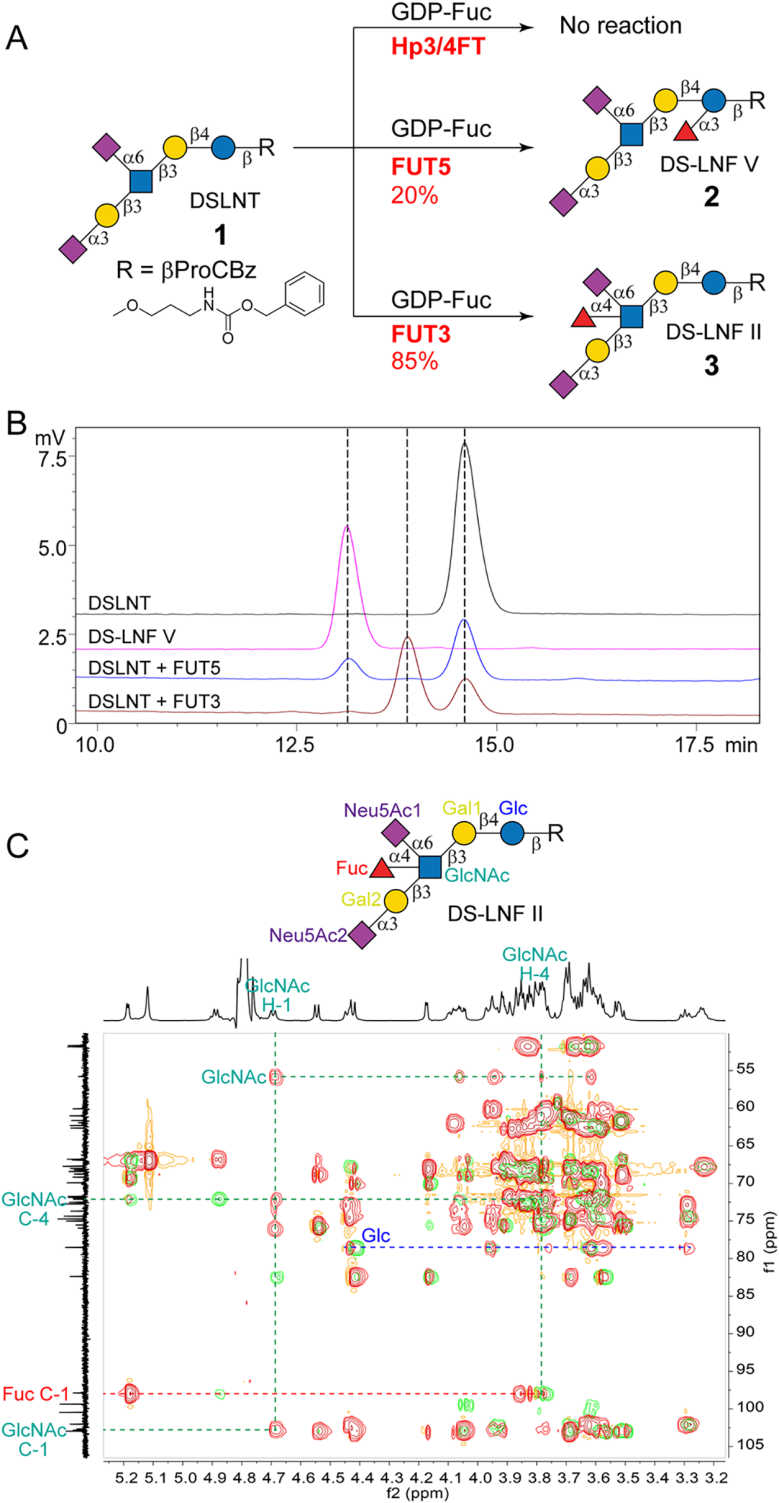
Activity assay of FucTs toward DSLNT and NMR characterization of product **3** from FUT3-catalyzed reaction. (A) Activity assay of FucTs using DSLNT as an acceptor (5 mM) in the presence of 2 equiv of GDP-Fuc and yields in mg scale synthesis; (B) HPLC profiles of FUT3- and FUT5-catalyzed reactions after 24 h of incubation at 37 °C; (C) Merged HSQC-TOCSY (in red) and HMBC (in green) spectra of **3** confirmed the location of Fuc on the C4 of GlcNAc.

### Exploring the Synthetic Capability of FucTs for Divergent Enzymatic Synthesis of Type-1 Determinants

Having determined the synthesis of DS-LNF II, we then focused on developing a divergent enzymatic strategy for the efficient production of all type-1 glycan determinants. Since most type-1 determinants contain α1-2 or α1-4 linked Fuc, we systematically surveyed the synthetic capability and efficiency of three α1-3/4FucTs (Hp3/4FT, FUT3, FUT5) and five enzymes with α1-2FucT activity (Figure 3). Three of the α1-2FucTs that demonstrated good recombinant expression levels in *E. coli* were of bacterial origin, from *H. mustelae* (Hm2FT),^26^ *E. coli* O128 (WbsJ),^50^ and *T. elongatus* BP-1 (Te2FT),^28^ respectively. Among these, Hm2FT has been applied to synthesize HMOs with both type-1 and type-2 (Galβ1-4GlcNAc) determinants.^26,36,39^ WbsJ tolerates several β-galactosides,^50^ whereas Te2FT only acts on β1-3 galactosides including type-1 LN and Galβ1-3GalNAc.^28^ Another α1-2FucT we tested, human FUT1, was believed to be responsible for the biosynthesis of type-2 H-antigen,^55^ although other reports suggested a broader substrate specificity.^56^ Finally, we evaluated α1-2 colitosyltransferase from *E. coli* O55 (WbgN),^25^ which naturally uses GDP-colitose (3-deoxy-Fuc) as the donor but also well-tolerates GDP-fucose. Uniquely, WbgN exhibited a strict acceptor specificity, exclusively fucosylating type-1 LN.^25^

**Figure 3:**
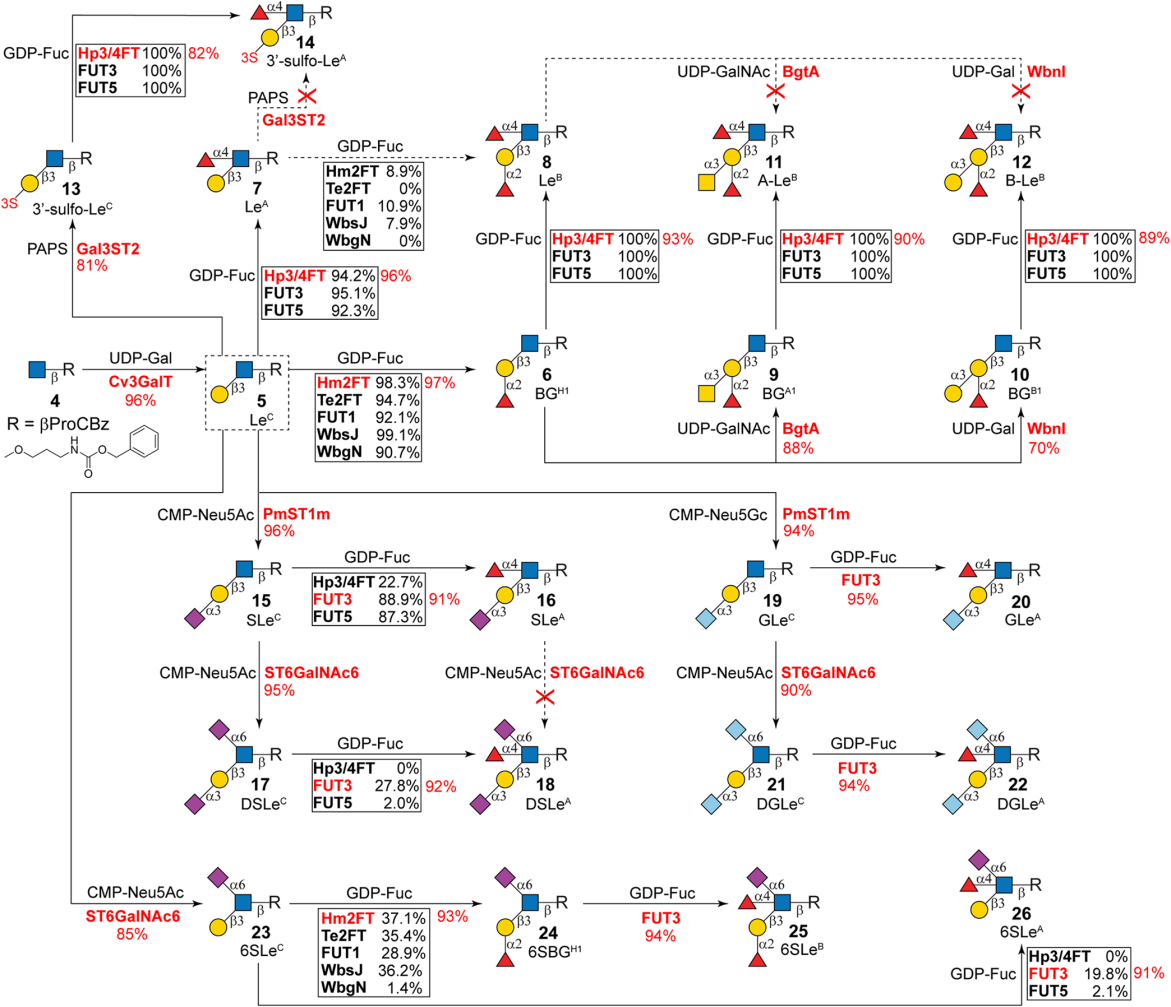
Exploring the synthetic capabilities of 8 fucosyltransferases (FucTs) and divergent enzymatic synthesis of basic type-1 determinants. Cv3GalT, β1-3 galactosyltransferase from *C. violaceum*; Hm2FT, α1-2FucT from *H. mustelae*; Te2FT, α1-2FucT from *T. elongatus* BP-1; FUT1, human fucosyltransferase 1; WbsJ, α1-2FucT from *E. coli* O128; WbgN, α1-2 colitosyltransferase from *E. coli* O55; Hp3/4FT, α1-3/4FucT from *H. pylori*; FUT3, human fucosyltransferase 3; FUT5, human fucosyltransferase 5; BgtA, α1-3-*N*-acetylgalactosaminyltransferase from *H. mustelae*; WbnI, α1-3 galactosyltransferase from *E. coli* O86; Gal3ST2, human galactose-3-O-sulfotransferase 2; PmST1m, α2-3 sialyltransferase mutant M144D from *P. multocida*; ST6GalNAc6, human ST6 *N*-acetylgalactosaminide α2-6 sialyltransferase 6; UDP-Gal, uridine-diphosphate-galactose; UDP-GalNAc, uridine-diphosphate-*N*-acetylgalactosamine; GDP-Fuc, guanosine-diphosphate fucose; CMP-Neu5Ac, cytidine-monophosphate *N*-acetylneuraminic acid; CMP-Neu5Gc, cytidine-monophosphate *N*-glycolylneuraminic acid; PAPS, 3′-phosphoadenosine-5′-phosphosulfate. Substrate specificity reactions of FucTs were carried out in 50 mL systems including 50 mM Tris-HCl (pH 8.0), 10 mM acceptor, 20 mM GDP-Fuc, 20 mM MgCl_2_, and appropriate amounts of FucTs. Reactions were allowed to proceed at 37 °C for 4 h and subjected to HPLC analysis (conversion rates are shown in rectangular boxes). Enzymes used in glycan synthesis and their corresponding yields are highlighted in red. Excess enzymes and extended incubation times were applied to achieve high yields in glycan synthesis.

To facilitate the synthetic capability study and the production of type-1 determinants, we first carried out the chemoenzymatic synthesis of type-1 LN (Figure 3). This was achieved via β1-3 galactosylation of a chemically synthesized precursor **4** (2-*O*-(*N*-benzyloxycarbonyl) aminopropyl β-*N*-acetylglucosaminide, GlcNAcβProCbz),^57^ catalyzed by β1-3 galactosyltransferase from *C. violaceum* (Cv3GalT)^31^ in the presence of sugar nucleotide UDP-Gal. Product purification was achieved by one-step C18 cartridge chromatography facilitated by the hydrophobic Cbz group, affording 203 mg of type-1 LN (**5**) with an excellent yield of 96%. With **5** in hand, fucosylation reactions were performed to access enzyme requirements for the synthetic capability studies of FucTs. Briefly, 50 mL of reactions contained 50 mM Tris-HCl (pH 8.0), 10 mM acceptor (**5**), 20 mM donor (2.0 equiv, GDP-Fuc), 20 mM MgCl_2_, and FucTs incubated at 37 °C for 4 h. Enzyme amounts are adjusted to ensure >90% conversion of **5**, monitored by HPLC (Supporting Information). The determined enzyme amounts were utilized in all subsequent fucosylation assays. Considering their high expression levels in *E. coli*,^26,29^ Hm2FT and Hp3/4FT were chosen for the synthesis of BG^H1^ (**6**) and Le^A^ (**7**), affording 51 mg of **6** (97% yield) and 7.3 mg of **7** (96% yield), respectively.

The enzymatic synthesis of Le^B^ has typically been conducted by a sequential α1-2 fucosylation and then α1-4 fucosylation of type-1 LN.^29,31–35,38–40^ Attempts to alter the fucosylation sequence failed in one case^29^ but resulted in satisfactory yields in another.^34^ With both **6** and **7** in hand (Figure 3), we evaluated the efficiency of the eight FucTs to synthesize Le^B^ (**8**). As shown in Figure 3, all three α1-3/4FucTs catalyzed complete conversions of **6** to **8**, indicating that α1-2 fucosylation had no impact on their enzymatic activity. In contrast, α1-2FucT-catalyzed reactions resulted in minimal (7.9–10.9%) to no conversions of **7** (Figure S2), suggesting that the α1-4 fucosylation strongly hindered the activity of α1-2FucTs. Consequently, Hp3/4FT was selected to prepare Le^B^ (**8**) from **6** with an excellent yield of 93% (12 mg). Notably, partially purified (one-step biogel P2-chromatography to >70%) sugar nucleotides were employed in all synthetic steps, to enhance conversions and simplify purification.

The attempts to generate A-Le^B^ (**11**) and B-Le^B^ (**12**) directly from Le^B^ (**8**) using α1-3-*N*-acetylgalactosaminyltransferase from *H. mustelae* (BgtA)^50^ and α1-3 galactosyltransferase from *E. coli* O86 (WbnI)^51^ were unsuccessful, suggesting that both enzymes are blocked by the internal α1-4 fucosylation. Alternatively, type-1 blood group A (BG^A1^, **9**) and B antigen (BG^B1^, **10**) were prepared from **6** with satisfactory yields (88 and 70%), catalyzed by BgtA and WbnI, respectively. Subsequent assays of all three α1-3/4FucTs showed complete conversions of **9** and **10** (Figure 3), further confirming the broad substrate scope of these enzymes.^32^ Accordingly, A-Le^B^ (**11**) and B-Le^B^ (**12**) were synthesized from **9** and **10** using Hp3/4FT, achieving 90 and 89% yields, respectively.

Additionally, human Gal3ST2 (expressed as reported^58^) was employed to sulfate type-1 LN, affording 3′-sulfo-Le^C^ (**13**) with 81% yield (11 mg). Notably, **13** is well-recognized by all α1-3/4FucTs, and 3′-sulfo-Le^A^ (**14**) was synthesized from **13** with a yield of 82% (7.5 mg) using Hp3/4FT. Conversely, attempts to generate **14** from **7** through Gal3ST2-catalyzed sulfation were unsuccessful, indicating that it does not tolerate α1-4 fucosylation. Collectively, our results demonstrate that α1-4 fucosylation on the GlcNAc of type-1 LN generally blocks modifications (including α1-2 fucosylation, α1-3 galactosylation, α1-3GalNAcylation, and 3-*O*-sulfation) on the distal Gal residue, whereas these modifications are well-tolerated by enzymes responsible for α1-4 fucosylation.

The synthesis of sialylated type-1 determinants is illustrated in Figure 3. SLe^C^ (**15**) and DSLe^C^ (**17**) were prepared sequentially, catalyzed by *P. multocida* α2-3 sialyltransferase mutant M144D (PmST1m)^59^ and human ST6GalNAc6,^36^ respectively, with excellent yields of 96 and 95%. Subsequent synthetic capability assays revealed that FUT3 and FUT5 catalyzed high conversion levels of **15**, whereas Hp3/4FT gave a low conversion rate of 22.7% (Figure S3). Meanwhile, FUT3 facilitated a low yet significant conversion (27.8%) of **17** to **18**, whereas Hp3/4FT and FUT5 showed negligible or no activity (Figure S4), consistent with prior attempts for the synthesis of DS-LNF II.^36,39^ Accordingly, FUT3 (excess amounts and prolonged incubation time) was employed to produce SLe^A^ (**16**) and DSLe^A^ (**18**), providing 91% (5.2 mg) and 92% yields (7.1 mg), respectively. Attempts to convert **16** to **18** using ST6GalNAc6 were unsuccessful, aligning with our recent report.^36^ Similarly, type-1 determinants bearing *N*-glycolylneuraminic acid (Neu5Gc) residues were synthesized by using CMP-Neu5Gc as the sialylation donor, affording GLe^C^ (**19**), GLe^A^ (**20**), DGLe^C^ (**21**), and DGLe^A^ (**22**) with excellent yields of 90–95% (Figure 3). Furthermore, 6SLe^C^ (**23**) was generated from **5**, catalyzed by ST6GalNAc6 in a very good yield of 85% (31 mg). It was then used to assess the synthetic capability of the eight FucTs. All α1-2FucTs except WbgN exhibited relatively low yet significant fucosylation (28.9–37.1%) of **23** (Figure 3 **and** S5), suggesting that the α2-6 sialylation is not favorable but remains tolerated by these enzymes. Additionally, only FUT3 catalyzed the α1-4 fucosylation of **23** at a noticeable rate of 19.8%, as expected. Nevertheless, Hm2FT and FUT3 were employed to synthesize 6SBG^H1^ (**24**) and 6SLe^A^ (**26**) with yields of 93% (15 mg) and 91% (6.4 mg), respectively. Lastly, 6SLe^B^ (**25**) was prepared from **24** via FUT3-catalyzed reaction with an excellent yield of 94% (9.2 mg). Taking together with our recent report,^36^ FUT3 and tested α1-2FucTs except WbgN tolerate α2-6 sialylation on the GlcNAc residue; however, α1-2/4 fucosylation hinders the activity of ST6GalNAc6.

### Exploring Internal Fucosylation for Divergent Enzymatic Synthesis of Extended Type-1 Determinants

We next sought to preparing a library of fucosylated type-1 tetrasaccharide (Le^C^–Le^C^) including TACAs Le^B^–Le^A^ and Le^B^–Le^A^. As shown in Figure 4, trisaccharide **27** was first obtained (43 mg, 95% yield) from **5**, catalyzed by *H. pylori* α1-3-*N*-acetylglucosaminyltransferase (HpLgtA)^60^ and then converted to Le^C^–Le^C^ (**29**) by Cv3GalT-catalyzed β1-3 galactosylation with 96% yield. The α1-2 fucosylation assay in the presence of four equivalents of GDP-Fuc revealed that Te2FT, FUT1, and WbsJ catalyzed efficient monofucosylation (*m*/*z* of 1086.20) of **29** (93.1–100% conversions, Figure S6). Product **30** was synthesized using WbsJ in an excellent yield of 96% (9.5 mg) and subjected to NMR analysis, which confirmed α1-2 fucosylation at the terminal Gal (Supporting Information). On the other hand, Hm2FT and WbgN attached up to two Fuc residues (*m*/*z* of 1232.65) (Figure S6). In addition, both Hm2FT and WbgN catalyzed low yet significant (29.6 and 32.4%) α1-2 fucosylation of **27** to generate **28**, whereas reactions catalyzed by Te2FT, FUT1, and WbsJ only resulted in trace conversion rates of 2.6–5.7% (Figure S7). These results suggest that Hm2FT and WbgN prefer terminal Gal while still tolerate internal Gal; however, Te2FT, FUT1, and WbsJ can barely recognize internal Gal residues. The α1-4 fucosylation assay toward both **29** and **30** produced difucosylated (*m*/*z* of 1233.70 for **29**, 1378.25 for **30**) and monofucosylated (*m*/*z* of 1086.20 for **29**, 1232.30 for **30**) products (Figures 4, S8, and S9), along with increased difucosylation products in prolonged incubations. Accordingly, Hp3/4FT was employed to prepare Le^A^–Le^A^ (**31**) and Le^B^–Le^A^ (**32**), with excellent yields of 92% (4.5 mg) and 90% (5.3 mg), respectively.

**Figure 4:**
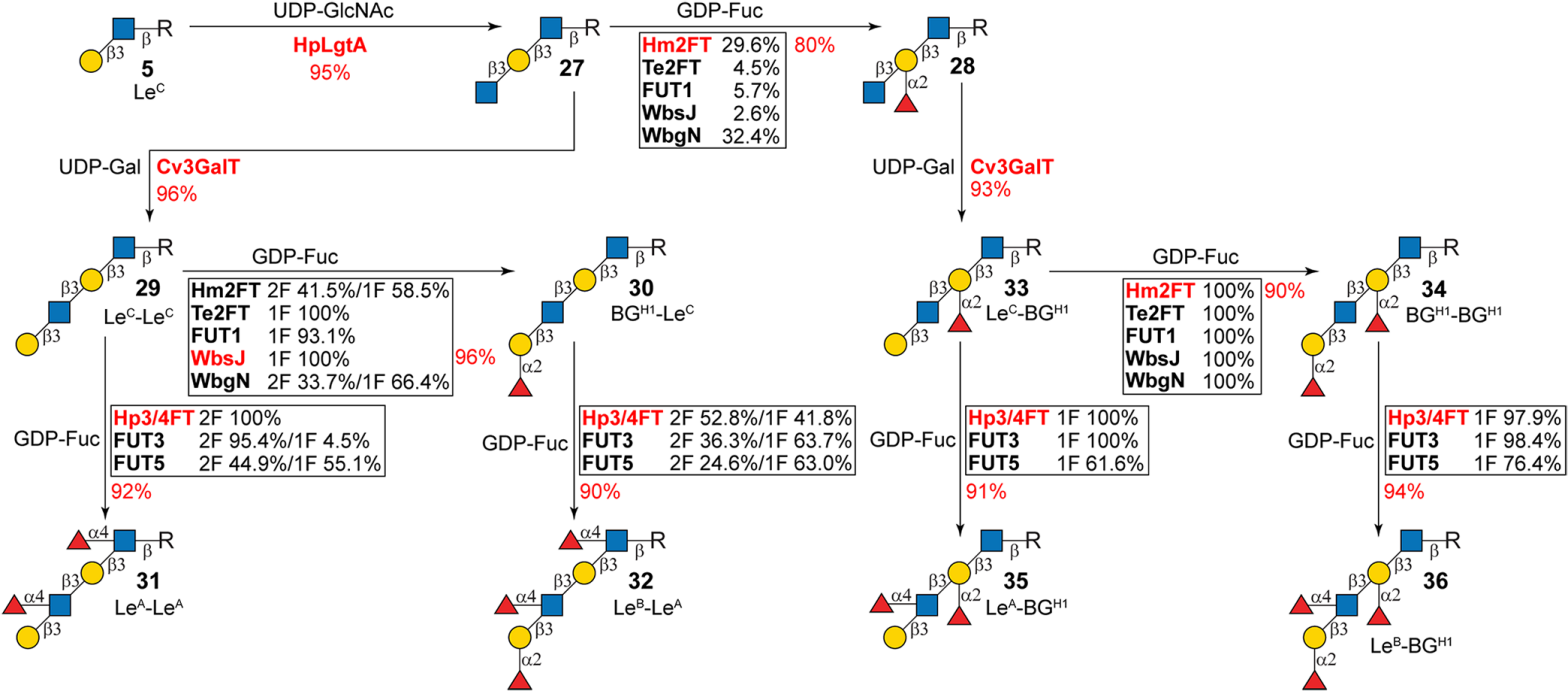
Exploring internal fucosylation and divergent enzymatic synthesis of extended type-1 determinants. HpLgtA, β1-3-*N*-acetylglucosaminyltransferase from *H. pylori*; UDP-GlcNAc, uridine-diphosphate-*N*-acetylglucosamine. Substrate specificity reactions of FucTs were carried out in 50 mL systems including 50 mM Tris-HCl (pH 8.0), 5 mM acceptor, 20 mM GDP-Fuc, 10 mM MgCl_2_, and appropriate amounts of FucTs. Reactions were allowed to proceed at 37 °C for 4 h and subjected to HPLC analysis (conversion rates are shown in rectangular boxes). Enzymes used in glycan synthesis and their corresponding yields are highlighted in red. Excess enzymes and extended incubation times were applied to achieve high yields of glycan synthesis.

To prepare an extended type-1 determinant with internal α1-2 fucosylation (**33**–**36**), **28** was first obtained using Hm2FT in 80% yield (25 mg), a portion of which was then converted to **33** by Cv3GalT in an excellent yield of 93% (22 mg) (Figure 4). As expected, all α1-2FucTs catalyzed complete fucosylation of the terminal Gal on **33** to generate dimeric BG^H1^ (BG^H1^–BG^H1^, **34**). Surprisingly, α1-4 fucosylation assays of all three α1-3/4FucTs toward both **33** and **34** resulted in the attachment of only one Fuc (*m*/*z* of 1233.49 for **33**, 1379.56 for **34**). Milligram-scale preparation of these products was then achieved through Hp3/4FT-catalyzed reactions, yielding **35** and **36** in excellent yields of 91% (4.3 mg) and 94% (5.1 mg), respectively. 2D NMR analysis confirmed that the newly attached Fuc residue was located on the distal GlcNAc (Supporting Information), identifying **35** and **36** as Le^A^–BG^H1^ and Le^B^–BG^H1^, respectively. This result indicates that unlike α1-4 fucosylation on the terminal type-1 unit, that on internal type-1 unit is blocked by the α1-2 fucosylation on the internal Gal residue. Notably, attempts to synthesize a yet unidentified tetra-fucosylated structure Le^B^–Le^B^ through either internal α1-2 fucosylation of **32** or α1-4 fucosylation of **36** were unsuccessful.

### Glycan Microarray Analysis

All type-1 determinants are prepared by starting from GlcNAc with the same linker, providing a consistent platform for functional studies, particularly for comparing different type-1 epitopes. To showcase their utility, we fabricated a unique glycan microarray on multivalent glass slides (Supporting Information), which includes 32 type-1 glycans prepared in this study and four type-2 reference epitopes (Figure S10). The array was validated using lectins and monoclonal antibodies (mAbs) against blood group ABH antigens (Figure S11) before being utilized to probe the binding of anti-Lewis antigen mAbs and various GBPs.

As shown in Figure 5A, anti-Le^A^ mAb clone 7LE, reported to be specific for Le^A^ antigen,^61^ displayed strong binding to Le^A^ (**7**) and extended determinants presenting terminal Le^A^ (**31**, **35**), but no binding to **32**, indicating that 7LE does not recognize internal Le^A^ motifs. Anti-Le^B^ mAb clone 2-25LE recognized Le^A^ (**7**), Le^B^ (**8**), and extended type-1 determinants featuring terminal Le^A^ or Le^B^ (**31**, **32**, **35**, **36**) (Figure 5B), in agreement with its broader specificity.^61^ Interestingly, 2-25LE also displayed robust binding to **25** and **26**, indicating a tolerance of α2-6 sialylation on GlcNAc.

**Figure 5:**
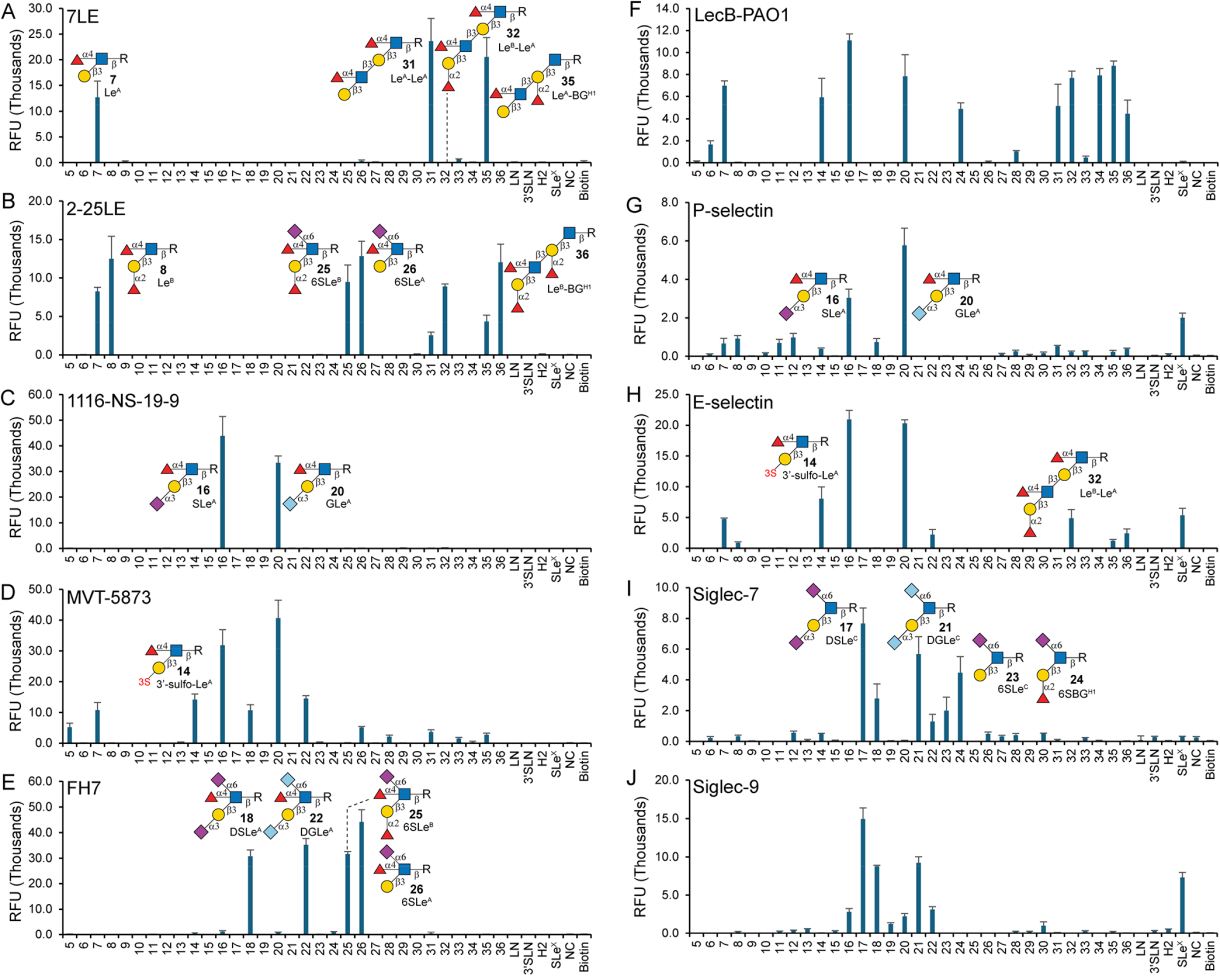
Microarray analysis of anti-Lewis antigen mAbs and various GBPs using type-1 glycan determinants. (A) Le^A^ mAb clone 7LE (10 μg/mL); (B) Le^B^ mAb clone 2-25LE (10 μg/mL); (C) SLe^A^ mAb clone 1116-NS-19-9 (10 μg/mL); (D) SLe^A^ mAb clone MVT-5873 (10 μg/mL); (E) DSLe^A^ mAb clone FH7 (10 μg/mL); (F) LecB (10 μg/mL); (G) P-selectin (20 μg/mL); (H) E-selectin (20 μg/mL); (I) Siglec-7 (50 μg/mL); and (J) Siglec-9 (25 μg/mL). LN, type-2 LacNAc (Galβ1-4GlcNAc); 3′SLN, Neu5Acα2-3Galβ1-4GlcNAc; H2, type-2 H-antigen (Fucα1-2Galβ1-4GlcNAc); SLe^X^, sialyl-Lewis X (Neu5Acα2-3Galβ1-4­(Fucα1-3)­GlcNAc); NC, printing buffer as negative control; Biotin, Biotin-PEG3-Amine (0.01 mg/mL). The *x*-axis shows glycans, and the *y*-axis shows relative fluorescence unit (RFU) readouts using goat anti-mouse IgG second antibody AF647-conjugate for 7LE, 2-25LE, 1116-NS-19-9, and FH7 at 5 μg/mL, goat anti-human IgG second antibody AF647-conjugate for MVT-5873, Siglec-7, Siglec-9, P-selectin, and E-selectin at 5 μg/mL, and anti-His Tag Alexa Fluor 647-conjugated Antibody in 10 μg/mL. Data are presented as mean values. Error bars represent the standard deviation of 6 replicates.

When probing anti-SLe^A^ mAbs, 1116-NS-19-9 ^62^ showed strict specificity for SLe^A^ (**16**) and its Neu5Gc counterpart GLe^A^ (**20**) (Figure 5C), whereas MVT-5873 (also known as HuMab-5B1 in clinical trial) not only bound to **16** and **20** but also exhibited moderate avidity for Le^A^ (**7**), 3′-sulfo-Le^A^ (**14**), DSLe^A^ (**18**), and DGLe^A^ (**22**). Notably, MVT-5873 showed weak binding to unsubstituted type-1 LN (**5**) and extended type-1 chains bearing terminal Le^A^ (**31**, **35**) or internal α1-2 fucosylation (**26**, **28**, and **32**), indicating a much broader epitope recognition than 1116-NS-19-9. Anti-DSLe^A^ clone FH7,^15^ widely recognized as specific for DSLe^A^, strongly bound to DSLe^A^ (**18**) and its Neu5Gc counterpart **22**, yet it also bound to **25** and **26** with comparable avidities (Figure 5E), suggesting a primary ligand of 6-sialyl-Lewis A (6SLe^A^). Notably, none of these mAbs could distinguish between Neu5Ac and Neu5Gc on sialylated type-1 determinants.

*Pseudomonas aeruginosa* LecB (PA-IIL) is a fucose-specific extracellular GBP that functions as a virulence factor and is essential for adhesion and biofilm formation.^63^ LecB from the PAO1 strain (LecB-PAO1) was reported to recognize various fucosylated glycans, with the highest apparent binding to poly-LacNAc chains presenting terminal type-2 H-antigen (H2) or Lewis X/Y (Le^X/Y^) epitopes.^64^ However, our results showed the strongest binding to Le^A^ (**7**), 3′-sulfo-Le^A^ (**14**), SLe^A^ (**16**), and GLe^A^ (**20**) but no binding to H2 and SLe^X^ (Figure 5F), suggesting a preferred ligand of α1-4Fuc, consistent with a prior observation.^65^ Interestingly, LecB-PAO1 did not bind to **18**, **22**, **25**, and **26**, indicating that internal α2-6Neu5Ac hinders the recognition of α1-4Fuc. In addition, LecB-PAO1 showed moderate bindings to extended type-1 structures containing two or three Fuc residues, regardless of the presence of α1-4Fuc (Figure 5F, compounds **31**, **32**, and **34**–**36**), suggesting a more complex mode of recognition.

Selectins are transmembrane glycoproteins containing C-type lectin domains that specifically bind to SLe^X^ and SLe^A^ presenting structures.^66^ They play essential roles in both chronic and acute inflammatory processes and lymphocyte homing.^66^ We investigated P- and E-selectin on a type-1 determinant microarray. As expected, P-selectin showed overall weak but specific binding to SLe^X^, SLe^A^ (**16**), and its Neu5Gc counterpart GLe^A^ (**20**) (Figure 5G), consistent with its known preferred ligands of O-glycans.^67^ E-selectin, on the other hand, bound strongly to SLe^A^ and GLe^A^, with approximately 4-fold higher avidity compared to that to SLe^X^ (Figure 5H). In addition, moderate to low bindings were observed for 3′-sulfo-Le^A^ (**14**), Le^A^ (**7**), and TACA trifucosyl-Le^B^ (**32**). Collectively, both P-selectin and E-selectin show a preference for SLe^A^ over SLe^X^ and are unable to distinguish between Neu5Ac and Neu5Gc.

Sialic acid-binding immunoglobulin-like lectins (Siglecs) are GBPs primarily expressed on immune cell surfaces, where they play key regulatory roles.^68^ Siglec-7 and Siglec-9, two closely related members of the Siglec family, have garnered significant interest in cancer immunotherapy, as a variety of cancers express ligands of both Siglecs.^69^ We evaluated the glycan recognition profiles of Siglec-7 and -9 using a type-1 glycan microarray. As shown in Figure 5I, Siglec-7 recognized DSLe^C^ (**17**), contrary to our previous observations using DSLe^C^-presenting HMOs,^36^ but consistent with other reports.^17,70^ It also bound to DSLe^A^ (**18**) and compounds **23** and **24**, echoing previous reports.^36,70^ Notably, binding to **18** was significantly reduced compared to **17**, suggesting an inhibitory effect of α1-4Fuc on GlcNAc for Siglec-7 recognition. Siglec-9 showed a similar binding profile to DSLe^C^ and DSLe^A^ (Figure 5J), consistent with prior reports.^36,70^ Importantly, both Siglec-7 and Siglec-9 recognized Neu5Gc counterparts of DSLe^C^ and DSLe^A^ (**18** and **22**), but with significantly reduced binding avidities (Figure 5I,J), suggesting a preference for Neu5Ac. Collectively, Siglec-7 and -9 show similar binding profiles to disialyl-Lewis epitopes (**17**, **18**, **21**, **22**), with α1-4 fucosylation and Neu5Gc substitution both leading to diminished bindings.

## Conclusion

In summary, we have developed a robust and divergent enzymatic platform for assembling a 30-member library of type-1 glycan determinants, including elusive structures such as disialyl-Lewis A and trifucosyl-Lewis B. We also achieved the first enzymatic synthesis of HMO structure DS-LNF II. Systematic evaluation of eight robust FucTs, together with other glycosyltransferases and sulfotransferases, revealed notably broad substrate tolerance of α1-3/4FucTs, especially FUT3. Conversely, once α1-4 fucosylation was introduced, it generally hinders subsequent modifications acting on the type-1 unit, including α1-2 fucosylation, α2-6 sialylation, sulfation, etc. These insights into the substrate scope of glycosyltransferases and their interplay (Table 1) provide guidelines for the chemoenzymatic synthesis of complex glycans and glycoconjugates.

**Table 1:** Substrate Specificities of Glycosyltransferases and Gal3ST2 toward Type-1 Structures^a^

		type-1 glycan determinants												extended type-1 glycan determinants					
enzymes		Le^C^	Le^A^	3′-sulfo-Le^C^	Le^B^	BG^H1^	BG^A1^	BG^B1^	SLe^C^	SLe^A^	DSLe^C^	6SLe^C^	6SBG^H1^	Le^C^–Le^C^	BG^H1^–Le^C^	Le^C^–BG^H1^	BG^H1^–BG^H1^	Le^B^–Le^A^	Le^B^–BG^H1^
α1-2 FucT	Hm2FT	Y	L									M		Y2				N	
	Te2FT	Y	N									M		Y1				N	
	FUT1	Y	L									M		Y1				N	
	WbsJ	Y	L									M		Y1				N	
	WbgN	Y	N									L		Y2				N	
α1-3/4FucT	Hp3/4FT	Y		Y		Y	Y	Y	M		N	N		Y2	Y2	Y1	Y1		N
	FUT3	Y		Y		Y	Y	Y	Y		M	M	M	Y2	Y2	Y1	Y1		N
	FUT5	Y		Y		Y	Y	Y	Y		L	L		Y2	Y2	Y1	Y1		N
ST6GalNAc6		Y	N		N	L			Y	N									
Gal3ST2		Y	N																
BgtA					N	Y													
WbnI					N	Y													

a Y, tolerate with a conversion rate >60%; M, tolerate with a conversion rate between 15 and 40%; L, tolerate with a conversion rate <15%; N, does not tolerate; Y1, tolerate with maximum one fucosylation; Y2, tolerate with up to two fucosylation; blank, not tested or not applicable.

Subsequent glycan microarray assays uncovered previously unrecognized subtleties of the specificity of anti-Lewis antigen antibodies and various glycan-binding proteins. For example, the anti-Le^A^ mAb 7LE strictly recognizes terminal Le^A^ epitopes, while the anti-Le^B^ mAb 2-25LE binds to both Le^A^ and Le^B^ and tolerates internal α2-6 sialylation. The anti-SLe^A^ mAb 1116-NS-19-9 strictly recognizes sialyl-Le^A^, whereas another anti-SLe^A^ mAb, MVT-5873, exhibits much broader specificity. We also identified 6SLe^A^ as the ligand for anti-DSLe^A^ mAb FH7. Furthermore, Siglec-7 and -9 exhibited similar binding profiles to disialyl-Lewis epitopes with both α1-4 fucosylation and Neu5Gc substitution resulting in reduced binding. These findings provide valuable guidelines for future academic and clinical application of anti-Lewis antibodies and glycan-binding proteins.

Collectively, the divergent enzymatic strategy, the comprehensive library of type-1 glycan determinants, and the unique glycan microarray provide valuable toolkits for complex glycan synthesis, functional glycomics studies, and therapeutic development targeting type-1 determinant-presenting glycans.

## Supplementary Material



## Notes

Supporting Information figures, microarray analysis, analytical data including HPLC profiles, and mass and NMR spectra of synthesized glycans (PDF)
