# Supplementary material for: Divergent Enzymatic Synthesis of a Comprehensive Type‑1 Glycan Determinant Library
Source: ACS Catal. 2025 Dec 1;15(24):20764–75. doi: 10.1021/acscatal.5c06702 (PMC12723683; doi:10.1021/acscatal.5c06702)
Supplement: Supplementary file 1 [file cs5c06702_si_001.pdf]

SUPPORTING INFORMATION

Divergent Enzymatic Synthesis of a Comprehensive Type-1 Glycan Determinant Library

Guitao Bai, Tangliang Shen, MohammadHossein Shabahang, Shumin Bao, Shuquan Fan, Hongshuai Lv, and Lei Li*

Department of Chemistry and Center for Diagnostics & Therapeutics, Georgia State University, Atlanta, GA 30303, USA

*To whom correspondence should be addressed, L.L., lli22@gsu.edu

Table of Contents

I. Expression and Purification of LecB…..…………………………………..….Page S2

II. Glycan Synthesis and Purification Procedures…………………………..…..Page S3

III. Glycan Microarray Fabrication and Assay………………………….....…….Page S8

IV. Structural Characterization of Synthesized Glycans..…….……………… Page S15

V. HPLC Analysis of Synthesized Glycans….………………………………….Page S35

VI. References……………………………………………………………………Page S42

VII. NMR Spectra of Synthesized Compounds………………………………..Page S43

I. Expression and Purification of LecB

The gene of fucose-binding lectin LecB (or named PA-IIL)[1] from *Pseudomonas aeruginosa* PAO1 (GeneID:77220128) was codon optimized and synthesized (Twist Bioscience) and cloned into plasmid pET22b between restriction sites NdeI and HindIII. The plasmid was sequenced and transformed into *E. coli* BL21(DE3) for protein expression and purification. Briefly, the transformed strain was cultured overnight and then transferred into 2 L of low-salt LB medium. The culture was incubated at 37 °C until the OD_{600nm} reached 0.6–0.8. The medium was then cooled to 16 °C, and IPTG was added to a final concentration of 0.1 mM to induce protein expression at 16 °C for an additional 20 hours. Cells were harvested, and the target LecB protein, bearing a C-terminal His tag, was purified using Ni-NTA chromatography as described above. The purified protein was buffer-exchanged into 50 mM Tris-HCl (pH 8.0), 100 mM NaCl. Purity was confirmed by SDS-PAGE (**Figure S1**, right panel), and protein concentration was determined using a NanoDrop spectrophotometer.

>Optimized LecB sequenced:

ATGGCGACACAAGGCGTCTTTACGCTTCCCGCAAATACACGCTTCGGCGTAACCGCCTTC
GCAAATTCCTCTGGTACACAGACTGTTAATGTTCTGGTAAATAACGAAACCGCTGCCACGT
TCAGCGGGCAATCTACAAACAATGCGGTGATAGGTACCCAAGTATTGAACTCGGGTAGTTC
AGGAAAGGTCCAGGTGCAAGTCAGCGTTAACGGCCGACCGTCTGACCTTGTATCAGCTCA
GGTTATCCTGACCAACGAGCTGAACTTTGCCCTGGTCGGTTCCGAGGACGGCACGGATAA
TGACTACAACGATGCAGTAGTCGTAATTAACTGGCCTCTTGGT

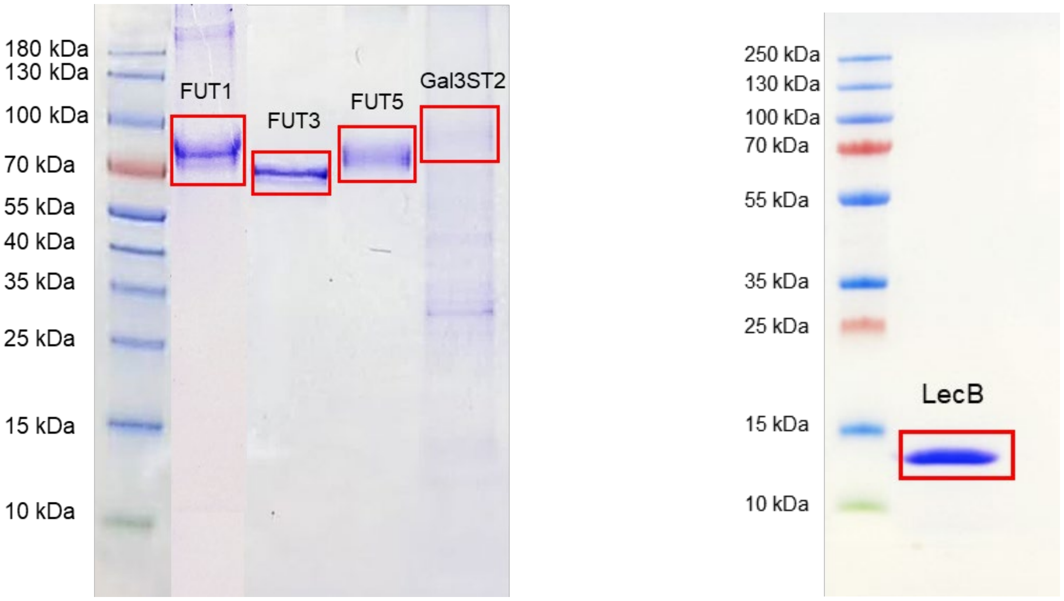

Figure S1. SDS-PAGE of purified GFP-fused FUT1 (mw of 87.7 kDa), FUT3 (mw of 83.1 kDa), FUT5 (mw of 85.9 kDa) and Gal3ST2 (mw of 91.5 kDa) (Left Panel), and LecB (mw of 13.4 kDa) (Right panel). Noting that Gal3ST2 has a low expression level but actively catalyzed the sulfation of type-1 LN.

II. Glycan Synthesis and Purification Procedures

General procedures for reaction monitoring and product purification:

Enzymatic reactions were monitored using analytical HPLC equipped with an XBridge peptide BEH C18 column (130Å, 5 μm, 4.6×250 mm) and a UV detector at 210 and 254 nm. The running solvents are solvent A (H_2O with 0.1% TFA) and solvent B (Acetonitrile with 0.1% TFA). The elution process involved a gradient increase in solvent B from 10% to 30% within 20 min, with a total flow rate of 1 mL/min for **Compounds 4−36**. The elution process involved a gradient increase in solvent B from 10% to 20% within 30 min, with a total flow rate of 1 mL/min for **DS-LNF V** (**2**) and **DS-LNF II** (**3**).

Synthetic reaction was quenched by adding the same volumes of ethanol, and store in 4 °C for 15 min. The mixture is then centrifuged to remove precipitates. The supernatant is concentrated using a rotary evaporator and subject to purification using a C18 column (Biotage, 120 Å, 50 µm, 3.0×20.0 cm or 1.8×11.4 cm). Products were eluted with solvent A (H_2O with 0.1% TFA) and solvent B (acetonitrile with 0.1% TFA) by a linear gradient increase of solvent B from 10% to 50% within 5 column volumes. Total flow rate is 12 mL/min. The product-containing fractions were concentrated and loaded to a P2 or P4 gel filtration column (2.5 x 120 cm, 50 mM NH_4HCO_3 as running buffer) based on the molecular weight (WM) of the products. P2 is used to purify products with MW between 300 to 1500 Da, and P4 is used to purify products with MW between 1500 to 3000 Da. Fractions were monitored by TLC with *p*-anisaldehyde staining.

General procedures for product characterization:

HR-MS analyses were performed on Waters Xevo G2_XS Mass Spectrometer (Waters Corporate, Milford, MA); ^1H,^{13}C NMR and 2-D NMR experiments were recorded on a Bruker AVANCE 600 (600 MHz) spectrometer at 25 °C. All ^1H Chemical shifts (in ppm) were assigned according to D_2O (δ= 4.79 ppm). The purity of synthesized compounds was analyzed by analytical HPLC equipped with an XBridge peptide BEH C18 column (130Å, 5 µm, 4.6×250 mm) and a UV detector at 210 and 254 nm. The running solvents are solvent A (H_2O with 0.1% TFA) and solvent B (Acetonitrile with 0.1% TFA), with a total flow rate of 1 mL/min.

HPLC profiles of synthetic capability analysis:

The 50 μL reaction system for enzyme test contains 100 mM Tris-HCl buffer (pH 8.0), 5 to 10 mM acceptor, 2.0 to 4.0 equivalents of sugar donor (GDP-Fuc), 20 mM $MgCl_2$, and purified FucTs, including Hm2FT (5 μg), Te2FT (5 μg), FUT1 (2 μg), WbsJ (5 μg), WbgN (5 μg), Hp3/4FT (5 μg), FUT3 (2 μg) or FUT5 (3 μg). The reactions were incubated at 37 °C for 4 h, monitored by HPLC.

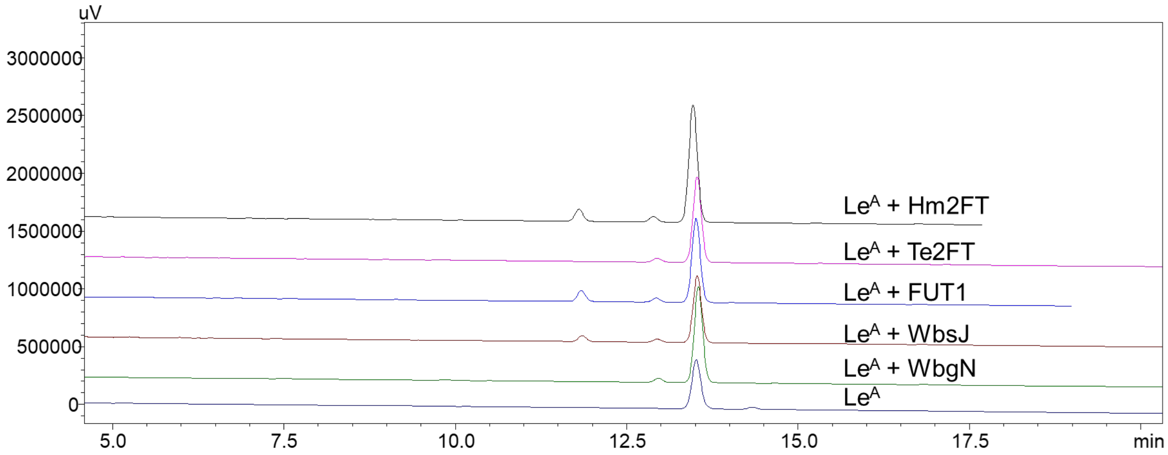

Figure S2. HPLC profiles of Hm2FT, Te2FT, FUT1, WbsJ and WbgN-catalyzed reactions using Le^A (**7**) as an acceptor. (Le^A: T_R = 13.5 min; fucosylation product: T_R = 11.8 min)

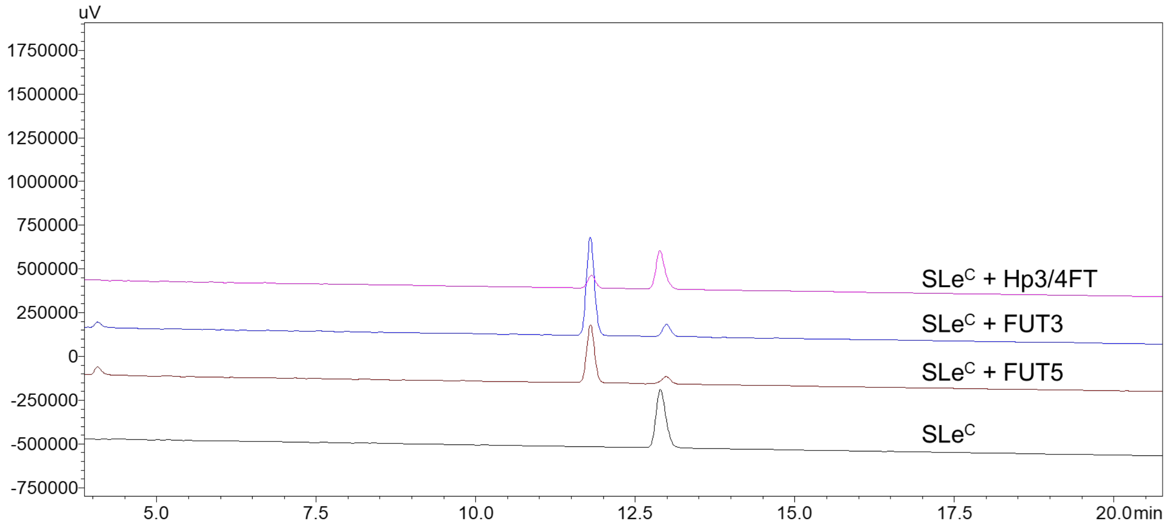

Figure S3. HPLC profiles of Hp3/4FT, FUT3 and FUT5-catalyzed reactions using SLe^C (**15**) as an acceptor. (SLe^C: T_R = 12.8 min; fucosylation product: T_R = 11.7 min).

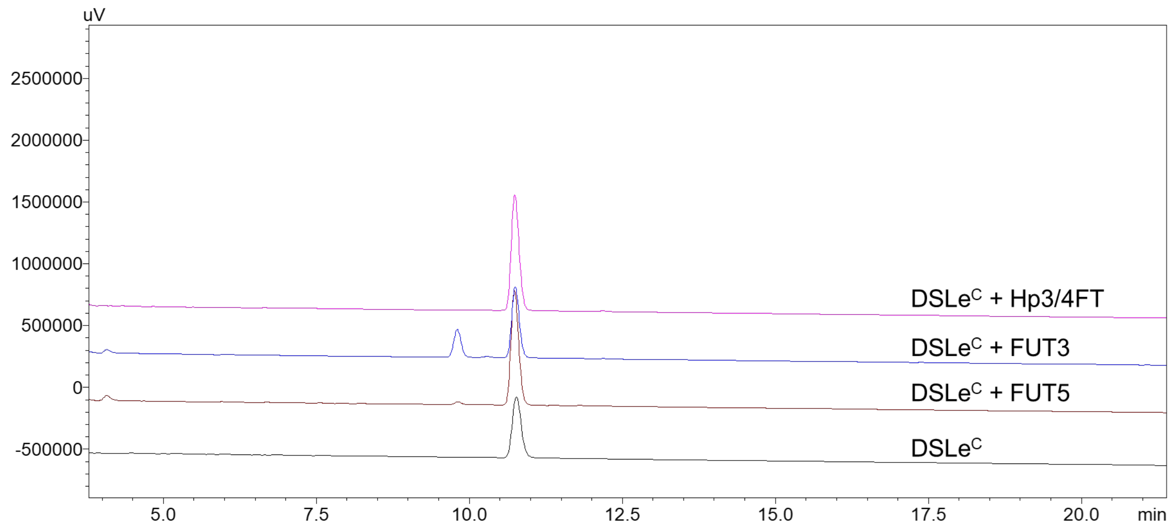

Figure S4. HPLC profiles of Hp3/4FT, FUT3 and FUT5-catalyzed reactions using DSLe^{C} (**17**). (DSLe^{C}: T_R = 10.7 min; fucosylation product: T_R = 9.8 min)

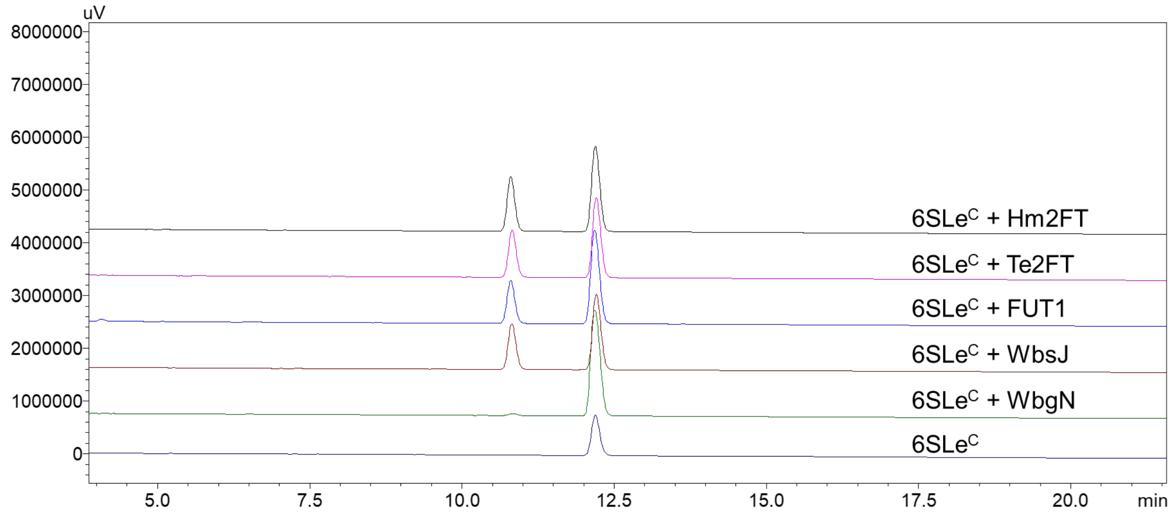

Figure S5. HPLC profiles of Hm2FT, Te2FT, FUT1, WbsJ and WbgN-catalyzed reactions using 6SLe^{C} (**23**) as an acceptor. (6SLe^{C}: T_R = 12.2 min; fucosylation product: T_R = 10.7 min)

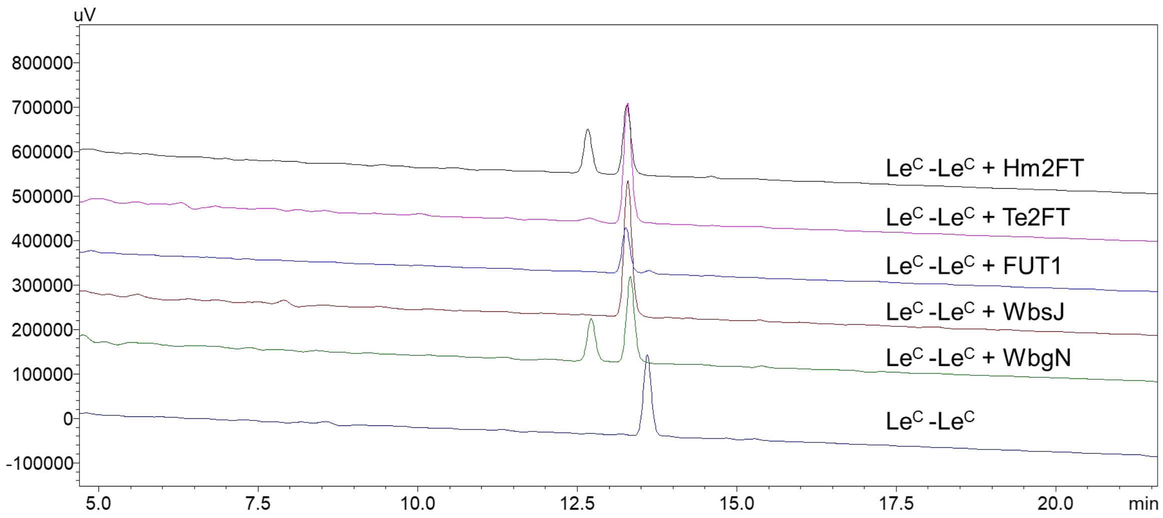

Figure S6. HPLC profiles of Hm2FT, Te2FT, FUT1, WbsJ and WbgN-catalyzed reactions using Le^{C}-Le^{C} (**29**) as an acceptor. (Le^{C}-Le^{C}: T_R = 13.6 min; mono-fucosylation product: T_R = 13.2 min; di-fucosylation product: T_R = 12.6 min)

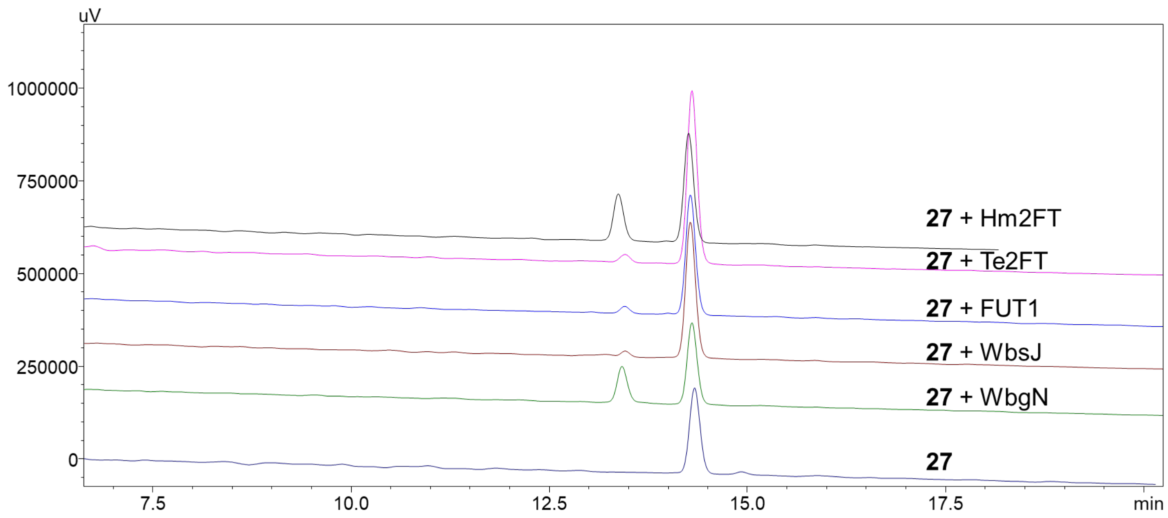

Figure S7. HPLC profiles of Hm2FT, Te2FT, FUT1, WbsJ and WbgN-catalyzed reactions using **27** as an acceptor. (**27**: T_R = 14.3 min; fucosylation product: T_R = 13.3 min)

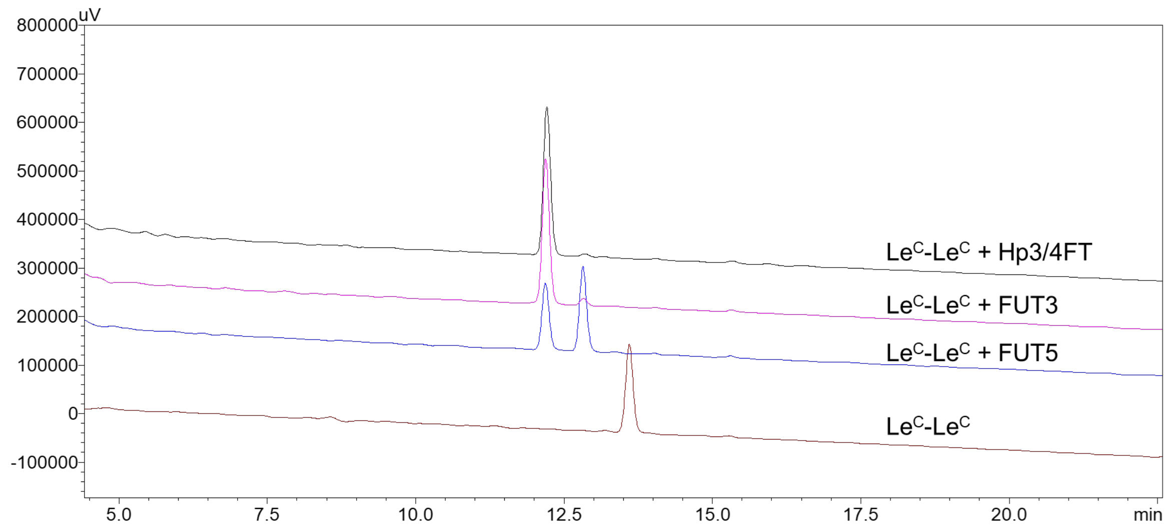

Figure S8. HPLC profiles of Hp3/4FT, FUT3 and FUT5-catalyzed reactions using Le^C-Le^C (**29**) as an acceptor. (Le^C-Le^C: T_R = 13.6 min; mono-fucosylation product: T_R = 12.8 min; di-fucosylation product: T_R = 12.1 min)

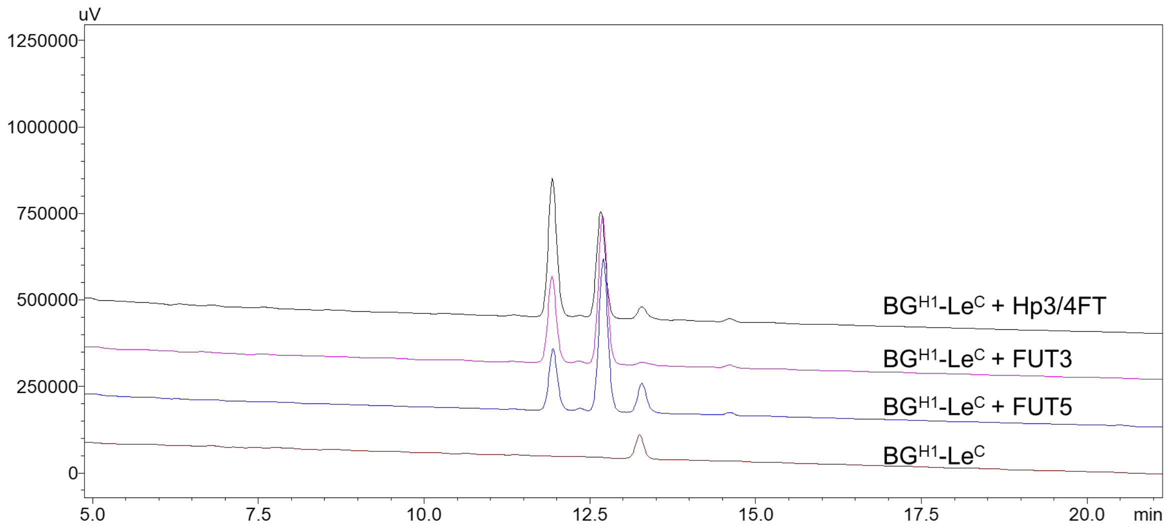

Figure S9. HPLC profiles of Hp3/4FT, FUT3 and FUT5-catalyzed reactions using BG^{H1}-Le^C (**30**) as an acceptor. (BG^{H1}-Le^C: T_R = 13.2 min; mono-fucosylation product: T_R = 12.7 min; di-fucosylation product: T_R = 11.9 min)

III. Glycan Microarray Fabrication and Assay

Table S1 Glycan microarray information based on MIRAGE.

Classification	Guidelines
1. Sample: Glycan Binding Sample	
Description of Sample	Glycan binding proteins (Lectins and monoclonal antibodies)
Sample modifications	Not applicable.
Assay protocol	Microarray analyses were performed as described in Section 4.
2. Glycan Library	
Glycan description for defined glycans	All glycans were synthesized as described in the body text.
Glycan description for undefined glycans	Not applicable.
Glycan modifications	Glycans were attached with *O*-aminopropyl by chemical synthesis prior to enzymatic synthesis. No modifications were applied after synthesis.
3. Printing Surface; e.g., Microarray Slide	
Description of surface	Z-biotech Multivalent Microarray Slides
Manufacturer	Z-biotech
Custom preparation of surface	Not applicable.
Covalent Immobilization	Amine-NHS cross-linking.
4. Arrayer (Printer)	
Description of Arrayer	sciFLEXARRAYER S3 spotter (Scienion) with a PDC 70 Piezo Dispense Capillary, and 16 subarrays were printed on each slide.
Dispensing mechanism	Non-contact liquid delivery.
Glycan deposition	Each glycan probe was printed at 1 deposit in 6 replicates.
Printing conditions	Samples were prepared at a concentration of 100 μM in the printing buffer (300 mM phosphate, pH 8.5), printing was performed at room temperature and relative humidity of 65%.
5. Glycan Microarray with “Map”	
Array layout	Each array slide contained 16 identical subarrays (pads). Each subarray contained 32 type-1 glycan determinants, 4 type-2 reference epitopes and 2 controls.
Glycan identification and QC	Quality control included analyses with plant lectins
6. Detector and Data Processing	
Scanning hardware	GenePix 4000B Microarray Scanner (Molecular Devices, LLC)
Scanner settings	Laser channel: wavelength 635 nm and 532 nm<br>PMT gain: 800 or 600<br>Scan power: 100%
Image analysis software	GenePix Pro (Molecular Devices, LLC)
Data processing	The gpr files were processed with in-house excel macro to obtain basic descriptive statistics. No particular normalization method or statistical analysis was used.
7. Glycan Microarray Data Presentation	
Data presentation	The microarray binding results are in main context and supporting information. Binding results are presented as relative fluorescence intensity units (RFU) of binding in mean and S.D.
8. Interpretation and Conclusion from Microarray Data	
Data interpretation	No software or algorithms were used to interpret processed data.
Conclusions	Described in Results parts.

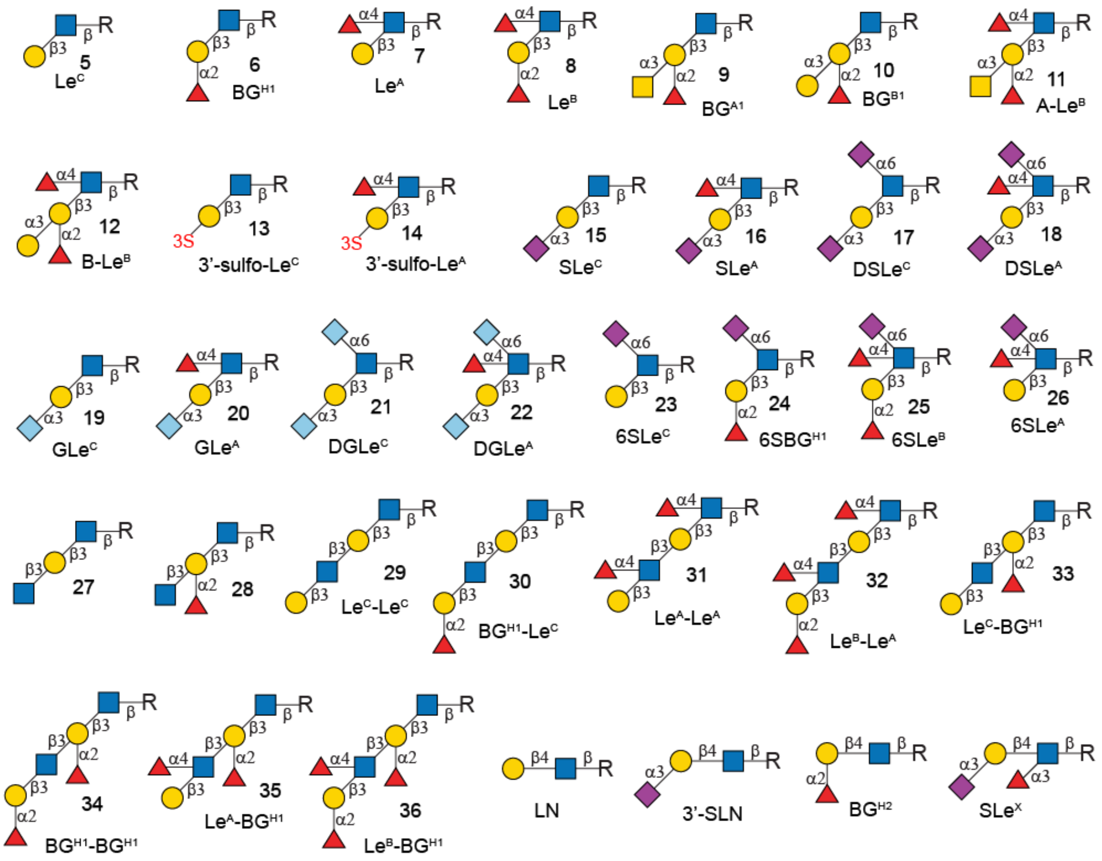

Figure S10. Structures of all glycan structures printed on the microarray.

Table S2. Glycan-binding proteins and antibodies used in this study.

REAGENT OR RESOURCE	SOURCE	CAT#	Concentration used in Assay
**Antibodies and Streptavidin**			
Goat anti-mouse IgG(H+L) 2nd antibody AF647-conjugated	Invitrogen	A21235	5 μg/mL
Goat anti-human IgG(H+L) 2nd antibody AF647-conjugated	Invitrogen	A21445	5 μg/mL
Goat anti-mouse IgM 2nd antibody AF555-conjugated	Invitrogen	A21426	5 μg/mL
Anti-His Tag Alexa Fluor® 647-conjugated Mouse IgG	R&D Systems	IC05051R	10 μg/mL
Streptavidin, Cyanine 5	Vector Laboratories	SA-1500-1	0.5 μg/mL
HE-193 (anti-blood group A monoclonal antibody)	Invitrogen	MA1-19693	10 μg/mL
HEB-29 (anti-blood group B monoclonal antibody)	Invitrogen	MA1-19691	10 μg/mL
7LE (anti-Lewis A monoclonal antibody)	Invitrogen	MA1-19344	10 μg/mL
2-25LE (anti-Lewis B monoclonal antibody)	Invitrogen	MA1-19346	10 μg/mL
FH7 (anti-disialyl Lewis A monoclonal antibody)	Fisher	NB019471	10 μg/mL
MVT-5873 (anti-sialyl Lewis A monoclonal antibody)	Fisher	NB466528	10 μg/mL
17-206 (anti-type-1 blood group H monoclonal antibody)	Invitrogen	14-9810-82	10 μg/mL
1116-NS-19-9 (anti-sialyl Lewis A monoclonal antibody)	InVivoMAb™	BE0355	10 μg/mL
**Lectins**			
Aleuria aurantia lectin (AAL), Biotinylated	Vector Laboratories	B-1395-1	10 μg/mL
Ulex europaeus agglutinin I (UEA-I), Biotinylated	Vector Laboratories	L-1065-2	10 μg/mL
Solanum Tuberosum (Potato) Lectin (STL, PL), Biotinylated	Vector Laboratories	B-1165-2	10 μg/mL
Pseudomonas aeruginosa PAO1 lectin LecB (PA-IIL) with N-terminal His-tag	Li Lab expressed	This work	10 μg/mL

GBPs			
Recombinant Human Siglec-7/CD328 hIgG1-Fc Chimera Protein	R&D Systems	1138-SL	50 μg/mL
Human SIGLEC9 Protein (ECD, His & Fc Tag)	Sino Biological	30109-H03H	25 μg/mL
Recombinant Human P-Selectin/CD62P hIgG1 Fc Chimera Protein	R&D Systems	137-PS	20 μg/mL
Recombinant Human E-Selectin/CD62E hIgG1 Fc Chimera Protein	R&D Systems	724-ES	20 μg/mL

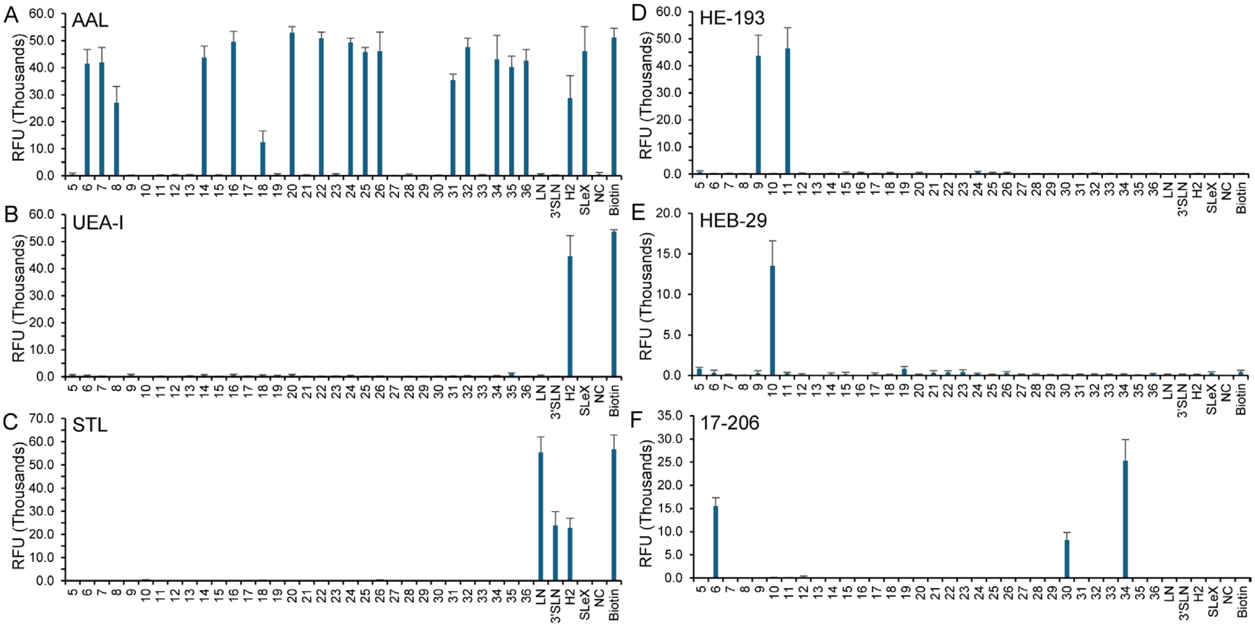

Figure S11. Microarray evaluation using lectins and anti-ABO blood group antigen antibodies. A) AAL (10 μg/mL); B) UEA-I (10 μg/mL); C) STL (10 μg/mL); D) Blood-group A antibody HE-193 (10 μg/mL); E) Blood-group B antibody HEB-29 (10 μg/mL); F) Blood-group H (type-1) antibody 17-206 (10 μg/mL).

AAL bound all fucosylated glycans except blood group A and B antigen as reported,[2] it also did not bind to internal α1-2fucosylated glycan **28** and **33**. Compound **31** with a terminal α1-2fucosylation was also not recognized, which is surprising. UEA-I only recognizes type-2 H-antigen, and STL recognize type-2 LacNAc, type-1 H-antigen and 3'SLN, as expected.[2] Anti-A antibody HE-193 recognize both type-1 A-antigen and A-Le^B whereas anti-B antibody HEB-29 solely recognize type-1 B-antigen, suggesting that HEB-29 is blocked by the internal α1-4fucosyaltion. Anti-type-1 H-antibody bound glycans with terminal type-1 H-antigen (**6**, **30** and **34**) as expected. These results confirmed the successful fabrication of the microarray and the glycan structures.

Table S3. Glycan microarray data

	**UEA-I** (10 μg/mL)		**AAL** (10 μg/mL)		**HE-193** (10 μg/mL)		**HEB-29** (10 μg/mL)	
**Glycan**	Ave_Mean	STDEV	Ave_Mean	STDEV	Ave_Mean	STDEV	Ave_Mean	STDEV
5	697.17	106.04	542.17	468.68	667.33	493.33	831.33	186.29
6	434.33	219.52	41509.67	5196.83	131.33	85.22	313.17	352.75
7	37.50	154.92	41906.00	5553.39	72.67	187.43	46.83	72.80
8	49.50	50.05	26949.83	6105.45	43.67	144.75	0.00	64.26
9	574.83	365.45	185.17	114.05	43633.83	7669.23	279.33	314.01
10	32.33	29.43	0.00	0.00	60.67	142.51	13500.33	3117.54
11	43.33	163.48	158.33	189.36	46474.00	7667.44	217.00	168.96
12	9.00	44.39	209.17	254.05	167.00	186.08	106.00	124.26
13	208.00	158.77	118.67	394.21	109.33	50.47	0.00	7.87
14	677.50	121.55	43717.83	4256.97	150.83	111.39	136.00	189.60
15	30.83	99.18	213.50	236.83	353.17	355.04	141.67	258.34
16	586.50	357.53	49600.00	3847.30	398.00	218.95	7.33	39.95
17	142.00	126.10	0.00	5.47	139.00	125.38	151.33	165.71
18	444.50	302.76	12414.17	4160.59	380.33	196.64	75.67	76.30
19	462.83	106.60	520.83	244.32	0.00	78.33	796.50	319.32
20	747.67	139.66	53011.50	2162.80	369.17	231.28	76.83	99.51
21	74.67	148.46	212.67	202.13	16.83	44.98	321.00	267.23
22	105.83	101.38	50811.17	2292.21	128.17	75.45	365.33	220.29
23	207.50	63.94	494.33	263.54	38.00	80.43	387.33	317.32
24	450.17	162.62	49246.17	1673.28	898.67	102.04	188.00	119.53
25	135.17	99.91	45818.83	1629.49	378.83	232.65	63.83	83.30
26	160.33	113.64	46102.50	7037.33	417.50	208.46	308.33	182.73
27	8.33	91.94	70.67	95.19	16.17	93.91	114.83	73.99
28	42.00	137.54	331.00	334.04	61.83	109.12	95.17	112.38
29	49.67	68.93	32.33	110.93	63.00	92.24	71.67	37.82
30	161.00	72.47	30.83	273.13	16.00	70.27	35.83	62.94
31	203.00	109.00	35421.50	2154.52	33.50	175.28	29.33	160.65
32	279.17	140.28	47593.33	3294.38	205.00	115.95	114.83	78.70
33	5.00	117.63	265.67	220.06	76.50	117.93	52.83	124.91
34	276.00	198.59	43118.33	8765.82	124.00	77.60	140.67	66.32
35	1067.50	378.82	40284.33	3933.43	33.67	99.71	38.17	54.76
36	8.17	111.23	42593.83	4128.58	9.33	203.88	176.50	109.75
LN	433.00	198.20	583.67	203.20	0.00	8.44	122.67	53.63
3'SLN	17.17	125.83	150.33	180.87	0.00	24.49	46.00	126.54
H2	44589.00	7589.02	28719.33	8314.20	43.50	140.95	47.83	117.67
SLe^X	0.00	37.86	46049.00	9123.78	42.83	91.70	283.50	179.91
NC	7.67	63.73	567.67	550.20	110.17	78.14	0.00	38.35
Biotin	53586.67	858.51	51183.17	3384.00	74.83	96.26	402.67	260.47

Table S3. Glycan microarray data (continued)

	**7LE** (10 μg/mL)		**2-25LE** (10 μg/mL)		**FH7** (10 μg/mL)		**STL** (10 μg/mL)	
**Glycan**	Ave_Mean	STDEV	Ave_Mean	STDEV	Ave_Mean	STDEV	Ave_Mean	STDEV
5	1.83	5.46	4.33	4.46	50.00	117.38	83.67	24.24
6	2.17	1.57	20.50	22.43	18.17	37.84	0.00	9.12
7	12620.50	3186.82	8225.00	565.49	46.17	33.85	29.33	14.33
8	0.33	3.20	12484.50	2952.84	0.00	7.93	20.50	29.84
9	218.33	113.97	1.50	0.96	0.00	17.18	0.50	18.58
10	0.00	3.35	0.00	1.71	5.83	30.87	163.17	324.30
11	0.83	1.07	0.00	2.65	41.67	53.18	7.50	7.11
12	18.00	33.17	3.17	6.52	18.17	29.02	25.33	48.58
13	0.33	1.70	1.17	5.43	8.67	5.65	29.33	6.60
14	0.83	6.99	2.50	10.18	467.33	321.12	28.50	44.38
15	7.00	17.08	2.83	3.80	4.00	16.37	4.83	7.51
16	2.67	6.97	1.00	3.11	1195.33	450.16	5.50	17.46
17	0.00	4.06	4.50	5.99	8.17	10.25	0.33	30.58
18	1.17	4.14	2.83	3.80	30730.67	2519.95	41.17	137.32
19	2.33	4.15	4.17	4.06	17.83	44.83	19.00	34.43
20	0.33	5.62	0.00	5.19	707.83	339.63	0.00	26.26
21	0.00	6.91	1.50	3.35	34.00	37.51	10.33	24.18
22	7.50	6.24	4.00	8.02	35224.50	2501.17	7.00	8.85
23	2.50	2.50	10.00	21.06	32.17	55.09	25.00	35.58
24	5.00	9.38	4.17	4.22	1050.00	248.72	5.83	22.12
25	3.83	5.81	9461.67	2234.95	31631.00	967.01	10.67	9.01
26	314.00	200.22	12807.33	1973.21	44093.83	4814.50	193.83	139.93
27	50.33	107.75	1.33	2.05	3.83	10.43	9.50	21.30
28	3.83	10.82	0.50	3.20	26.67	23.90	17.00	11.55
29	11.67	19.52	0.00	2.57	8.83	42.68	11.00	13.47
30	8.00	10.18	83.50	50.80	38.67	34.68	23.50	76.27
31	23629.00	4417.70	2534.00	417.84	416.83	589.60	28.67	30.71
32	4.33	3.68	8894.00	327.24	0.00	34.35	9.33	5.56
33	700.83	39.11	1.17	17.00	49.50	16.95	17.83	24.76
34	43.33	82.90	4.33	7.02	0.00	11.80	11.83	18.98
35	20519.00	3796.18	4324.00	849.74	50.67	99.11	10.50	18.95
36	14.00	10.46	12018.83	2372.11	0.00	13.63	24.83	31.96
LN	46.83	106.14	3.67	4.35	4.67	14.66	55394.50	6681.22
3'SLN	0.67	4.46	0.00	2.27	0.00	12.84	23936.33	5837.85
H2	43.17	78.80	44.33	91.79	0.00	19.32	22833.00	4136.35
SLe^X	5.50	7.27	3.50	4.89	0.00	13.67	0.00	11.50
NC	2.50	5.12	16.50	32.14	76.17	80.73	0.00	37.32
Biotin	171.67	200.13	0.83	3.02	161.67	61.16	56836.67	6094.65

Table S3. Glycan microarray data (continued)

	**P-selectin** (20 μg/mL)		**E-selectin** (20 μg/mL)		**Siglec-7** (50 μg/mL)		**Siglec-9** (25 μg/mL)	
**Glycan**	Ave_Mean	STDEV	Ave_Mean	STDEV	0.00	0.00	0.00	0.00
5	0.00	0.00	24.67	7.52	214.17	95.91	0.00	0.00
6	93.17	32.50	0.00	0.00	0.00	0.00	9.00	12.00
7	655.17	272.04	4743.83	163.10	329.17	77.75	247.00	18.00
8	912.83	165.93	867.83	199.31	5.17	33.92	0.00	5.00
9	14.67	8.90	7.33	5.62	0.00	0.00	0.00	0.00
10	143.83	43.46	0.00	0.00	0.00	0.00	292.50	35.50
11	680.17	199.06	24.17	27.76	553.17	114.96	432.00	0.00
12	966.67	227.21	0.00	0.00	75.50	63.53	549.00	90.00
13	1.33	4.89	16.17	11.07	498.33	33.51	80.50	2.50
14	380.00	54.71	8039.33	1931.55	43.00	46.59	348.00	22.00
15	12.67	8.77	2.33	5.91	0.00	0.00	2820.00	416.00
16	3037.33	456.64	20902.00	1501.78	7655.00	1023.02	14943.50	1429.50
17	0.00	9.70	0.00	0.00	2780.83	965.09	8793.00	121.00
18	730.33	190.09	35.50	28.24	23.67	26.61	1227.00	163.00
19	1.33	16.03	7.50	12.33	47.17	30.62	2238.00	359.00
20	5773.83	887.18	20278.00	608.78	5664.83	1138.03	9239.50	798.50
21	3.33	7.56	2.67	3.73	1289.50	472.08	3114.00	380.00
22	0.00	0.00	2179.33	878.63	1988.83	889.45	21.00	13.00
23	8.50	9.03	3.67	3.68	4454.83	1060.92	0.00	0.00
24	2.33	2.43	4.83	3.18	0.00	0.00	0.00	0.00
25	0.00	0.00	0.00	0.00	475.67	134.24	19.00	1.00
26	2.33	5.56	1.83	15.88	308.67	87.85	17.50	1.50
27	109.00	43.07	0.17	9.48	391.83	121.80	232.00	63.00
28	252.50	64.75	5.00	4.93	0.00	0.00	281.50	17.50
29	81.17	17.63	8.83	5.43	493.83	46.08	1024.00	494.00
30	159.50	57.64	9.50	3.25	107.83	24.44	107.50	10.50
31	516.67	48.21	22.67	9.76	0.00	0.00	11.50	9.50
32	211.83	59.46	4858.67	1440.52	205.83	47.60	351.00	24.00
33	251.67	29.03	2.67	9.10	20.17	52.32	0.00	0.00
34	9.00	5.72	16.83	10.98	0.00	0.00	206.00	66.00
35	219.50	83.95	1197.67	246.84	22.00	15.24	79.50	12.50
36	376.33	45.29	2407.67	750.09	88.33	274.00	0.00	0.00
LN	0.00	0.00	1.33	5.15	274.33	57.69	345.50	55.50
3'SLN	38.50	8.32	4.33	13.88	45.83	15.12	543.00	53.00
H2	105.17	29.26	4.67	9.14	297.50	41.84	7305.00	687.00
SLe^{X}	2008.50	231.57	5351.67	1139.32	237.83	76.66	130.50	11.50
NC	38.67	19.33	0.33	4.42	20.17	40.40	88.50	6.50
Biotin	26.83	16.23	0.83	3.72	0.00	0.00	0.00	0.00

Table S3. Glycan microarray data (continued)

	**1116-NS-19-9** (10 μg/mL)		**MVT-5873** (10 μg/mL)		**17-206** (10 μg/mL)		**LecB** (10 μg/mL)	
**Glycan**	Ave_Mean	STDEV	Ave_Mean	STDEV	Ave_Mean	STDEV	Ave_Mean	STDEV
5	104.00	40.52	5154.50	1329.05	9.50	4.27	139.17	28.16
6	6.33	25.43	25.17	75.57	15487.00	1857.61	1643.33	347.22
7	29.67	21.15	10734.83	2486.23	1.17	1.77	6956.33	459.05
8	4.50	22.69	0.00	72.67	9.33	37.46	44.67	4.68
9	6.33	5.44	0.00	12.00	0.00	4.31	1.17	1.07
10	8.67	13.06	0.00	44.10	60.67	71.49	0.50	1.89
11	0.00	22.98	0.00	39.36	26.83	59.13	5.50	2.36
12	70.67	42.70	34.67	18.95	146.83	296.52	1.50	1.98
13	16.17	8.03	304.00	113.36	3.50	2.75	3.67	1.25
14	4.00	14.11	14177.33	1826.95	0.00	3.20	5912.33	1745.84
15	29.00	20.07	0.00	15.01	0.00	2.99	1.50	1.26
16	43802.83	7685.73	31842.67	5029.90	1.00	1.15	11092.83	607.01
17	7.50	17.00	21.67	14.15	0.17	3.24	1.33	2.21
18	8.00	33.03	10652.83	1844.17	0.67	2.05	1.67	2.56
19	22.17	18.32	0.00	27.33	0.33	4.35	1.17	1.77
20	33313.33	2738.62	40595.17	5909.31	13.67	36.94	7827.67	1963.85
21	57.50	52.79	0.00	18.43	4.33	4.61	0.17	1.21
22	74.67	57.43	14517.00	934.77	0.33	3.94	5.83	4.49
23	25.17	21.02	293.83	109.05	6.50	6.68	2.17	2.41
24	26.50	22.08	0.00	36.94	0.17	11.10	4881.33	543.90
25	35.33	65.91	0.00	136.49	0.00	3.34	2.50	1.89
26	61.00	25.11	5057.50	379.63	13.00	26.20	127.17	29.25
27	11.17	37.37	0.00	86.50	0.67	4.46	1.33	1.80
28	18.33	20.21	2113.17	508.36	12.50	13.78	1020.33	94.80
29	60.33	139.91	3.50	90.20	0.00	3.59	2.67	2.92
30	12.00	8.79	0.00	22.11	8182.00	1653.93	2.00	3.37
31	61.83	71.98	3593.00	767.20	0.00	19.64	5156.50	1952.63
32	80.67	148.53	0.00	46.24	0.33	1.11	7667.00	640.42
33	0.00	6.24	1550.00	328.54	14.67	21.72	449.17	149.53
34	30.33	46.99	348.00	301.46	25268.00	4630.67	7908.33	633.02
35	23.17	26.35	2676.67	625.84	0.00	2.52	8767.67	461.59
36	16.33	11.70	0.00	42.35	0.00	3.94	4448.00	1222.50
LN	3.67	10.51	0.00	63.54	0.17	2.91	0.00	0.00
3'SLN	25.33	51.07	0.00	12.93	2.50	15.59	0.83	2.41
H2	28.33	10.83	35.17	6.39	0.00	1.25	3.67	3.40
SLe^{X}	13.17	5.96	0.00	34.36	0.00	1.86	122.67	17.93
NC	20.67	35.33	81.67	83.29	0.00	1.95	0.83	2.61
Biotin	12.17	25.43	59.67	12.15	0.00	5.35	0.33	2.69

IV. Structural Characterization of Synthesized Glycans

NMR Signals were assigned based on ^{1}H-NMR, ^{13}C-NMR and 2D-NMR including HSQC (Heteronuclear Single Quantum Coherence), HMBC (Heteronuclear Multiple Bond Correlation), ^{1}H-^{1}H COSY (Correlated Spectroscopy) and HSQC-TOCSY (Heteronuclear Single Quantum Coherence-Total Correlation Spectroscopy). The Compound DS-LNF II, BG^{H1}-Le^{C} and Le^{B}-BG^{H1} were taken for example to elucidate the NMR signal assignment method.

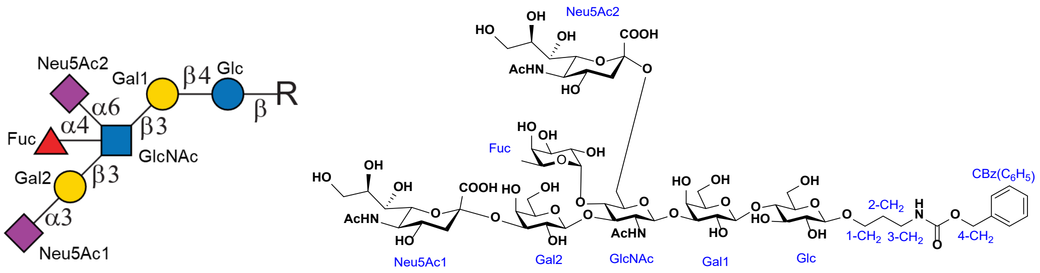

The protons of 2-CH_2 from the linker of DS-LNF II could be assigned at (δ 1.80, 28.94) and 1-CH_2, 3-CH_2 could be found out in HSQC-TOCSY spectra were assigned at (δ 3.94/3.69, 67.73) and (δ 3.24, 37.38) (**Figure S12**). The H-1 of the reducing end Glc could be assigned at (δ 4.45, 102.96) by a two-bond coupling signal in merged HSQC and HMBC spectra (**Figure S13**). According to the correlation between acetylcarbonyl carbon and GlcNAc H-2, Neu5Ac1 H-5 and Neu5Ac2 H-5 could be assigned at (δ 3.94, 55.78), (δ 3.85, 51.66) and (δ 3.83, 51.83), respectively (**Figure S14**).

In ^{1}H-^{1}H COSY (**Figure S15**), the GlcNAc H-1 could be assigned at (δ 4.69, 102.63), which has the correlation with GlcNAc H-2 assigned at δ 3.94; GlcNAc H-3 has the correlation with GlcNAc H-2 at δ 4.07; the H-4 of GlcNAc has the correlation with H-3 at δ 3.79. Meanwhile, Glc H-2 has the correlation with Glc H-1 at δ 3.30, and H-3 of Glc could be assigned at δ 3.62. The H-1 of Fuc assigned at δ 5.18 has correlation with Fuc H-2 at δ 3.83. Merged HMBC and HSQC-TOCSY spectra (**Figure S16**) shows the proton signals of Glc, Gal1, GlcNAc, Gal2 and Fuc. It is noteworthy that Fuc C-1 has correlation with GlcNAc H-4 assigned at (δ 3.79, 72.05) in merged HSQC and HMBC spectra (**Figure S16**), which suggests that Fucose is linked to GlcNAc rather than Glucose H-3 assigned at (δ 3.62, 68.22).

The H-1 of Gal1 has the correlation to the C-4 of the Glc at δ 78.51; the H-1 of GlcNAc has the correlation to the C-3 of the Gal1 at δ 82.37; the H-1 of Gal2 has the correlation to the C-3 of the GlcNAc at δ 75.54; the H-3 of Gal2 has the correlation to the C-2 of the Neu5Ac1 at δ 99.37 and the H-6 of GlcNAc has the correlation to the C-2 of the Neu5Ac2 at δ 100.48 in Merged HSQC and HMBC spectra (**Figure S17**). Gal1 H-1 has correlations with Gal1 H-2 in ^{1}H-^{1}H COSY could be assigned at δ 4.42 and δ 3.59. Gal1 H-3 and H-4 could be assigned at δ 3.69 and δ 4.17. Gal2 H-1 has correlations with Gal2 H-2 in ^{1}H-^{1}H COSY could be assigned at δ 4.54 and δ 3.54. Gal2 H-3 and H-4 could be assigned at δ 4.08 and δ 3.91 in ^{1}H-^{1}H COSY spectra (**Figure S17**). The remaining protons that show intra-spin system signals were assigned by COSY, HSQC, HSQC-TOCSY and Merged HSQC-HMBC analysis.

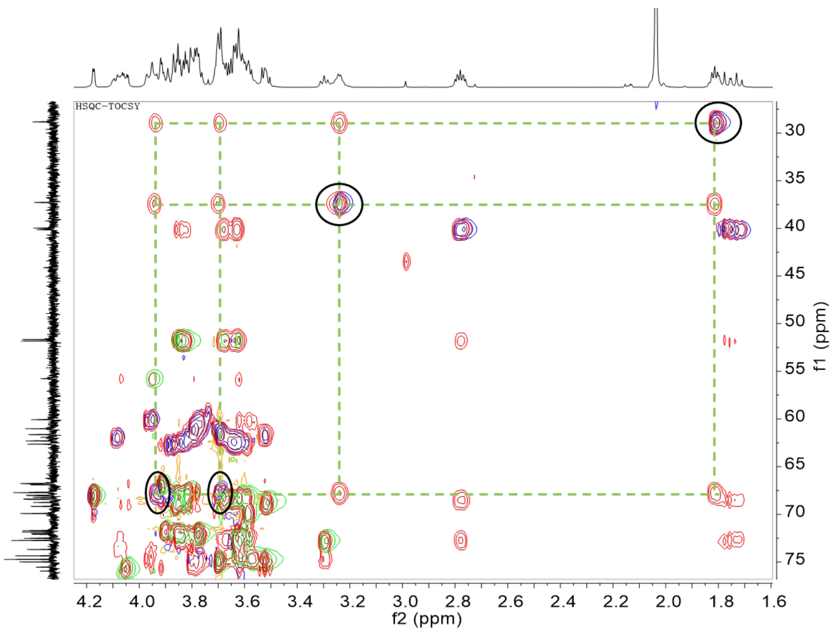

Figure S12. Merged HSQC (in green and blue) and HSQC-TOCSY (in red) of Compound DS-LNF II.

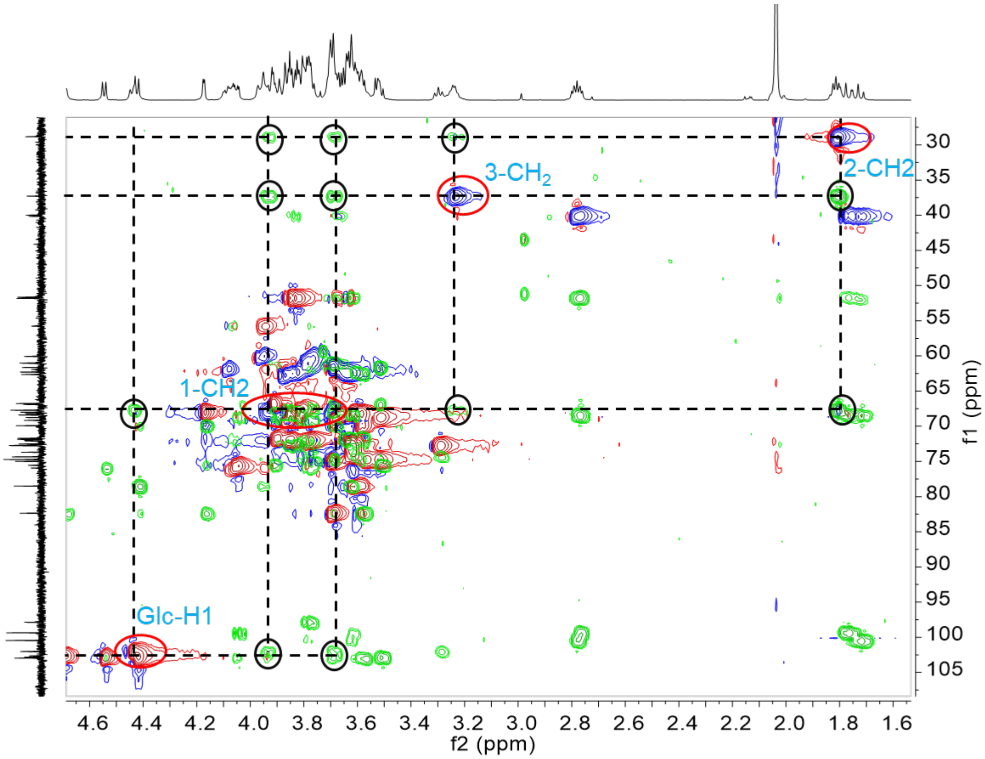

Figure S13. Merged HSQC (in red and blue) and HMBC (in green) of Compound DS-LNF II showing the correlation of CH_2 groups (1, 2 and 3) on the linker with the anomeric Glc (H-1).

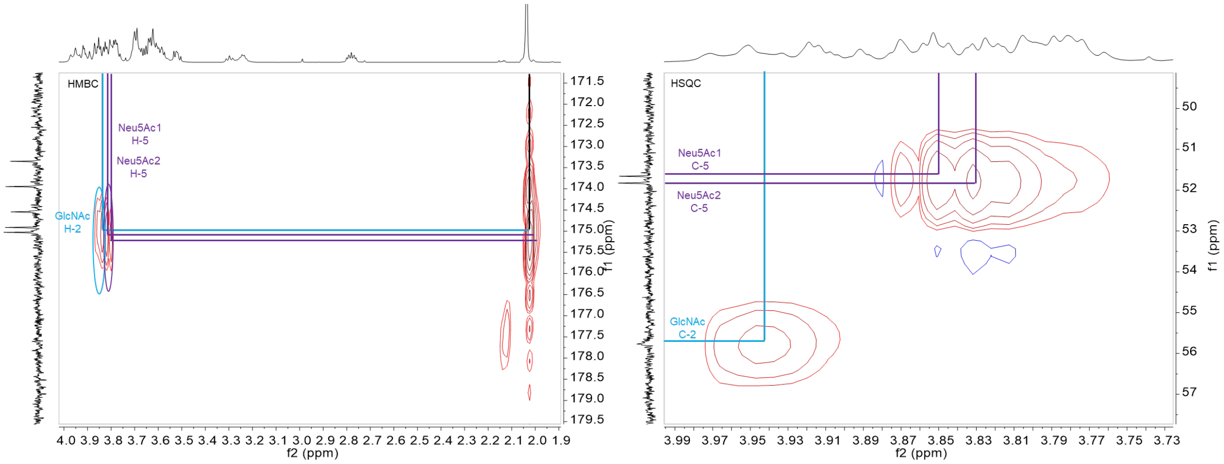

Figure S14. HMBC and HSQC spectra of Compound DS-LNF II.

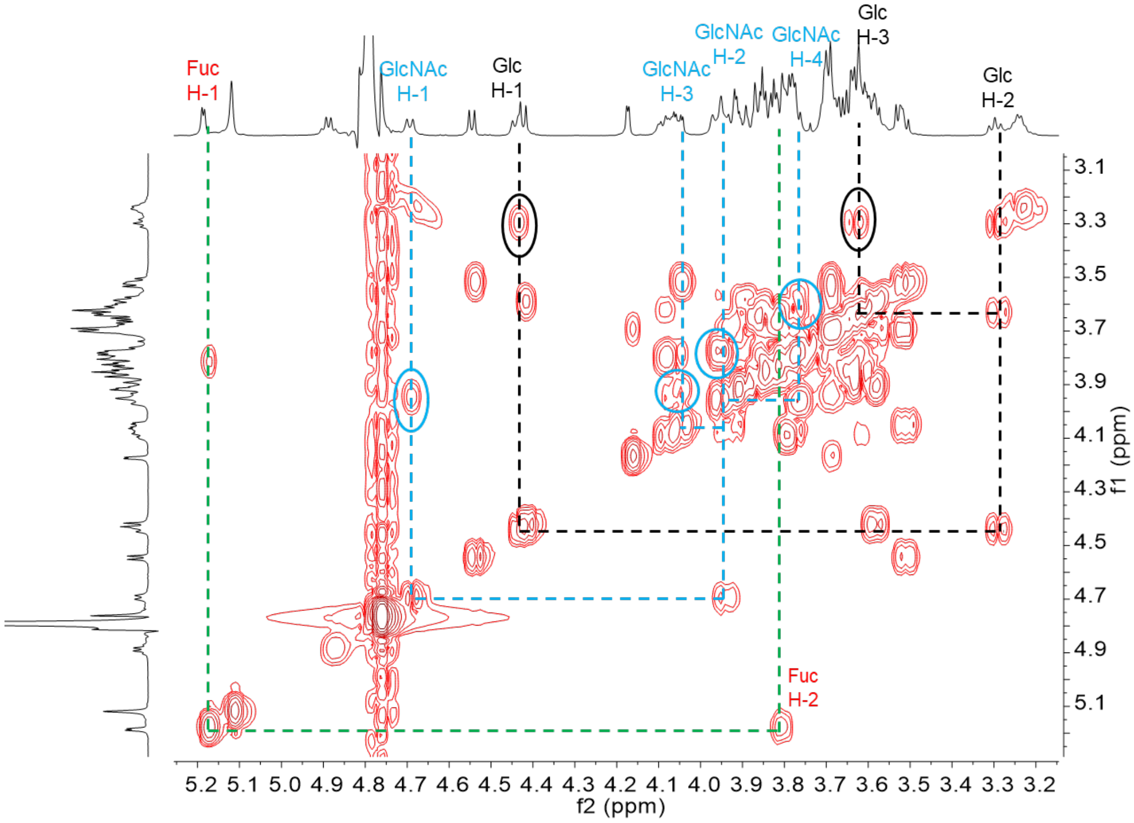

Figure S15. ^1H-^1H COSY spectra of Compound DS-LNF II

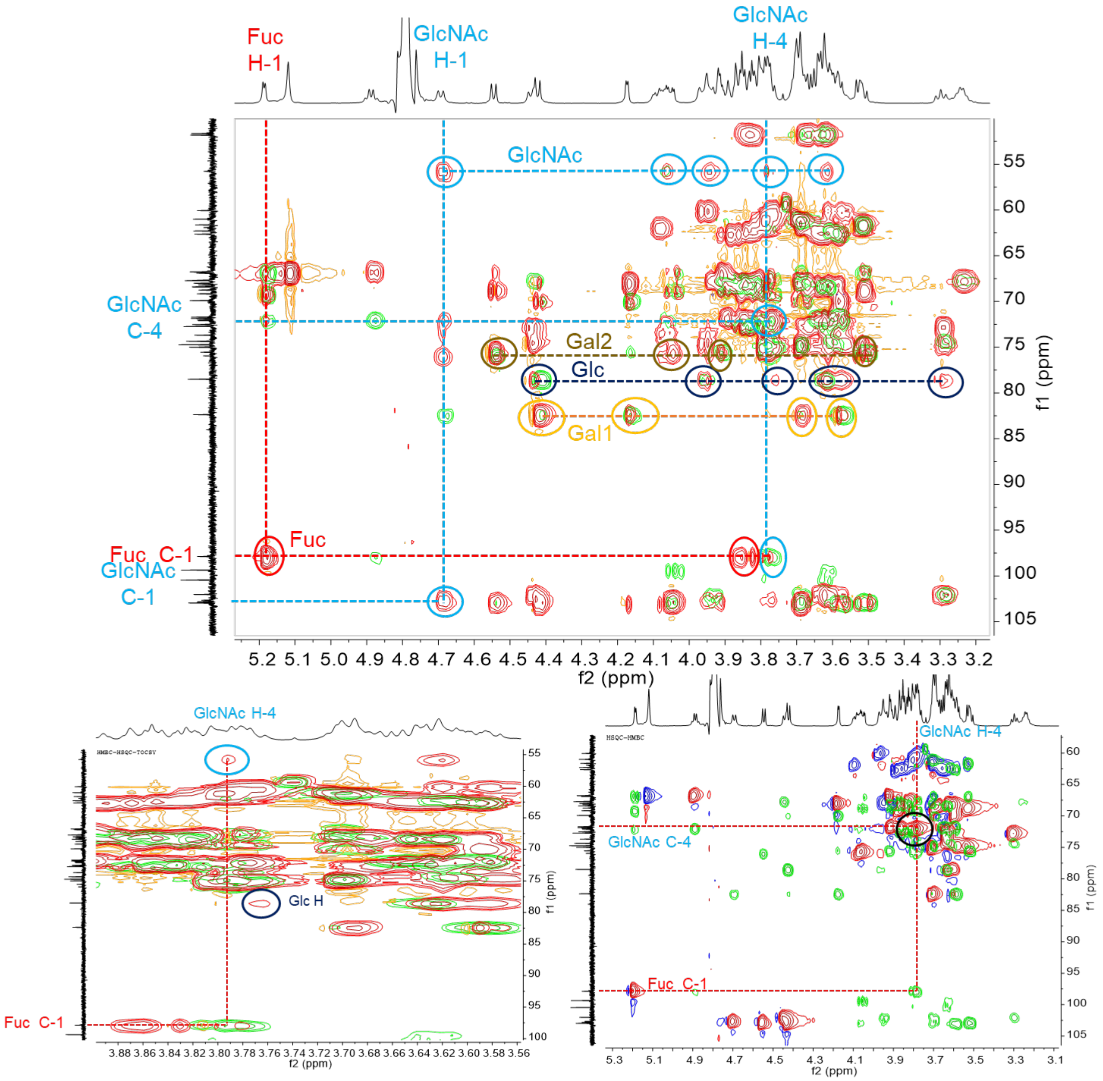

Figure S16. Merged HSQC-TOCSY (in red) and HMBC (in green), along with the combined HSQC (in red and blue) and HMBC (in green) spectra of Compound DS-LNF II.

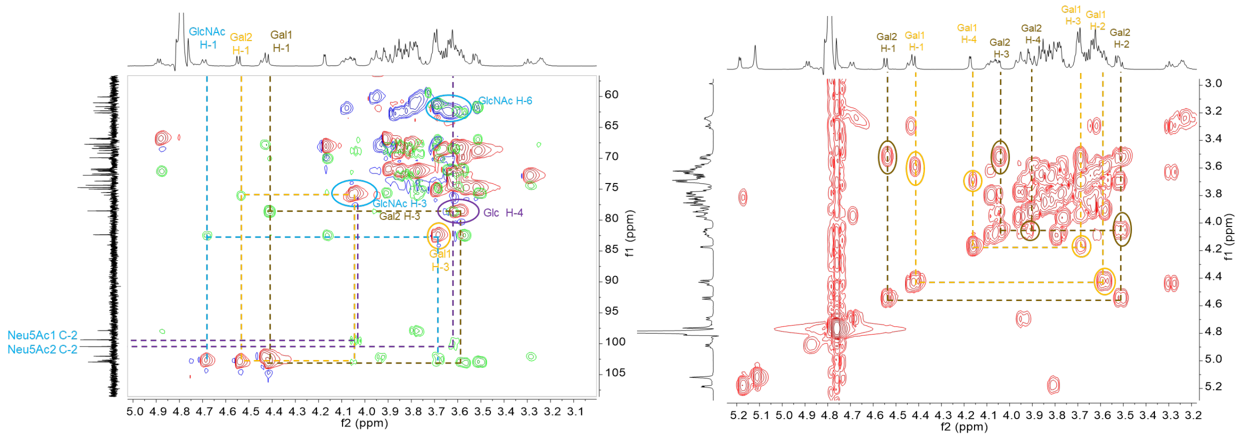

Figure S17. Merged HSQC (in red and blue) and HMBC (in green), along with the ^1H-^1H COSY spectra of Compound DS-LNF II.

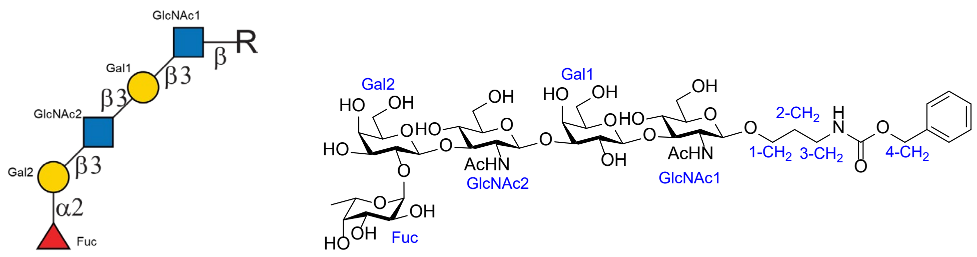

The protons from the linker of BG^{H1}-Le^{C} can be assigned in a manner analogous to those of DS-LNF II, as above delineated. The H-1 of the reducing end GlcNAc1 could be assigned at δ 4.51 by a two-bond coupling signal with 1-CH_2 (δ 3.93/3.62) of linker in the merged HSQC and HMBC spectra (**Figure S18**). Given that Gal1 C-1 was linked with GlcNAc1 via a β1-3 glycosidic bond, the anomeric Gal1 C-1 has correlation with GlcNAc1 H-3 (δ 3.79). GlcNAc2 C-1 has correlation with Gal1 H-3 assigned at (δ 3.72). The anomeric Gal2 C-1 has correlation with GlcNAc2 H-3 assigned at (δ 4.01). Fuc C-1 was linked with Gal via a α1-2 glycosidic bond, the anomeric Fuc C-1 has correlation with Gal2 H-2 that could be assigned at δ 3.61.

Additionally, from merged HSQC-TOCSY and HMBC spectra, along with ^{1}H-^{1}H COSY spectra (**Figure S19-20**), Gal1 H-2 was assigned at δ 3.56 which also has clear correlation with Gal1 H-1 in TOCSY spectra, same with Gal2 protons. All these information confirms that Fucose has correlation with Gal2 H-2 rather than Gal1 H-2.

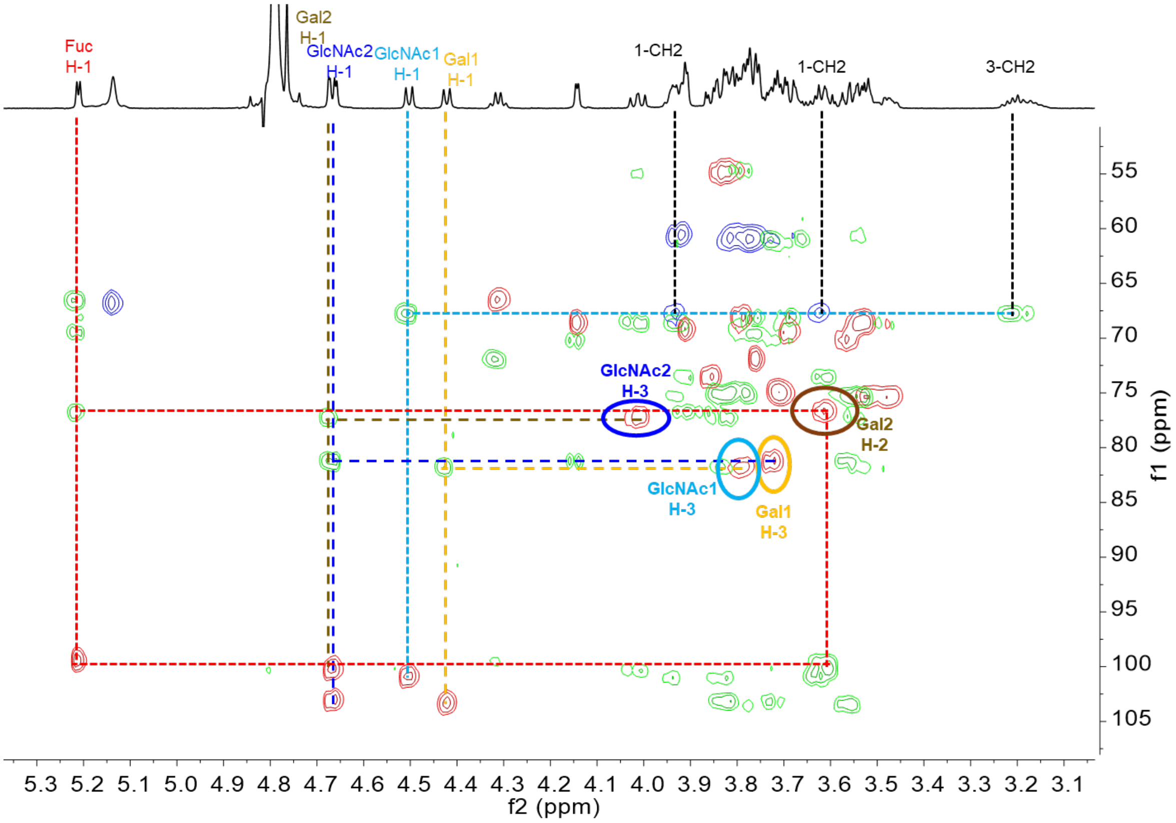

Figure S18. Merged HSQC (in red and blue) and HMBC (in green) spectra of Compound BG^{H1}-Le^{C}.

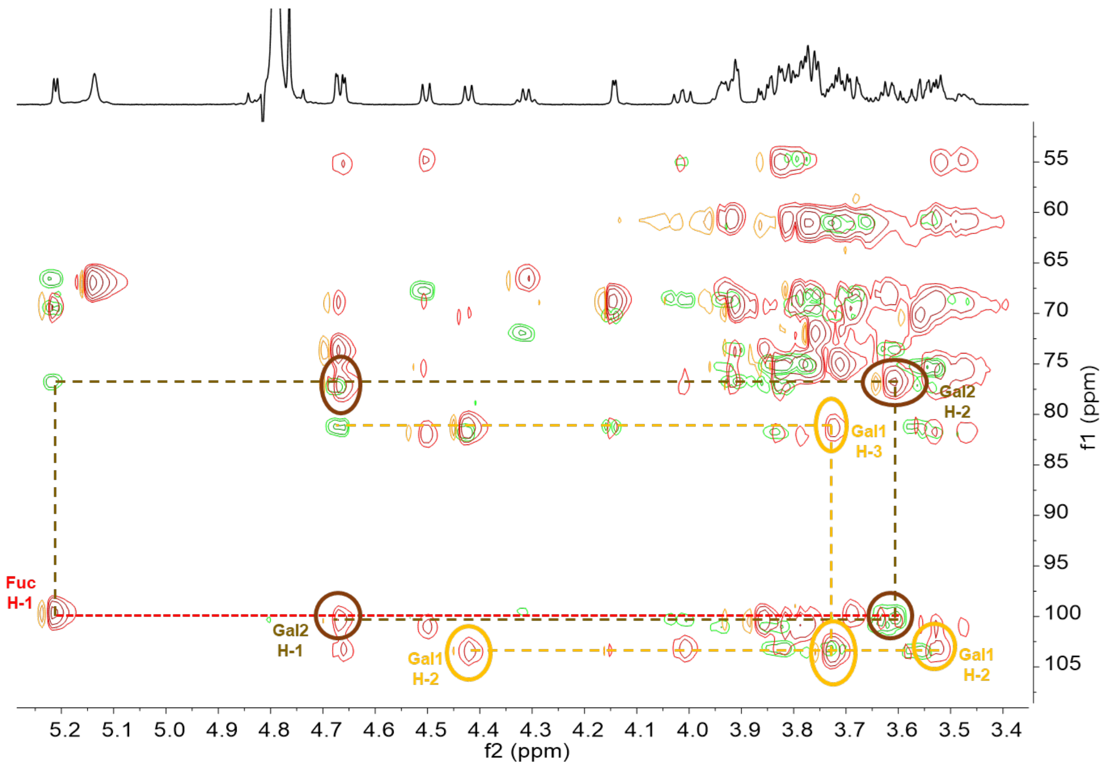

Figure S19. Merged HSQC-TCOSY (in red) and HMBC (in green) spectra of Compound BG^{H1}-Le^{C}.

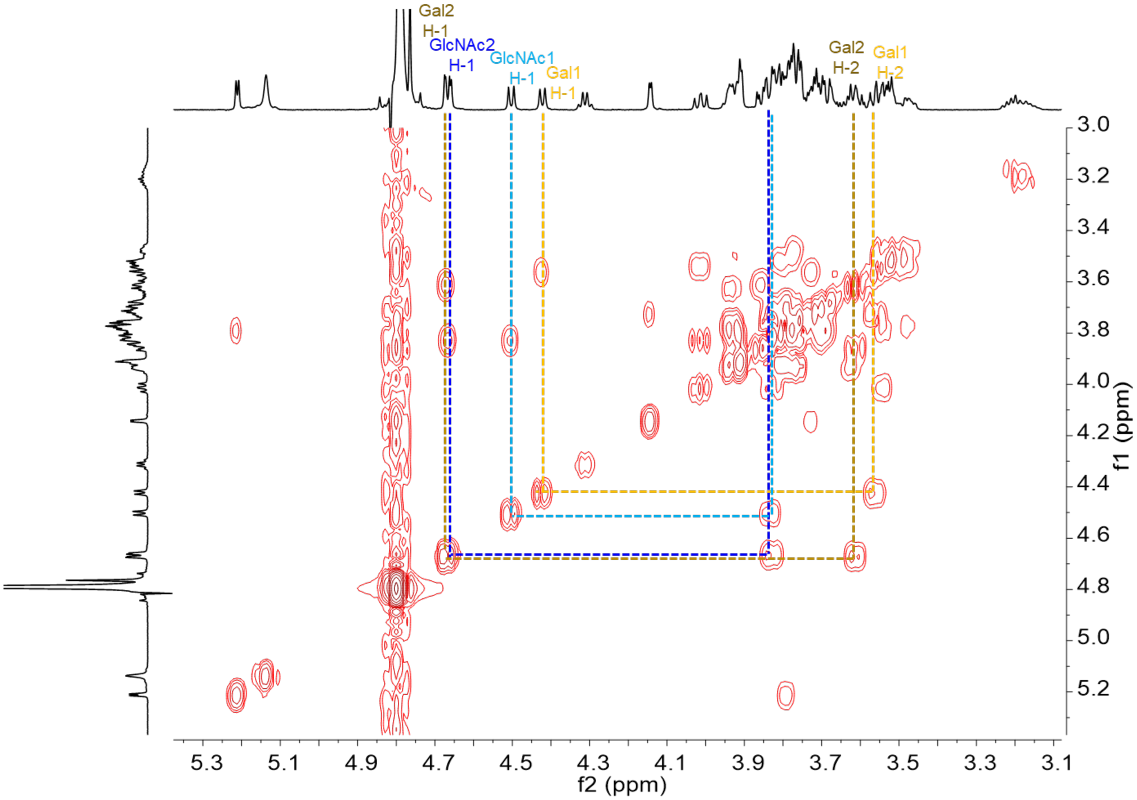

Figure S20. ^{1}H-^{1}H COSY spectra of Compound BG^{H1}-Le^{C}.

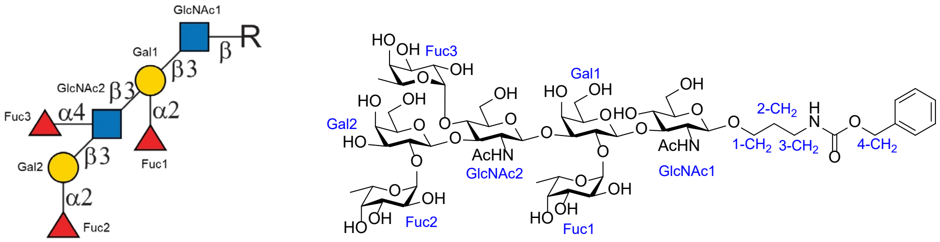

The protons from the linker of Le^B-BG^H1 can be assigned in a manner analogous to those of DS-LNF II, as above delineated. The H-1 of the reducing end GlcNAc1 could be assigned at (δ 4.47, 101.07) by a two-bond coupling signal with 1-CH_2 (δ 3.90/3.60, 67.78) of linker in the merged HSQC and HMBC spectra (**Figure S21**). Given that Gal1 C-1 was linked with GlcNAc1 via a β1-3 glycosidic bond, the anomeric Gal1 C-1 (δ 4.46, 102.68) has correlation with GlcNAc1 H-3 that could be assigned at (δ 4.06, 75.40). GlcNAc2 C-1 (δ 4.65, 103.01) has correlation with Gal1 H-3 assigned at (δ 3.67, 80.67). The anomeric Gal2 C-1 (δ 4.47, 100.60) has correlation with GlcNAc2 H-3 assigned at (δ 4.14, 74.45). Fuc C-1 was linked with Gal via a α1-2 glycosidic bond, the anomeric Fuc3 C-1 (δ 5.03, 97.80) has correlation with GlcNAc2 H-4 assigned at (δ 3.73, 72.01). Fuc2 C-1 (δ 5.16, 99.55) has correlation with Gal2 H-2 that could be assigned at (δ 3.60, 76.44).

All the H-2 could be assigned as shown in ^{1}H-^{1}H COSY spectra (**Figure S22**), GlcNAc1 H-2 has the correlation with GlcNAc1 H-1 at δ 3.87, and H-3 of GlcNAc1 could be assigned at δ 4.06. Gal1 H-2 has the correlation with Gal1 H-1 at δ 3.47, and H-3 and H-4 of Gal1 could be assigned at δ 4.07 and δ 3.51, respectively. GlcNAc2 H-2 has the correlation with GlcNAc2 H-1 at δ 3.81, and H-3 and H-4 of GlcNAc2 could be assigned at δ 4.14 and δ 3.73. Gal2 H-2 has the correlation with Gal1 H-1 at δ 3.60. H-2 of Fuc1 and Fuc3 has the correlation with H-1 at δ 3.80. H-2 of Fuc2 has the correlation with Fuc2 H-1 at δ 3.75.

Some other protons signals could be assigned from merged HSQC-TOCSY and HMBC (**Figure S23**). The H-5 of GlcNAc1 and GlcNAc2 could be assigned at δ 3.55 and δ 3.53. It is noteworthy that Fuc3 C-1 has correlation with H-4 of GlcNAc2 in this merged map, which also suggests that Fuc3 is linked to GlcNAc2 rather than GlcNAc1. The remaining protons that show intra-spin system signals were assigned by COSY, HSQC, HSQC-TOCSY and Merged HSQC-HMBC analysis.

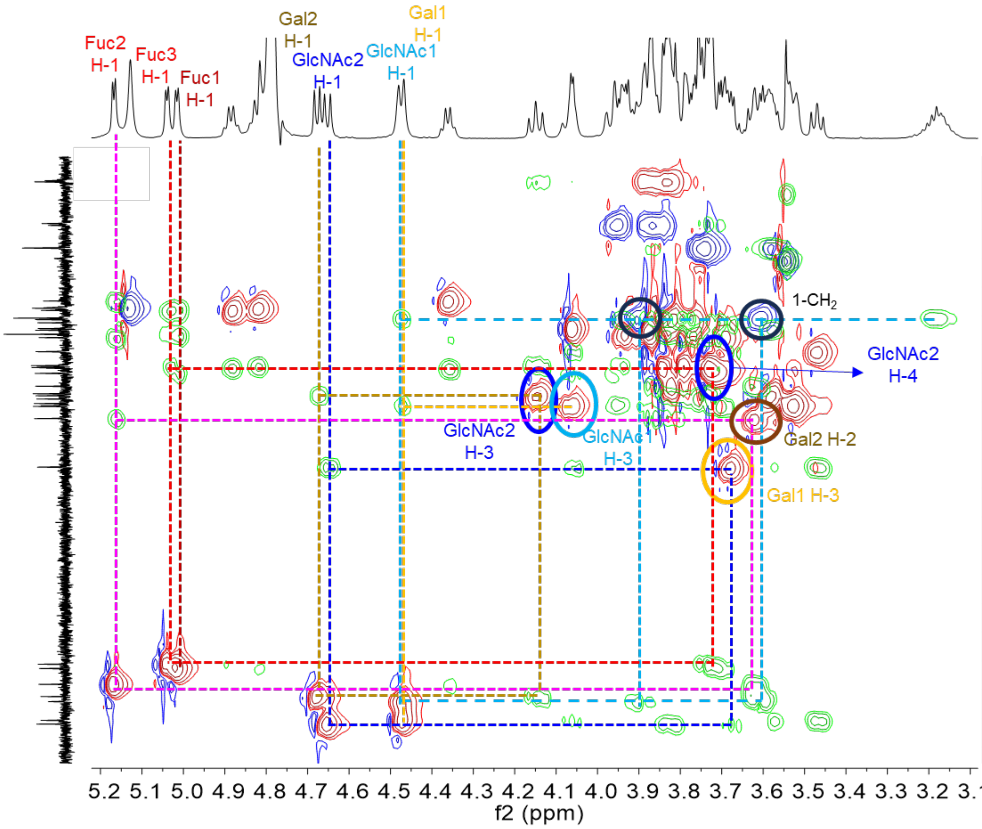

Figure S21. Merged HSQC (in red and blue) and HMBC (in green) spectra of Compound Le^{B}-BG^{H1}.

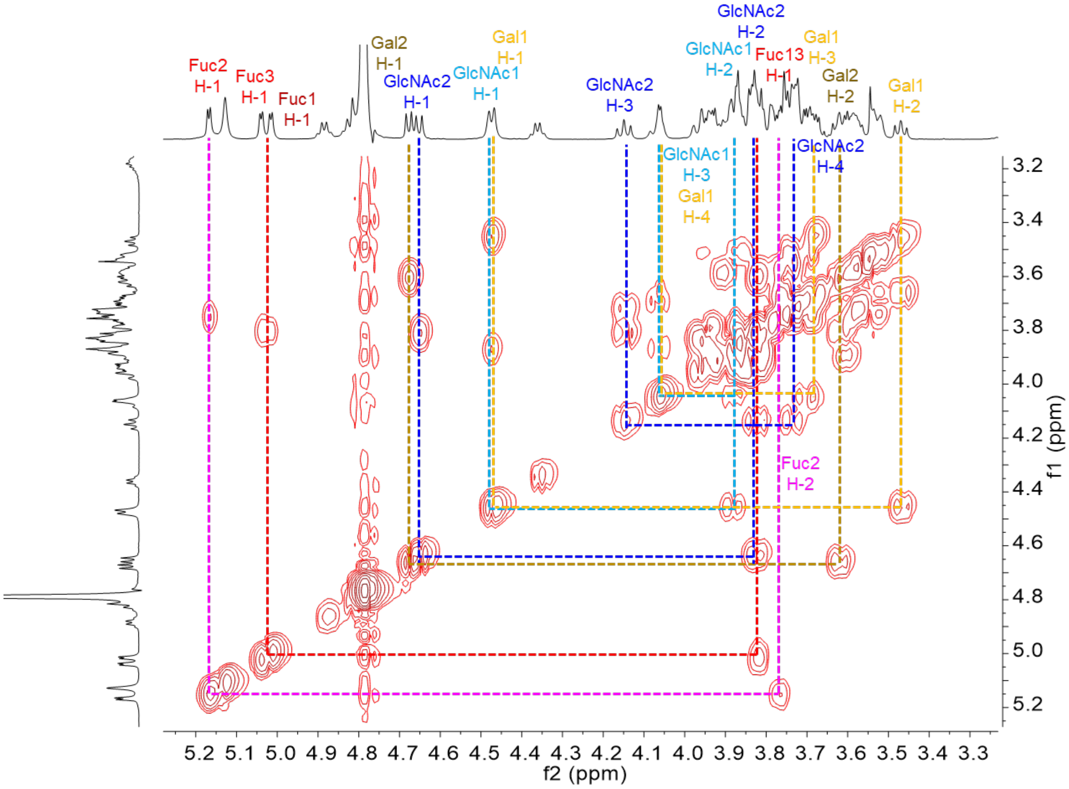

Figure S22. ^{1}H-^{1}H COSY spectra of Compound Le^{B}-BG^{H1}.

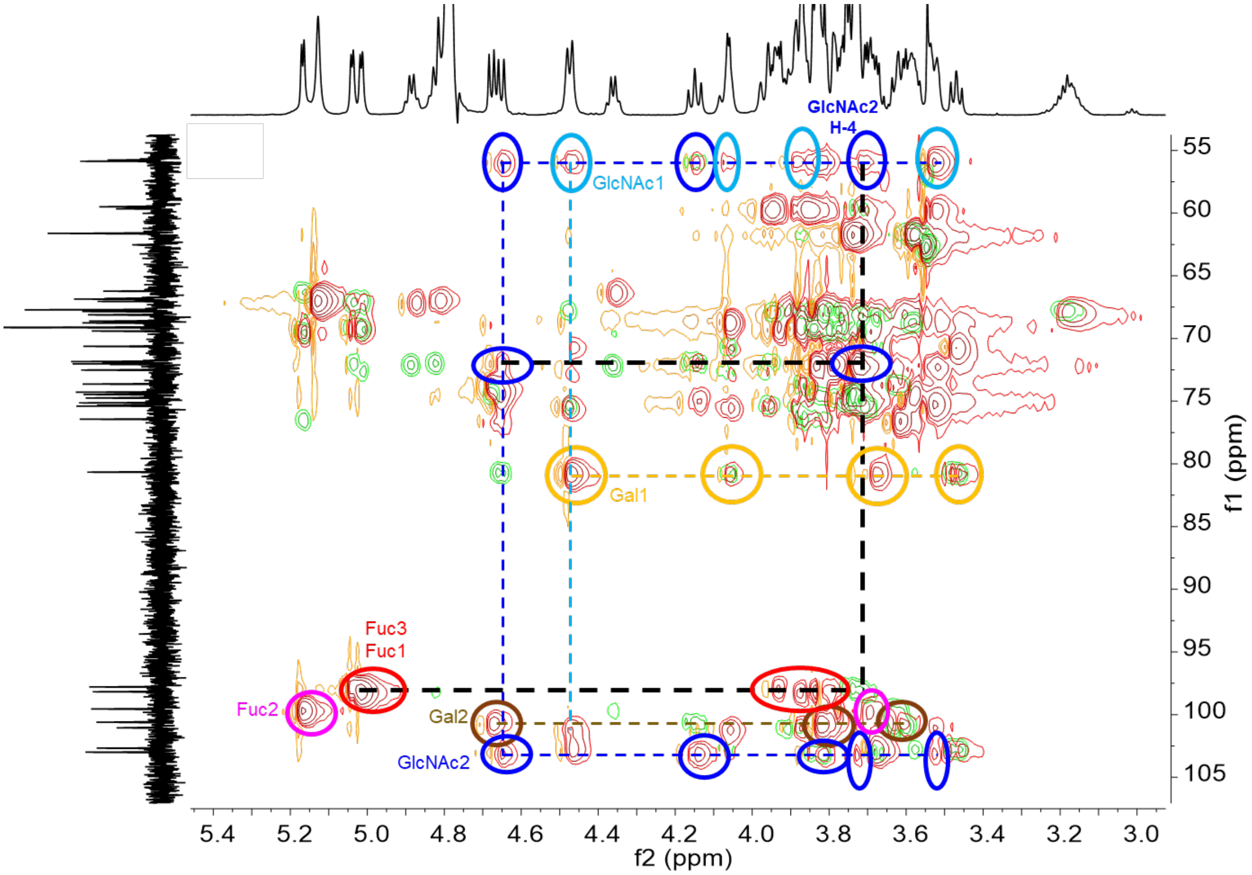

Figure S23. Merged HSQC-TOCSY (in red) and HMBC (in green) spectra of Compound Le^{B}-BG^{H1}.

*NMR assignment and MS data of compound DS-LNF II (**3**):*

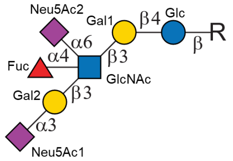

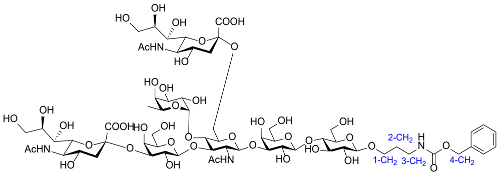

^{1}H NMR (600 MHz, D_2O): δ (ppm)

	Proton							Anomeric carbon
	H-1	H-2	H-3	H-4	H-5	H-6	NHAc	
Glc	4.45	3.30	3.62	3.61	n/a[a]	3.96	-[b]	102.02
Gal1	4.42	3.59	3.69	4.17	n/a	3.84	-	102.74
GlcNAc	4.69	3.94	4.07	3.79	3.62	4.04, 3.52	2.04-2.02	102.63
Gal2	4.54	3.54	4.08	3.91	n/a	n/a	-	102.83
Fuc	5.18	3.83	3.86	3.78	4.88	1.18	-	97.81
Neu5Ac1[c]	-	-	2.78, 1.76	3.67	3.85	3.62	2.04-2.02	100.53
Neu5Ac2[c]	-	-	2.78, 1.73	3.68	3.83	3.63	2.04-2.02	99.37

[a] Not assigned. [b] Not applicable. [c] The H-7 to H-9 of sialic acid are not assigned.

Linker	1-CH2	2-CH2	3-CH2	4-CH2
Chemical Shift (H)	3.91, 3.65	1.76	3.17	5.12
Chemical Shift (C)	67.73	28.94	37.38	66.83

HRMS (ESI) m/z calcd for $C_{65}H_{102}N_4O_{43}$ $[M-H]^-$ 1625.5845, found 1625.5840.

*NMR assignment and MS data of compound A-Le^B (**11**):*

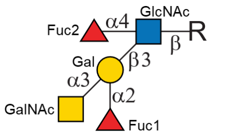

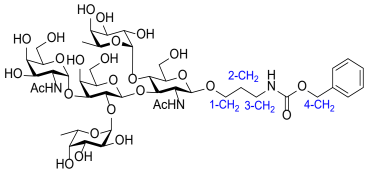

^{1}H NMR (600 MHz, D_2O): δ (ppm)

	Proton							Anomeric carbon
	H-1	H-2	H-3	H-4	H-5	H-6	NHAc	
GlcNAc	4.37	3.84	4.16	3.72	3.53	3.97, 3.88	2.04-2.02	101.92
Gal	4.72	3.85	3.78	4.32	n/a[a]	n/a	-[b]	100.39
GalNAc	5.25	4.21	3.99	3.87	n/a	n/a	2.04-2.02	90.90
Fuc1	5.23	3.76	3.78	3.70	4.86	1.30	-	99.27
Fuc2	5.05	3.85	3.94	3.82	4.40	1.29	-	97.88

[a] Not assigned. [b] Not applicable.

Linker	1-CH2	2-CH2	3-CH2	4-CH2
Chemical Shift (H)	3.90, 3.57	1.74	3.16	5.14
Chemical Shift (C)	67.76	28.98	37.26	66.79

HRMS (ESI) m/z calcd for $C_{45}H_{71}N_3O_{26}$ $[M+H]^+$ 1070.4399, found 1070.4347.

*NMR assignment and MS data of compound B-Le^B (**12**):*

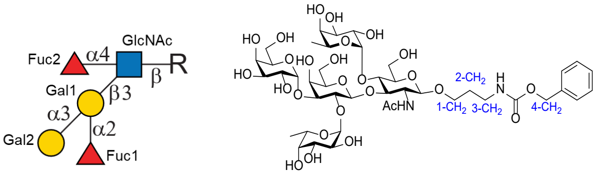

^{1}H NMR (600 MHz, D_2O): δ (ppm)

	Proton							Anomeric carbon
	H-1	H-2	H-3	H-4	H-5	H-6	NHAc	
GlcNAc	4.37	3.83	4.16	3.73	3.53	3.97, 3.87	2.04-2.02	101.92
Gal1	4.71	3.84	3.95	4.26	n/a[a]	n/a	-[b]	100.60
Gal2	5.26	3.91	3.93	n/a	n/a	n/a	-	93.30
Fuc1	5.19	3.77	3.80	3.71	4.39	1.29	-	99.32
Fuc2	5.05	3.84	3.96	3.85	4.84	1.29	-	97.77

[a] Not assigned. [b] Not applicable.

Linker	1-CH2	2-CH2	3-CH2	4-CH2
Chemical Shift (H)	3.92, 3.57	1.75	3.16	5.13
Chemical Shift (C)	67.75	29.00	37.31	66.79

HRMS (ESI) m/z calcd for $C_{43}H_{68}N_2O_{26}$ $[M+H]^+$ 1029.4133, found 1029.4081.

*NMR assignment and MS data of compound 3'-sulfo-Le^A (**14**):*

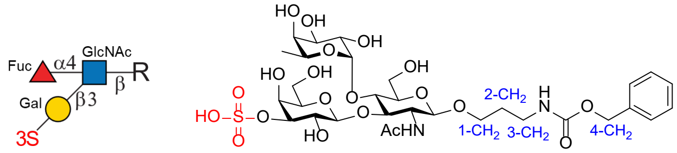

^{1}H NMR (600 MHz, D_2O): δ (ppm)

	Proton							Anomeric carbon
	H-1	H-2	H-3	H-4	H-5	H-6	NHAc	
GlcNAc	4.52	3.89	4.11	3.74	3.55	3.97, 3.85	2.04-2.02	100.86
Gal	4.60	3.63	4.31	4.30	3.65	n/a[a]	-[b]	102.53
Fuc	5.03	3.83	3.91	3.81	4.86	1.20	-	97.97

[a] Not assigned. [b] Not applicable.

Linker	1-CH2	2-CH2	3-CH2	4-CH2
Chemical Shift (H)	3.93, 3.62	1.76	3.19	5.14
Chemical Shift (C)	67.71	28.88	37.28	66.79

HRMS (ESI) m/z calcd for $C_{31}H_{48}N_2O_{20}S$ $[M-H]^-$ 799.2448, found 799.2620.

*NMR assignment and MS data of compound DSLe^{A} (**18**):*

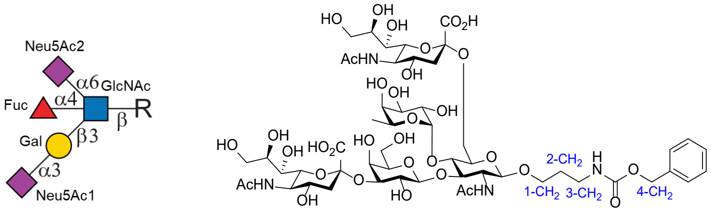

^{1}H NMR (600 MHz, D_2O): δ (ppm)

	Proton							Anomeric carbon
	H-1	H-2	H-3	H-4	H-5	H-6	NHAc	
GlcNAc	4.47	3.85	4.01	3.76	3.63	4.07, 3.74	2.04-2.02	100.97
Gal	4.50	3.51	4.05	3.90	n/a[a]	3.86, 3.64	-[b]	102.87
Fuc	5.17	3.81	3.86	3.78	4.86	1.18	-	97.83
Neu5Ac1[c]	-	-	2.76, 1.74	3.66	3.83	3.64	2.04-2.02	n/a
Neu5Ac2[c]	-	-	2.76, 1.74	3.67	3.83	3.62	2.04-2.02	n/a

[a] Not assigned. [b] Not applicable. [c] The H-7 to H-9 of sialic acid are not assigned.

Linker	1-CH2	2-CH2	3-CH2	4-CH2
Chemical Shift (H)	3.90, 3.59	1.74	3.17	5.13
Chemical Shift (C)	67.87	28.87	37.33	66.79

HRMS (ESI) m/z calcd for $C_{53}H_{82}N_4O_{33}$ $[M-H]^-$ 1301.4789, found 1301.4783.

*NMR assignment and MS data of compound 6SLe^{B} (**25**):*

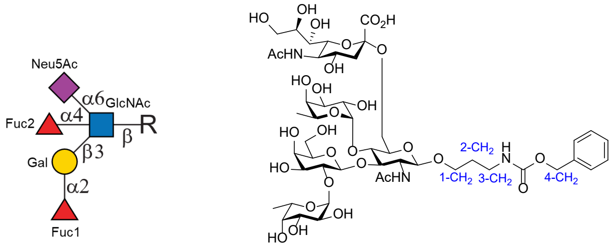

^{1}H NMR (600 MHz, D_2O): δ (ppm)

	Proton							Anomeric carbon
	H-1	H-2	H-3	H-4	H-5	H-6	NHAc	
GlcNAc	4.34	3.80	4.07	3.72	3.62	4.10, 3.74	2.04-2.02	101.97
Gal	4.64	3.61	3.80	n/a[a]	3.64	3.89, 3.63	-[b]	100.62
Fuc1	5.19	3.83	3.92	3.86	4.87	1.27	-	97.67
Fuc2	5.15	3.75	n/a	3.77	4.33	1.26	-	99.50
Neu5Ac [c]	-	-	2.76, 1.75	3.65	3.83	3.70	2.04-2.02	100.55

[a] Not assigned. [b] Not applicable. [c] The H-7 to H-9 of sialic acid are not assigned.

Linker	1-CH2	2-CH2	3-CH2	4-CH2
Chemical Shift (H)	3.82, 3.52	1.73	3.15	5.12
Chemical Shift (C)	67.78	28.99	37.35	66.79

HRMS (ESI) m/z calcd for $C_{48}H_{75}N_3O_{29}$ $[M-H]^-$ 1156.4413, found 1156.4408.

*NMR assignment and MS data of compound Le^B-Le^A (**32**):*

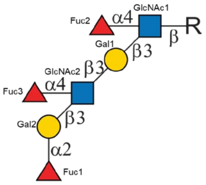

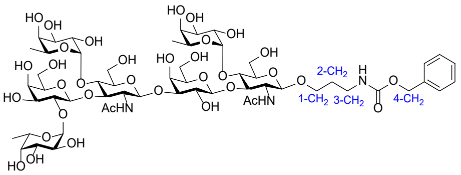

^{1}H NMR (600 MHz, D_2O): δ (ppm)

	Proton							Anomeric carbon
	H-1	H-2	H-3	H-4	H-5	H-6	NHAc	
GlcNAc1	4.47	3.88	4.06	3.72	3.52	3.95, 3.86	2.04-2.02	100.95
Gal1	4.47	3.46	3.68	4.07	3.45	n/a[a]	-[b]	102.51
GlcNAc2	4.65	3.82	4.14	3.73	n/a	n/a	2.04-2.02	102.90
Gal2	4.68	3.62	3.82	n/a	n/a	3.74	-	100.48
Fuc1	5.17	3.76	3.69	n/a	4.36	1.28	-	99.43
Fuc2	5.02	3.81	3.93	3.80	4.82	1.16	-	97.95
Fuc3	5.04	3.81	3.84	3.88	4.88	1.27	-	97.48

[a] Not assigned. [b] Not applicable.

Linker	1-CH$_2$	2-CH$_2$	3-CH$_2$	4-CH$_2$
Chemical Shift (H)	3.90, 3.60	1.75	3.17	5.13
Chemical Shift (C)	67.69	28.84	37.25	66.79

HRMS (ESI) m/z calcd for $C_{57}H_{91}N_3O_{35}$ $[M+2H]^{2+}$ 1379.5579, found 1379.5562.

*NMR assignment and MS data of compound Le^A-BGH (**35**):*

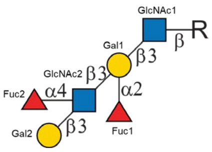

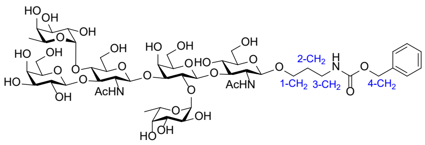

^{1}H NMR (600 MHz, D_2O): δ (ppm)

	Proton							Anomeric carbon
	H-1	H-2	H-3	H-4	H-5	H-6	NHAc	
GlcNAc1	4.49	3.88	4.06	3.77	n/a[a]	n/a	2.04-2.02	101.02
Gal1	4.48	3.50	3.69	4.07	n/a	n/a	-[b]	102.69
GlcNAc2	4.75	3.95	4.07	3.72	3.54	3.95, 3.85	2.04-2.02	102.41
Gal2	4.53	3.49	3.63	3.90	n/a	3.74	-	102.85
Fuc1	5.01	3.81	3.84	3.88	4.82	1.16	-	98.07
Fuc2	5.04	3.81	3.84	3.88	4.89	1.19	-	98.11

[a] Not assigned. [b] Not applicable.

Linker	1-CH$_2$	2-CH$_2$	3-CH$_2$	4-CH$_2$
Chemical Shift (H)	3.92, 3.61	1.75	3.17	5.13
Chemical Shift (C)	67.71	28.91	37.30	66.79

HRMS (ESI) m/z calcd for $C_{51}H_{81}N_3O_{31}$ $[M+2H]^{2+}$ 1233.5000, found 1233.4934.

*NMR assignment and MS data of compound Le^{B}-BGH (**36**):*

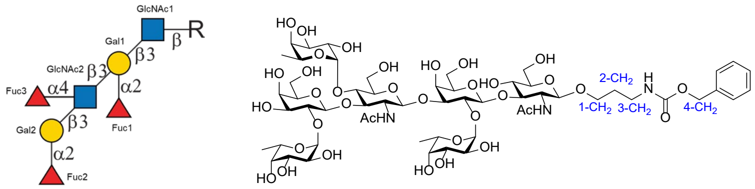

^{1}H NMR (600 MHz, D_2O): δ (ppm)

	Proton							Anomeric carbon
	H-1	H-2	H-3	H-4	H-5	H-6	NHAc	
GlcNAc1	4.47	3.87	4.06	n/a[a]	3.55	3.95, 3.87	2.04-2.02	101.06
Gal1	4.46	3.47	3.67	4.07	3.51	3.74	-[b]	102.56
GlcNAc2	4.65	3.81	4.14	3.73	3.53	n/a	2.04-2.02	100.58
Gal2	4.67	3.60	3.82	n/a	n/a	n/a	-	102.94
Fuc1	5.02	3.80	3.84	3.87	4.82	1.16	-	98.02
Fuc2	5.16	3.75	3.69	n/a	4.36	1.27	-	99.47
Fuc3	5.03	3.80	3.84	3.93	4.88	1.29	-	97.79

[a] Not assigned. [b] Not applicable.

Linker	1-CH_2	2-CH_2	3-CH_2	4-CH_2
Chemical Shift (H)	3.90, 3.61	1.75	3.17	5.13
Chemical Shift (C)	67.78	28.88	37.30	66.79

HRMS (ESI) m/z calcd for $C_{57}H_{91}N_3O_{35}$ $[M+2H]^{2+}$ 1379.5579, found 1379.5562.

NMR and MS data of other Glycans:

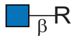

Compound **4** was obtained as a white solid (150 mg); ^{1}H NMR (600 MHz, D_2O) δ 7.45 – 7.37 (m, 5H), 5.13 (s, 2H), 4.46 (d, *J* = 8.5 Hz, 1H), 3.94 – 3.92 (m, 2H), 3.78 – 3.72 (m, 1H), 3.69 (m, 1H), 3.66 – 3.58 (m, 1H), 3.54 (m, 1H), 3.45 (m, 2H), 3.23 – 3.13 (m, 2H), 2.04 (s, 3H), 1.76 (p, *J* = 6.0 Hz, 2H). HRMS (ESI) m/z calcd for $C_{19}H_{28}N_2O_{18}$ $[M+H]^+$ 413.1918, found 413.1932.

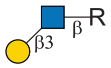

Compound **5** was obtained as a white solid (203 mg); ^{1}H NMR (600 MHz, D_2O) δ 7.44 – 7.37 (m, 5H), 5.12 (s, 2H), 4.50 (d, *J* = 8.3 Hz, 1H), 4.43 (d, *J* = 7.8 Hz, 1H), 3.96 – 3.89 (m, 3H), 3.85 – 3.70 (m, 6H), 3.66 – 3.59 (m, 2H), 3.56 – 3.51 (m, 2H), 3.49 – 3.44 (m, 1H), 3.23 – 3.12 (m, 2H), 2.04 (s, 3H), 1.75 (p, *J* = 6.1 Hz, 2H). HRMS (ESI) m/z calcd for $C_{25}H_{38}N_2O_{13}$ $[M+H]^+$ 575.2447, found 575.2452.

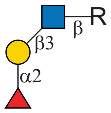

Compound **6** was obtained as a white solid (51 mg); ^{1}H NMR (600 MHz, D_2O) δ 7.50 – 7.37 (m, 5H), 5.18 (d, *J* = 3.8 Hz, 1H), 5.12 (s, 2H), 4.63 (d, *J* = 7.6 Hz, 1H), 4.36 (d, *J* = 8.3 Hz, 1H), 4.29 (q, *J* = 6.4 Hz, 1H), 3.98 – 3.86 (m, 4H), 3.85 – 3.71 (m, 7H), 3.70 – 3.62 (m, 2H), 3.61 – 3.50 (m, 2H), 3.47 (d, *J* = 4.9 Hz, 2H), 3.23 –3.13 (m, 2H), 2.04 (s, 3H), 1.74 (p, *J* = 6.3 Hz, 2H), 1.19 (d, *J* = 6.5 Hz, 3H). HRMS (ESI) m/z calcd for $C_{31}H_{48}N_2O_{17}$ $[M+H]^+$ 721.3026, found 721.3031.

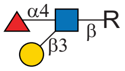

Compound **7** was obtained as a white solid (7.3 mg); ^{1}H NMR (600 MHz, D_2O) δ 7.45 – 7.37 (m, 5H), 5.12 (s, 2H), 5.01 (d, *J* = 4.0 Hz, 1H), 4.86 (q, J = 6.4 Hz, 1H), 4.49 (d, *J* = 7.8 Hz, 2H), 4.04 (m, 1H), 3.95 (dd, *J* = 12.4, 2.3 Hz, 1H), 3.94 – 3.79 (m, 7H), 3.77 – 3.70 (m, 3H), 3.66 – 3.56 (m, 3H), 3.52 –3.48 (m, 1H), 3.48 (dd, *J* = 9.8, 7.7 Hz, 1H), 3.23 – 3.13 (m, 2H), 2.02 (s, 3H), 1.76 (p, *J* = 6.2 Hz, 2H), 1.18 (d, *J* = 6.6 Hz, 3H). HRMS (ESI) m/z calcd for $C_{31}H_{48}N_2O_{17}$ $[M+H]^+$ 721.3026, found 721.3031.

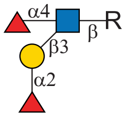

Compound **8** was obtained as a white solid (12 mg); ^{1}H NMR (600 MHz, D_2O) δ 7.51 – 7.40 (m, 5H), 5.15 (d, *J* = 3.9 Hz, 1H), 5.13 (s, 2H), 5.01 (d, *J* = 3.9 Hz, 1H), 4.85 (q, *J* = 6.6 Hz, 1H), 4.64 (d, *J* = 7.7 Hz, 1H), 4.40 – 4.32 (m, 2H), 4.10 (t, *J* = 9.9 Hz, 1H), 4.00 – 3.50 (m, 19H), 3.22 – 3.08 (m, 2H), 2.04 (s, 3H), 1.74 (p, *J* = 6.3 Hz, 2H), 1.27 (d, *J* = 6.5 Hz, 6H). HRMS (ESI) m/z calcd for $C_{37}H_{58}N_2O_{21}$ $[M+H]^+$ 867.3605, found 867.3610.

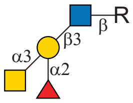

Compound **9** was obtained as a white solid (10 mg); ^{1}H NMR (600 MHz, D_2O) δ 7.45 – 7.37 (m, 5H), 5.27 (d, *J* = 4.2 Hz, 1H), 5.20 (d, *J* = 3.8 Hz, 1H), 5.14 (s, 2H), 4.71 (d, *J* = 7.6 Hz, 1H), 4.39 – 4.31 (m, 3H), 4.25 – 4.23 (m, 2H), 4.05 – 3.88 (m, 6H), 3.84 – 3.75 (m, 9H), 3.70 – 3.63 (m, 2H), 3.60 – 3.55 (m, 1H), 3.52 – 3.48 (m, 2H), 3.23 – 3.13 (m, 2H), 2.04 – 2.02 (m, 6H), 1.78 – 1.70 (m, 2H), 1.25 (d, *J* = 6.5 Hz, 3H). HRMS (ESI) m/z calcd for $C_{39}H_{61}N_3O_{22}$ $[M+H]^+$ 924.3819, found 924.3836.

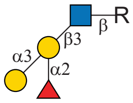

Compound **10** was obtained as a white solid (9 mg); ^{1}H NMR (600 MHz, D_2O) δ 7.51 – 7.40 (m, 5H), 5.25 (d, *J* = 3.8 Hz, 1H), 5.23 (d, *J* = 4.2 Hz, 1H), 5.13 (s, 2H), 4.71 (d, *J* = 7.5 Hz, 1H), 4.41 – 4.32 (m, 2H), 4.29 – 4.27 (m, 2H), 4.02 – 3.82 (m, 10H), 3.81 – 3.73 (m, 7H), 3.72 – 3.69 (m, 1H), 3.65 – 3.63 (m, 1H), 3.58 – 3.54 (m, 1H), 3.51 – 3.47 (m, 2H), 3.23 – 3.13 (m, 2H), 2.04 (s, 3H), 1.78 – 1.70 (m, 2H), 1.24 (d, *J* = 6.7 Hz, 3H). HRMS (ESI) m/z calcd for $C_{37}H_{58}N_2O_{22}$ $[M+H]^+$ 883.3554, found 883.3582.

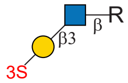

Compound **13** was obtained as a white solid (11 mg); ^{1}H NMR (600 MHz, D_2O) δ 7.50 – 7.40 (m, 5H), 5.13 (s, 2H), 4.56 (d, *J* = 7.8 Hz, 1H), 4.51 (d, *J* = 8.3 Hz, 1H), 4.34 – 4.29 (m, 2H), 3.95 – 3.92 (m, 2H), 3.84 – 3.73 (m, 6H), 3.65 – 3.59 (m, 2H), 3.56 (t, *J* = 9.2 Hz, 1H), 3.48 – 3.46 (m, 1H), 3.23 – 3.13 (m, 2H), 2.02 (s, 3H), 1.76 (p, *J* = 6.1 Hz, 2H). HRMS (ESI) m/z calcd for $C_{25}H_{38}N_2O_{16}S$ $[M-H]^-$ 653.1869, found 653.1864.

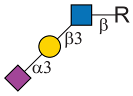

Compound **15** was obtained as a white solid (30 mg); ^{1}H NMR (600 MHz, D_2O) δ 7.50 – 7.39 (m, 5H), 5.12 (s, 2H), 4.50 (t, *J* = 8.3 Hz, 2H), 4.09 (dd, *J* = 9.8, 3.0 Hz, 1H), 3.97 – 3.50 (m, 18H), 3.47 – 3.45 (m, 1H), 3.25 – 3.12 (m, 2H), 2.77 (dd, *J* = 12.4, 4.5 Hz, 1H), 2.04 – 2.02 (m, 6H), 1.83 – 1.69 (m, 3H). HRMS (ESI) m/z calcd for $C_{36}H_{55}N_3O_{21}$ $[M-H]^-$ 864.3255, found 864.3250.

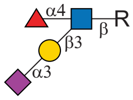

Compound **16** was obtained as a white solid (5.2 mg); ^{1}H NMR (600 MHz, D_2O) δ 7.50 – 7.39 (m, 5H), 5.12 (s, 2H), 5.01 (d, *J* = 3.9 Hz, 1H), 4.87 (q, *J* = 6.6 Hz, 1H), 4.53 (d, *J* = 7.7 Hz, 1H), 4.50 (d, *J* = 8.4 Hz, 1H), 4.07 – 4.04 (m, 2H), 3.96 (d, *J* = 10.9 Hz, 1H), 3.93 – 3.76 (m, 12H), 3.75 – 3.58 (m, 9H), 3.55 – 3.49 (m, 3H), 3.24 – 3.10 (m, 2H), 2.78 (dd, *J* = 12.4, 4.6 Hz, 1H), 2.04 – 2.02 (m, 6H), 1.82 – 1.71 (m, 3H), 1.18 (d, *J* = 6.6 Hz, 3H). HRMS (ESI) m/z calcd for $C_{42}H_{65}N_3O_{25}$ $[M-H]^-$ 1010.3834, found 1010.3829.

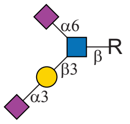

Compound **17** was obtained as a white solid (20 mg); ^{1}H NMR (600 MHz, D_2O) δ 7.50 – 7.38 (m, 5H), 5.12 (s, 2H), 4.47 (d, *J* = 8.1 Hz, 2H), 4.08 (d, *J* = 9.8 Hz, 1H), 3.97 – 3.49 (m, 27H), 3.26 – 3.09 (m, 2H), 2.81 – 2.70 (m, 2H), 2.04 – 2.02 (m, 6H), 1.83 – 1.67 (m, 4H). HRMS (ESI) m/z calcd for $C_{47}H_{72}N_4O_{29}$ $[M-H]^-$ 1155.4209, found 1155.4204.

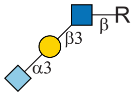

Compound **19** was obtained as a white solid (30 mg); ^{1}H NMR (600 MHz, D_2O) δ 7.48 – 7.35 (m, 5H), 5.09 (s, 2H), 4.49 – 4.47 (m, 2H), 4.13 (s, 2H), 4.07 (dd, *J* = 9.8, 3.2 Hz, 1H), 3.94 – 3.55 (m, 17H), 3.54 – 3.47 (m, 2H), 3.45 – 3.42 (m, 1H), 3.22 – 3.08 (m, 2H), 2.76 (dd, *J* = 12.4, 4.7 Hz, 1H), 2.02 (s, 3H), 1.81 – 1.67 (m, 3H). HRMS (ESI) m/z calcd for $C_{36}H_{55}N_3O_{22}$ $[M-H]^-$ 880.3203, found 880.3199.

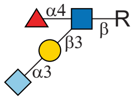

Compound **20** was obtained as a white solid (7.2 mg); ^{1}H NMR (600 MHz, D_2O) δ 7.50 – 7.38 (m, 5H), 5.12 (s, 2H), 5.00 (d, *J* = 3.9 Hz, 1H), 4.87 (q, *J* = 6.6 Hz, 1H), 4.53 (d, *J* = 7.7 Hz, 1H), 4.49 (d, *J* = 8.4 Hz, 1H), 4.13 (s, 2H), 4.07 – 4.04 (m, 2H), 3.99 – 3.68 (m, 15H), 3.63 (m, 4H), 3.56 – 3.48 (m, 3H), 3.24 – 3.09 (m, 2H), 2.78 (dd, *J* = 12.4, 4.6 Hz, 1H), 2.02 (s, 3H), 1.82 – 1.71 (m, 3H), 1.16 (d, *J* = 6.6 Hz, 3H). HRMS (ESI) m/z calcd for $C_{42}H_{66}N_3O_{26}$ $[M-H]^-$ 1026.3784, found 1026.3778.

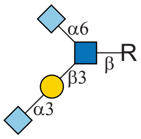

Compound **21** was obtained as a white solid (18 mg); ^{1}H NMR (600 MHz, D_2O) δ 7.49 – 7.39 (m, 5H), 5.12 (s, 2H), 4.49 – 4.47 (m, 2H), 4.13 (s, 4H), 4.08 (dd, *J* = 9.8, 3.0 Hz, 1H), 3.99 – 3.51 (m, 28H), 3.24 – 3.11 (m, 2H), 2.80 – 2.77 (m, 2H), 2.02 (s, 3H), 1.84 – 1.70 (m, 4H). HRMS (ESI) m/z calcd for $C_{47}H_{72}N_4O_{31}$ $[M-H]^-$ 1187.4108, found 1187.4102.

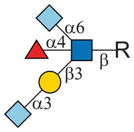

Compound **22** was obtained as a white solid (10 mg); ^{1}H NMR (600 MHz, D_2O) δ 7.50 – 7.40 (m, 5H), 5.18 (d, *J* = 3.8 Hz, 1H), 5.13 (s, 2H), 4.89 (q, *J* = 6.7 Hz, 1H), 4.52 (d, *J* = 7.6 Hz, 1H), 4.48 (d, *J* = 8.2 Hz, 1H), 4.13 (s, 4H), 4.11 – 4.01 (m, 3H), 3.97 – 3.73 (m, 19H), 3.70 – 3.69 (m, 1H), 3.68 – 3.63 (m, 6H), 3.55 – 3.50 (m, 2H), 3.23 – 3.10 (m, 2H), 2.81 – 2.77 (m, 2H), 2.02 (s, 3H), 1.83 – 1.71 (m, 4H), 1.19 (d, *J* = 6.6 Hz, 3H). HRMS (ESI) m/z calcd for $C_{53}H_{82}N_4O_{35}$ $[M-2H]^-$ 1332.4609, found 1332.4614.

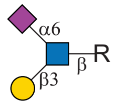

Compound **23** was obtained as a white solid (31 mg); ^{1}H NMR (600 MHz, D_2O) δ 7.50 – 7.39 (m, 5H), 5.13 (s, 2H), 4.47 (d, *J* = 8.4 Hz, 1H), 4.42 (d, *J* = 7.7 Hz, 1H), 4.02 – 3.49 (m, 21H), 3.25 – 3.11 (m, 2H), 2.73 (dd, *J* = 12.4, 4.7 Hz, 1H), 2.04 – 2.02 (m, 6H), 1.79 – 1.70 (m, 3H). HRMS (ESI) m/z calcd for $C_{36}H_{55}N_3O_{21}$ $[M-H]^-$ 864.3255, found 864.3250.

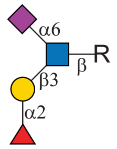

Compound **24** was obtained as a white solid (15 mg); ^{1}H NMR (600 MHz, D_2O) δ 7.43 (m, 5H), 5.18 (d, *J* = 3.9 Hz, 1H), 5.12 (s, 2H), 4.63 (d, *J* = 7.6 Hz, 1H), 4.35 (d, *J* = 8.3 Hz, 1H), 4.30 (q, *J* = 6.6 Hz, 1H), 4.00 – 3.48 (m, 24H), 3.20 – 3.08 (m, 2H), 2.76 (dd, *J* = 12.4, 4.6 Hz, 1H), 2.04 – 2.02 (m, 6H), 1.78 – 1.63 (m, 3H), 1.15 (d, *J* = 6.6 Hz, 3H). HRMS (ESI) m/z calcd for $C_{42}H_{65}N_3O_{25}$ $[M-H]^-$ 1010.3834, found 1010.3829.

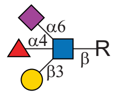

Compound **26** was obtained as a white solid (6.4 mg); ^{1}H NMR (600 MHz, D_2O) δ 7.50 – 7.37 (m, 5H), 5.18 (d, *J* = 3.9 Hz, 1H), 5.12 (s, 2H), 4.87 (q, *J* = 6.6 Hz, 1H), 4.49 – 4.47 (m, 2H), 4.09 (dd, *J* = 10.7, 4.1 Hz, 1H), 4.03 (t, *J* = 9.7 Hz, 1H), 3.94 – 3.55 (m, 21H), 3.49 – 3.47 (m, 1H), 3.23 – 3.10 (m, 2H), 2.73 (dd, *J* = 12.5, 4.7 Hz, 1H), 2.04 – 2.02 (m, 6H), 1.80 – 1.68 (m, 3H), 1.20 (d, *J* = 6.6 Hz, 3H). HRMS (ESI) m/z calcd for $C_{42}H_{65}N_3O_{25}$ $[M-H]^-$ 1010.3834, found 1010.3829.

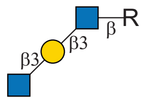

Compound **27** was obtained as a white solid (43 mg);^{1}H NMR (600 MHz, D_2O) δ 7.51 – 7.40 (m, 5H), 5.14 (s, 2H), 4.73 (d, *J* = 8.4 Hz, 1H), 4.51 (d, *J* = 8.3 Hz, 1H), 4.44 (d, *J* = 7.8 Hz, 1H), 4.16 (d, *J* = 2.8 Hz, 1H), 3.97 – 3.89 (m, 3H), 3.86 – 3.69 (m, 9H), 3.67 – 3.43 (m, 7H), 3.26 – 3.12 (m, 2H), 2.04 – 2.02 (m, 6H), 1.81 – 1.71 (m, 2H). HRMS (ESI) m/z calcd for $C_{33}H_{51}N_3O_{18}$ $[M+H]^+$ 778.3240, found 778.3268.

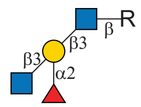

Compound **28** was obtained as a white solid (25 mg); ^{1}H NMR (600 MHz, D_2O) δ 7.51 – 7.40 (m, 5H), 5.14 (s, 2H), 5.02 (d, *J* = 3.7 Hz, 1H), 4.82 (q, *J* = 6.4 Hz, 1H), 4.74 (d, *J* = 8.5 Hz, 1H), 4.49 (d, *J* = 8.0 Hz, 2H), 4.14 – 4.03 (m, 2H), 4.00 – 3.43 (m, 25H), 3.28 – 3.12 (m, 2H), 2.04 – 2.02 (m, 6H), 1.83 – 1.71 (m, 2H), 1.17 (d, *J* = 6.4 Hz, 3H). HRMS (ESI) m/z calcd for $C_{39}H_{61}N_3O_{22}$ $[M+H]^+$ 924.3819, found 924.3826.

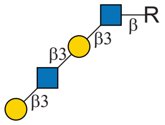

Compound **29** was obtained as a white solid (15 mg); ^{1}H NMR (600 MHz, D_2O) δ 7.53 – 7.41 (m, 5H), 5.15 (s, 2H), 4.52 (d, *J* = 8.3 Hz, 1H), 4.48 (d, *J* = 7.7 Hz, 1H), 4.45 (d, *J* = 7.8 Hz, 1H), 4.17 (d, *J* = 2.9 Hz, 1H), 3.98 – 3.89 (m, 5H), 3.88 – 3.71 (m, 14H), 3.70 – 3.46 (m, 9H), 3.31 – 3.12 (m, 2H), 2.04 – 2.02 (m, 6H), 1.78 (p, *J* = 6.4 Hz, 2H). HRMS (ESI) m/z calcd for $C_{39}H_{61}N_3O_{23}$ $[M+H]^+$ 940.3769, found 940.3795.

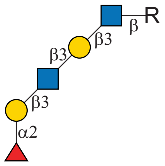

Compound **30** was obtained as a white solid (9.5 mg); ^{1}H NMR (600 MHz, D_2O) δ 7.51 – 7.39 (m, 5H), 5.21 (d, *J* = 4.0 Hz, 1H), 5.14 (s, 2H), 4.68 – 4.65 (m, 2H), 4.50 (d, *J* = 8.3 Hz, 1H), 4.42 (d, *J* = 7.8 Hz, 1H), 4.31 (q, *J* = 6.5 Hz, 1H), 4.14 (d, *J* = 3.0 Hz, 1H), 4.01 (dd, *J* = 10.3, 8.5 Hz, 1H), 3.97 – 3.89 (m, 4H), 3.87 – 3.44 (m, 26H), 3.25 – 3.12 (m, 2H), 2.04 – 2.02 (m, 6H), 1.77 (p, *J* = 6.4, 6.0 Hz, 2H), 1.25 (d, *J* = 6.6 Hz, 3H). HRMS (ESI) m/z calcd for $C_{45}H_{71}N_3O_{27}$ $[M+H]^+$ 1086.4348, found 1086.4282.

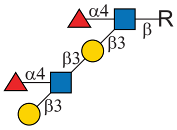

Compound **31** was obtained as a white solid (4.5 mg); ^{1}H NMR (600 MHz, D_2O) δ 7.52 – 7.40 (m, 5H), 5.14 (s, 2H), 5.04 (d, *J* = 3.7 Hz, 1H), 5.02 (d, *J* = 3.7 Hz, 1H), 4.89 (q, *J* = 6.7 Hz, 1H), 4.81 (q, *J* = 6.9 Hz, 1H), 4.74 (d, *J* = 7.5 Hz, 1H), 4.53 (d, *J* = 7.7 Hz, 1H), 4.50 – 4.48 (m, 2H), 4.14 – 4.04 (m, 3H), 4.01 – 3.68 (m, 23H), 3.67 – 3.47 (m, 6H), 3.18 (m, 2H), 2.04 – 2.02 (m, 6H), 1.77 (p, *J* = 6.5 Hz, 2H), 1.19 (d, *J* = 6.5, 3H), 1.16 (d, *J* = 6.6 Hz, 3H). HRMS (ESI) m/z calcd for $C_{51}H_{81}N_3O_{31}$ $[M+2H]^{2+}$ 1233.5000, found 1233.4934.

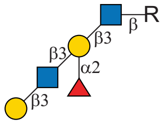

Compound **33** was obtained as a white solid (22 mg); ^{1}H NMR (600 MHz, D_2O) δ 7.55 – 7.39 (m, 5H), 5.14 (s, 2H), 5.02 (d, *J* = 4.0 Hz, 1H), 4.82 (q, *J* = 6.3 Hz, 1H), 4.78 (d, *J* = 8.5 Hz, 1H), 4.52 – 4.48 (m, 3H), 4.13 – 4.07 (m, 2H), 3.99 – 3.71 (m, 20H), 3.70 – 3.49 (m, 7H), 3.25 – 3.15 (m, 2H), 2.04 – 2.02 (m, 6H), 1.82 – 1.74 (m, 2H), 1.19 (d, *J* = 6.6 Hz, 3H). HRMS (ESI) m/z calcd for $C_{45}H_{71}N_3O_{27}$ $[M+H]^+$ 1086.4348, found 1086.4282.

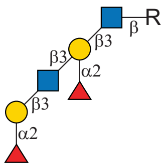

Compound **34** was obtained as a white solid (10 mg); ^{1}H NMR (600 MHz, D_2O) δ 7.52 – 7.41 (m, 5H), 5.20 (d, *J* = 3.9 Hz, 1H), 5.13 (s, 2H), 5.01 (d, *J* = 3.8 Hz, 1H), 4.83 (q, *J* = 6.2 Hz, 1H), 4.69 – 4.66 (m, 2H), 4.49 –4.48 (m, 2H), 4.32 (q, *J* = 6.6 Hz, 1H), 4.12 – 4.05 (m, 2H), 4.05 – 3.95 (m, 2H), 3.94 – 3.65 (m, 22H), 3.65 – 3.57 (m, 3H), 3.56 – 3.46 (m, 3H), 3.24 – 3.11 (m, 2H), 2.04 – 2.02 (m, 6H), 1.77 (p, *J* = 6.6 Hz, 2H), 1.26 (d, *J* = 6.6 Hz, 3H), 1.17 (d, *J* = 6.6 Hz, 3H). HRMS (ESI) m/z calcd for $C_{51}H_{81}N_3O_{31}$ $[M+2H]^{2+}$ 1233.5000, found 1233.4934.

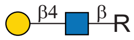

LN was obtained as a white solid (15 mg); ^{1}H NMR (600 MHz, D_2O) δ 7.51 – 7.39 (m, 5H), 5.13 (s, 2H), 4.49 – 4.47 (m, 2H), 3.98 (d, *J* = 11.2 Hz, 1H), 3.96 – 3.89 (m, 2H), 3.82 – 3.65 (m, 7H), 3.63 – 3.59 (m, 1H), 3.59 – 3.52 (m, 2H), 3.24 – 3.11 (m, 2H), 2.03 (s, 3H), 1.75 (p, *J* = 6.3 Hz, 2H). HRMS (ESI) m/z calcd for $C_{25}H_{38}N_2O_{13}$ $[M+H]^+$ 575.2447, found 575.2452.

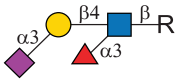

SLe^{X} was obtained as a white solid (5 mg); ^{1}H NMR (600 MHz, D_2O) δ 7.51 – 7.39 (m, 5H), 5.12 (s, 2H), 5.10 (d, *J* = 3.5 Hz, 1H), 4.52 (d, *J* = 7.8 Hz, 1H), 4.48 (d, *J* = 8.2 Hz, 1H), 4.09 (dd, *J* = 9.8, 2.9 Hz, 1H), 4.00 (d, *J* = 11.1 Hz, 1H), 3.96 – 3.76 (m, 12H), 3.75 – 3.48 (m, 12H), 3.17 (s, 2H), 2.77 (dd, *J* = 12.5, 4.6 Hz, 1H), 2.04 – 2.02 (m, 6H), 1.84 – 1.71 (m, 3H), 1.18 (d, *J* = 6.5 Hz, 3H). HRMS (ESI) m/z calcd for $C_{42}H_{65}N_3O_{25}$ $[M-H]^-$ 1010.3834, found 1010.3817.

V. HPLC Analysis of Synthesized Glycans

DS-LNF V (Compound **2**), Retention time = 11.1 min

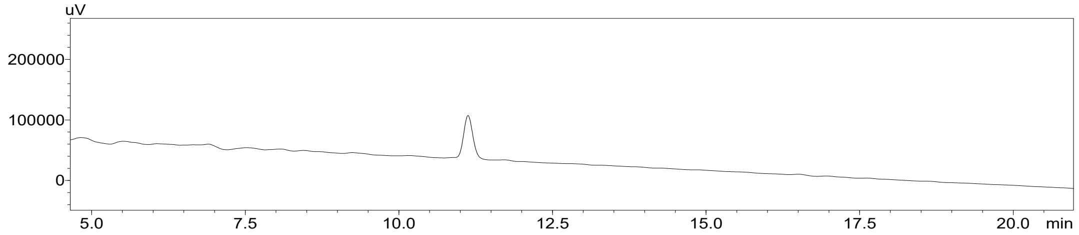

DS-LNF II (Compound **3**), Retention time = 15.9 min

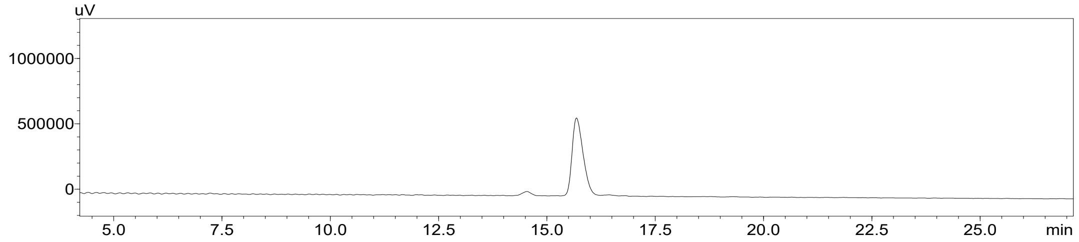

Le^C (Compound **5**), Retention time = 14.3 min

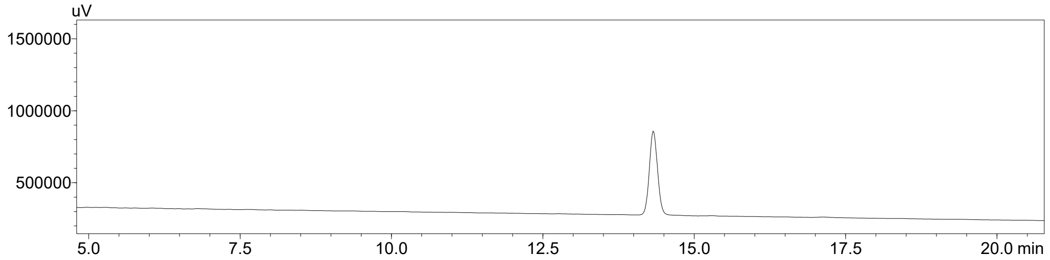

BG^{H1} (Compound **6**), Retention time = 12.9 min

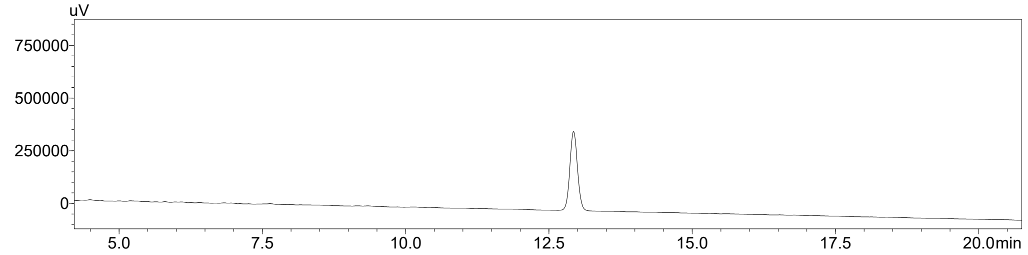

Le^A (Compound **7**), Retention time = 13.5 min

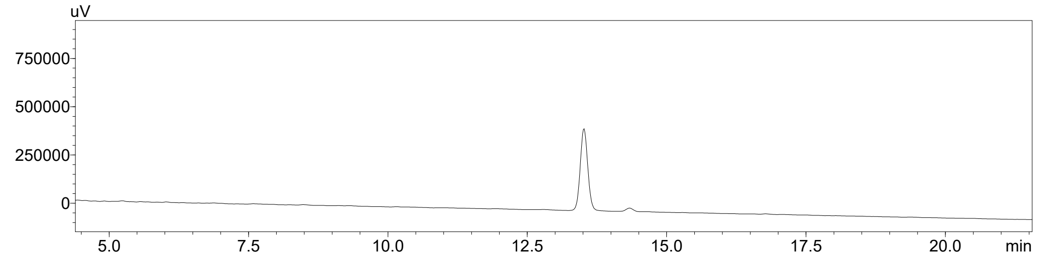

Le^{B} (Compound **8**), Retention time = 11.8 min

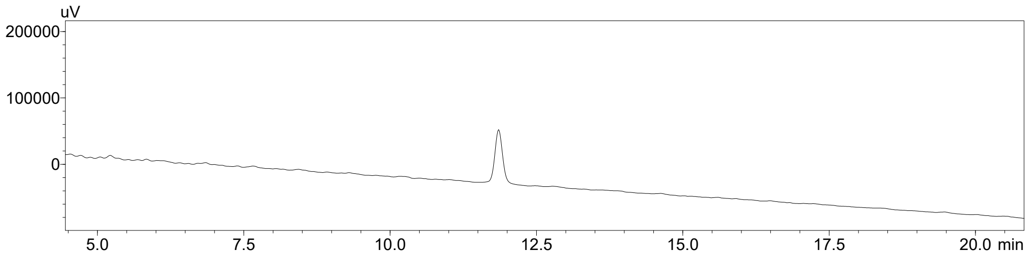

BG^{A1} (Compound **9**), Retention time = 13.1 min

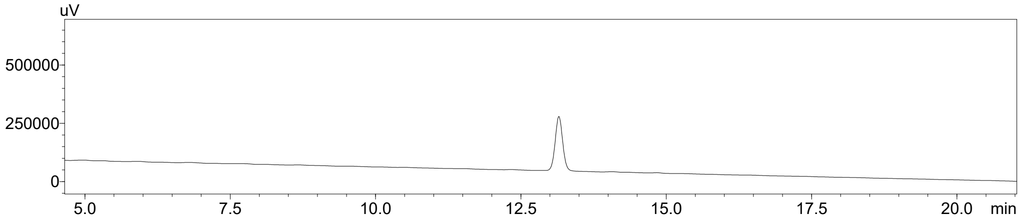

BG^{B1} (Compound **10**), Retention time = 13.0 min

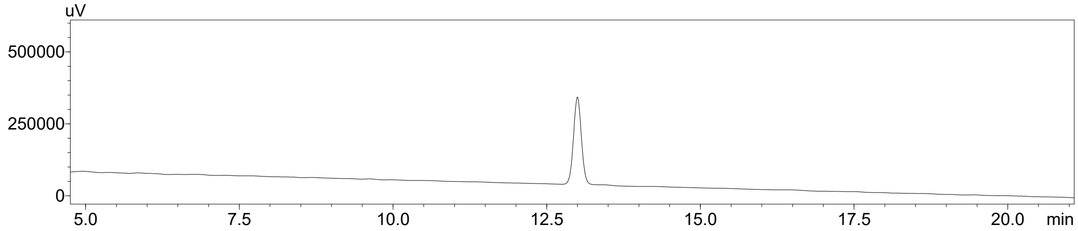

A-Le^{B} (Compound **11**), Retention time = 11.9 min

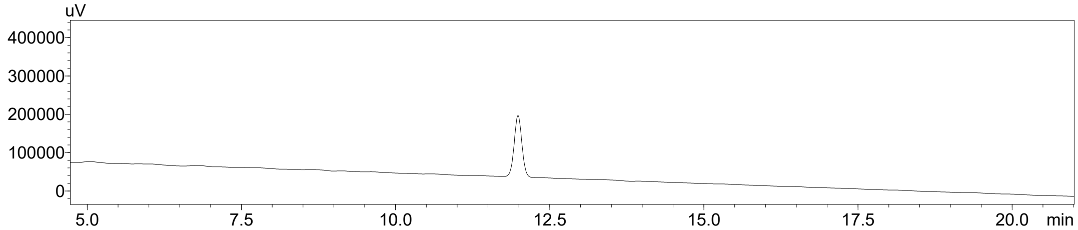

B-Le^{B} (Compound **12**), Retention time = 11.9 min

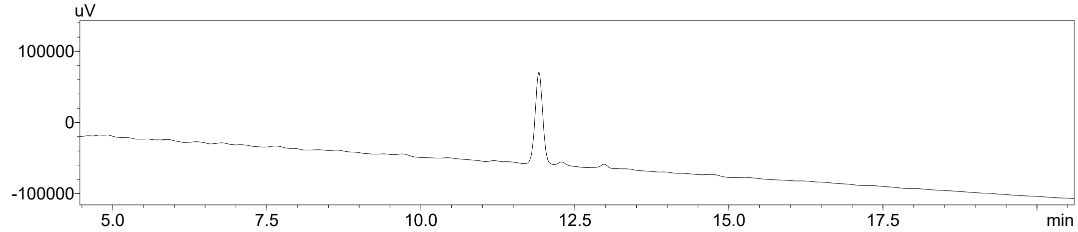

3'-sulfo Le^{C} (Compound **13**), Retention time =13.6 min

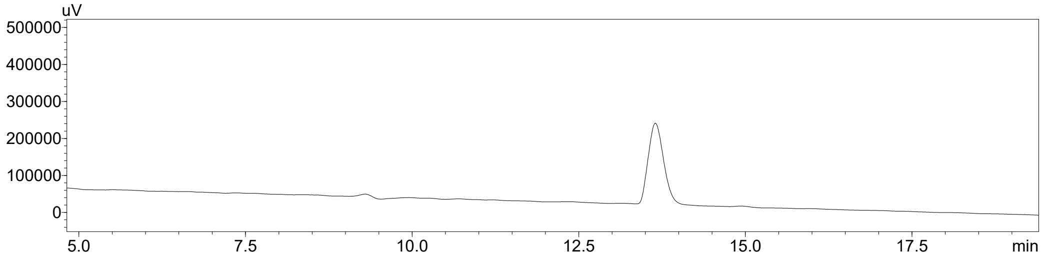

3'-sulfo Le^{A} (Compound **14**), Retention time =12.6 min

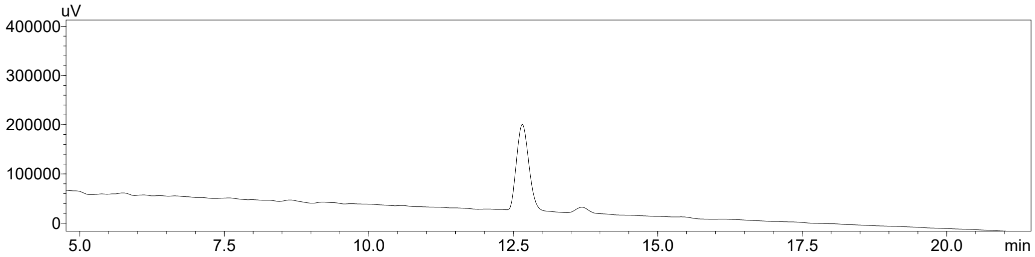

SLe^{C} (Compound **15**), Retention time = 12.8 min

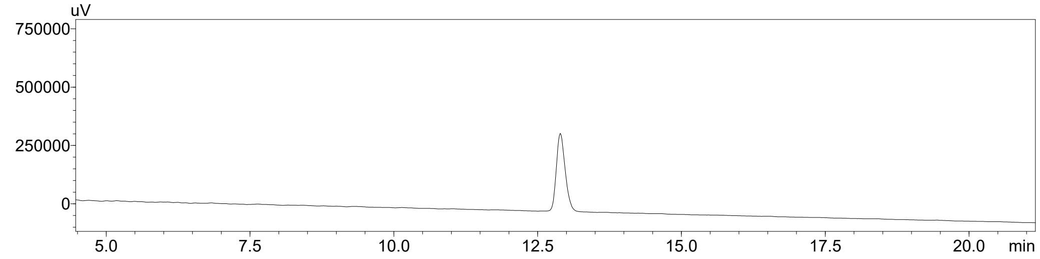

SLe^{A} (Compound **16**), Retention time = 11.7 min

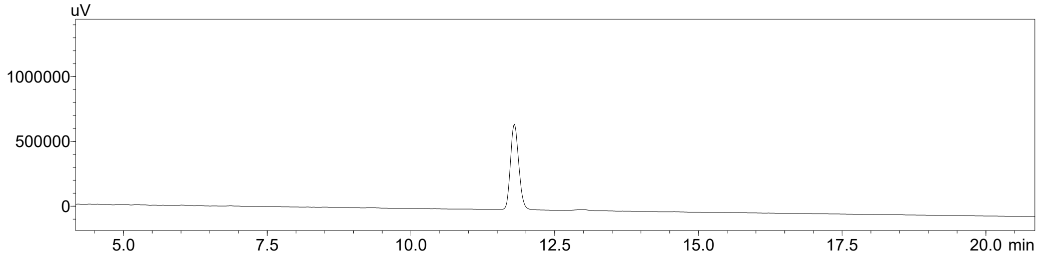

DSLe^{C} (Compound **17**), Retention time = 10.7 min

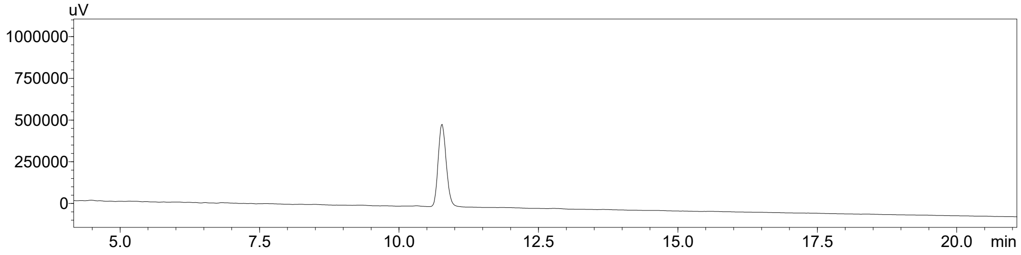

DSLe^{A} (Compound **18**), Retention time = 9.8 min

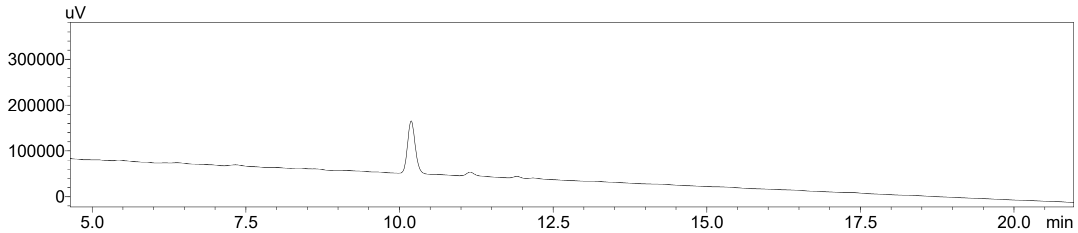

GLe^{C} (Compound **19**), Retention time = 12.4 min

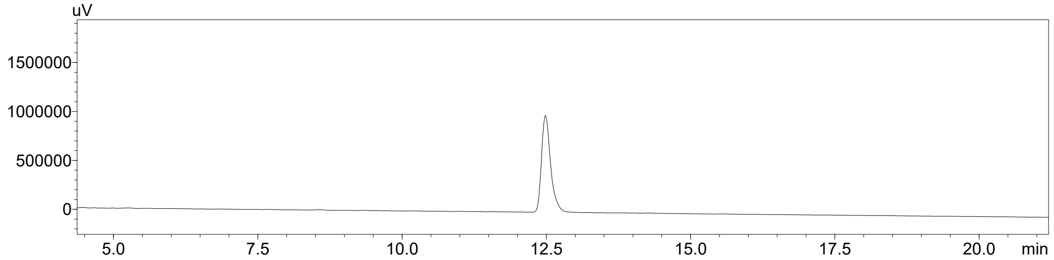

GLe^{A} (Compound **20**), Retention time = 11.3 min

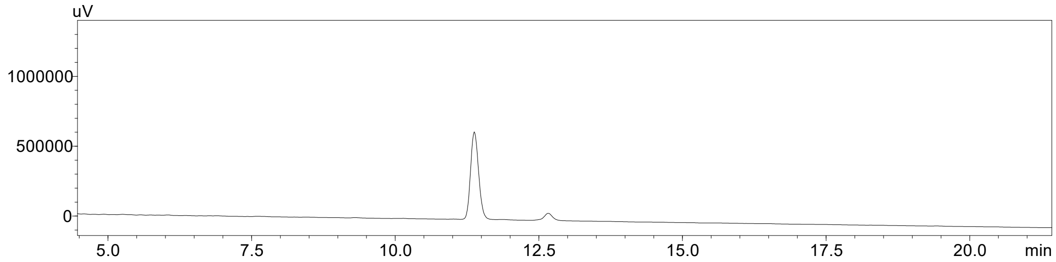

DGLe^{C} (Compound **21**), Retention time = 9.9 min

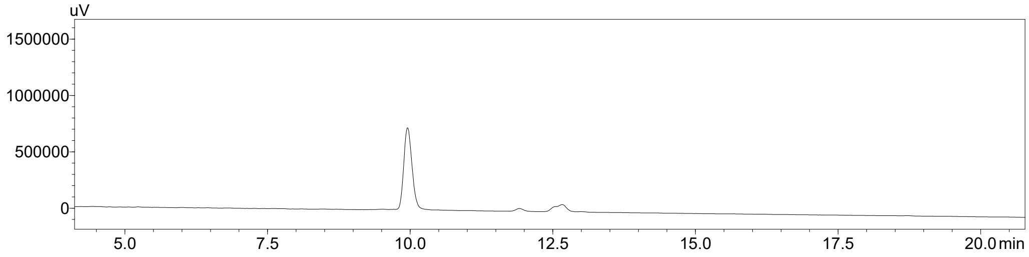

DGLe^{A} (Compound **22**), Retention time = 8.9 min

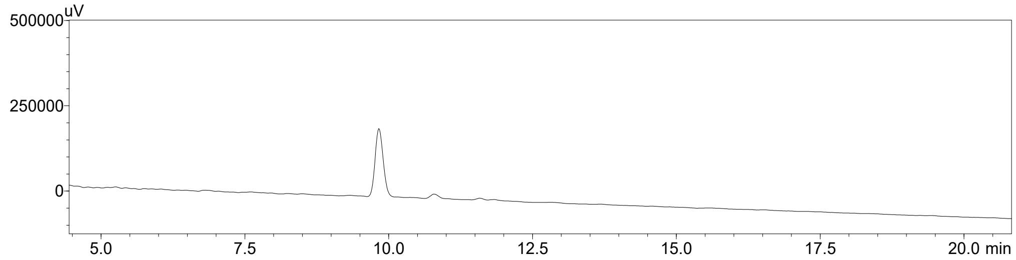

6SLe^{C} (Compound **23**), Retention time = 12.2 min

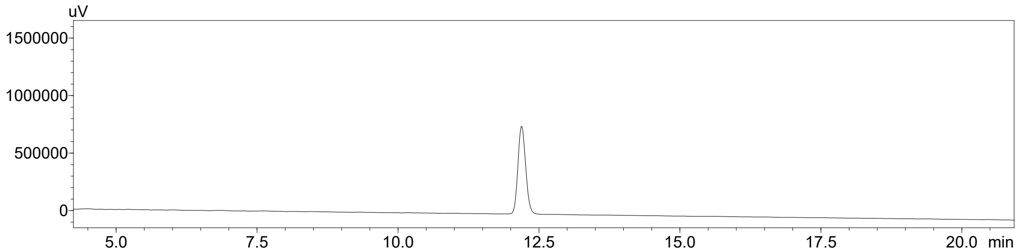

6SBG^{H1} (Compound **24**), Retention time = 10.7 min

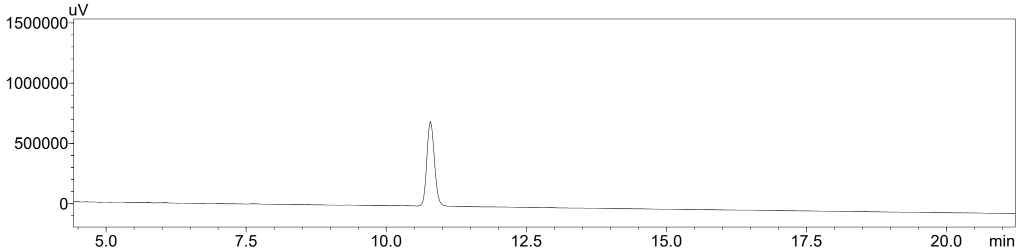

6SLe^{B} (Compound **25**), Retention time = 9.8 min

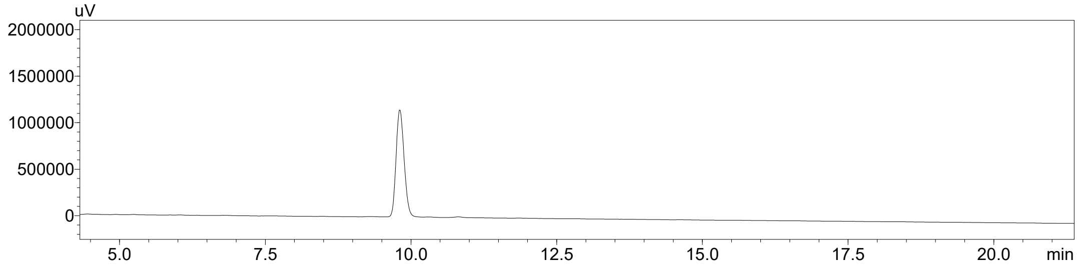

6SLe^{A} (Compound **26**), Retention time = 11.5 min

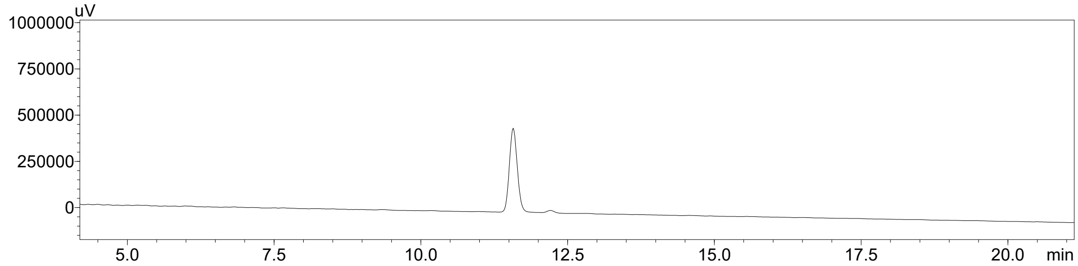

Compound **28**, Retention time = 13.3 min

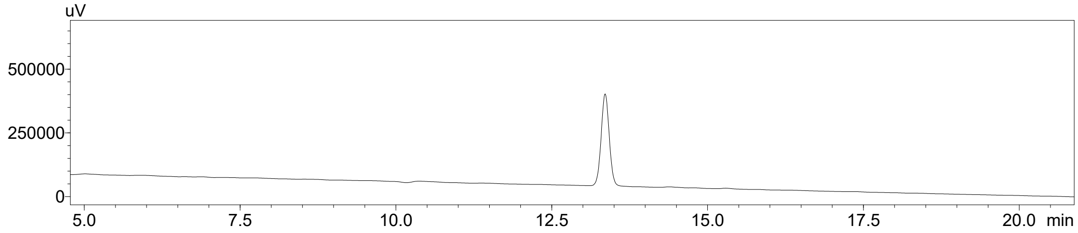

Le^{C}-Le^{C} (Compound **29**), Retention time = 13.6 min

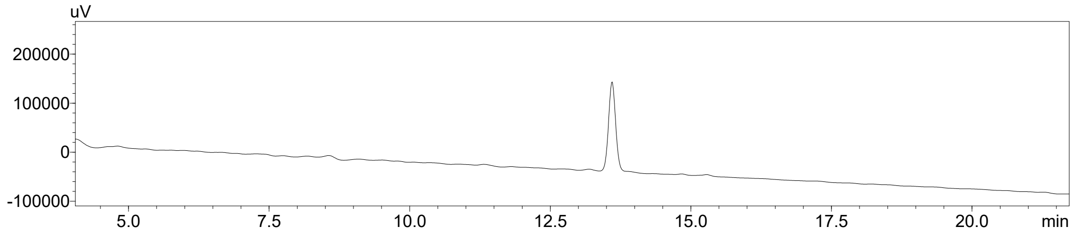

BG^{H1}-Le^{C} (Compound **30**), Retention time = 13.2 min

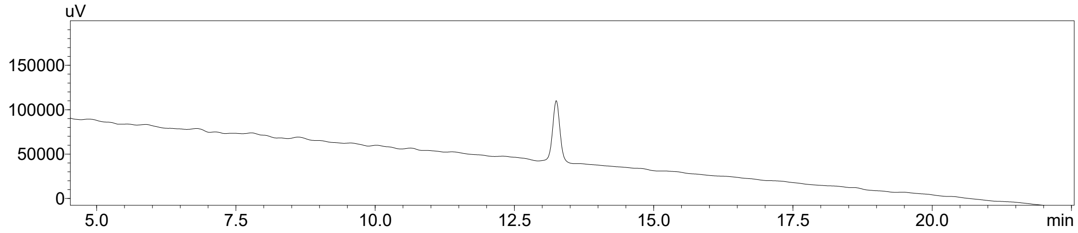

Le^{A}-Le^{A} (Compound **31**), Retention time = 12.1 min

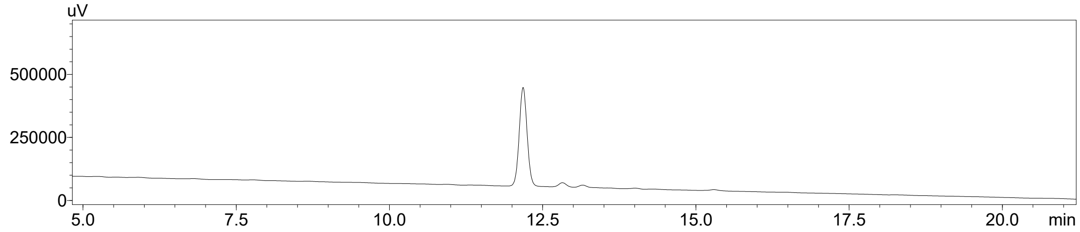

Le^{B}-Le^{A} (Compound **32**), Retention time = 11.9 min

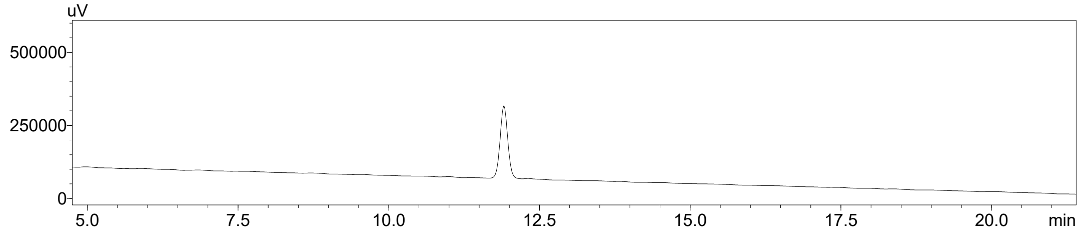

Le^{C}-BG^{H1} (Compound **33**), Retention time = 12.8 min

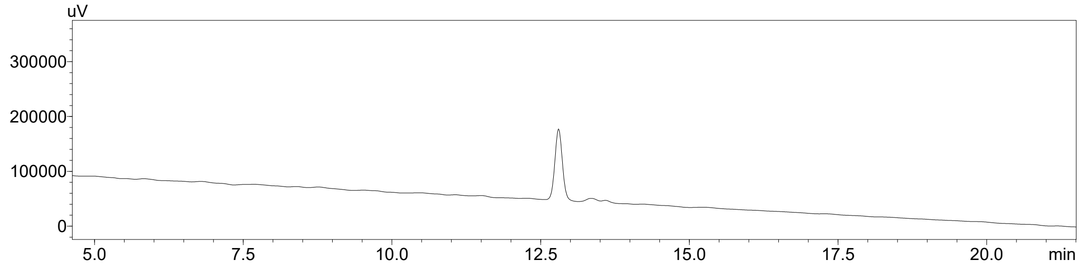

BG^{H1}-BG^{H1} (Compound **34**), Retention time = 12.6 min

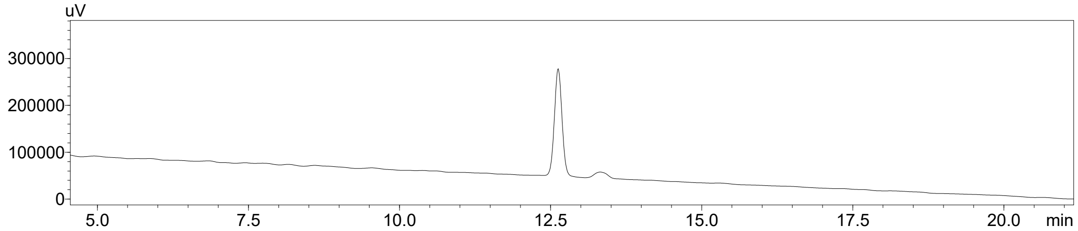

Le^{A}-BG^{H1} (Compound **35**), Retention time = 12.1 min

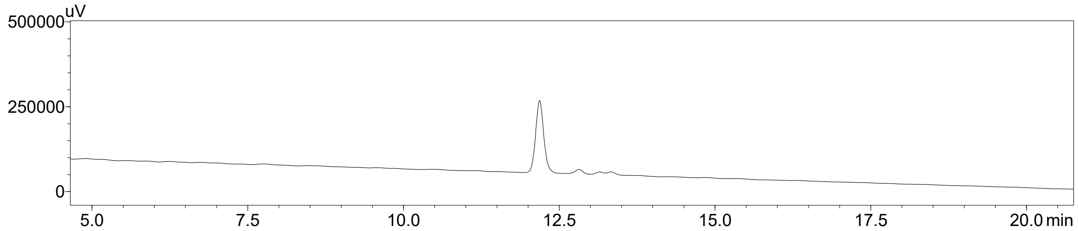

Le^{B}-BG^{H1} (Compound **36**), Retention time = 11.8 min

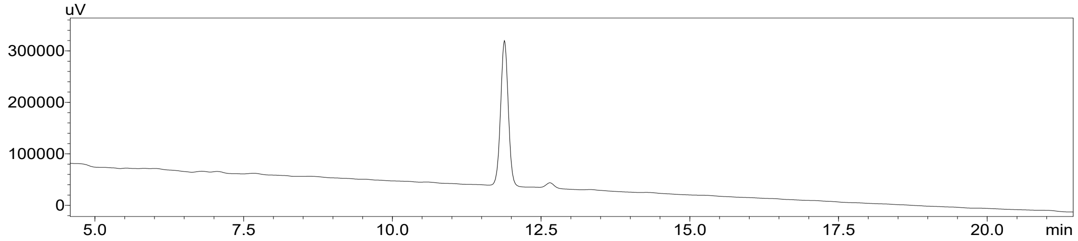

LN, Retention time = 14.7 min

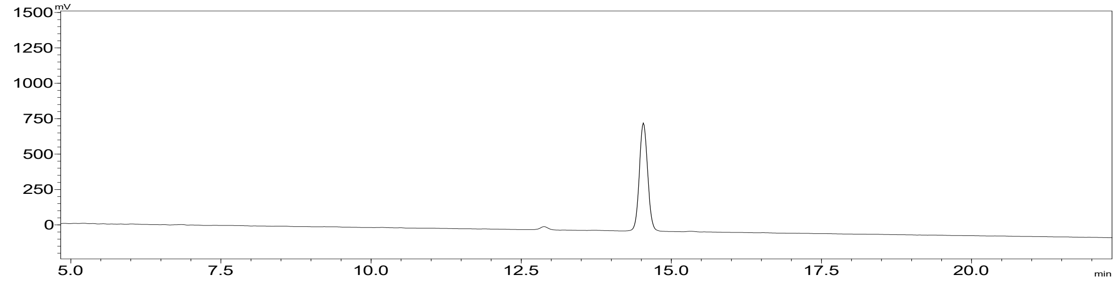

SLe^{X}, Retention time = 12.5 min

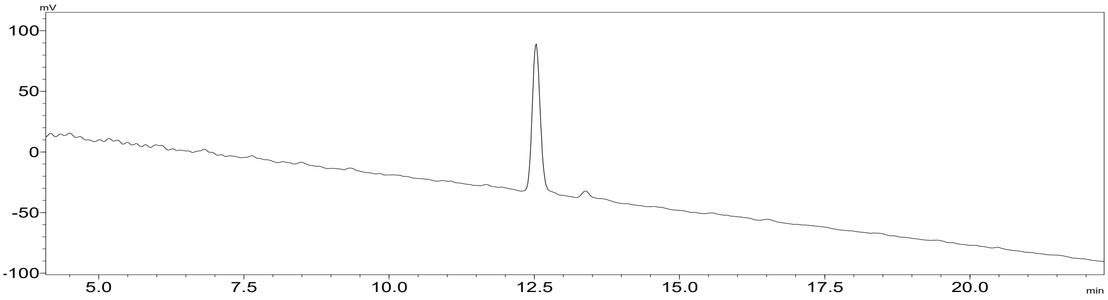

VI. References

1. Grishin, A. V.; Krivozubov, M. S.; Karyagina, A. S.; Gintsburg, A. L., Pseudomonas Aeruginosa Lectins As Targets for Novel Antibacterials. *Acta Naturae* **2015,** *7* (2), 29-41.
2. Bojar, D.; Meche, L.; Meng, G.; Eng, W.; Smith, D. F.; Cummings, R. D.; Mahal, L. K., A Useful Guide to Lectin Binding: Machine-Learning Directed Annotation of 57 Unique Lectin Specificities. *ACS Chem Biol* **2022,** *17* (11), 2993-3012.

VII. NMR Spectra of Synthesized Compounds

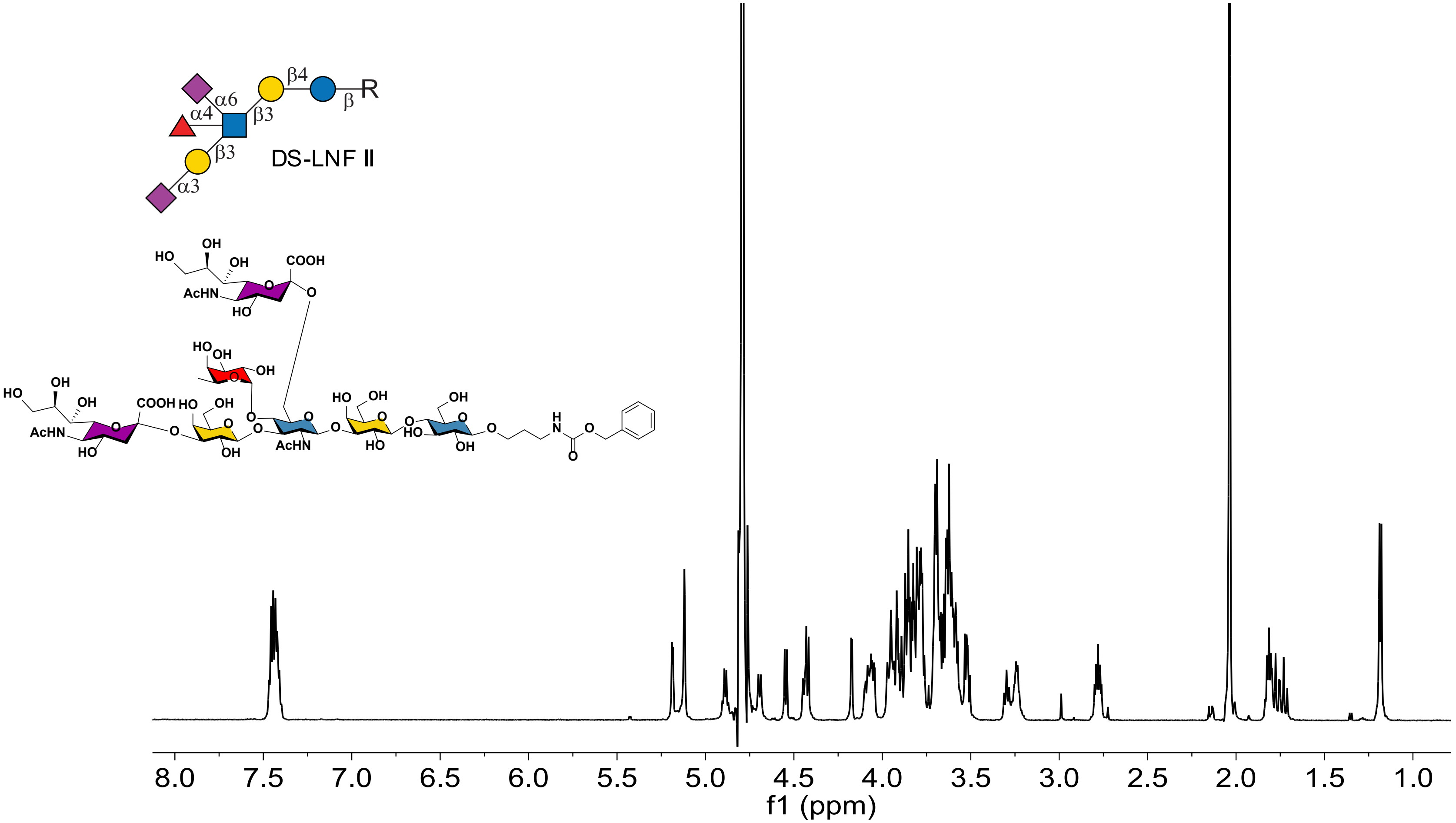

^{1}H NMR of Compound **3**

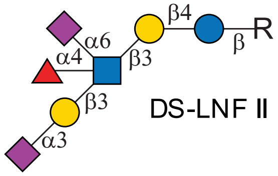

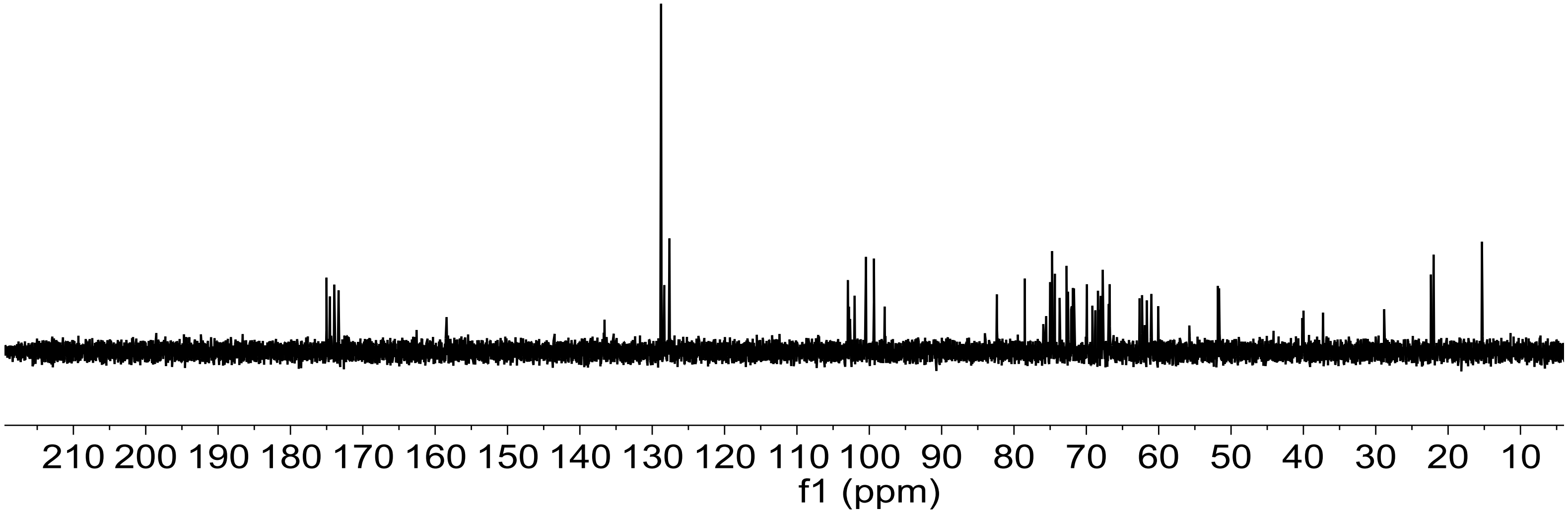

^{13}C NMR of Compound **3**

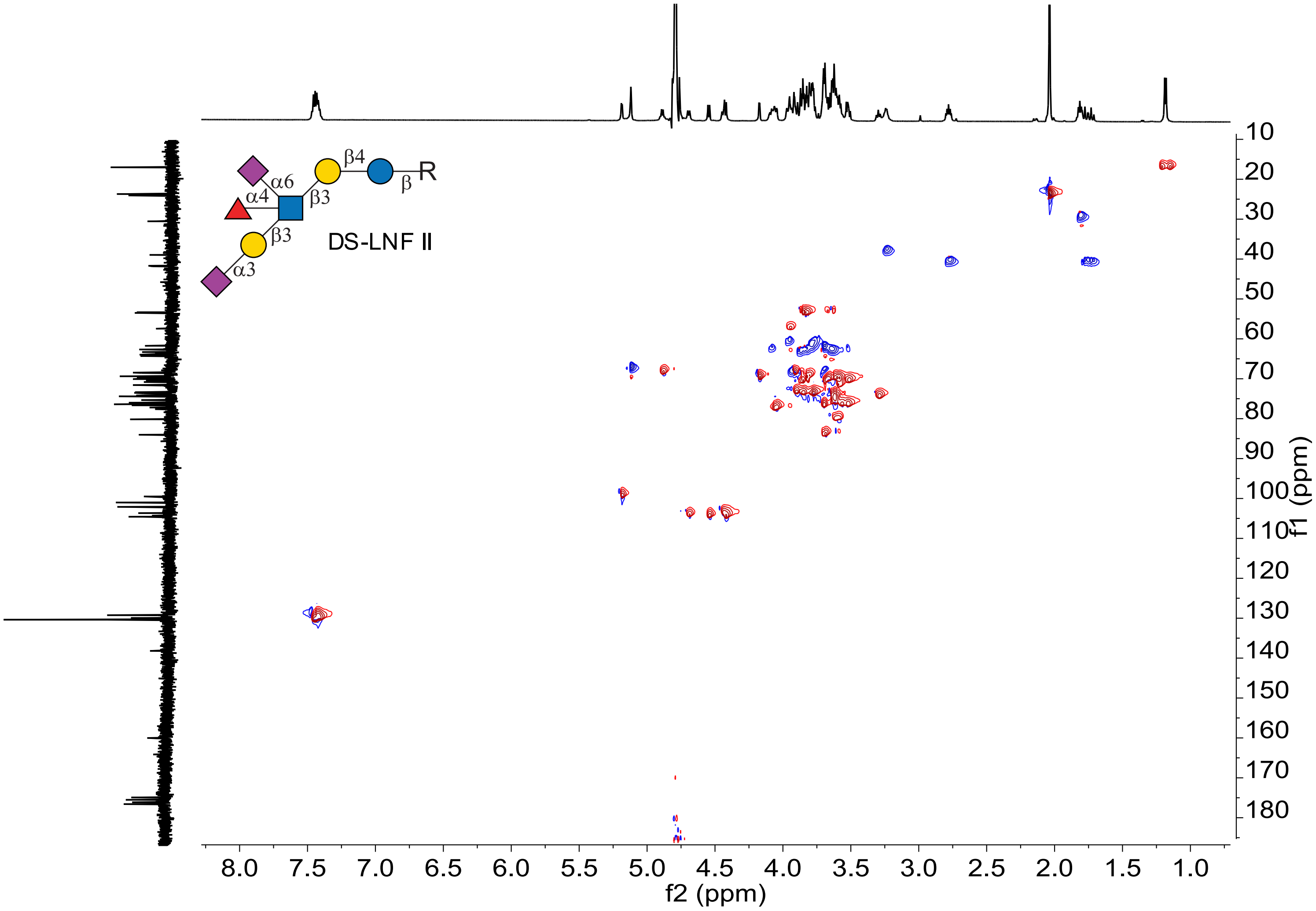

HSQC of Compound **3**

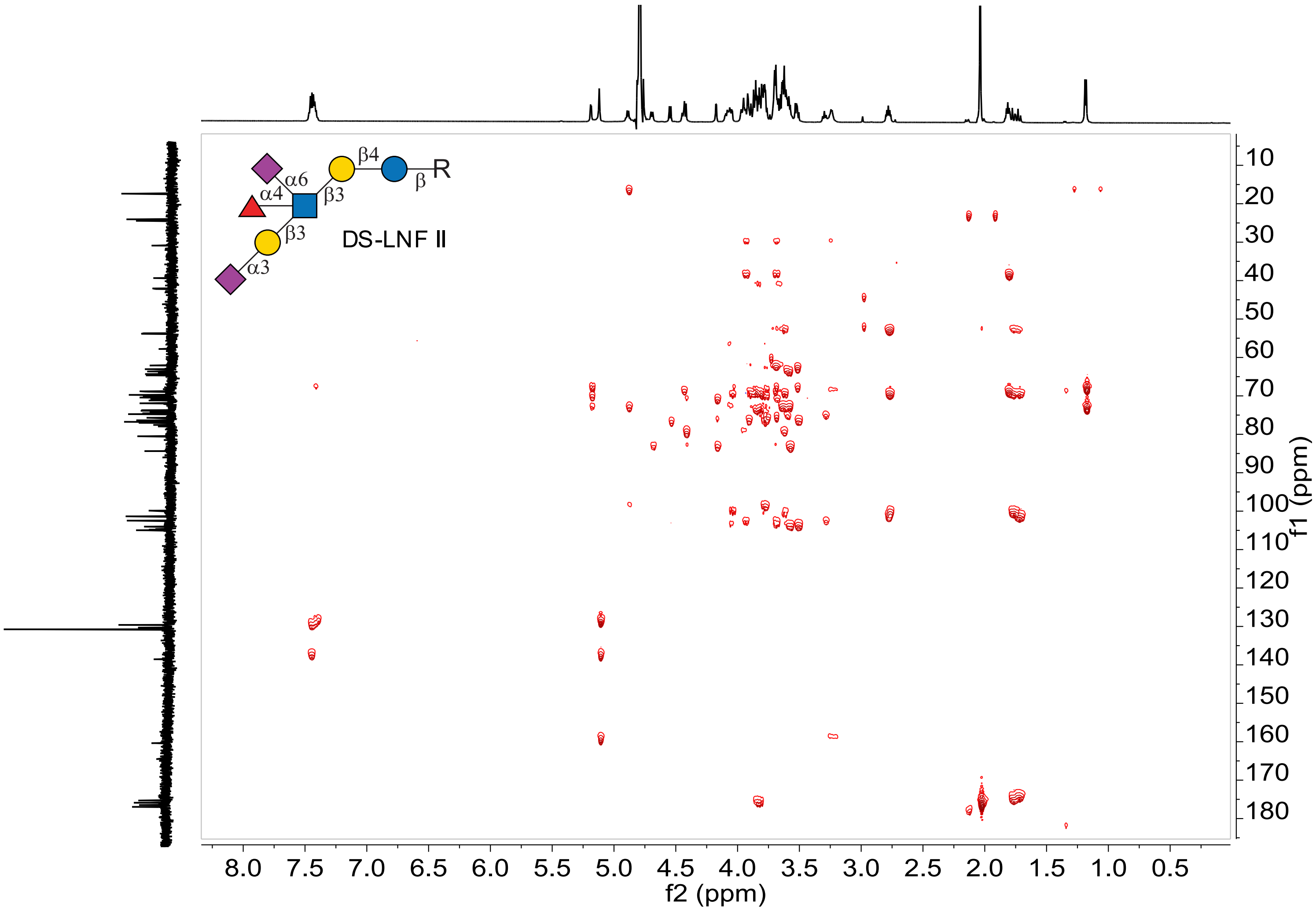

HMBC of Compound **3**

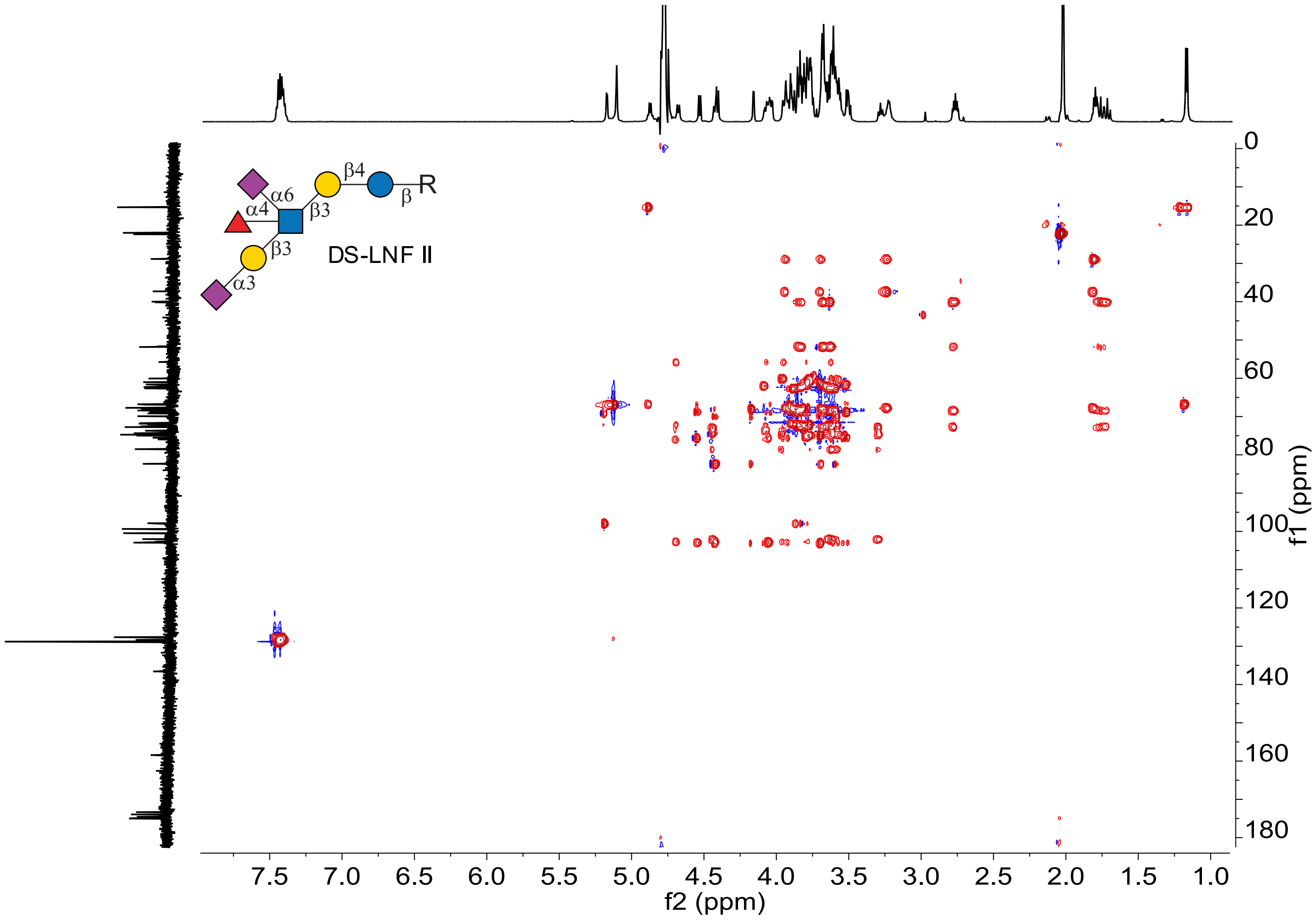

HSQC-TOCSY of Compound **3**

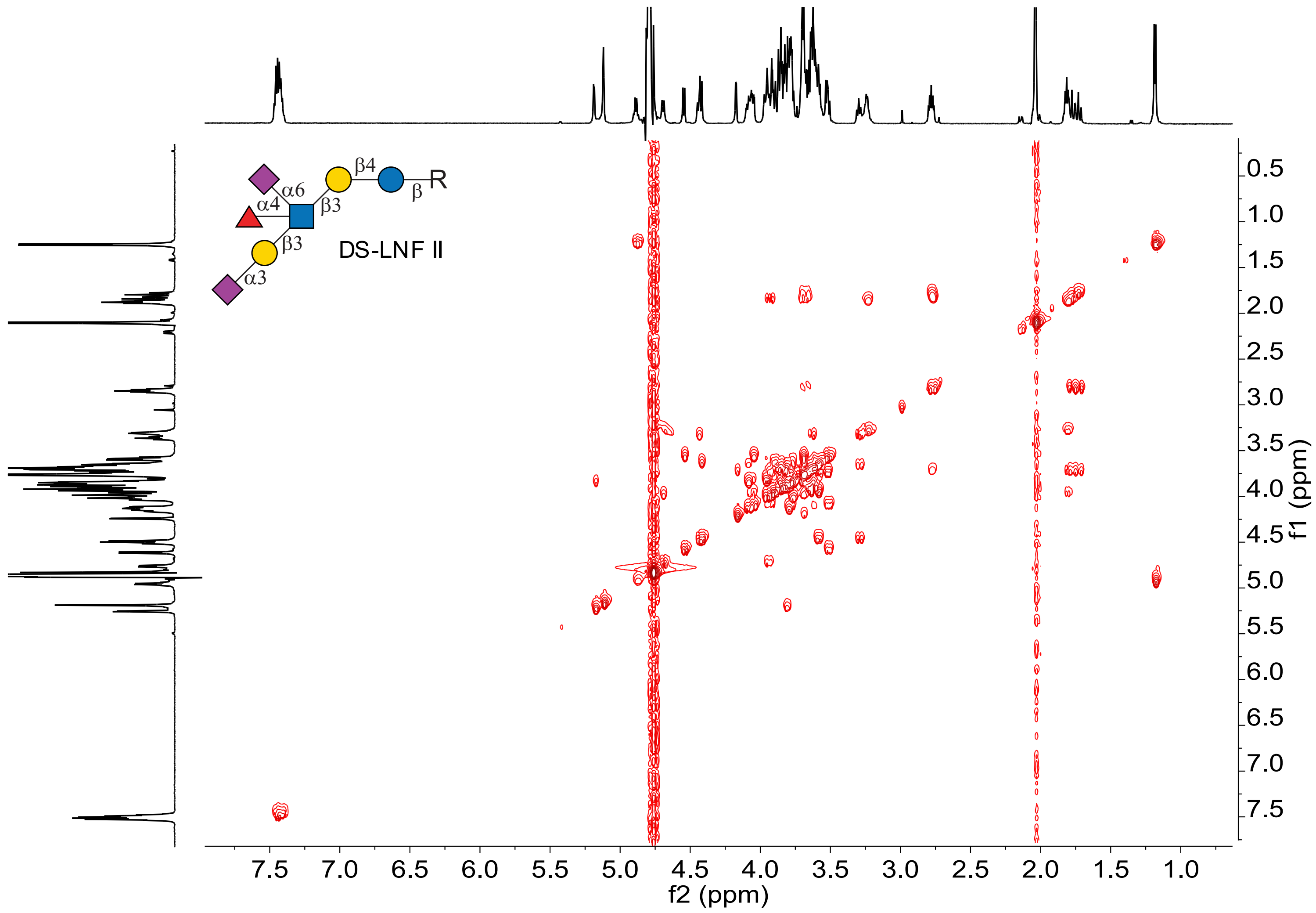

^{1}H-^{1}H COSY of Compound **3**

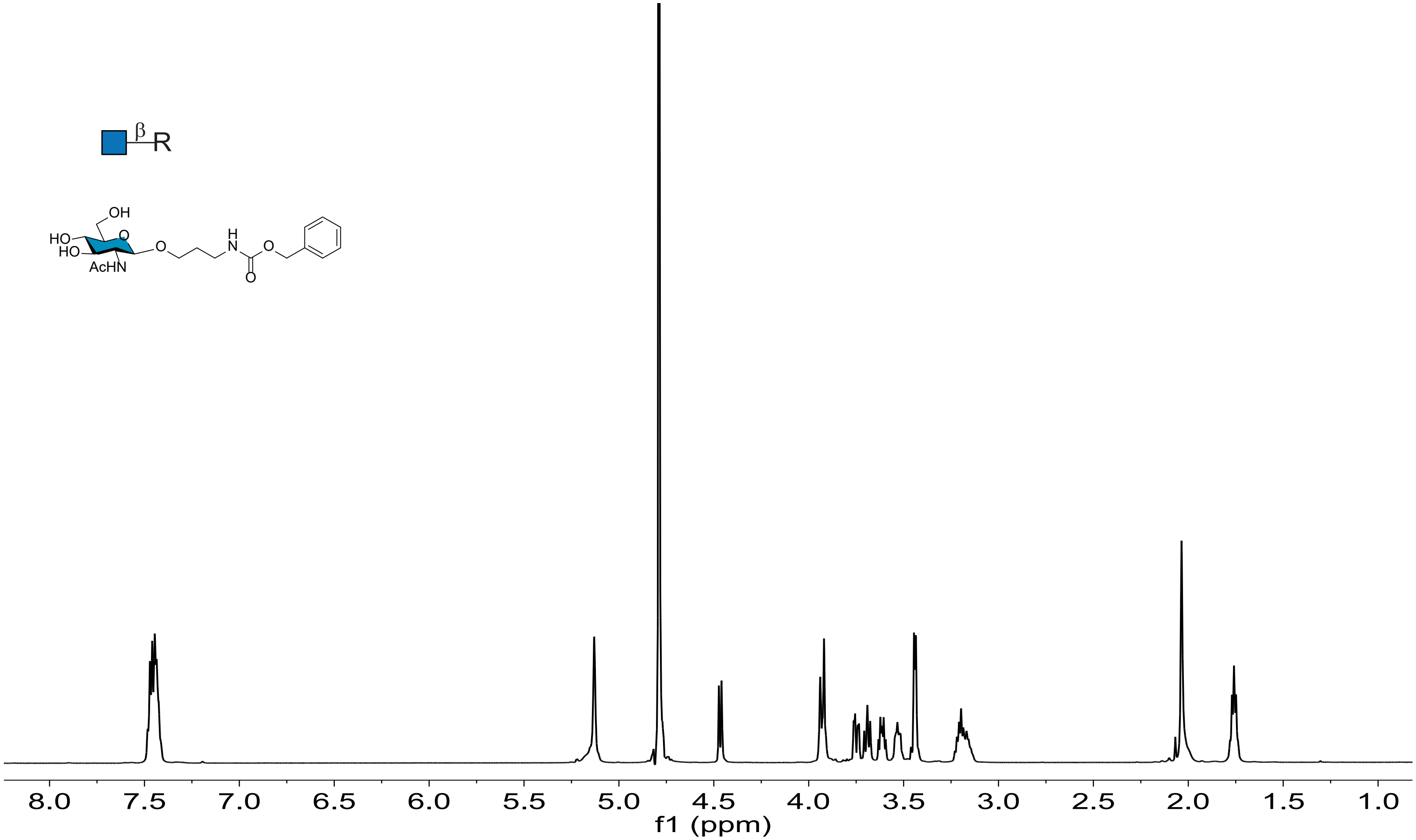

^{1}H NMR of Compound **4**

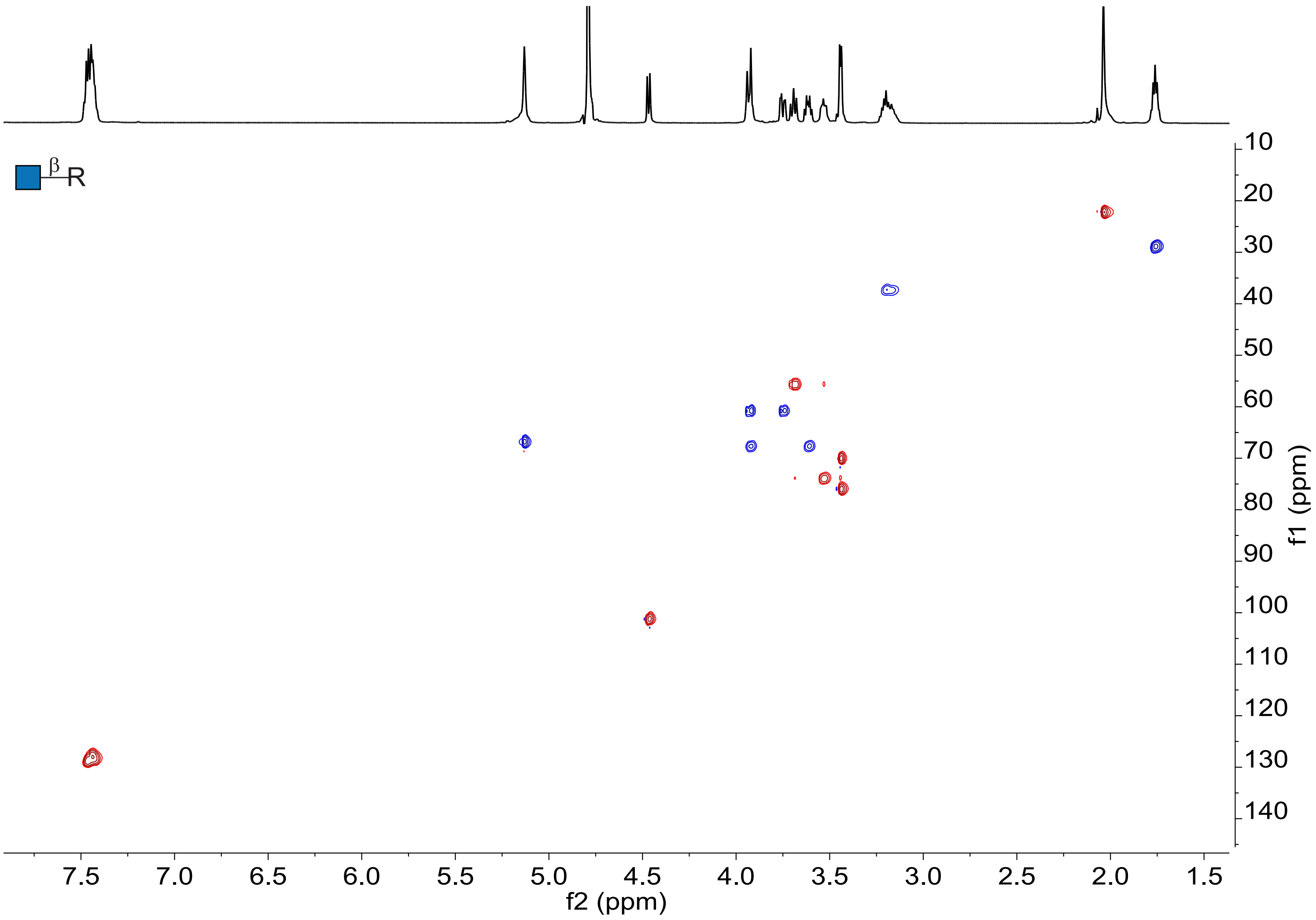

HSQC of Compound **4**

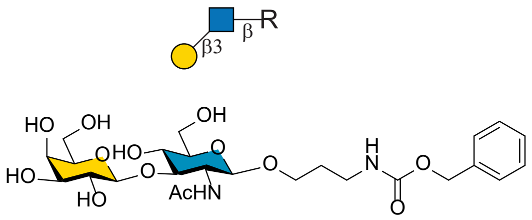

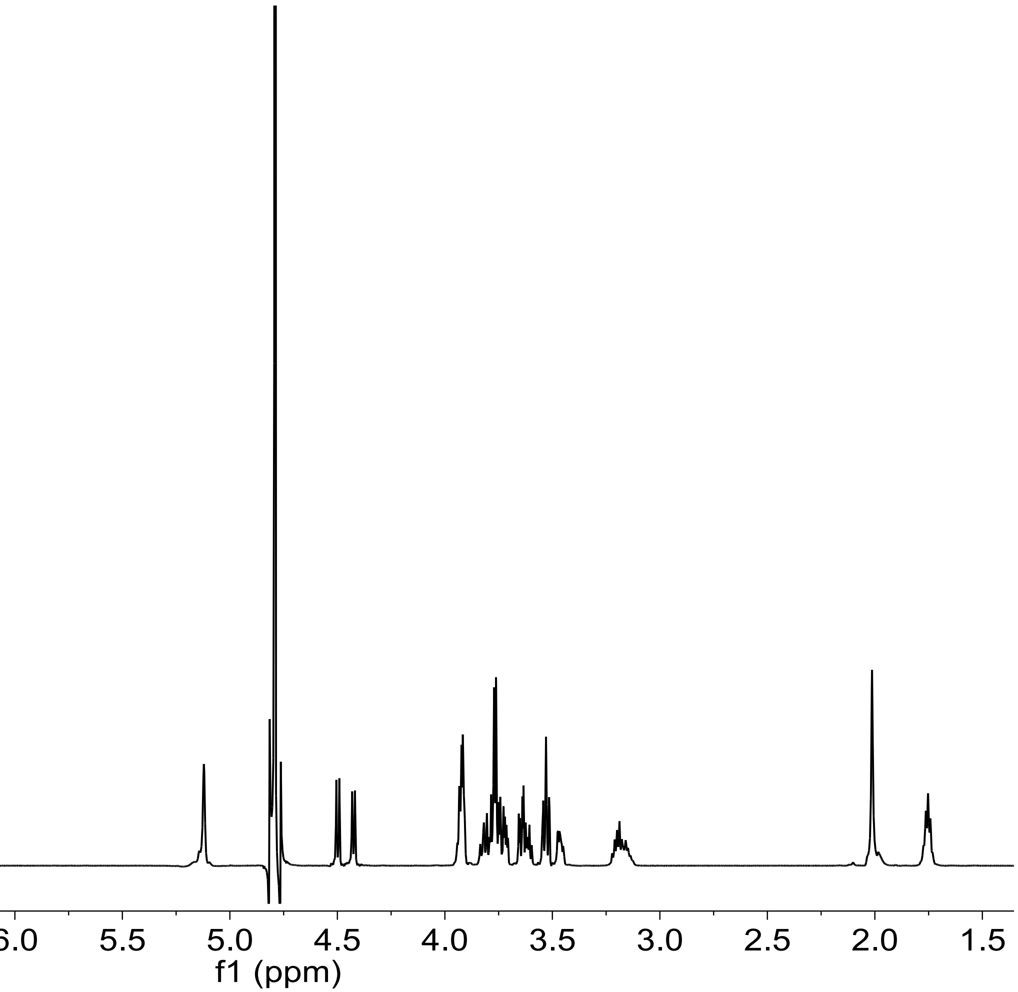

^{1}H NMR of Compound **5**

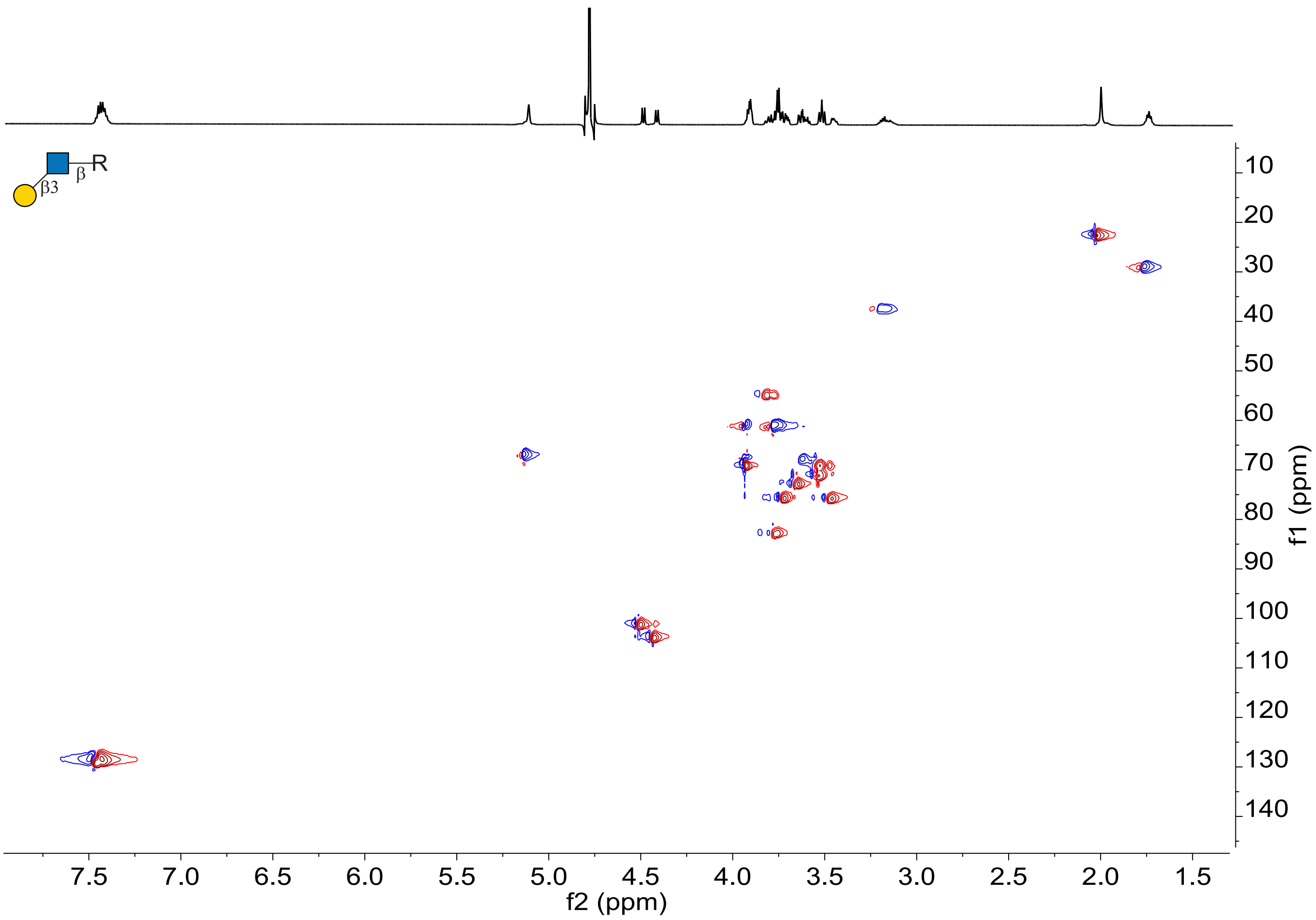

HSQC of Compound **5**

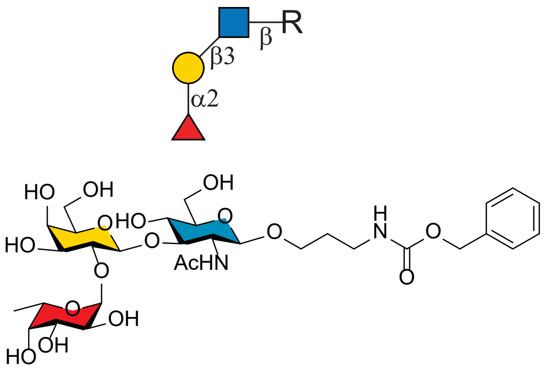

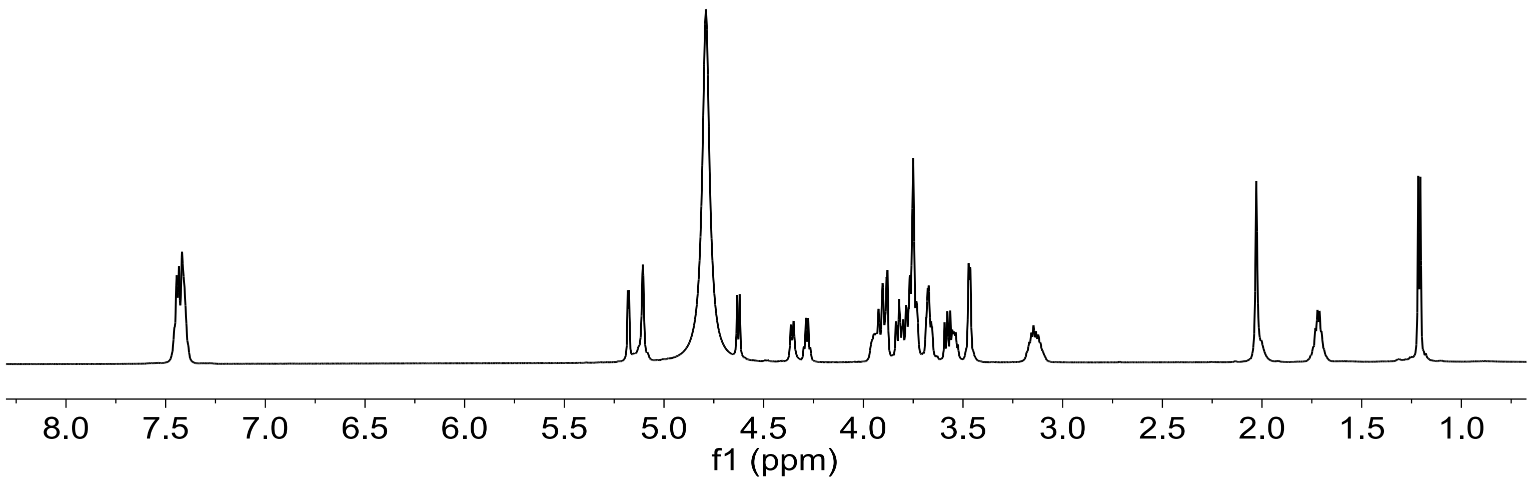

^{1}H NMR of Compound **6**

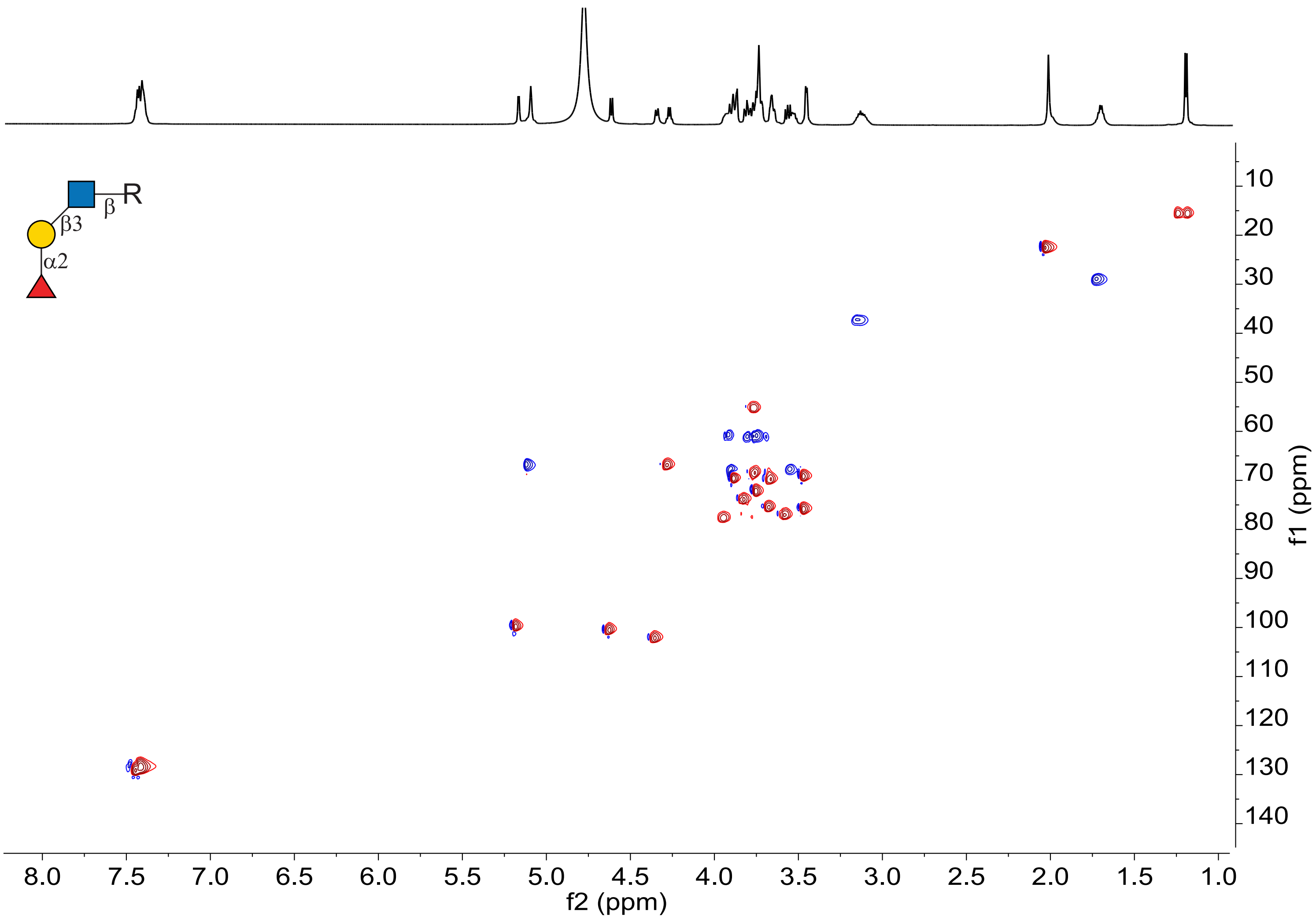

HSQC of Compound **6**

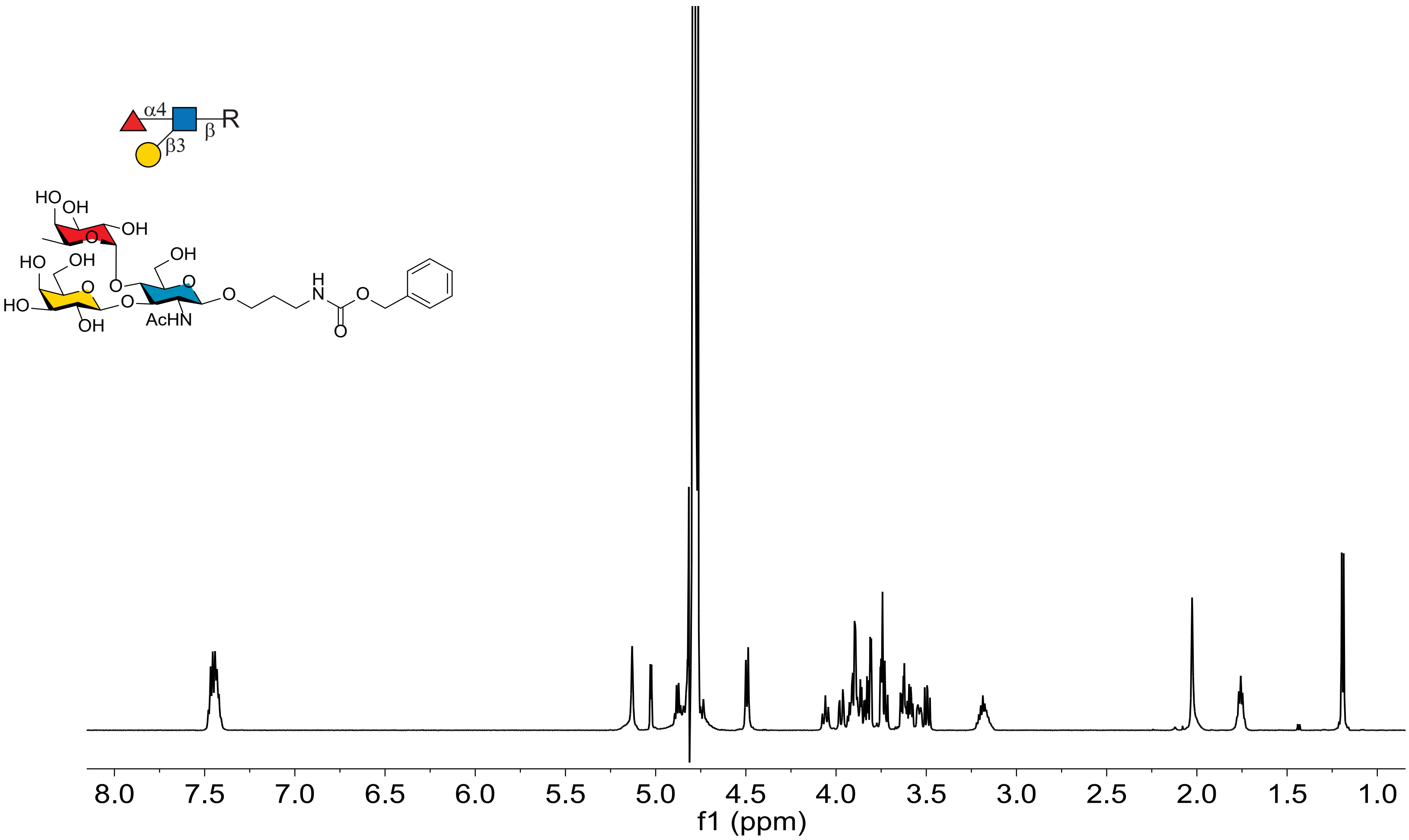

^{1}H NMR of Compound **7**

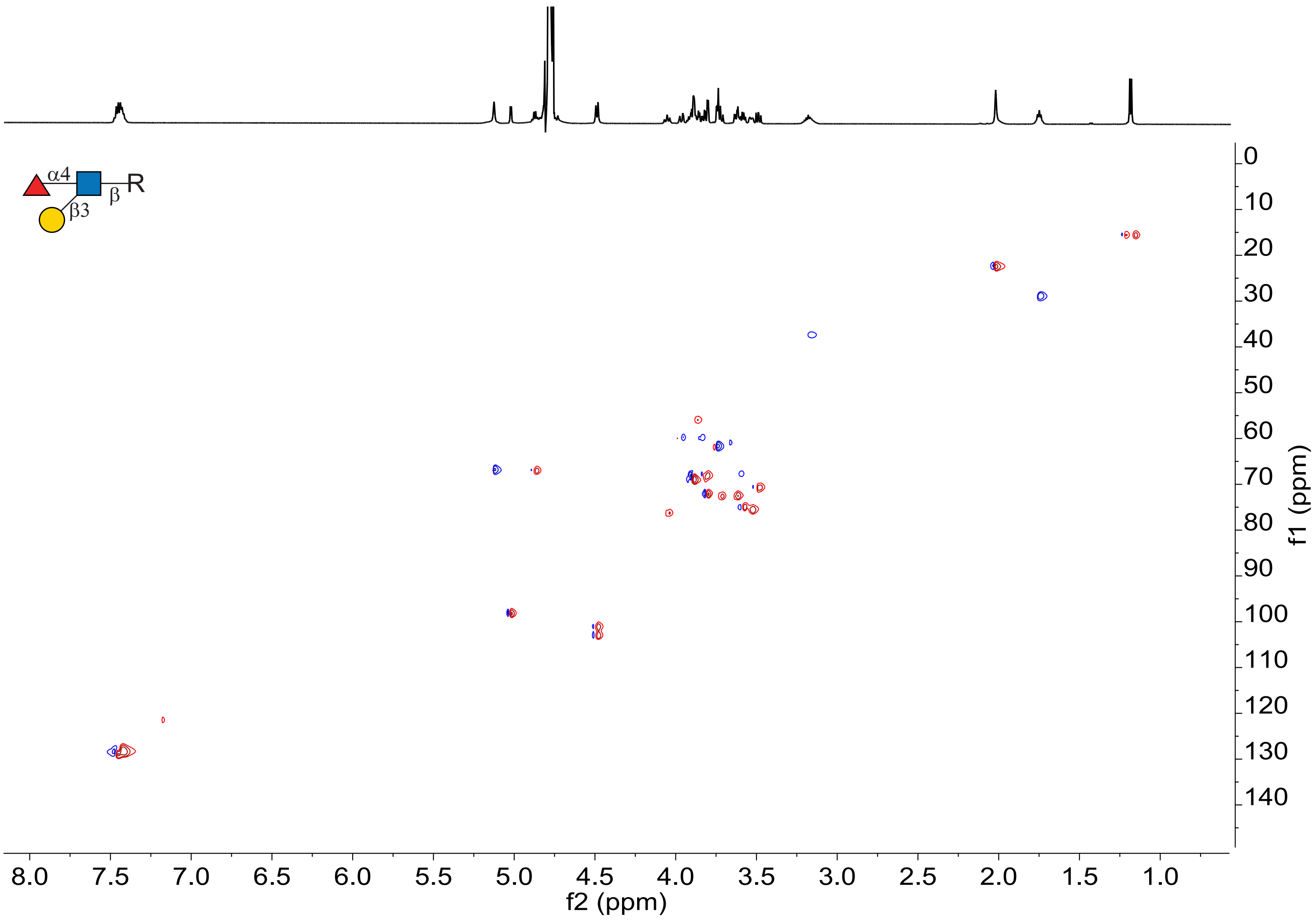

HSQC of Compound **7**

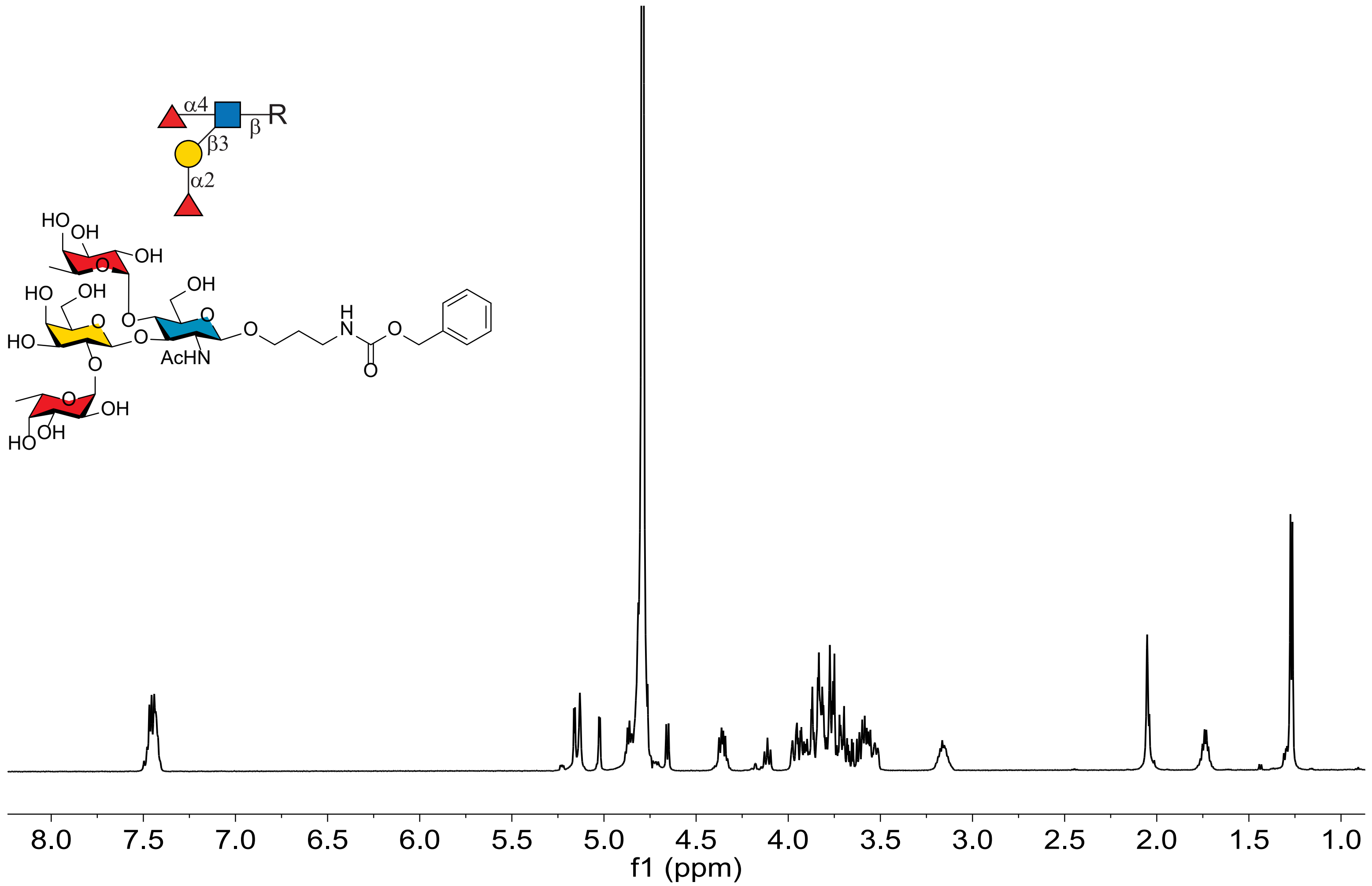

^{1}H NMR of Compound **8**

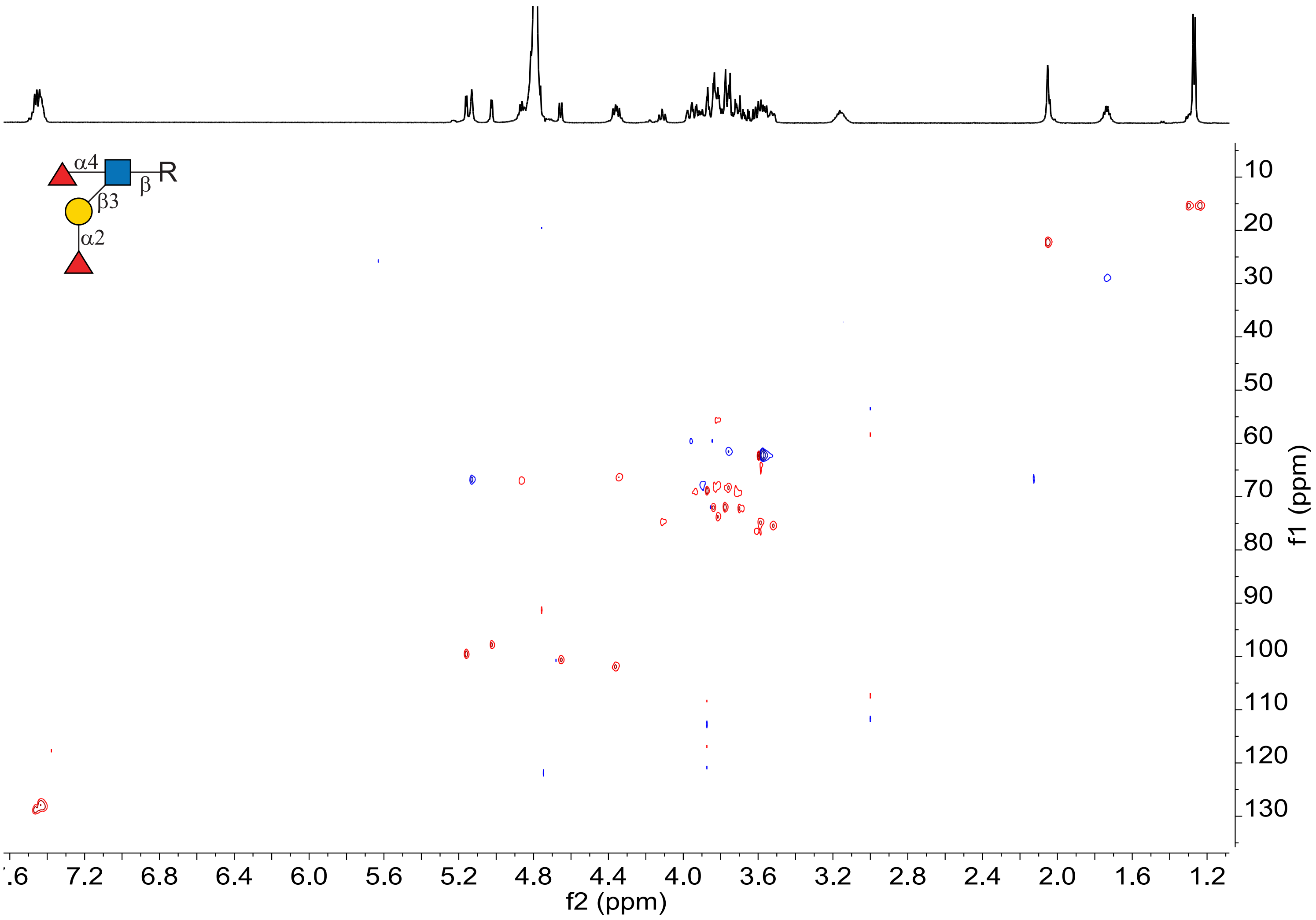

HSQC of Compound **8**

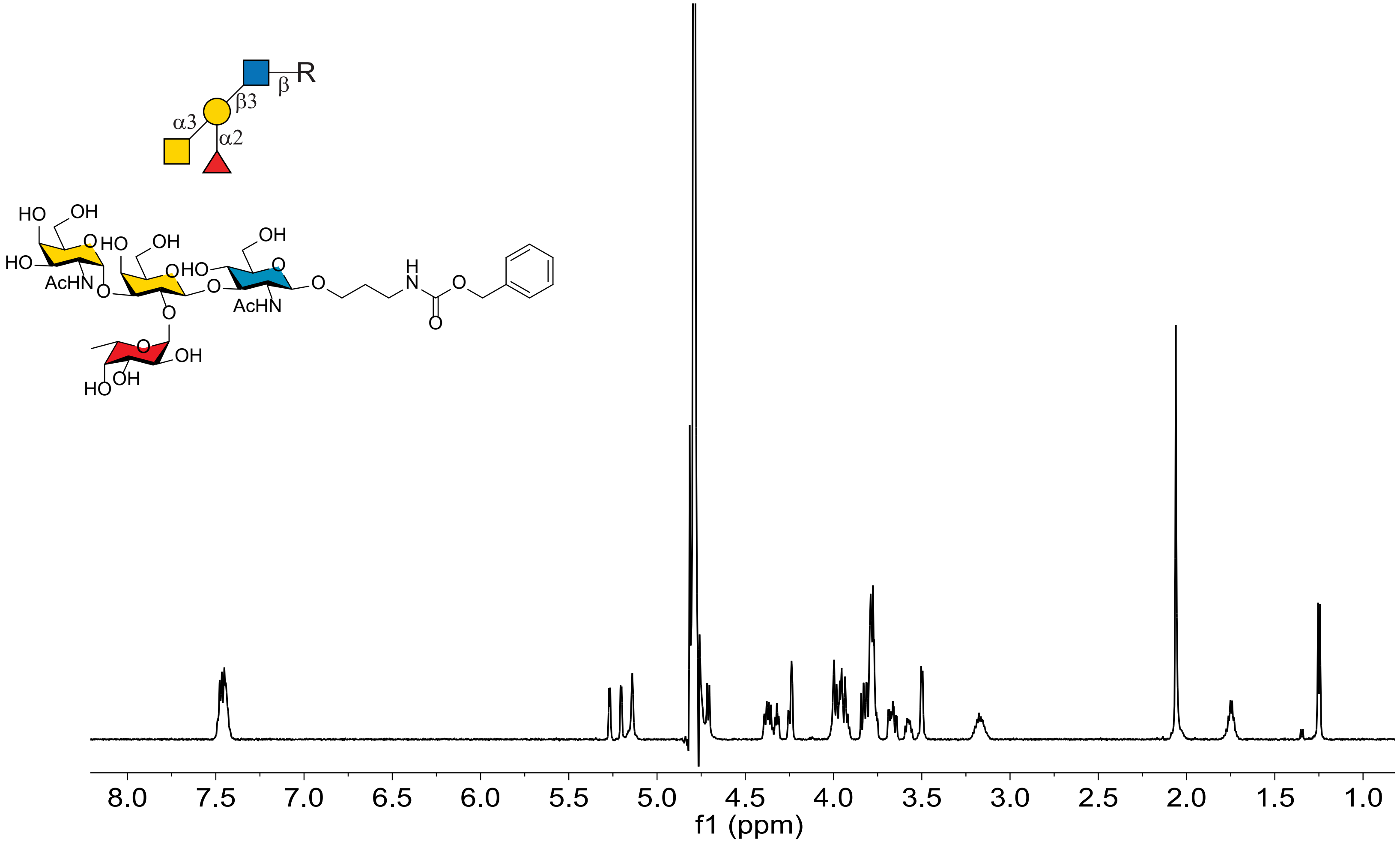

^{1}H NMR of Compound **9**

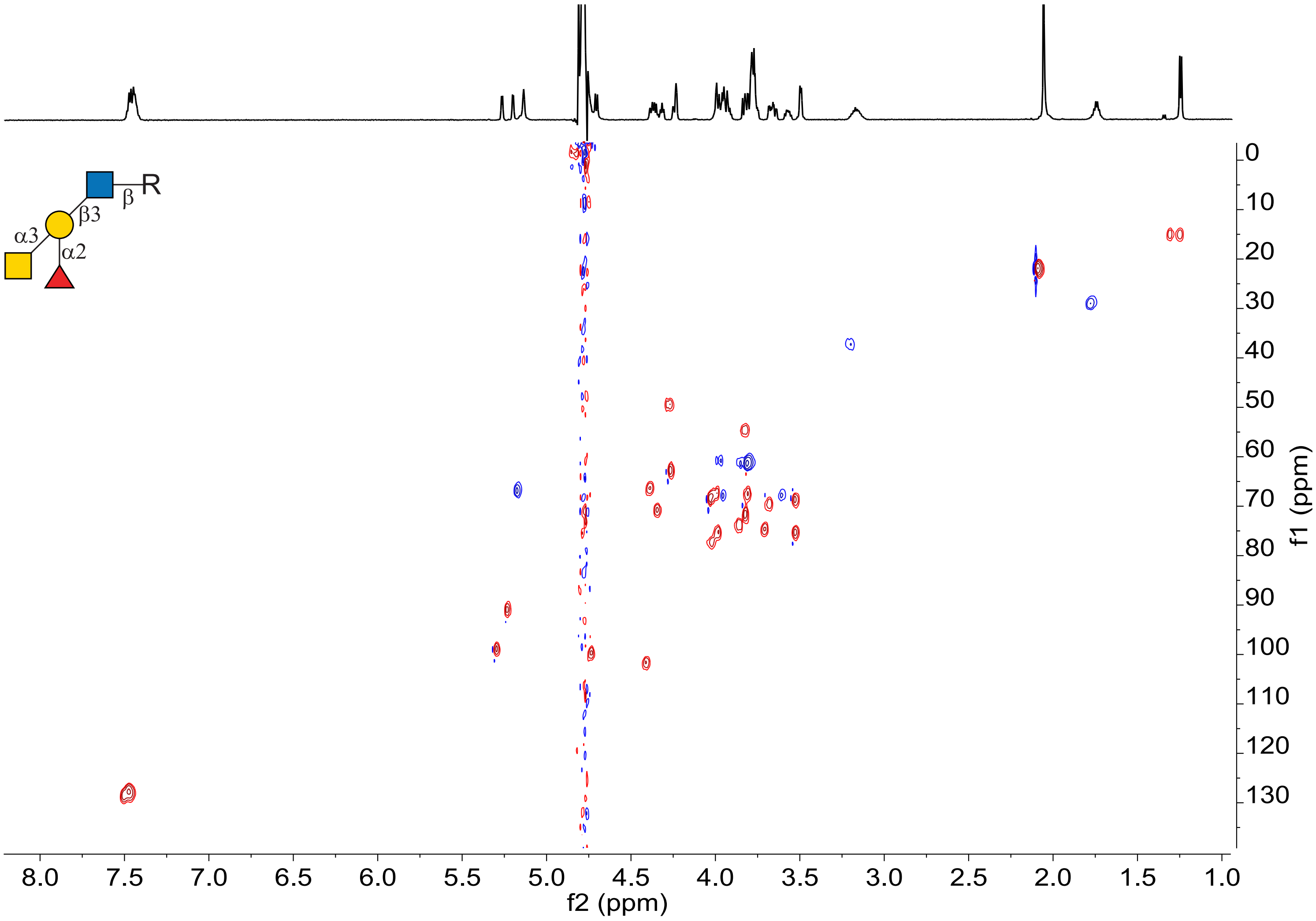

HSQC of Compound **9**

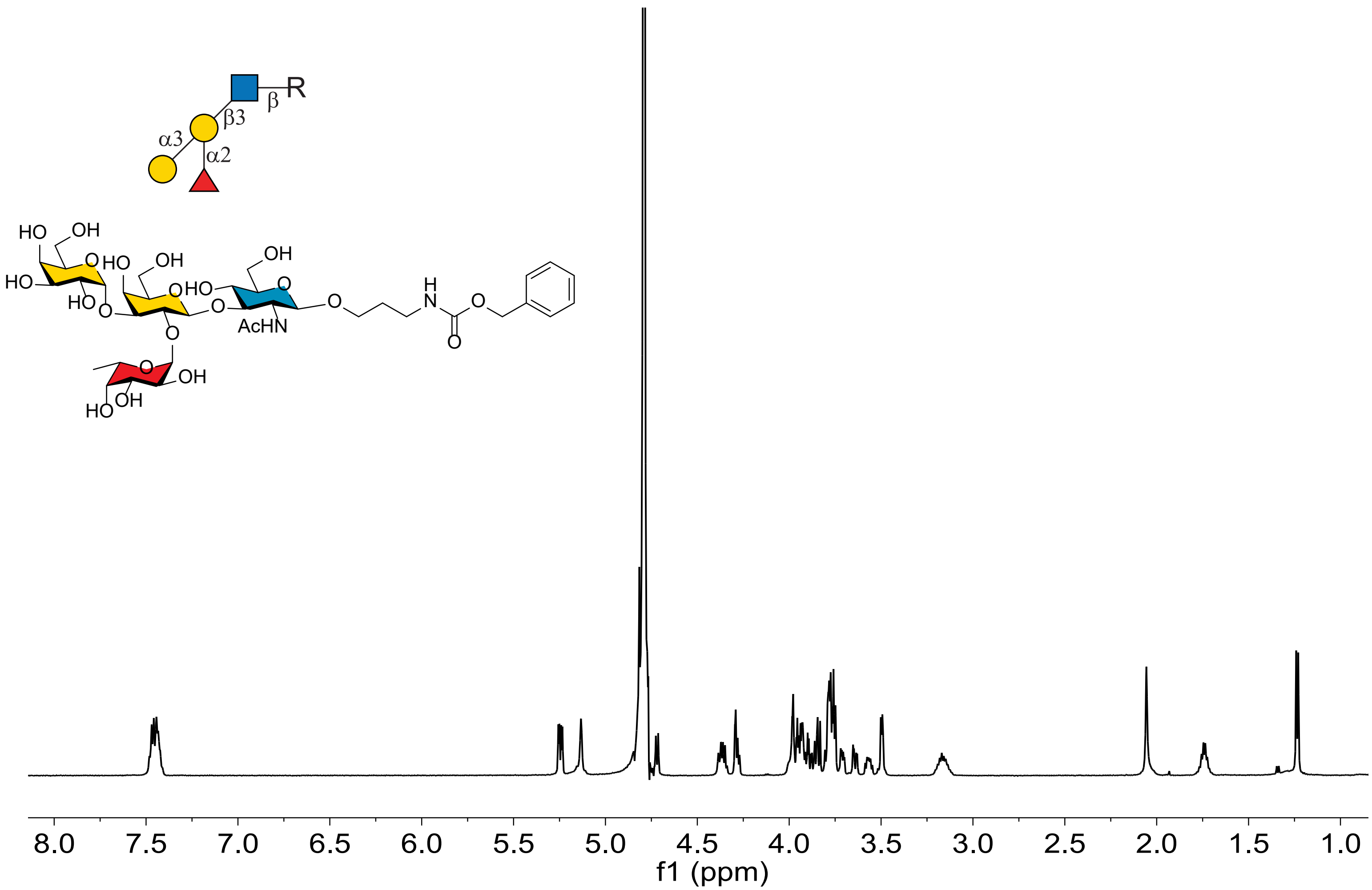

^{1}H NMR of Compound **10**

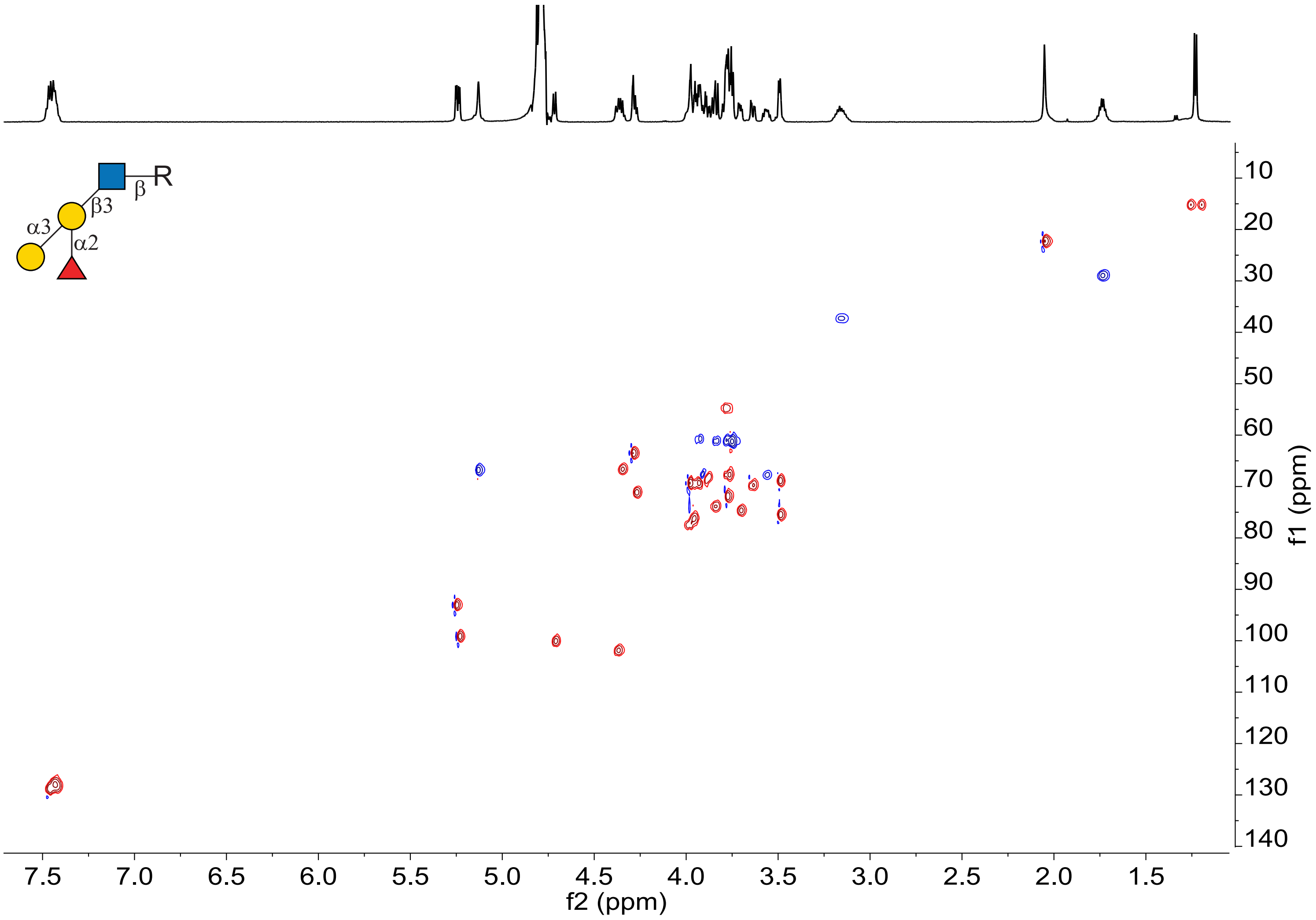

HSQC of Compound **10**

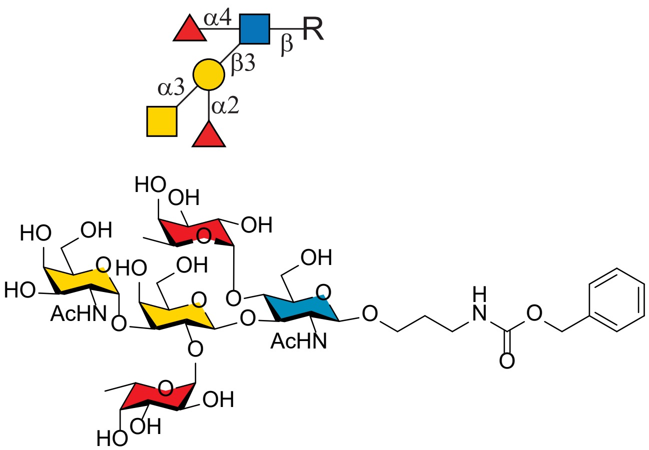

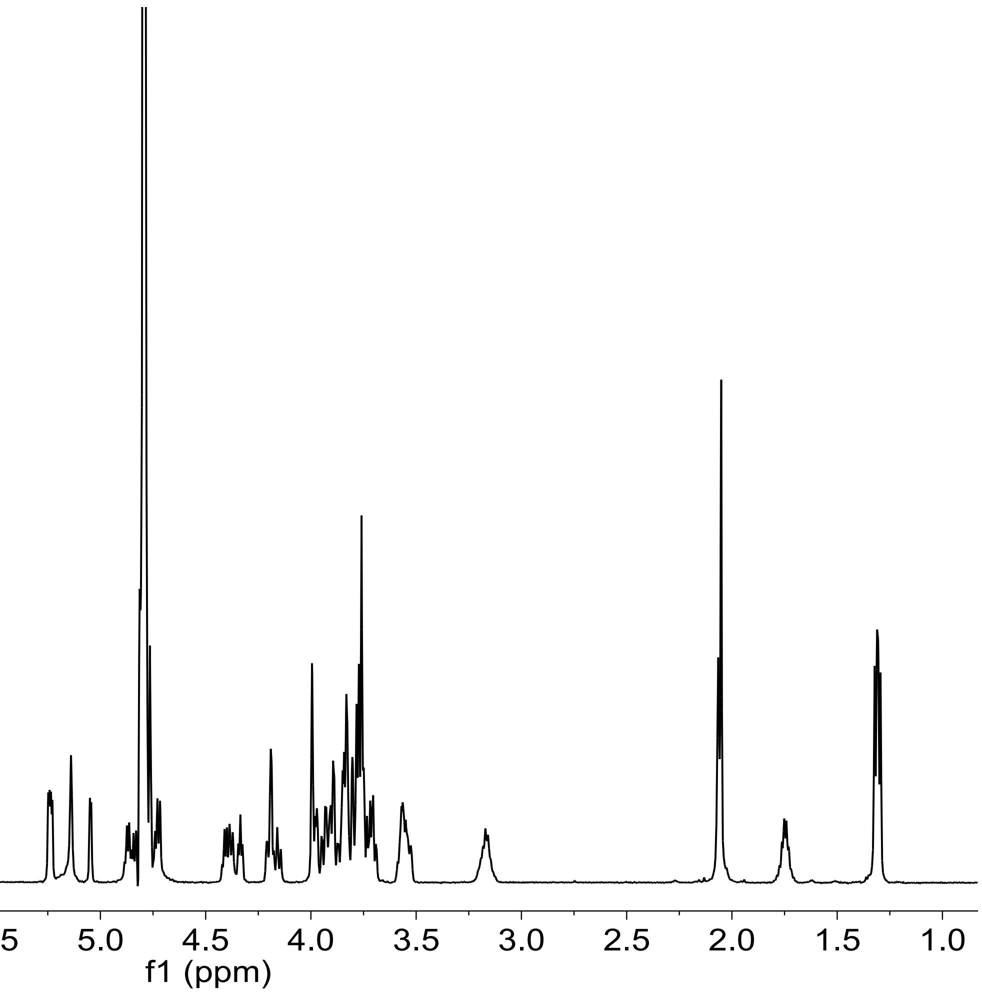

^{1}H NMR of Compound **11**

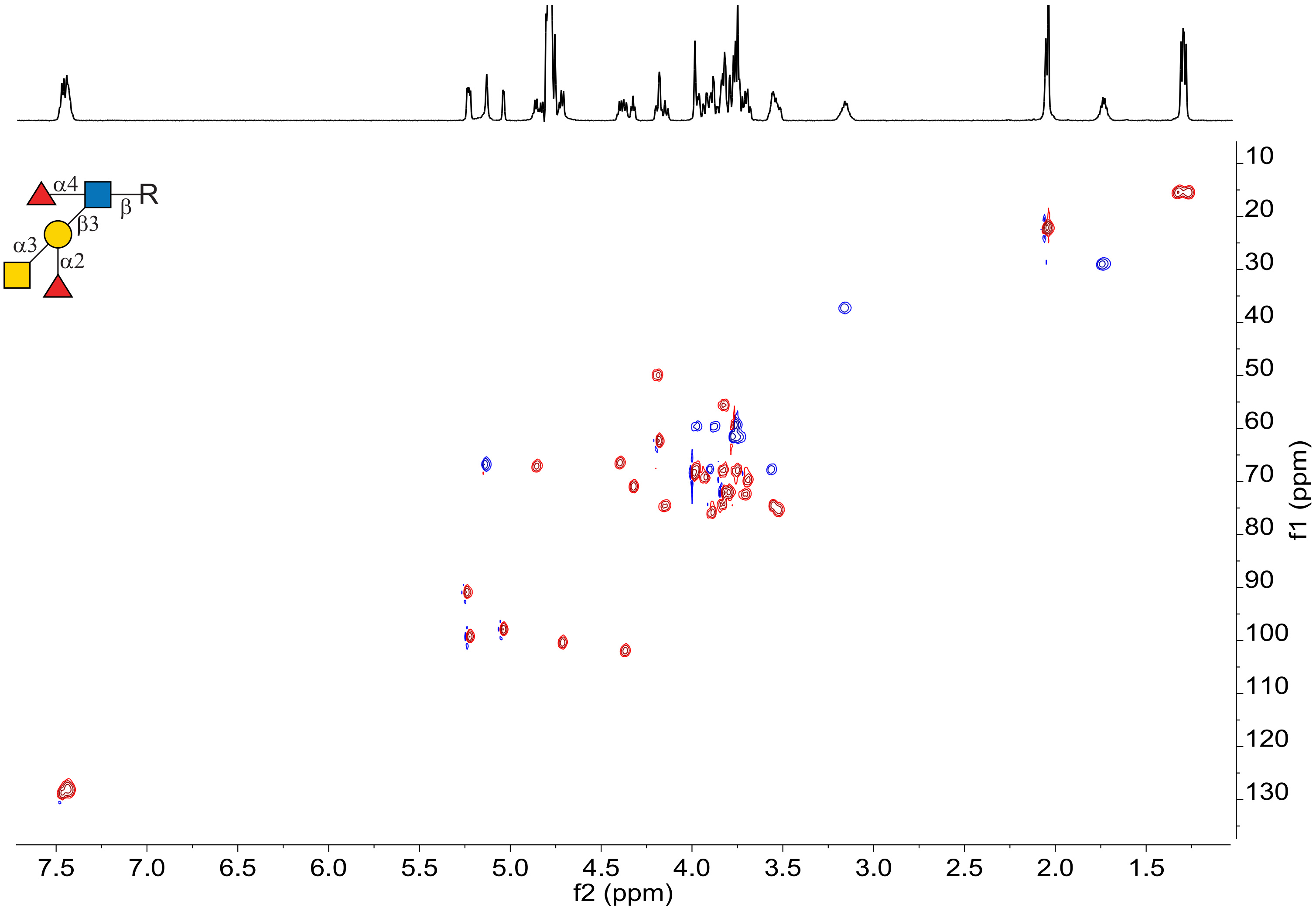

HSQC of Compound **11**

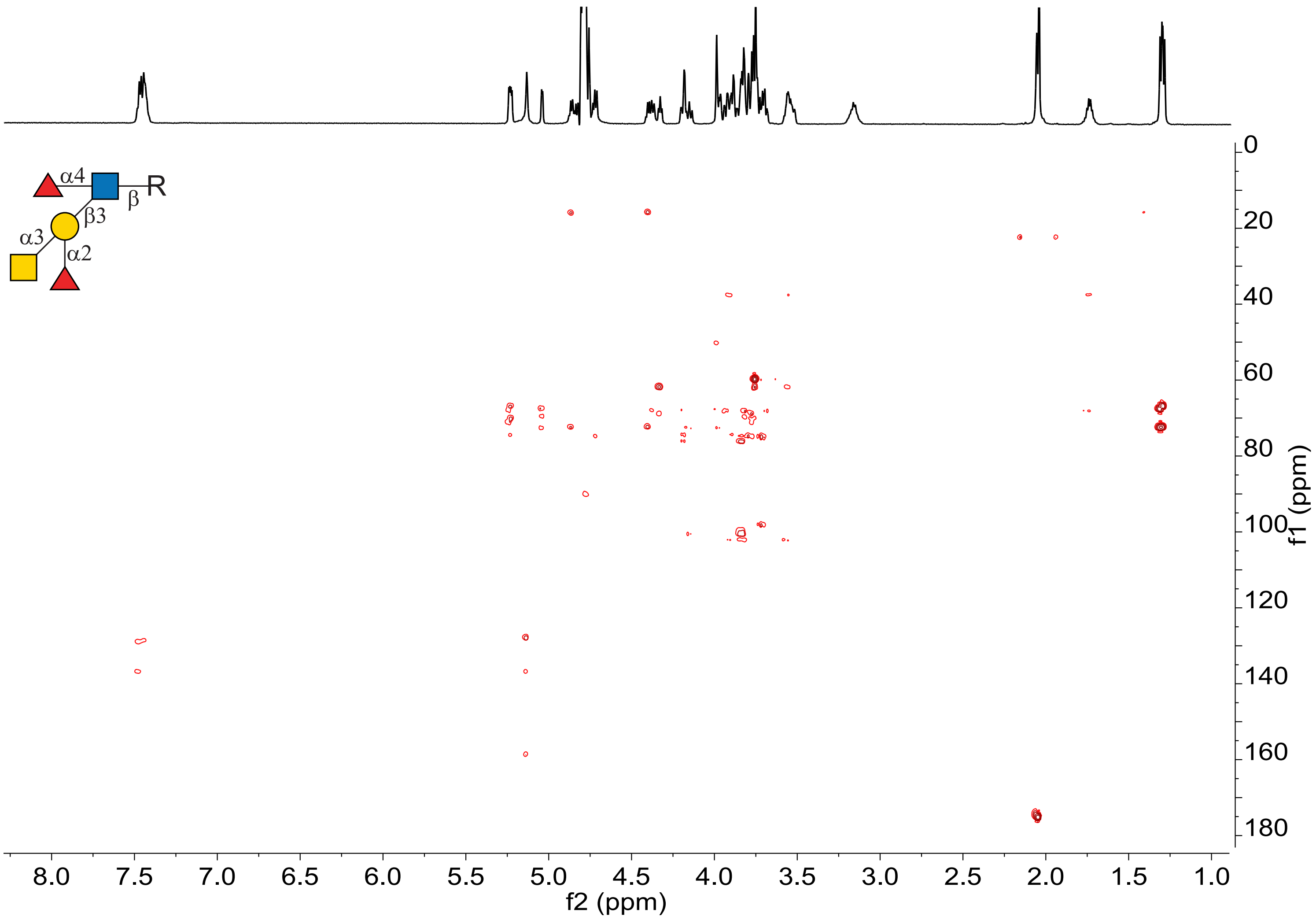

HMBC of Compound **11**

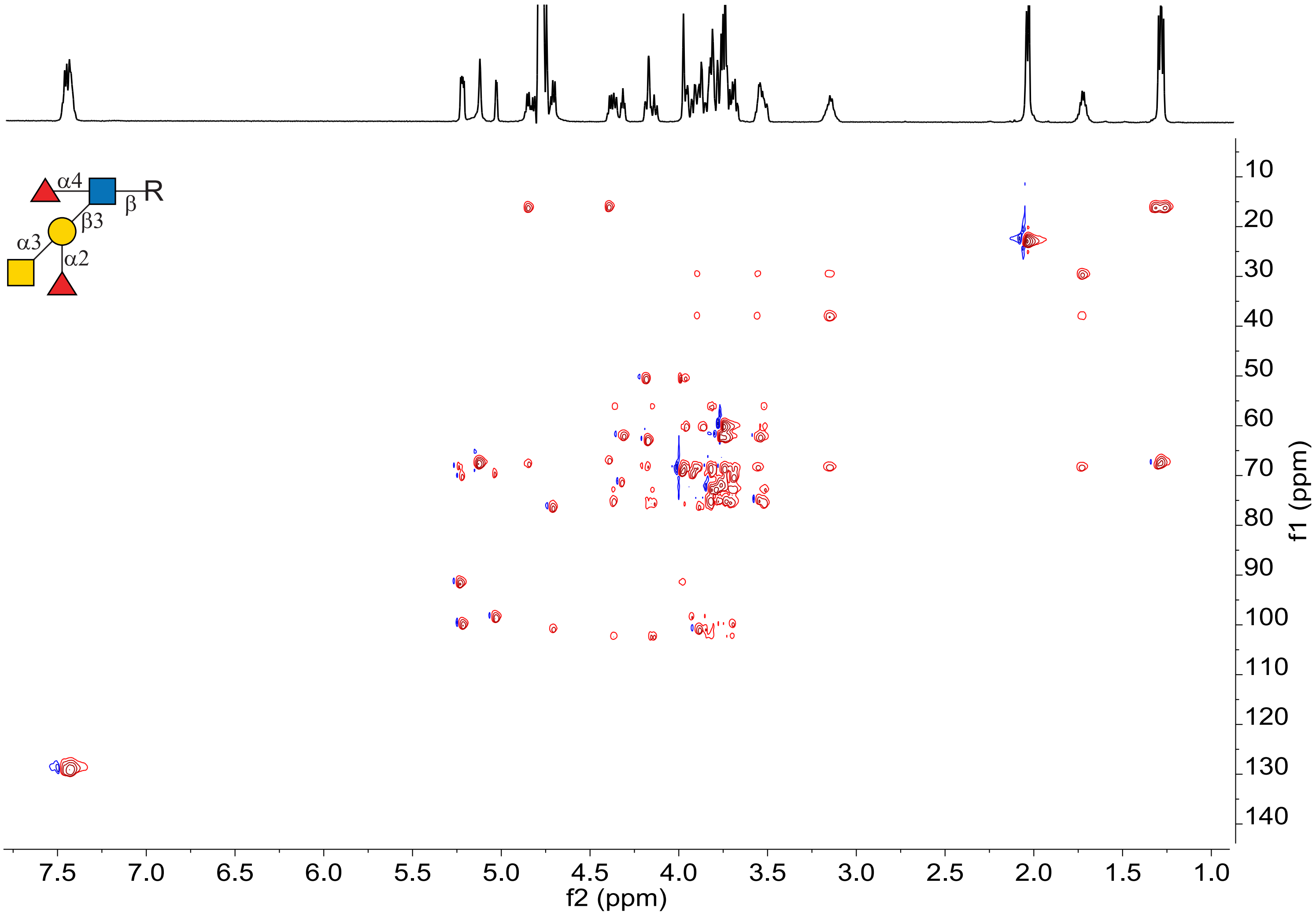

HSQC-TOCSY of Compound **11**

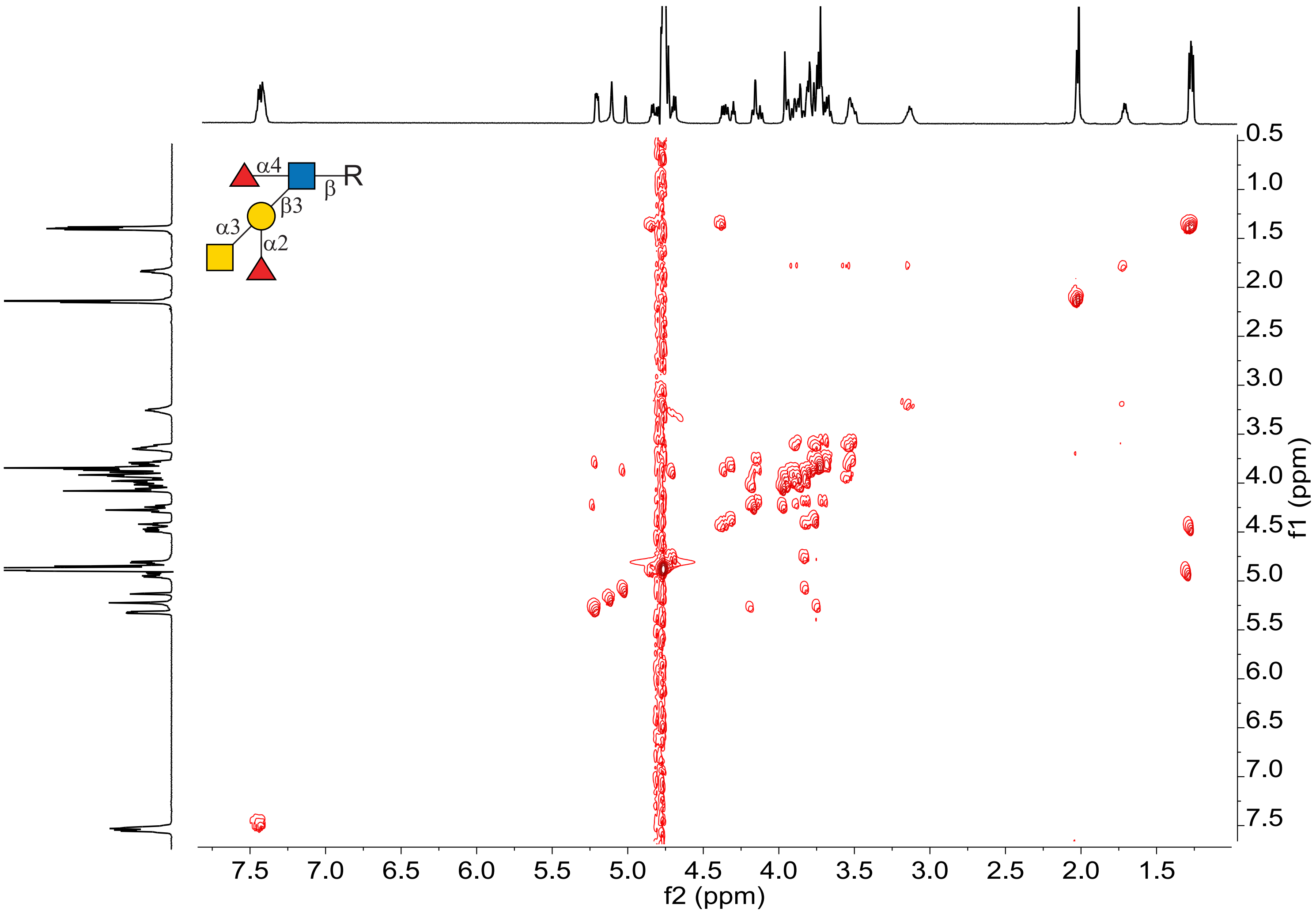

^{1}H-^{1}H COSY of Compound **11**

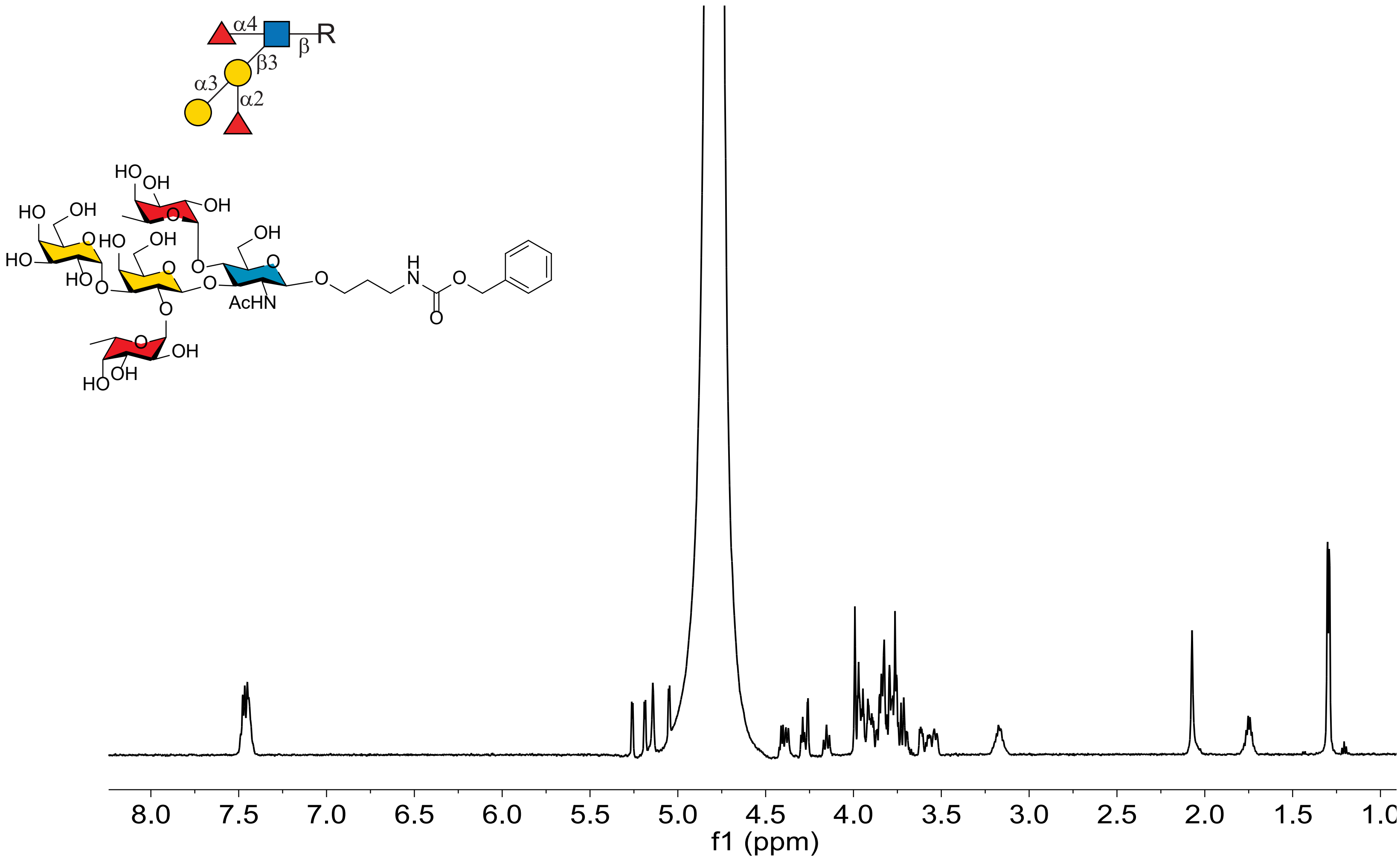

^{1}H NMR of Compound **12**

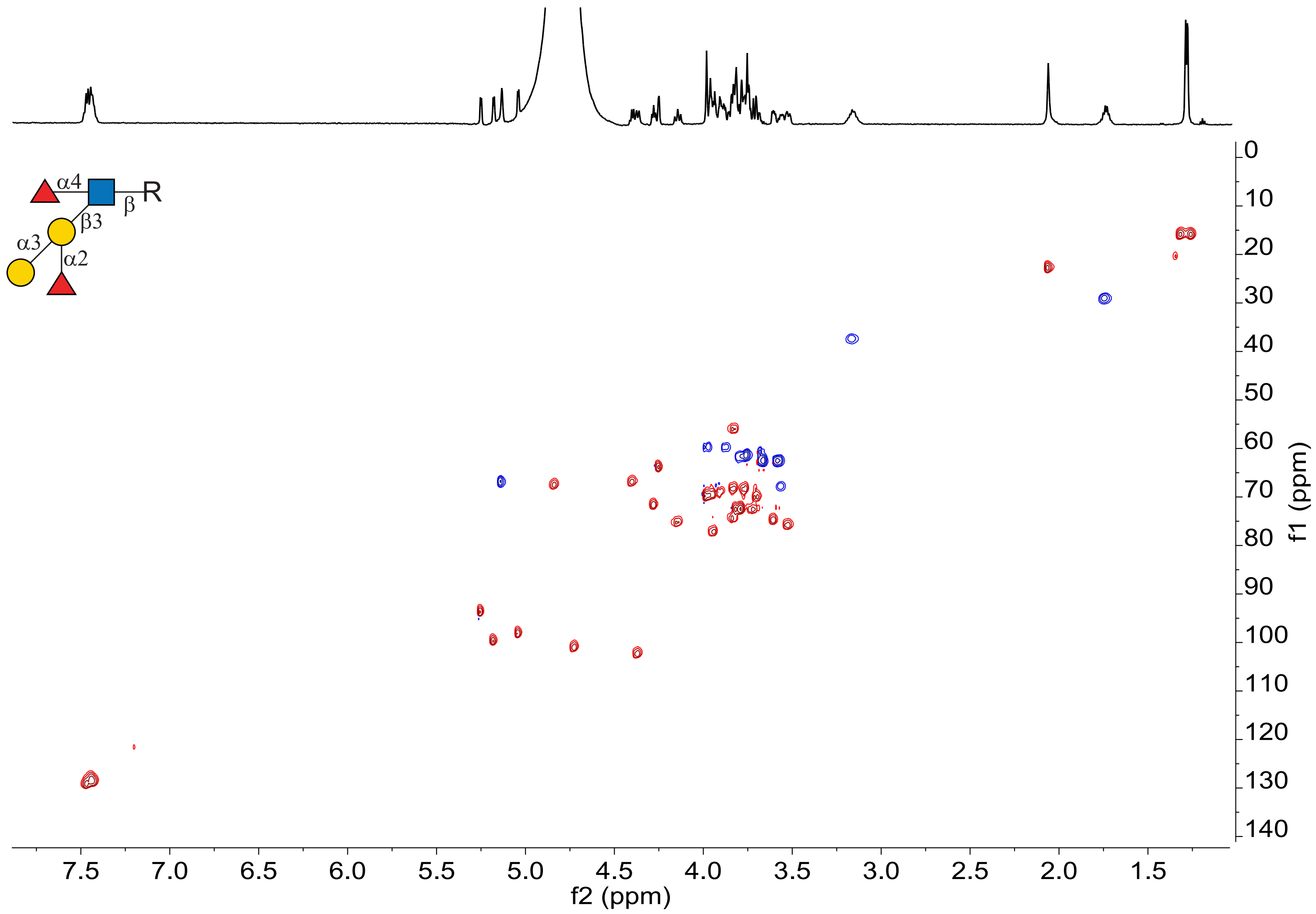

HSQC of Compound **12**

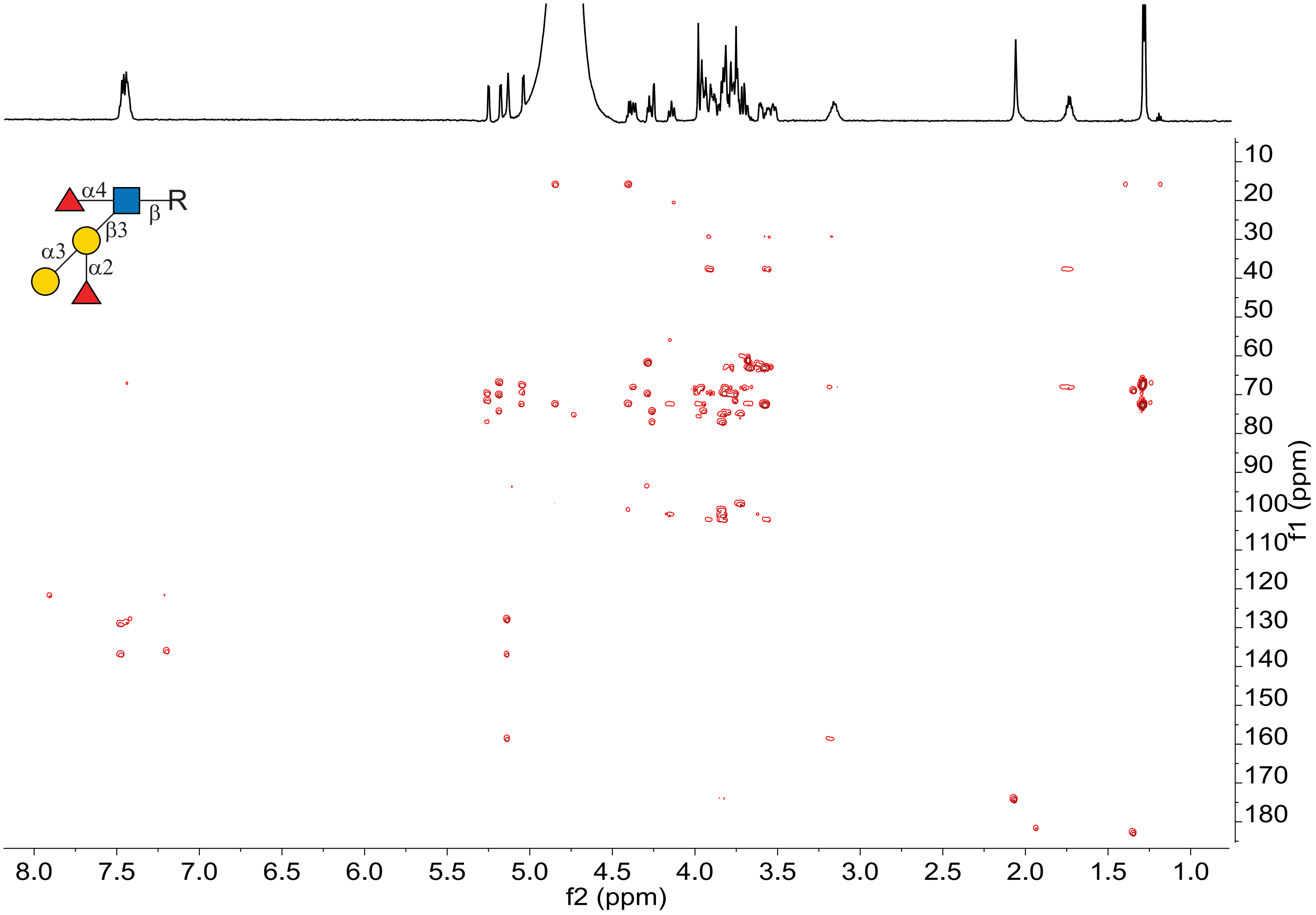

HMBC of Compound **12**

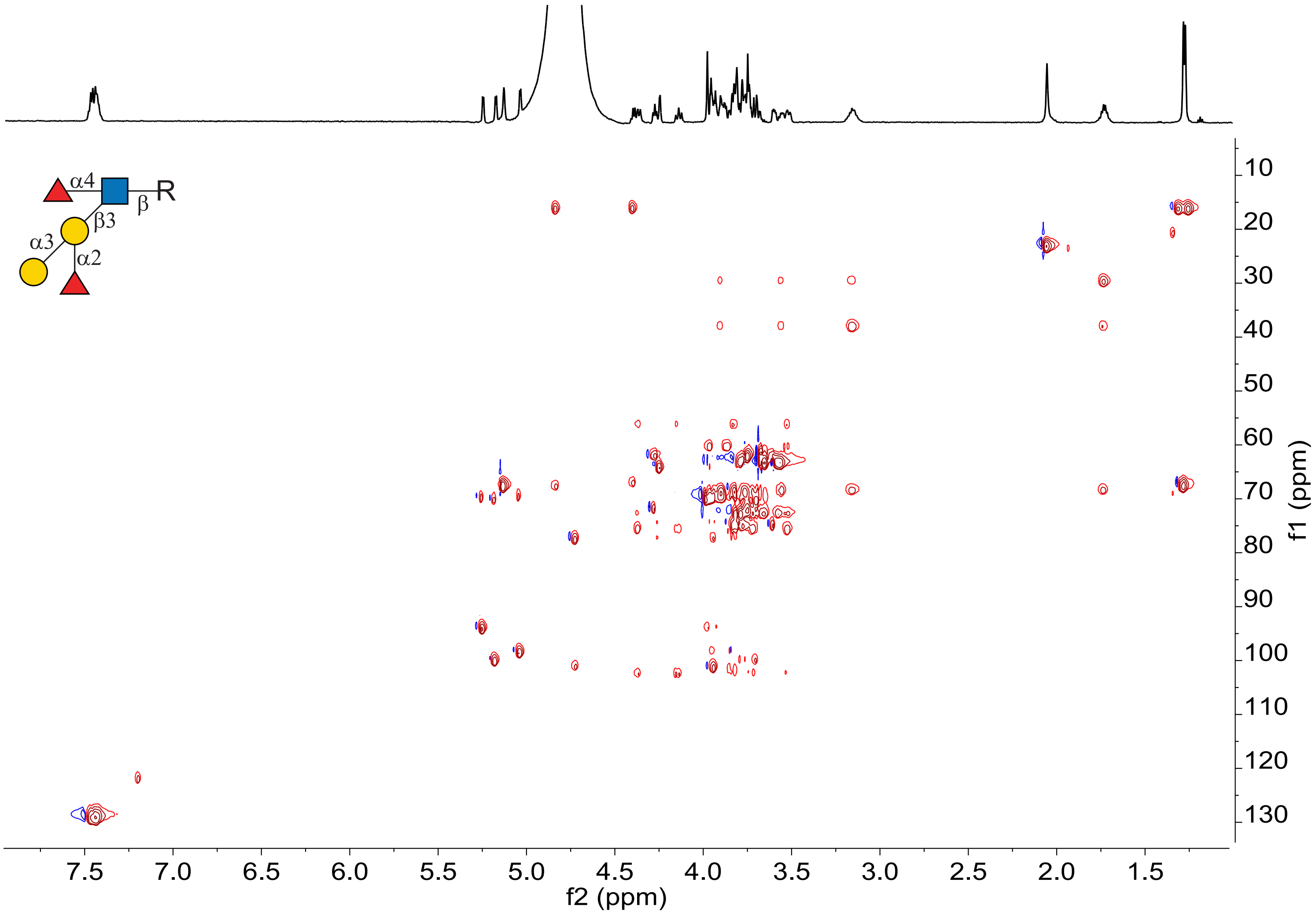

HSQC-TOCSY of Compound **12**

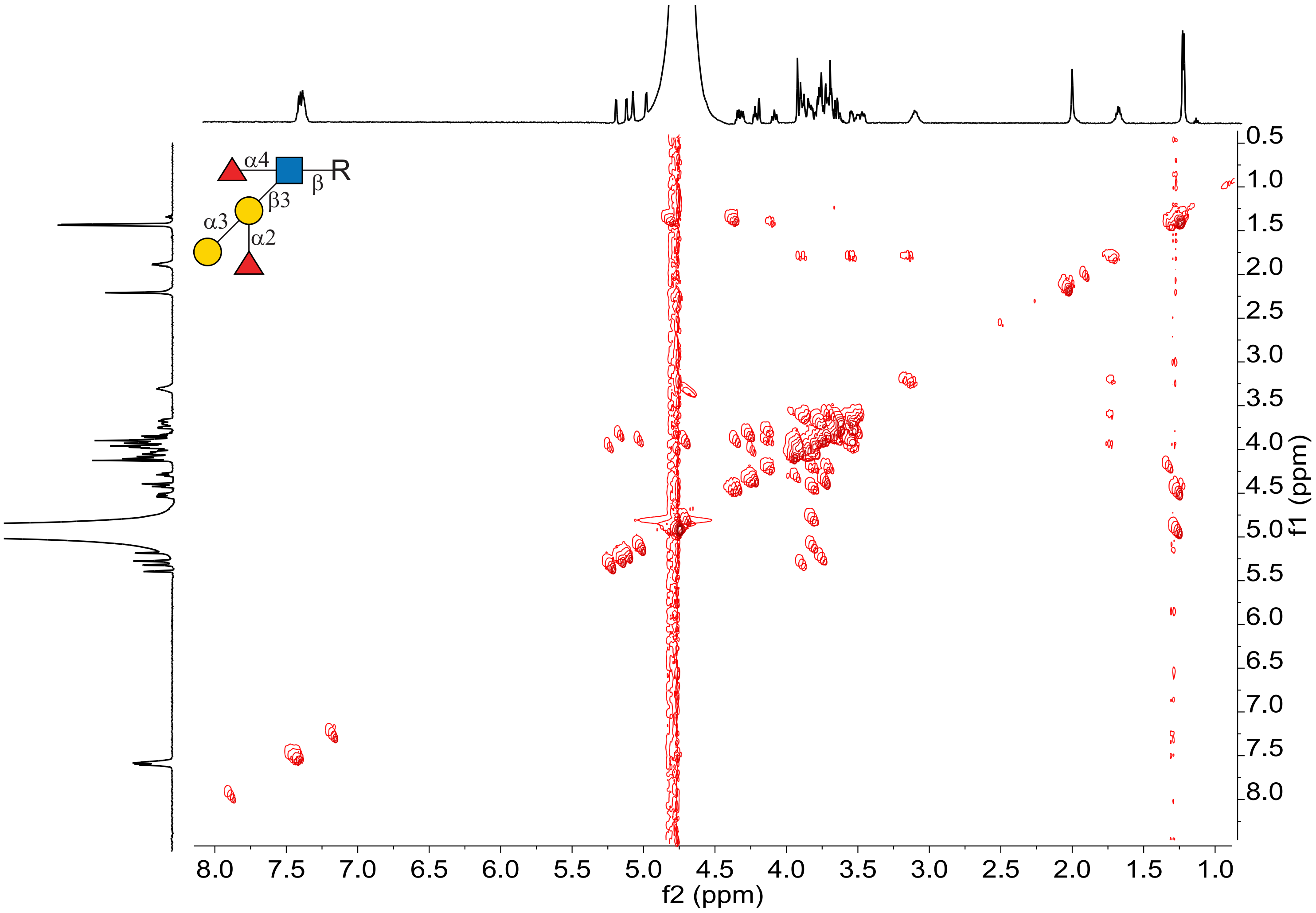

^{1}H-^{1}H COSY of Compound **12**

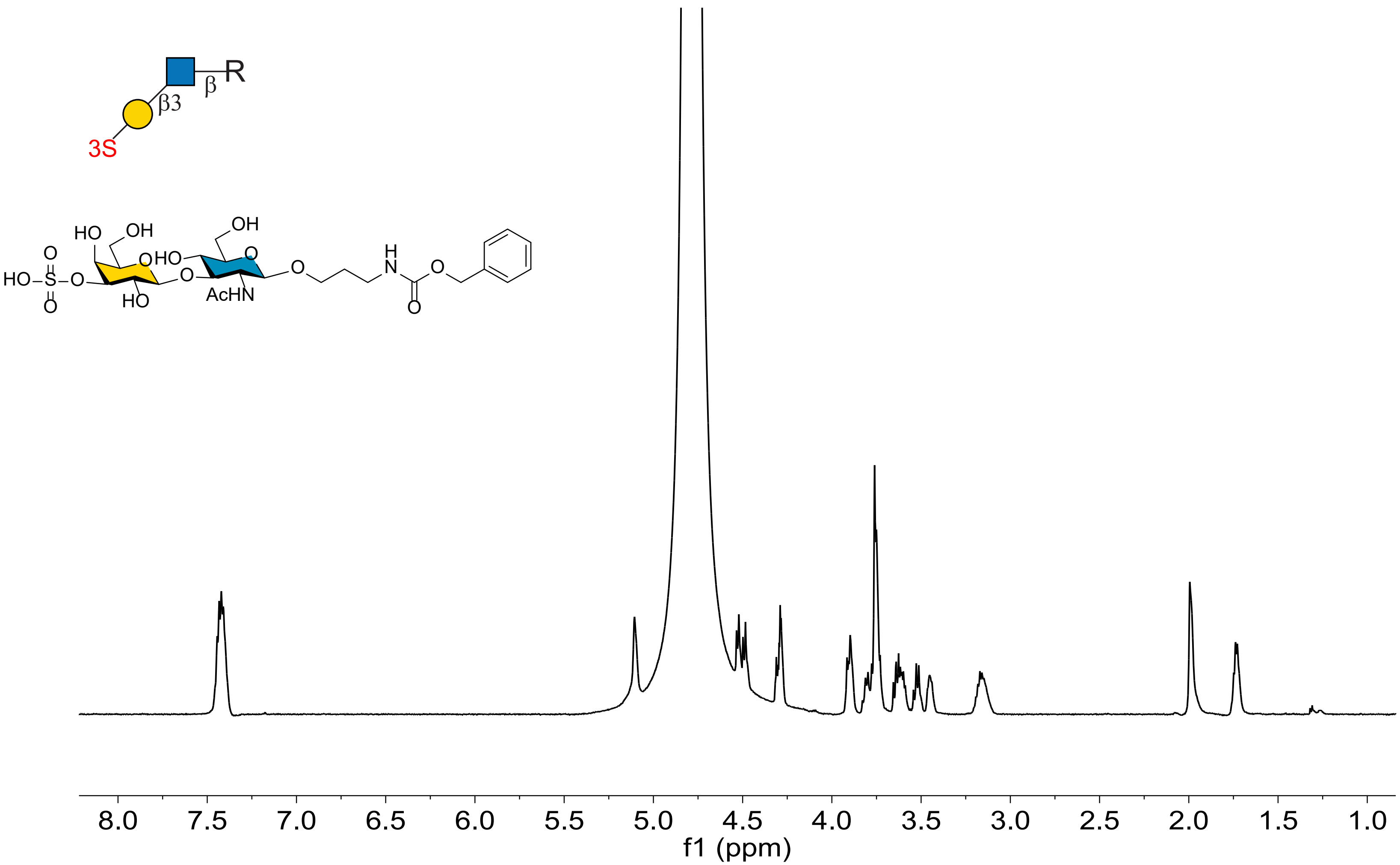

^{1}H NMR of Compound **13**

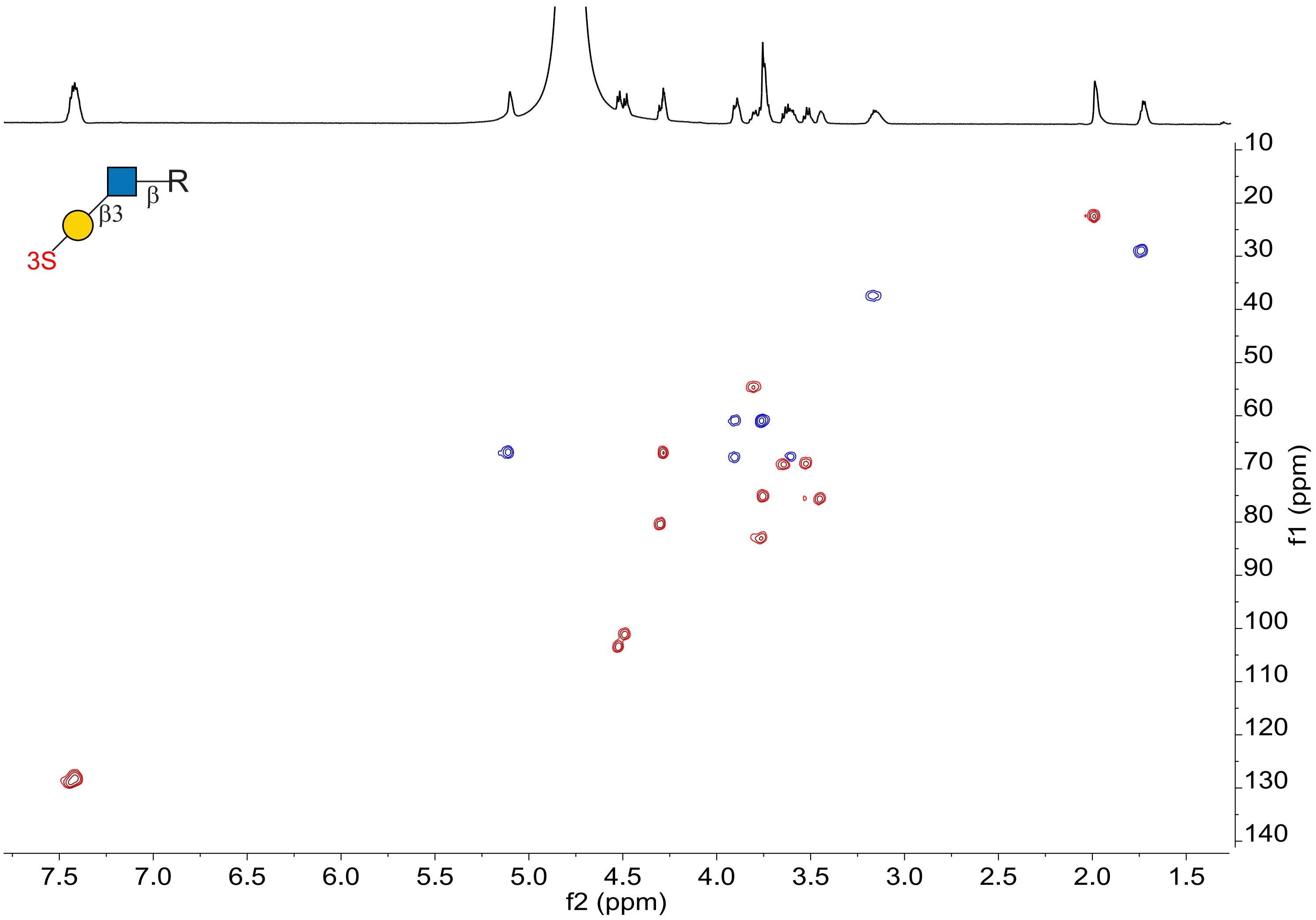

HSQC of Compound **13**

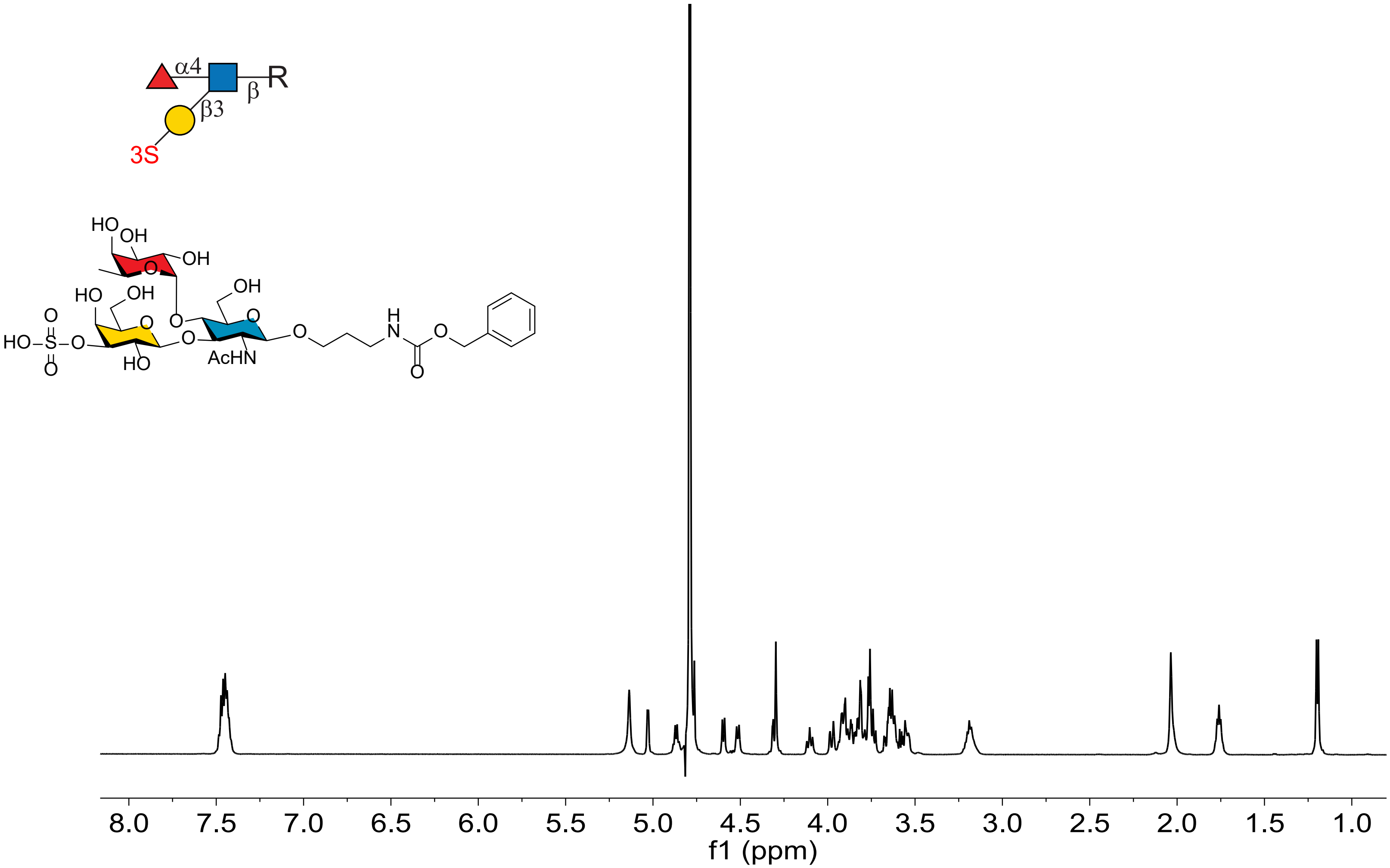

^{1}H NMR of Compound **14**

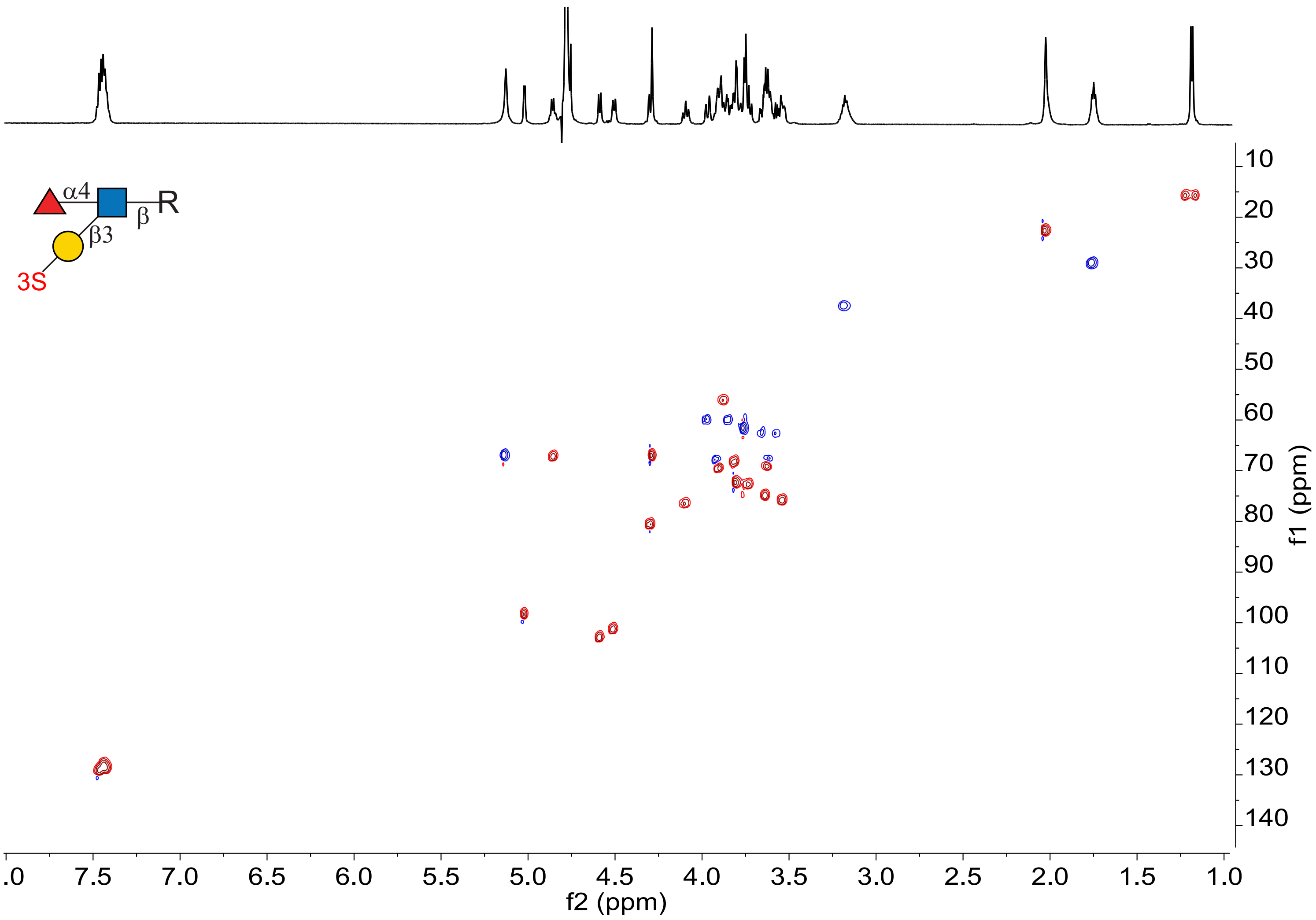

HSQC of Compound **14**

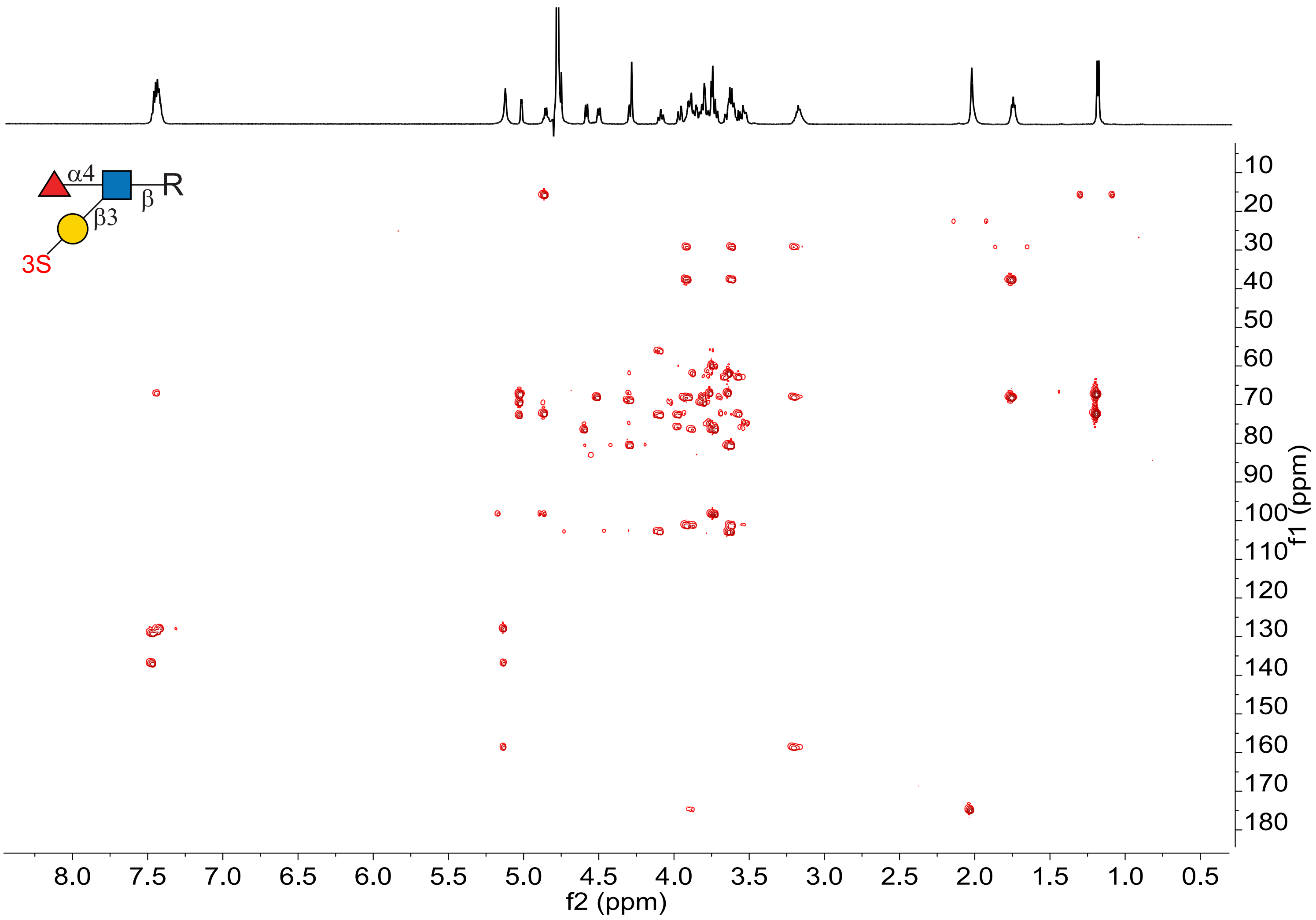

HMBC of Compound **14**

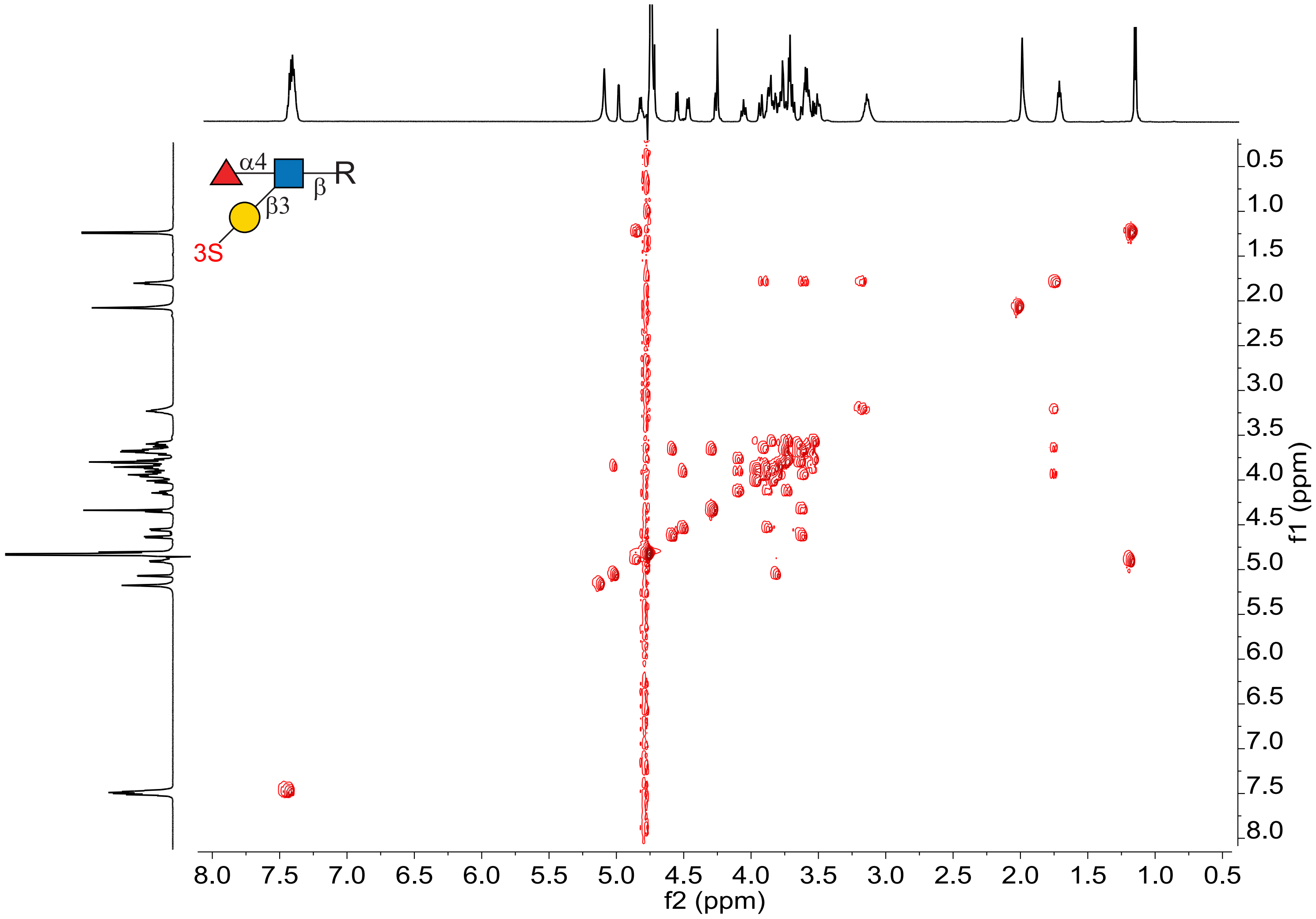

^{1}H-^{1}H COSY of Compound **14**

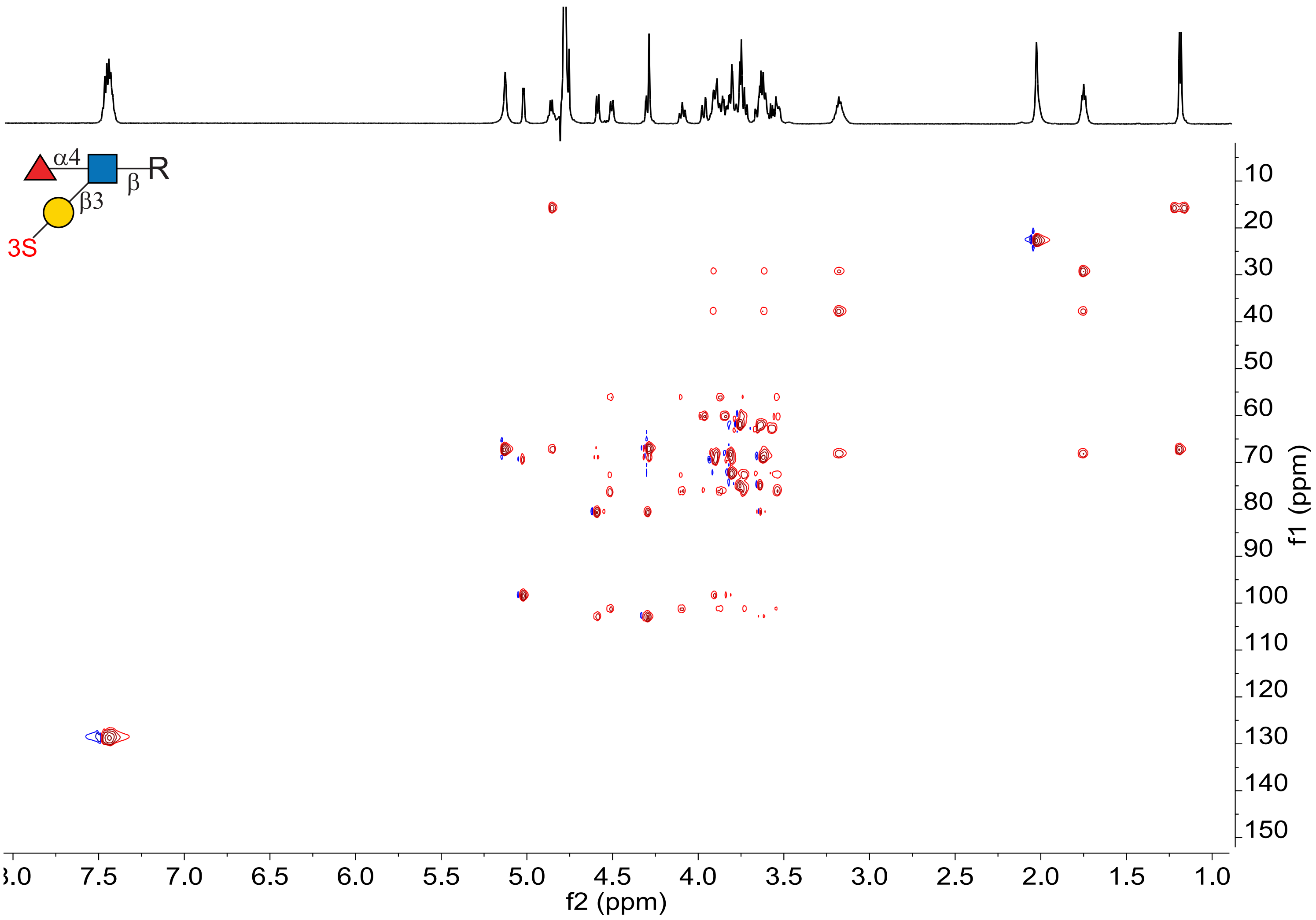

HSQC-TOCSY of Compound **14**

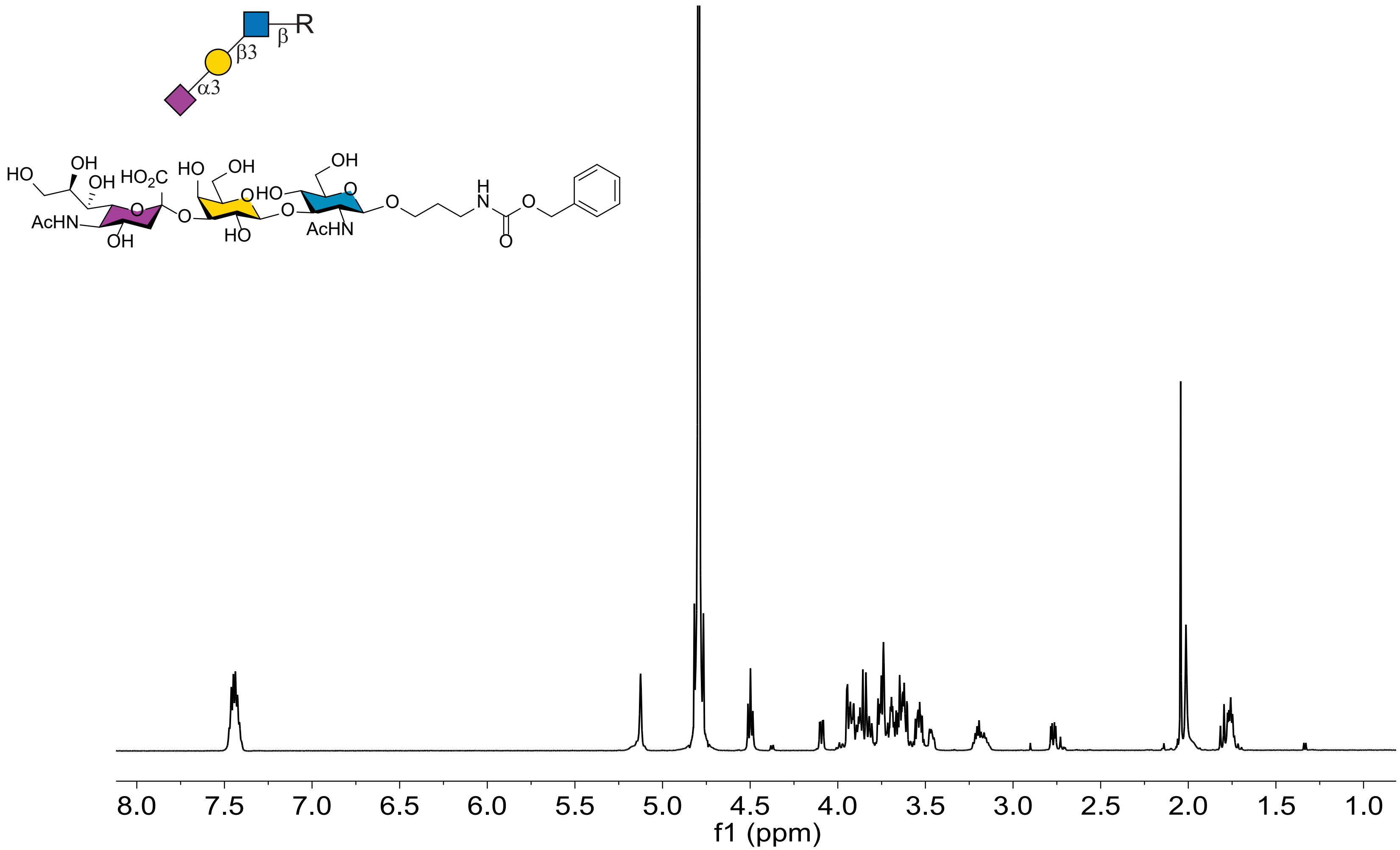

^{1}H NMR of Compound **15**

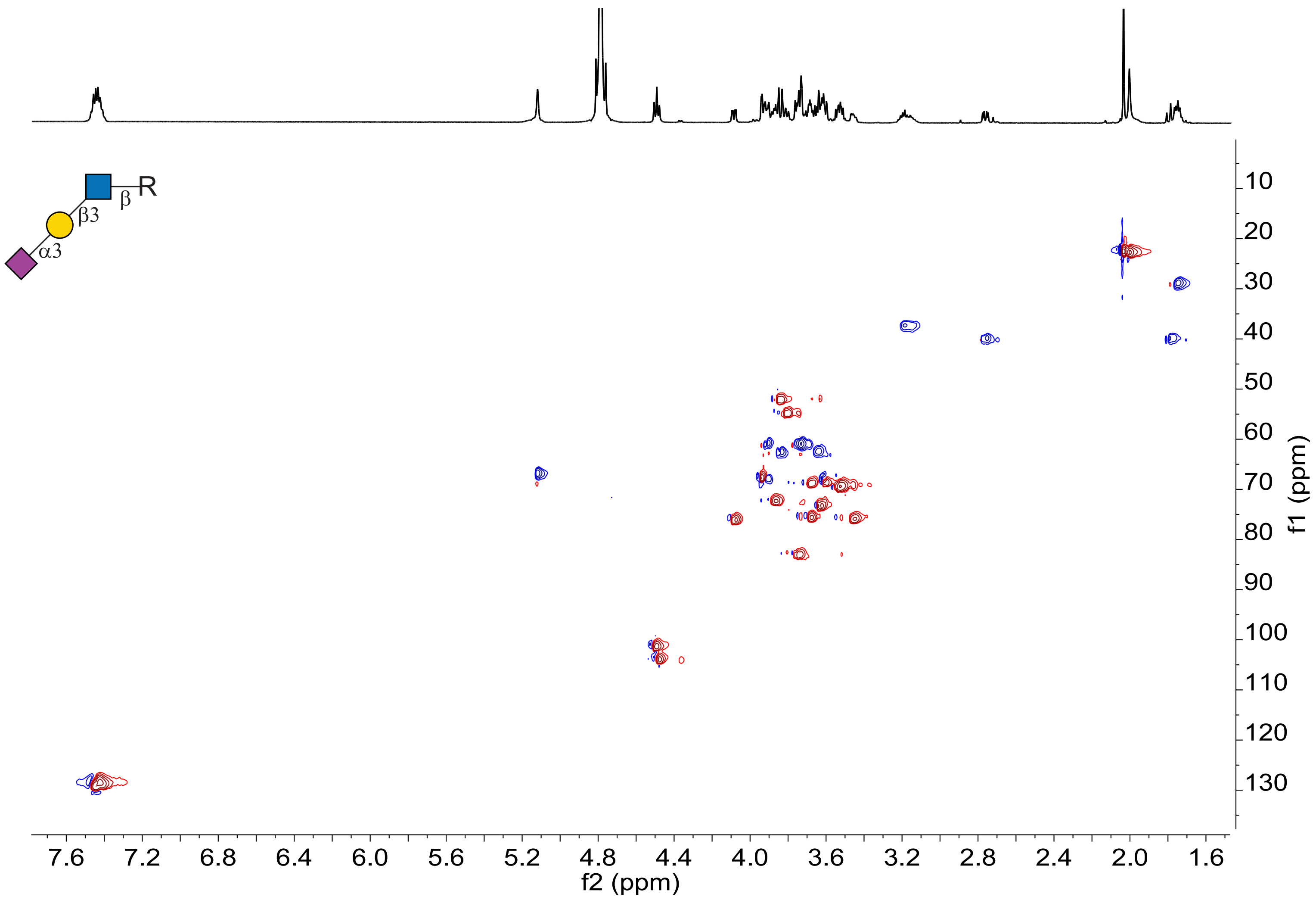

HSQC of Compound **15**

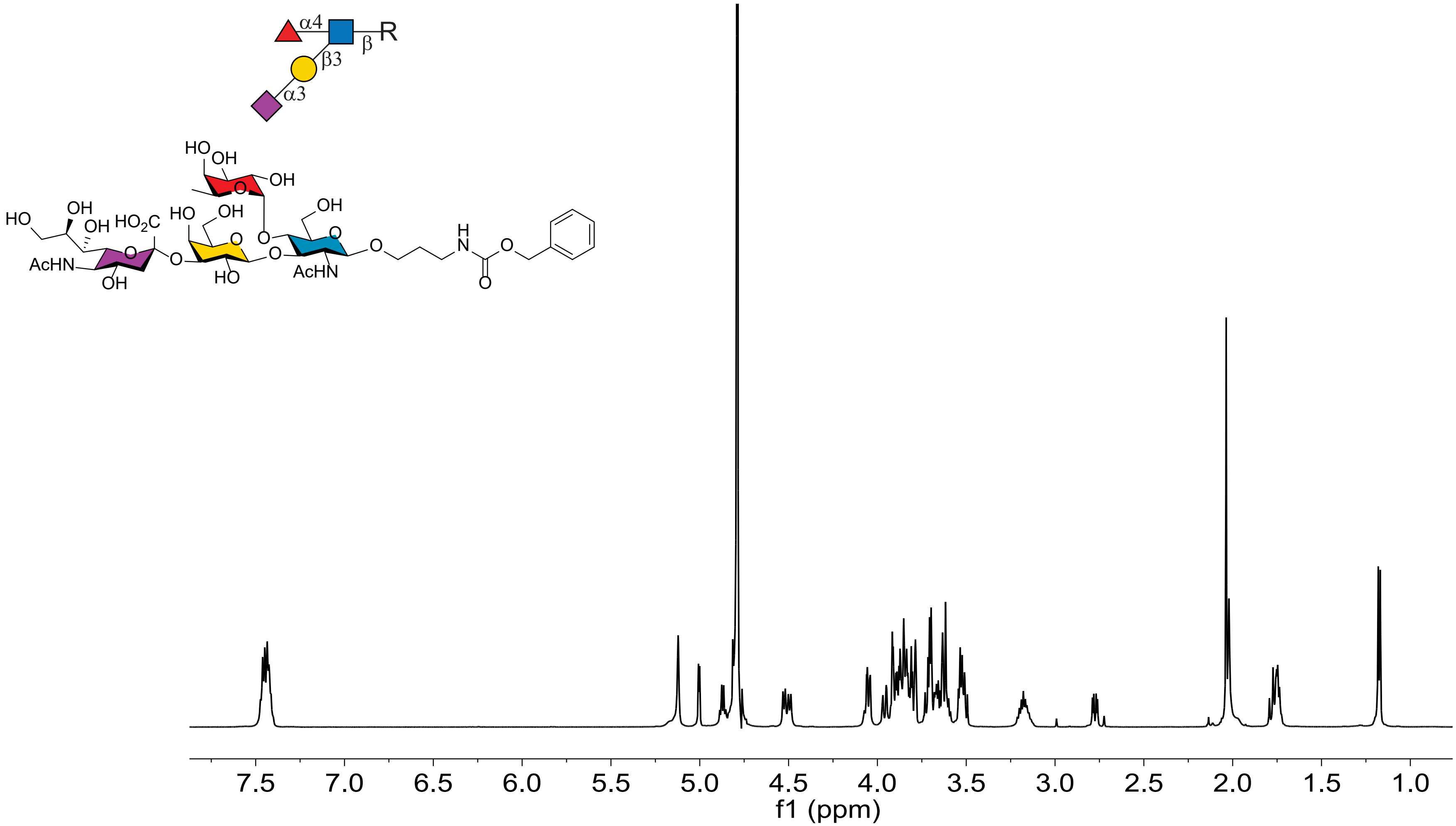

^{1}H NMR of Compound **16**

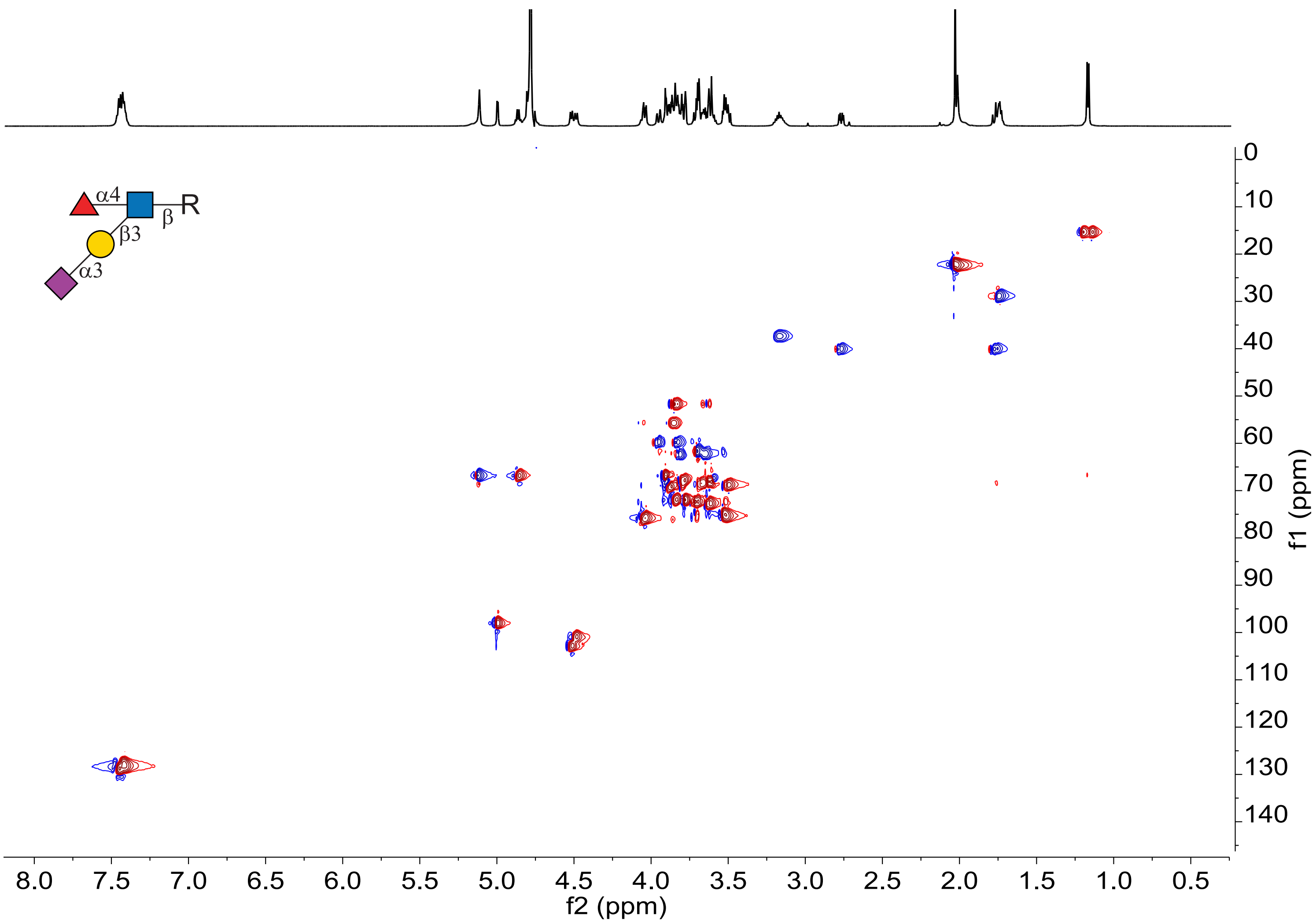

HSQC of Compound **16**

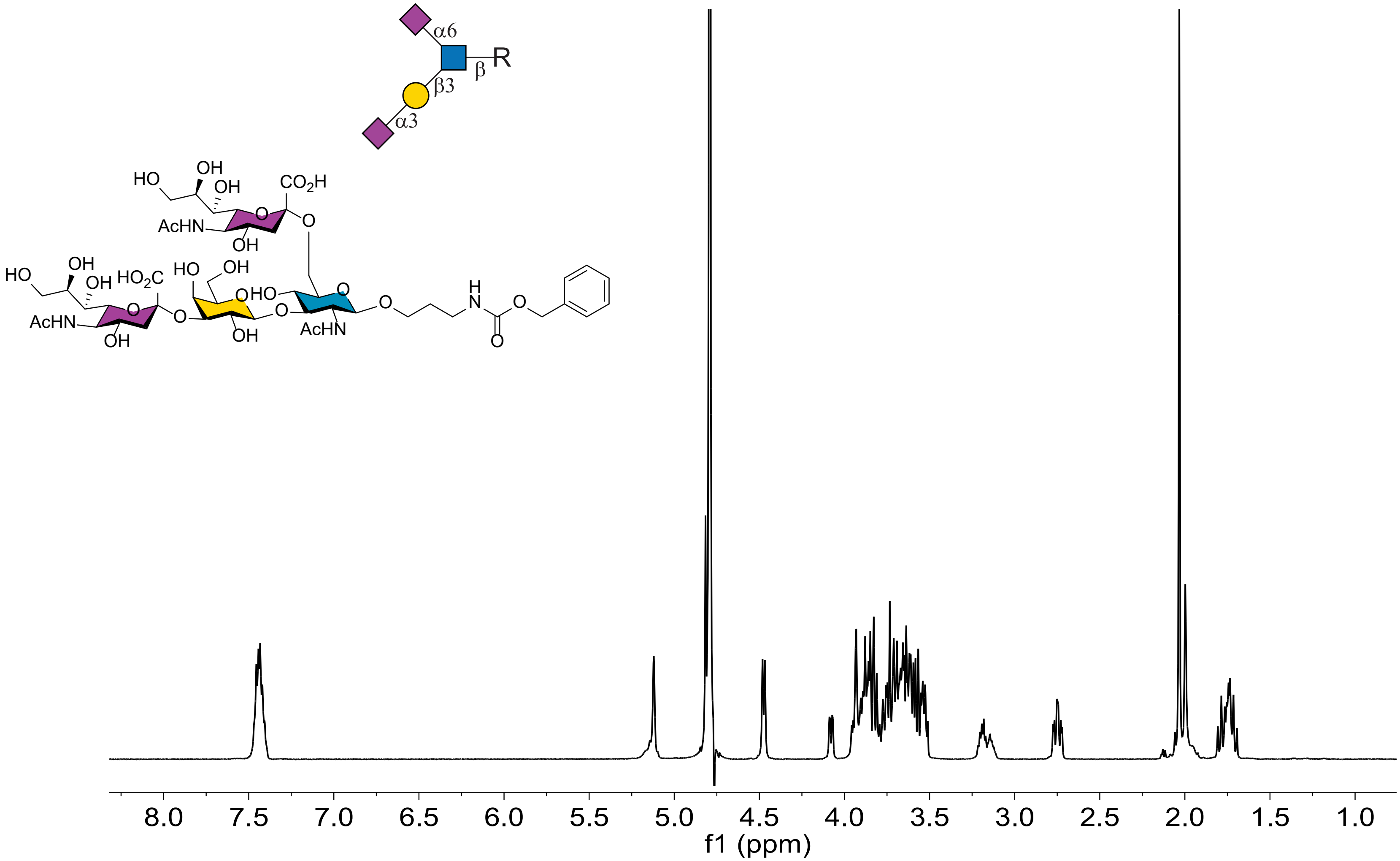

^{1}H NMR of Compound **17**

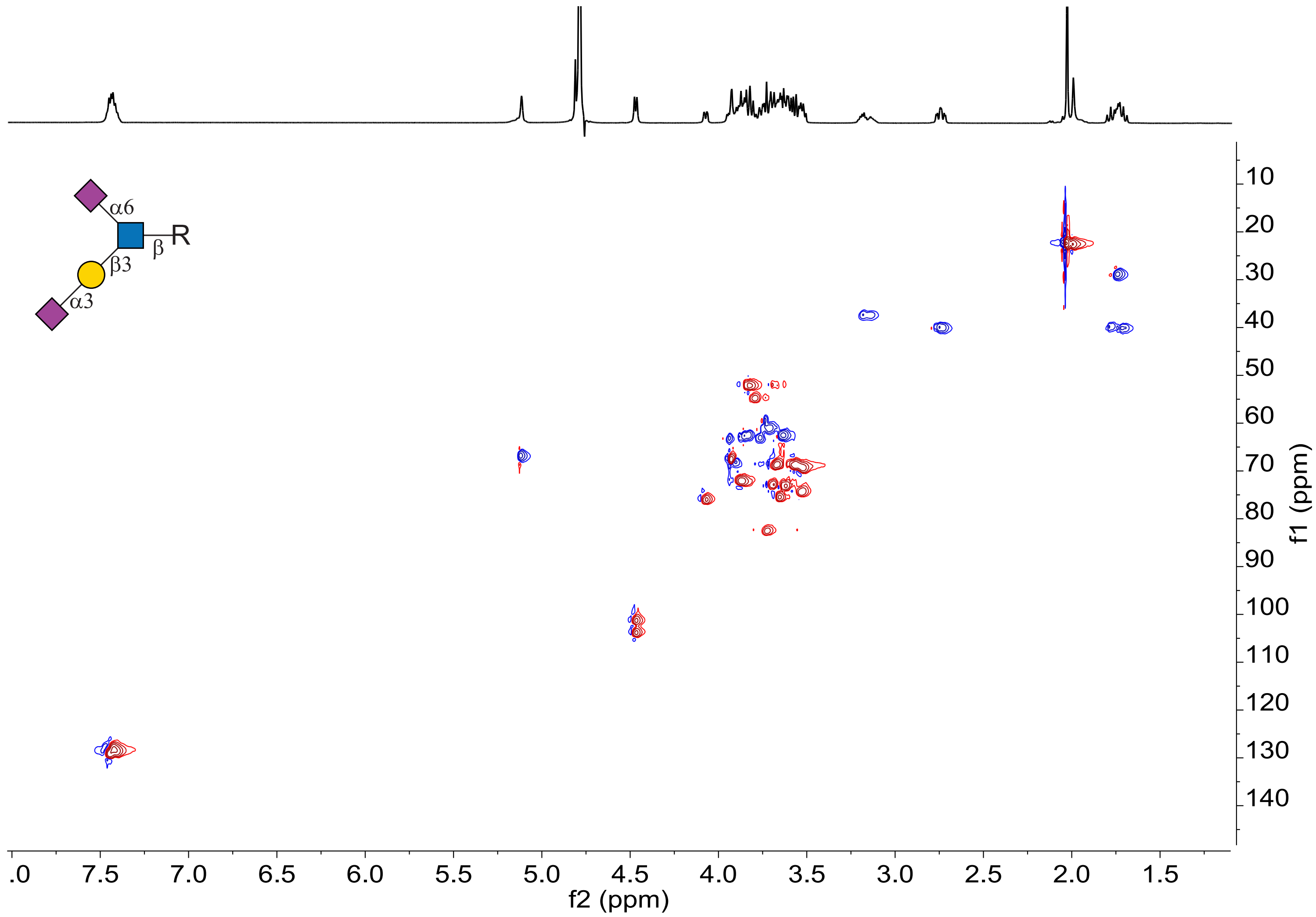

HSQC of Compound **17**

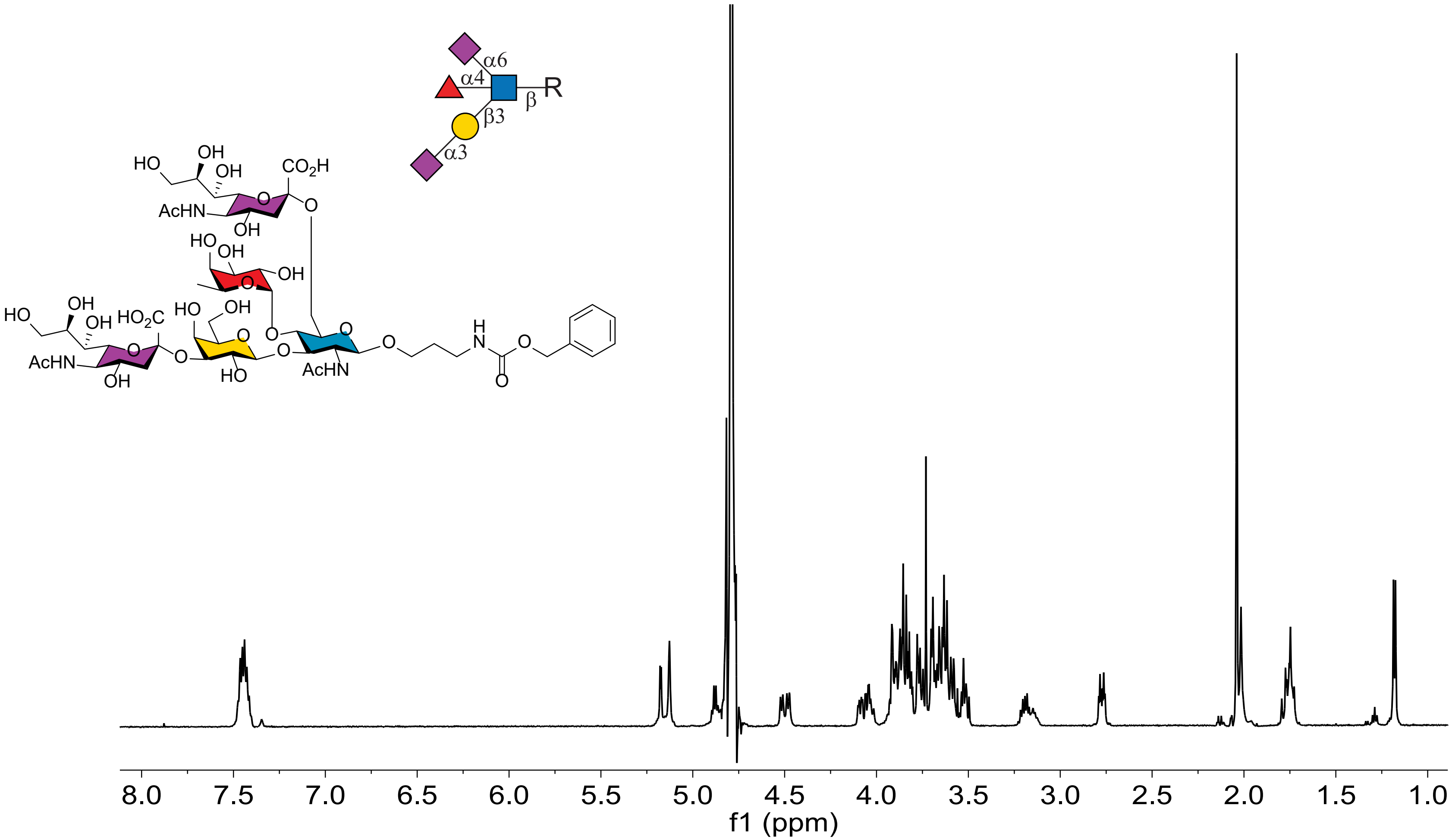

^{1}H NMR of Compound **18**

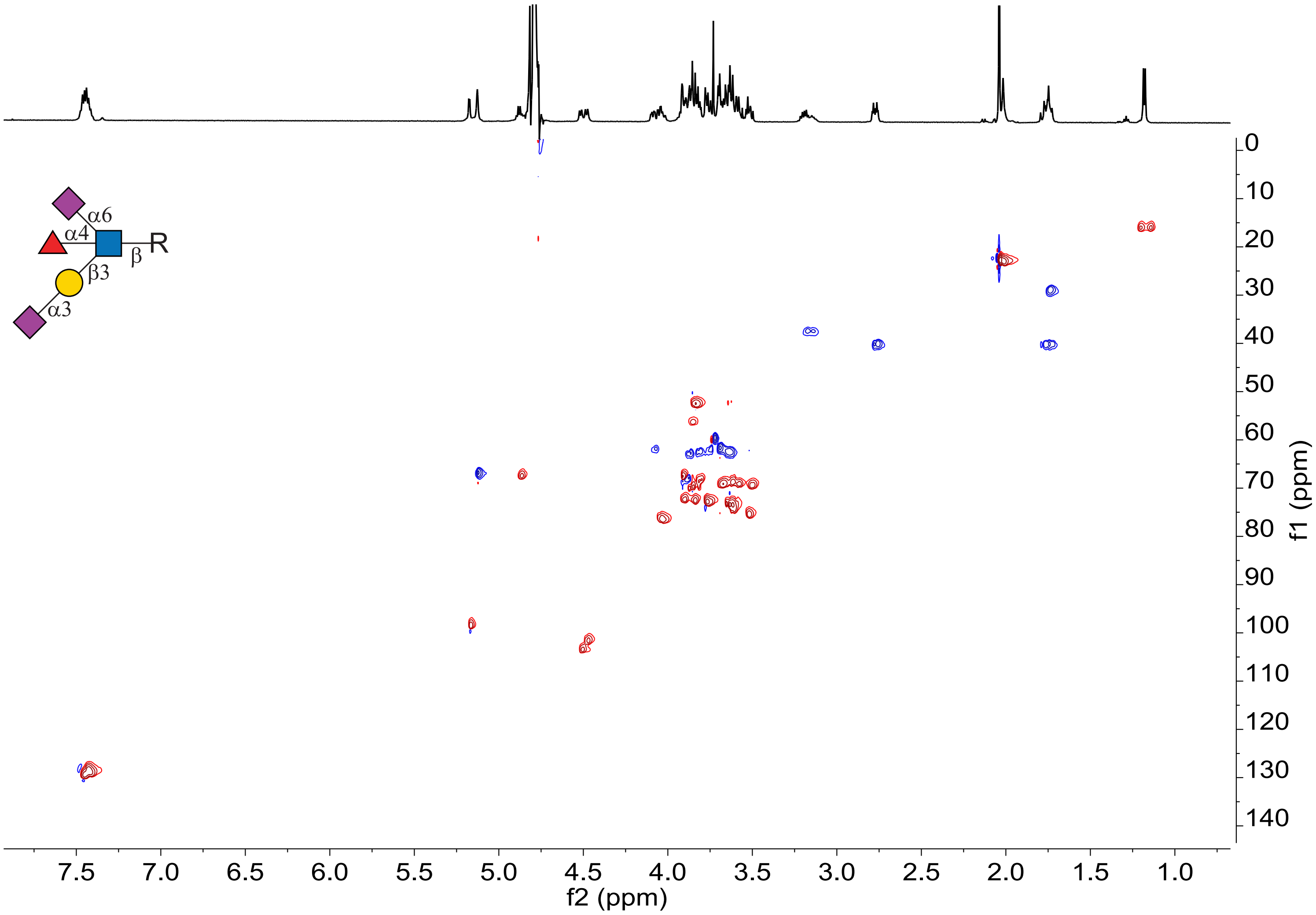

HSQC of Compound **18**

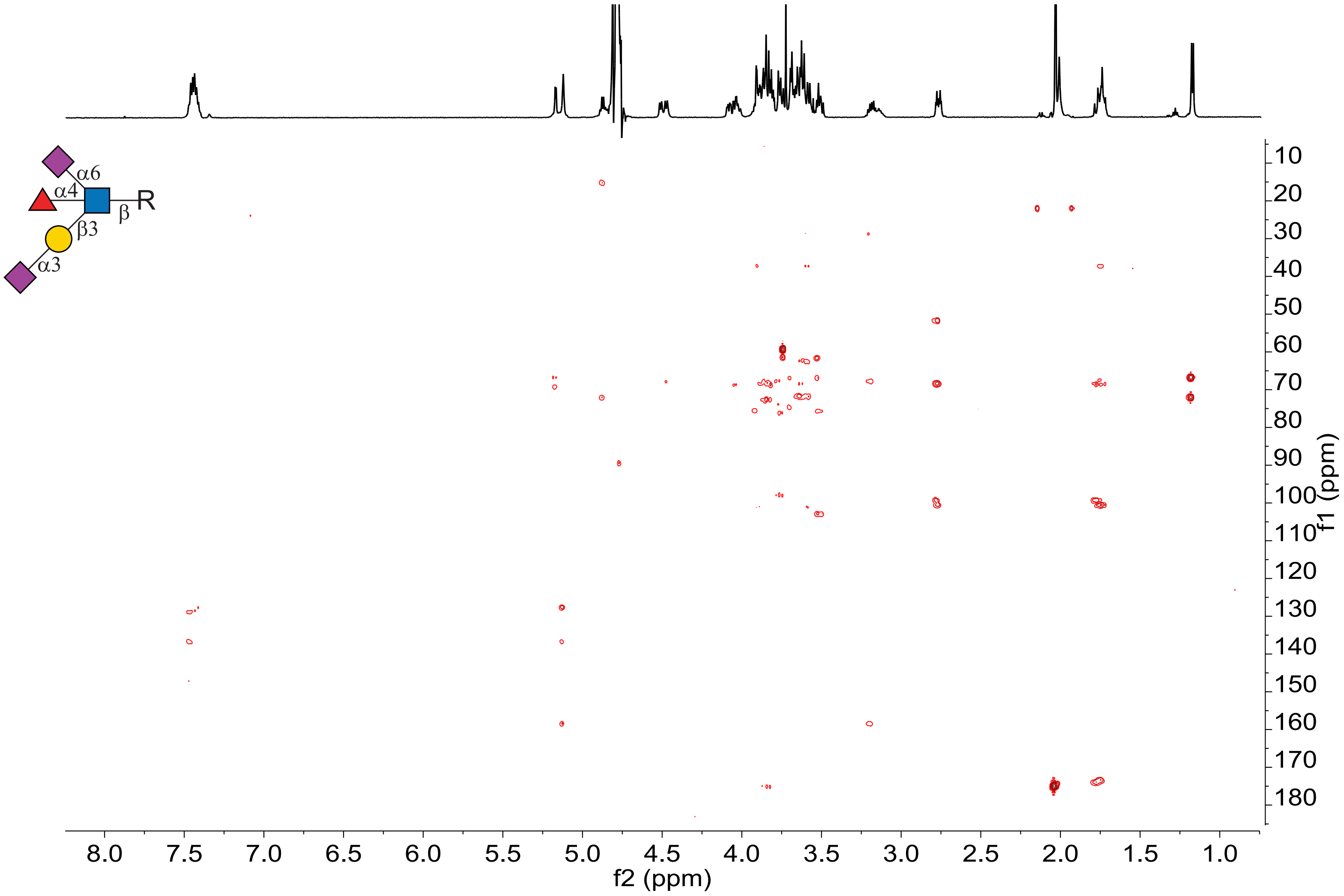

HMBC of Compound **18**

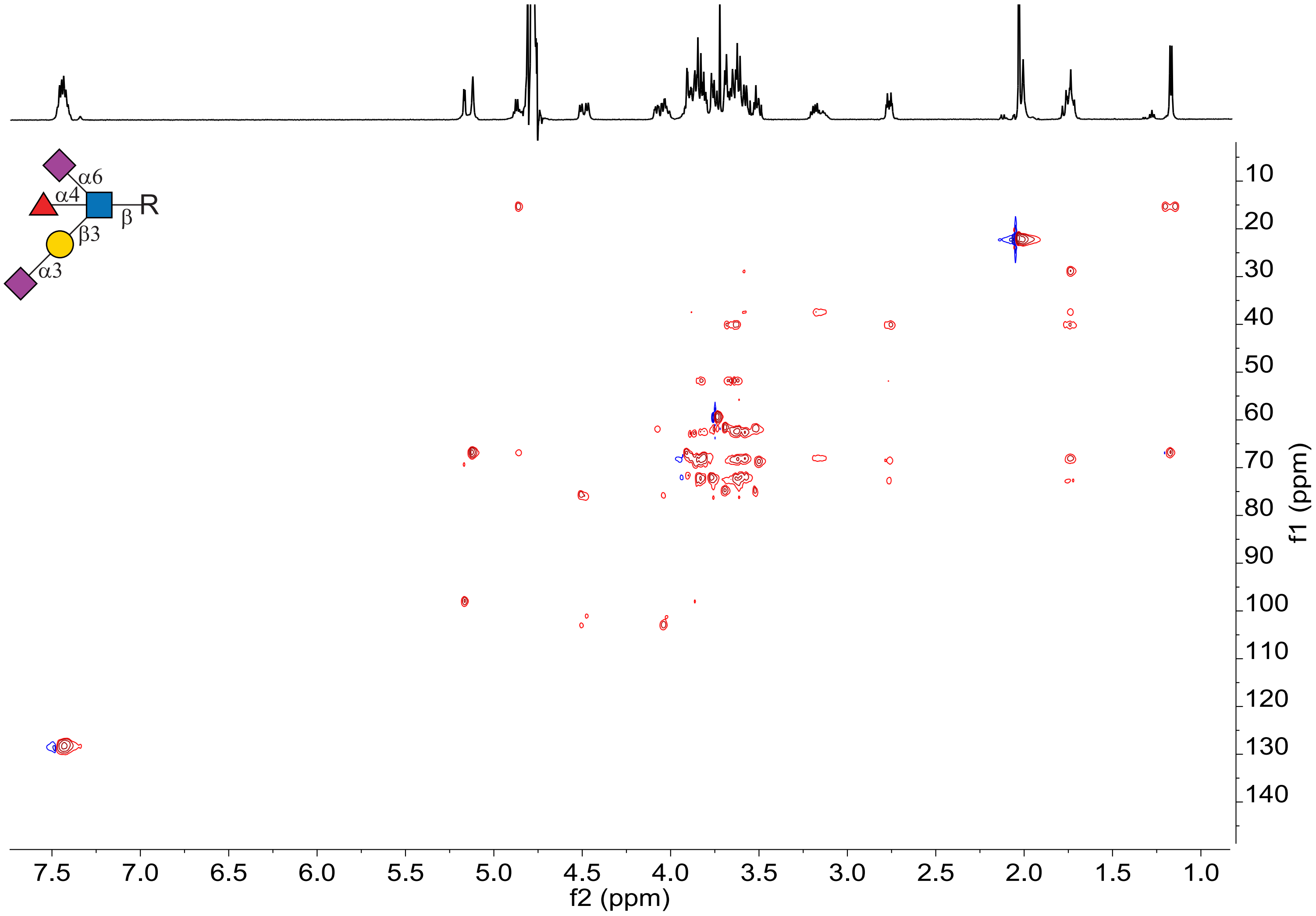

HSQC-TOCSY of Compound **18**

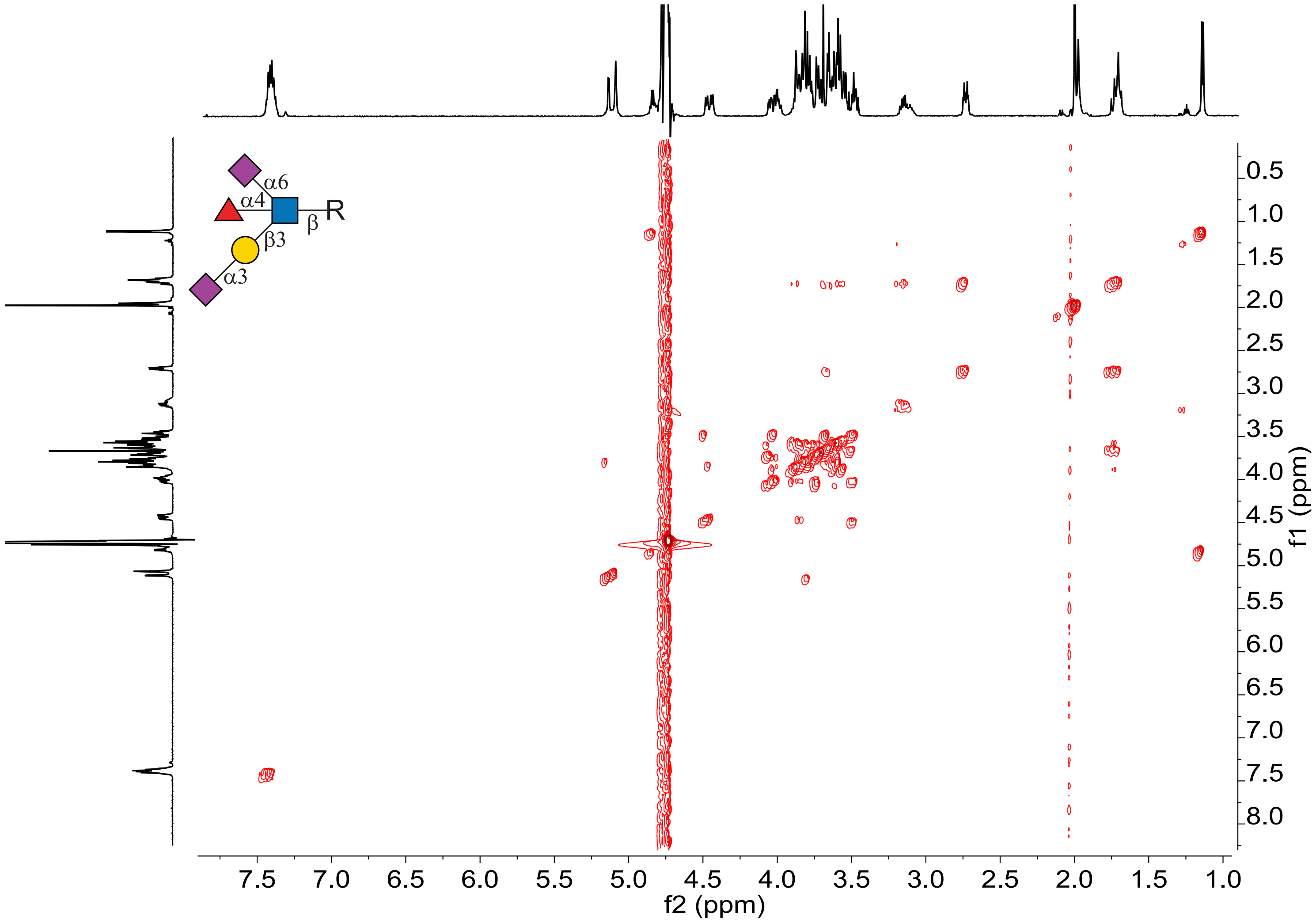

^{1}H-^{1}H COSY of Compound **18**

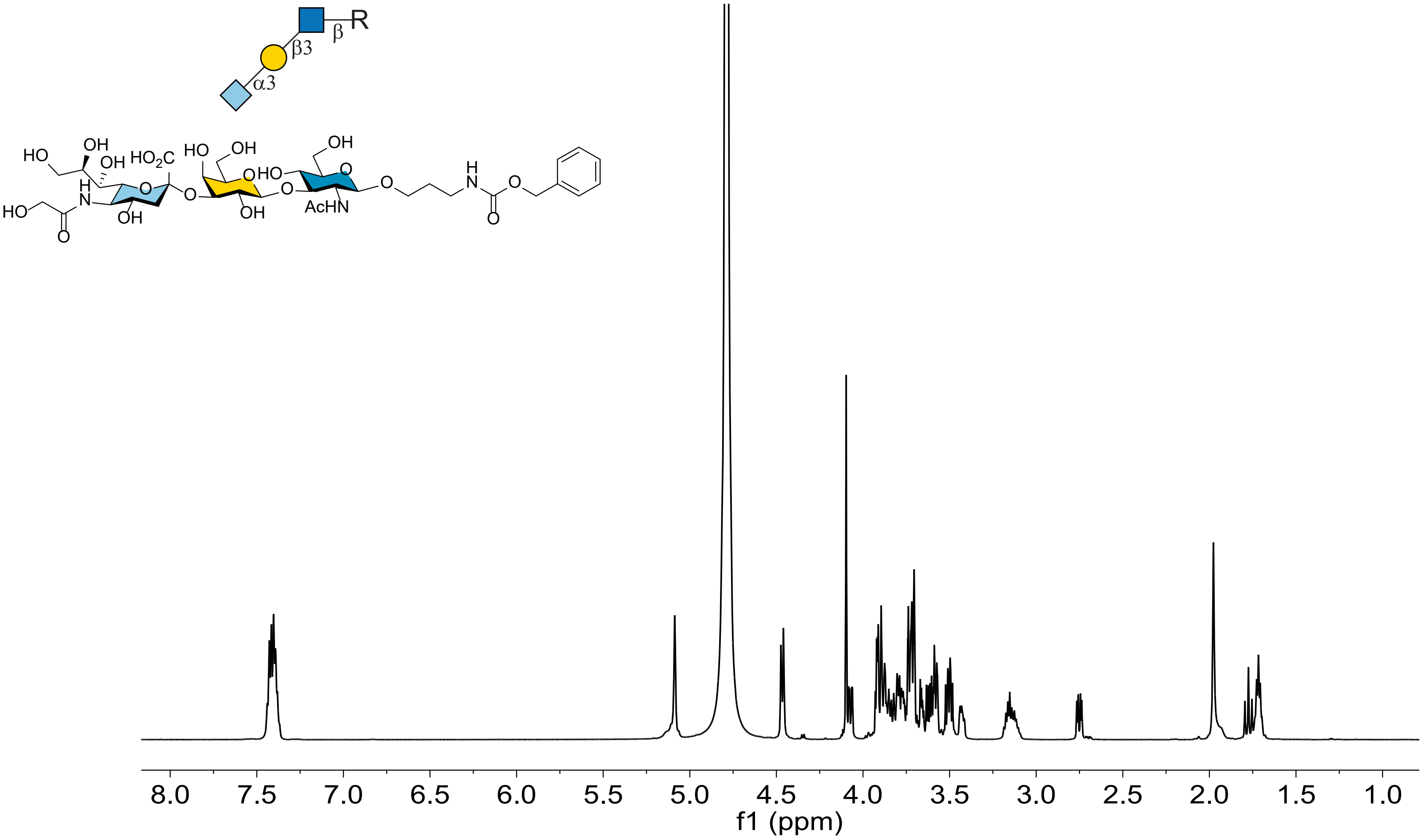

^{1}H NMR of Compound **19**

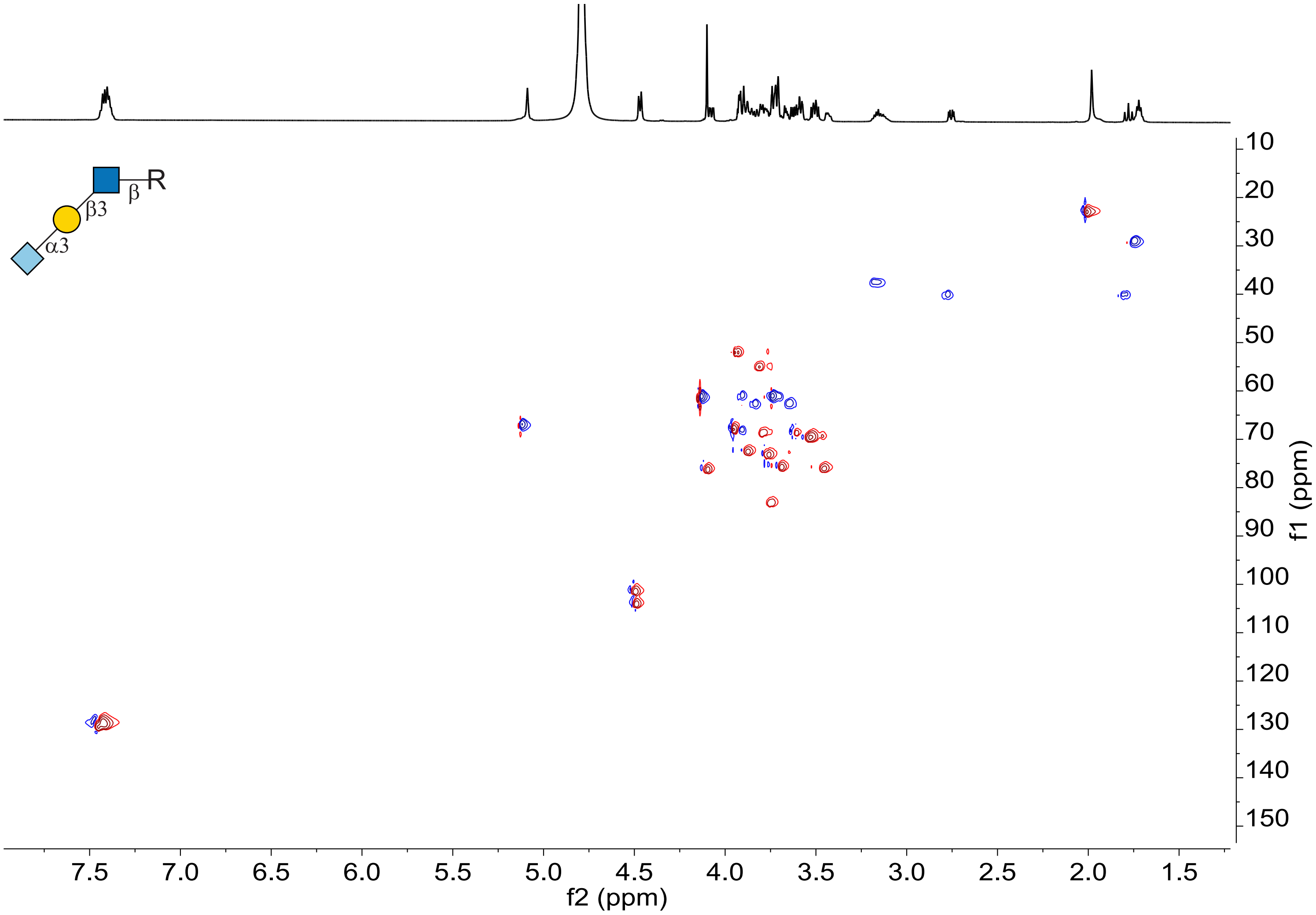

HSQC of Compound **19**

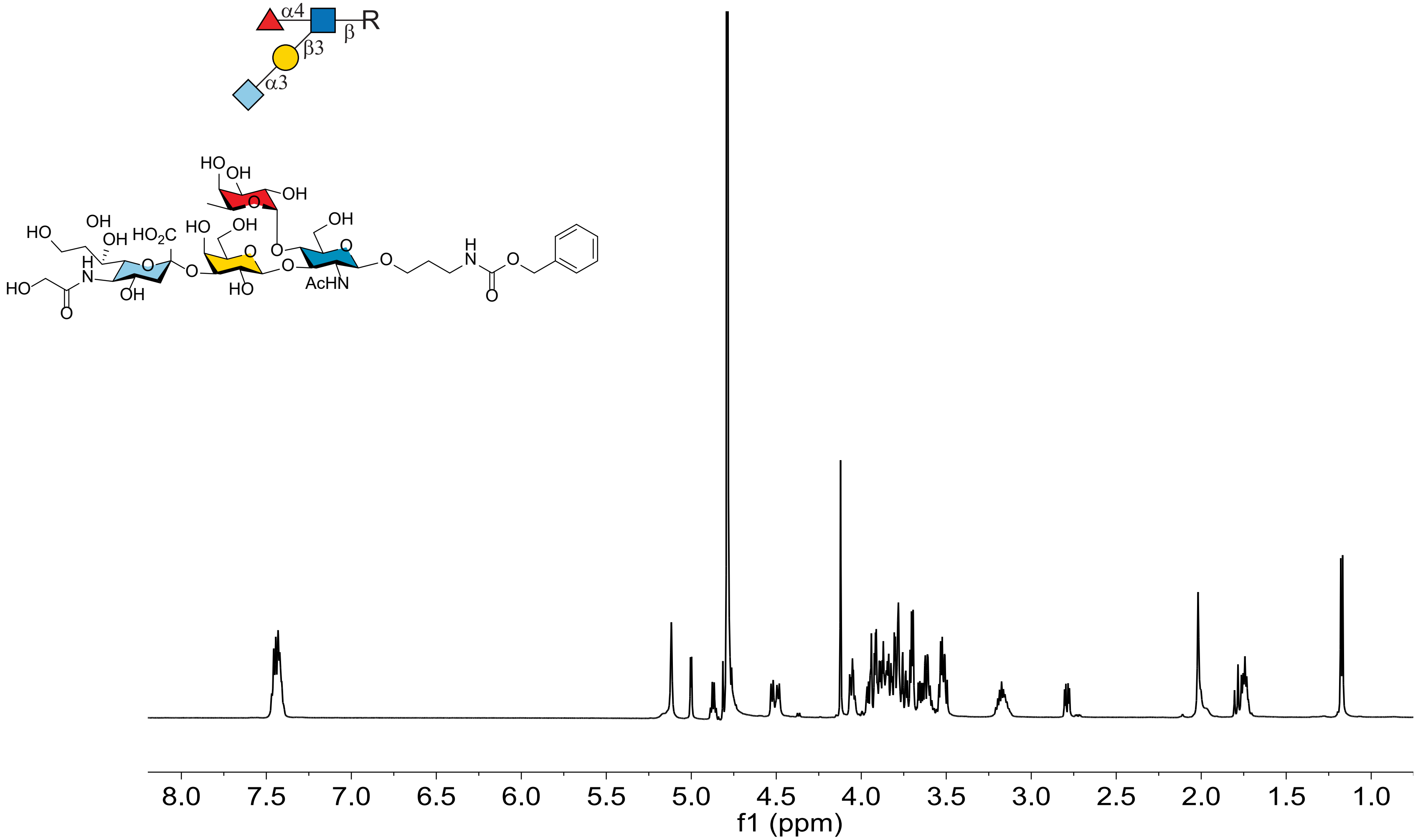

^{1}H NMR of Compound **20**

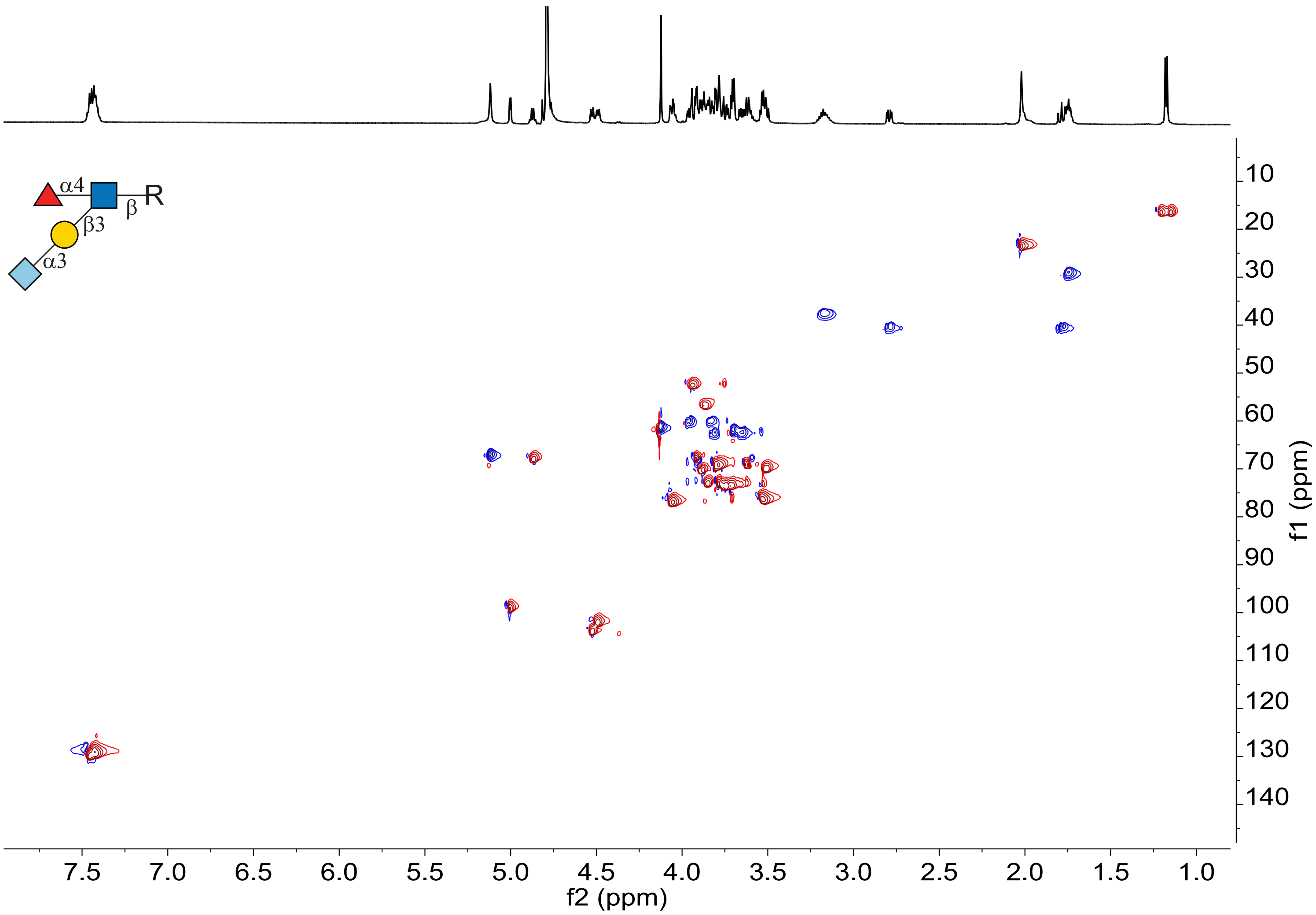

HSQC of Compound **20**

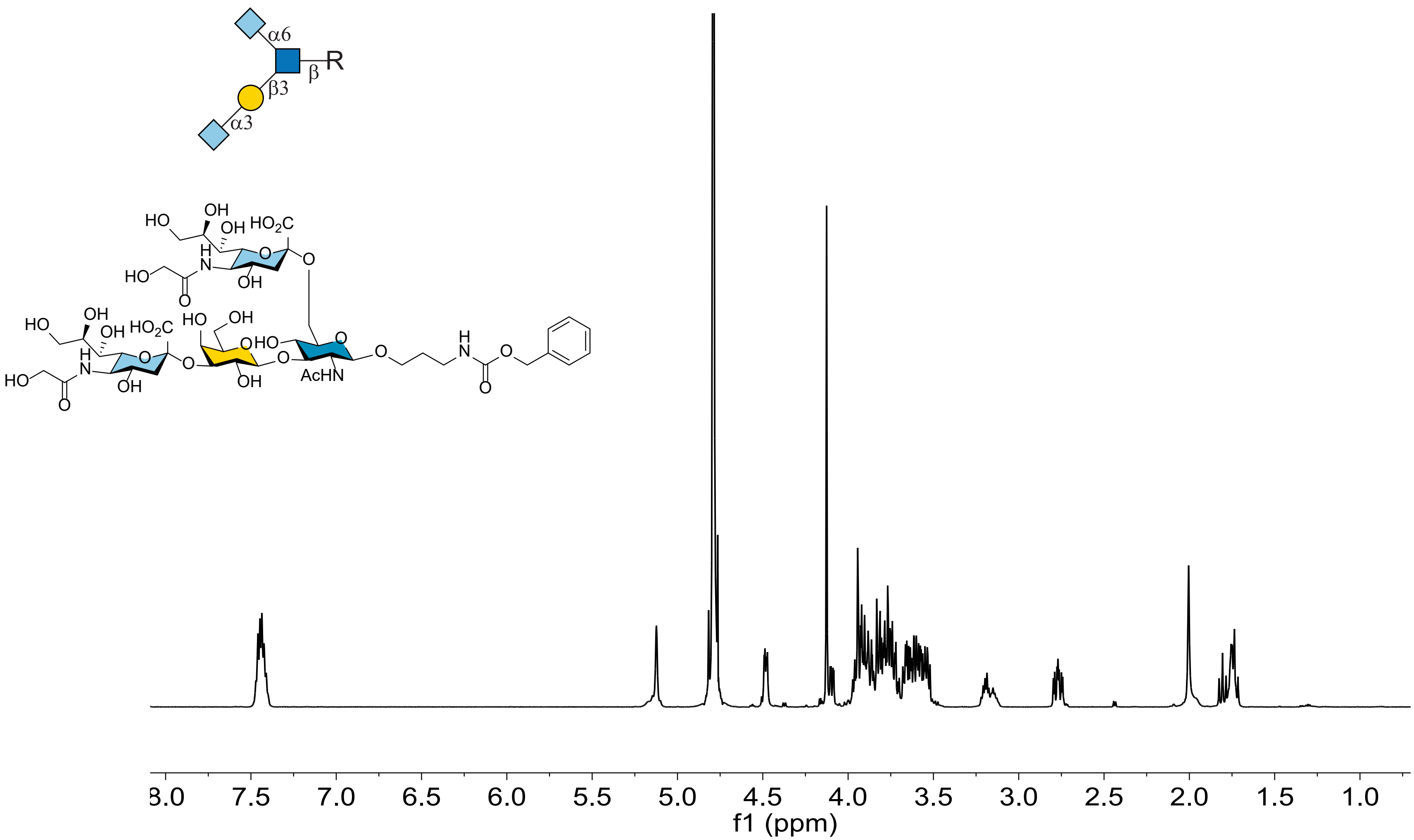

^{1}H NMR of Compound **21**

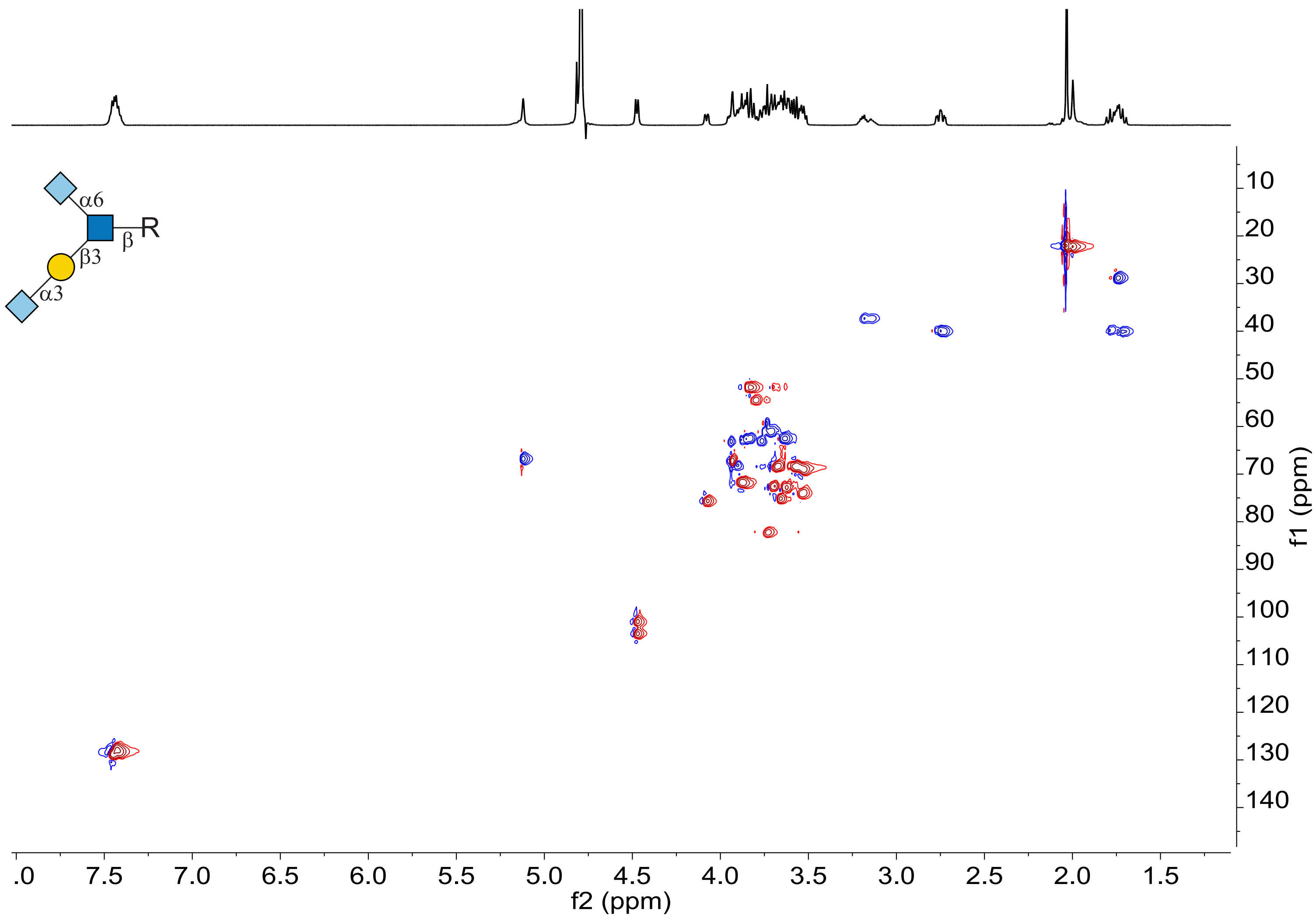

HSQC of Compound **21**

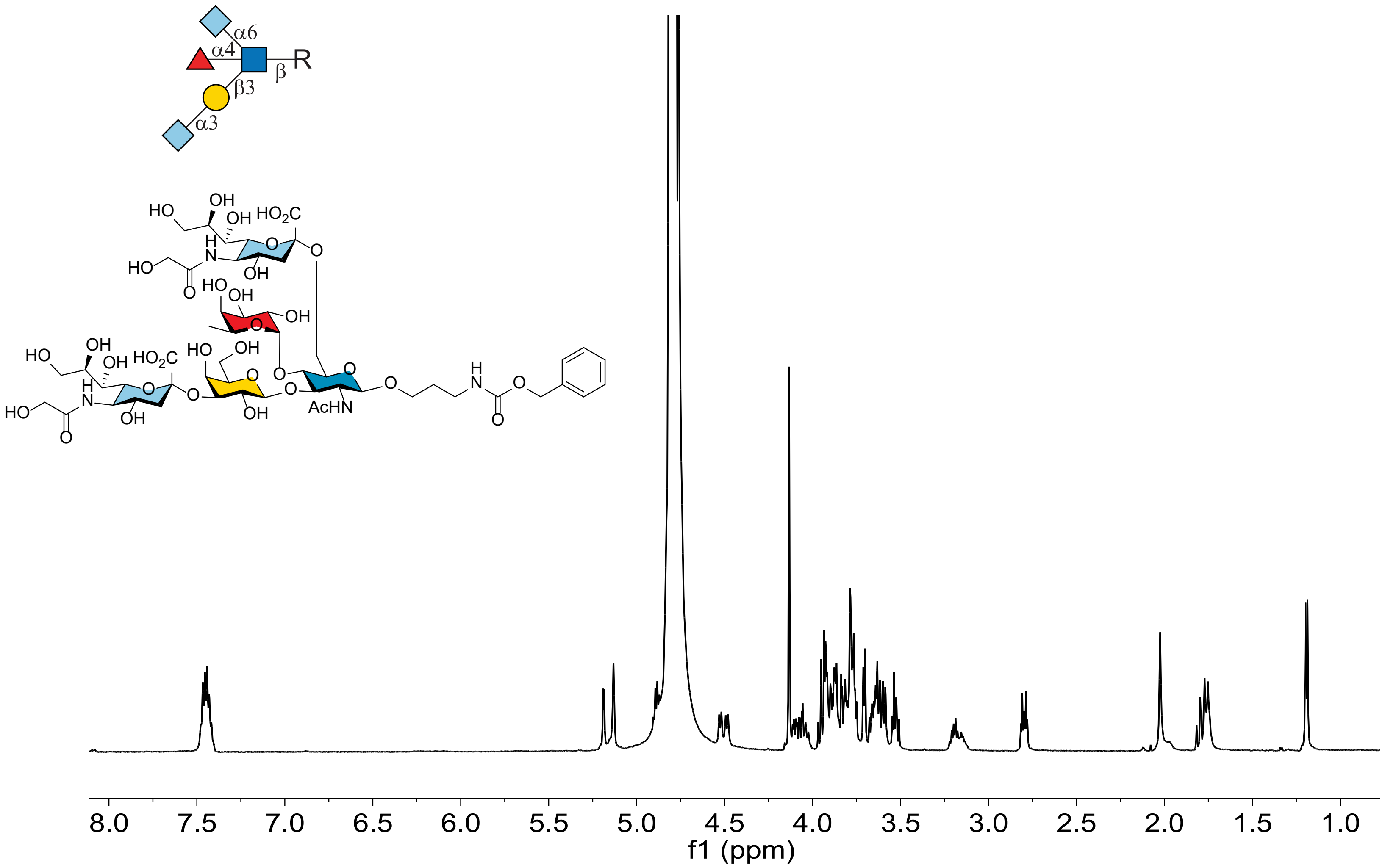

^{1}H NMR of Compound **22**

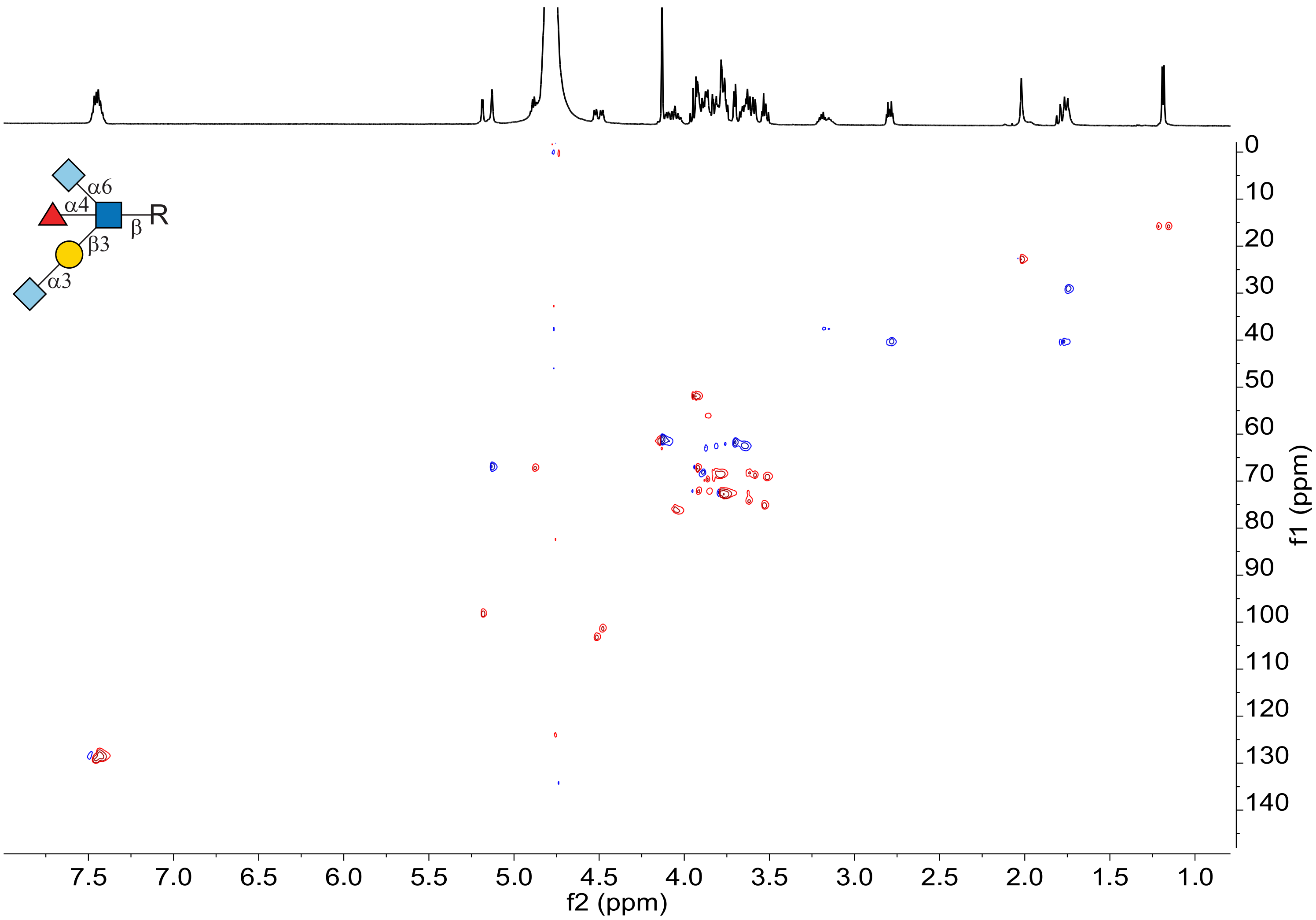

HSQC of Compound **22**

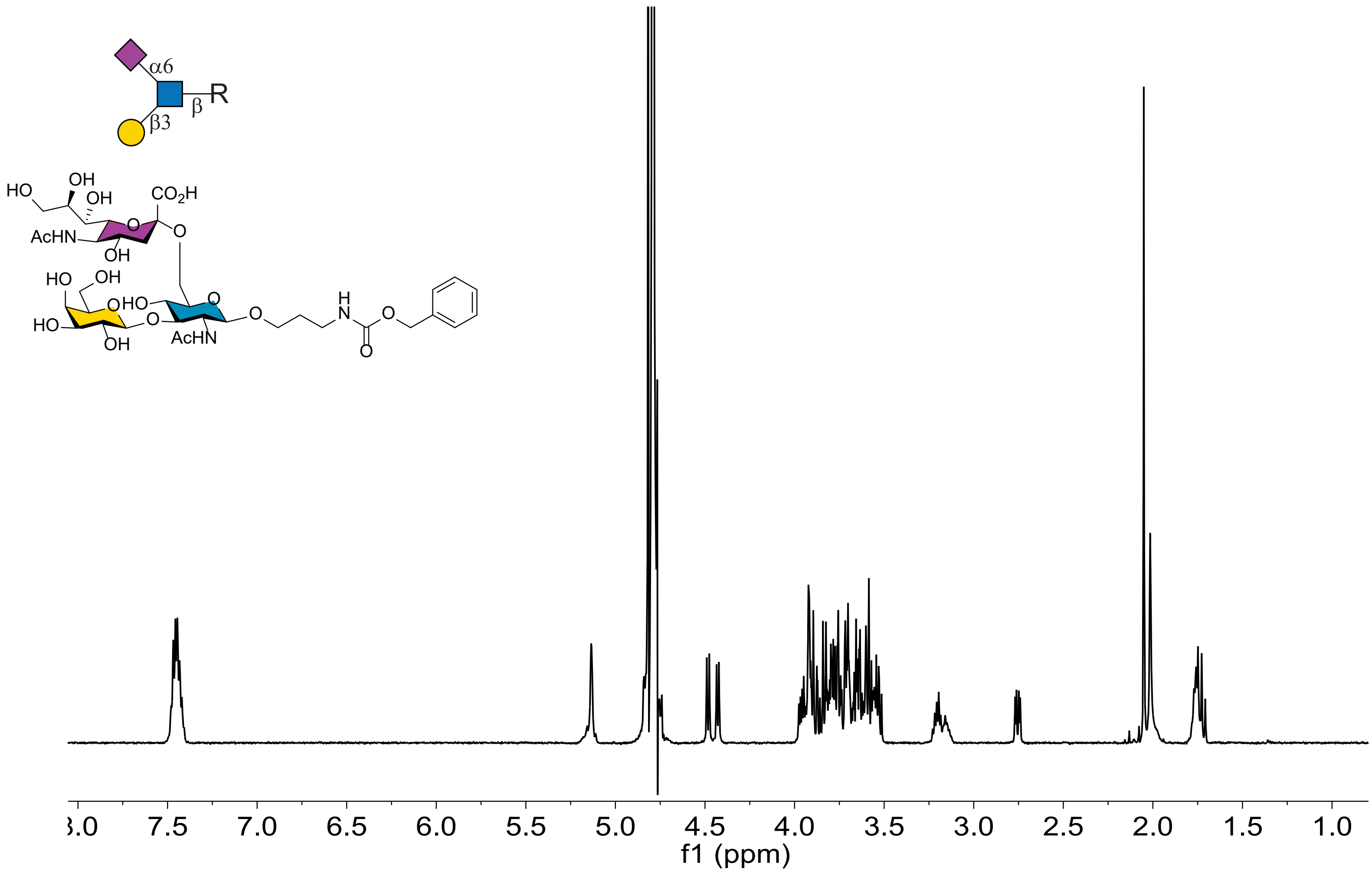

^{1}H NMR of Compound **23**

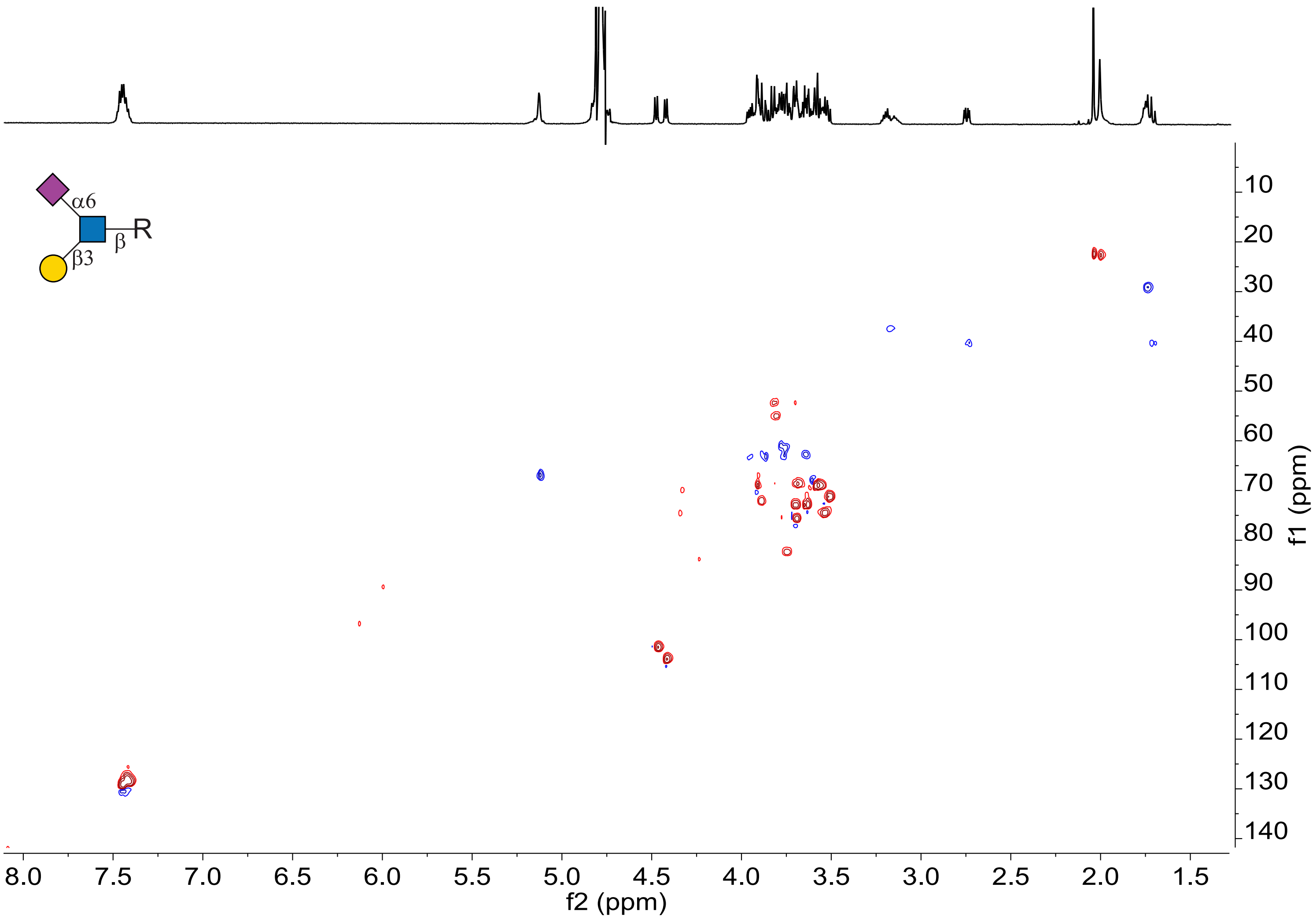

HSQC of Compound **23**

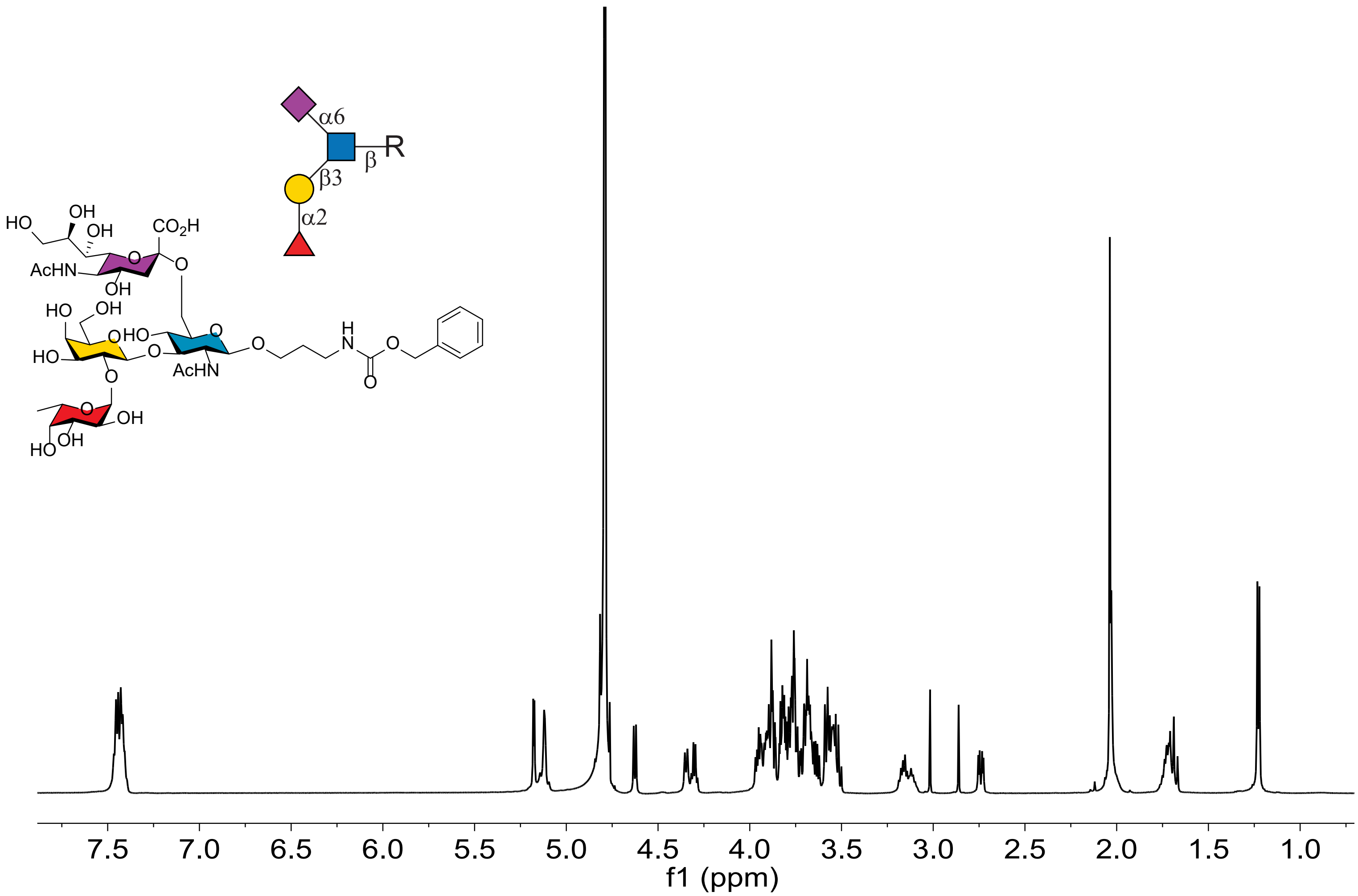

^{1}H NMR of Compound **24**

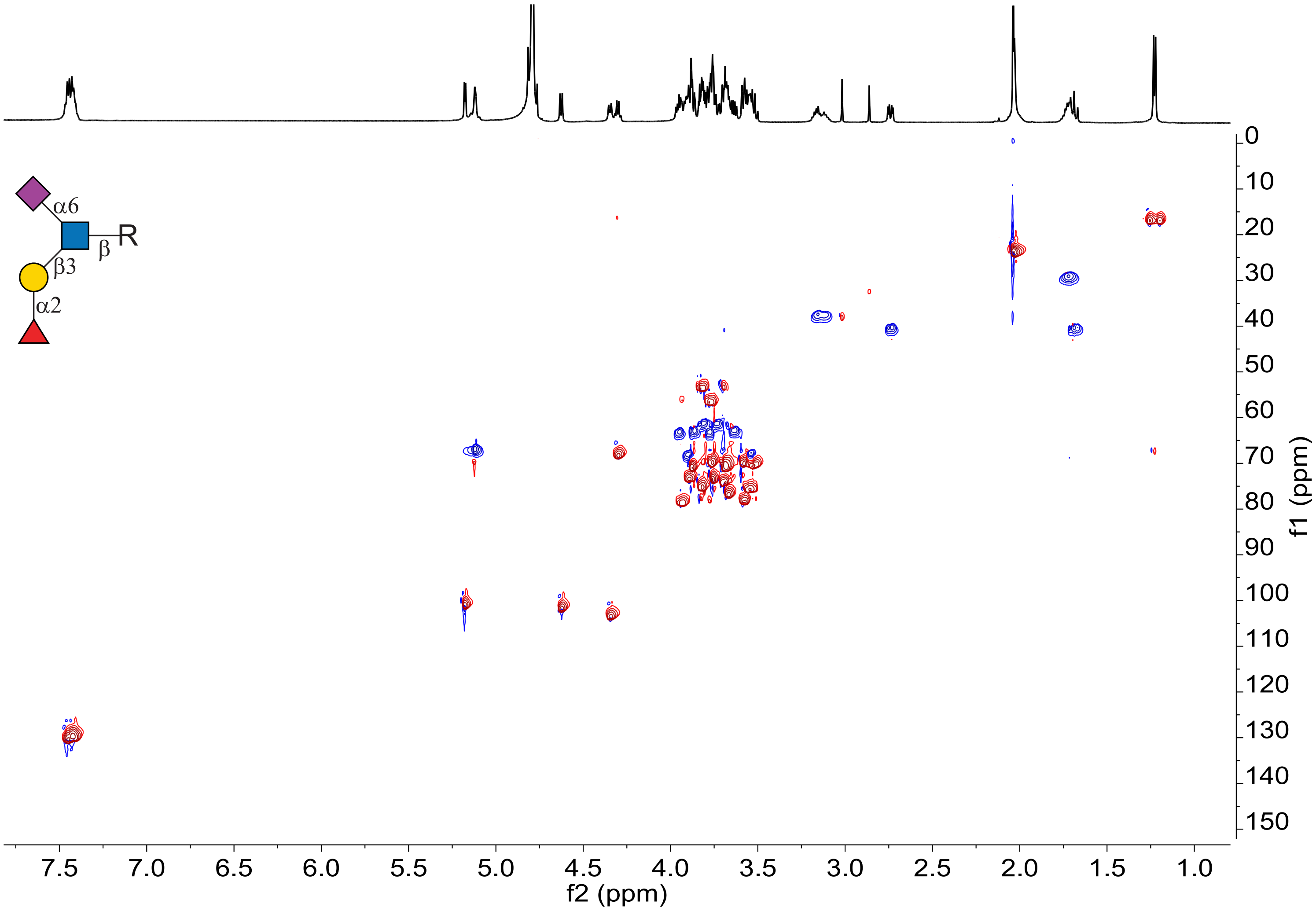

HSQC of Compound **24**

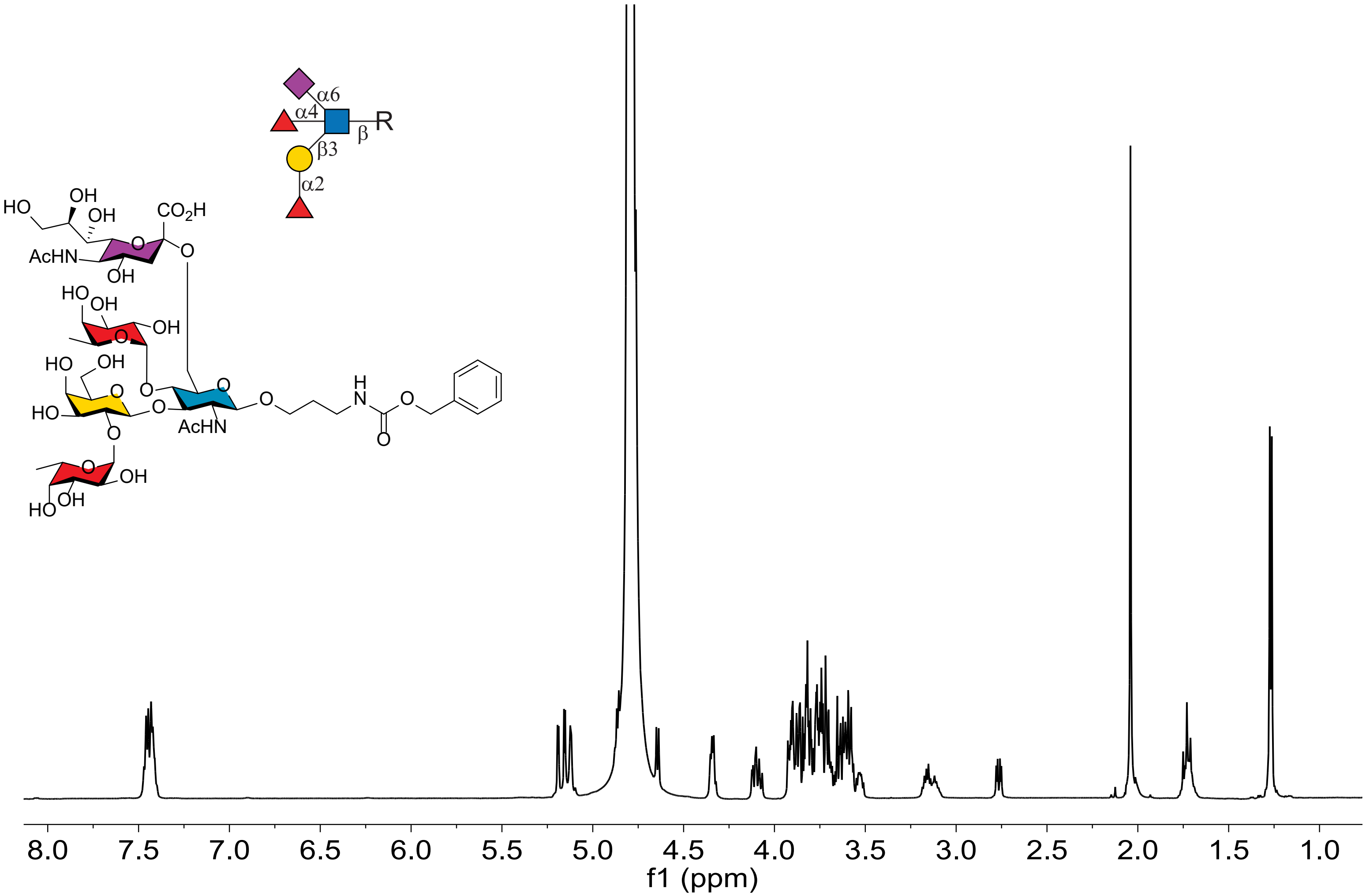

^{1}H NMR of Compound **25**

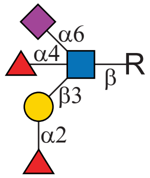

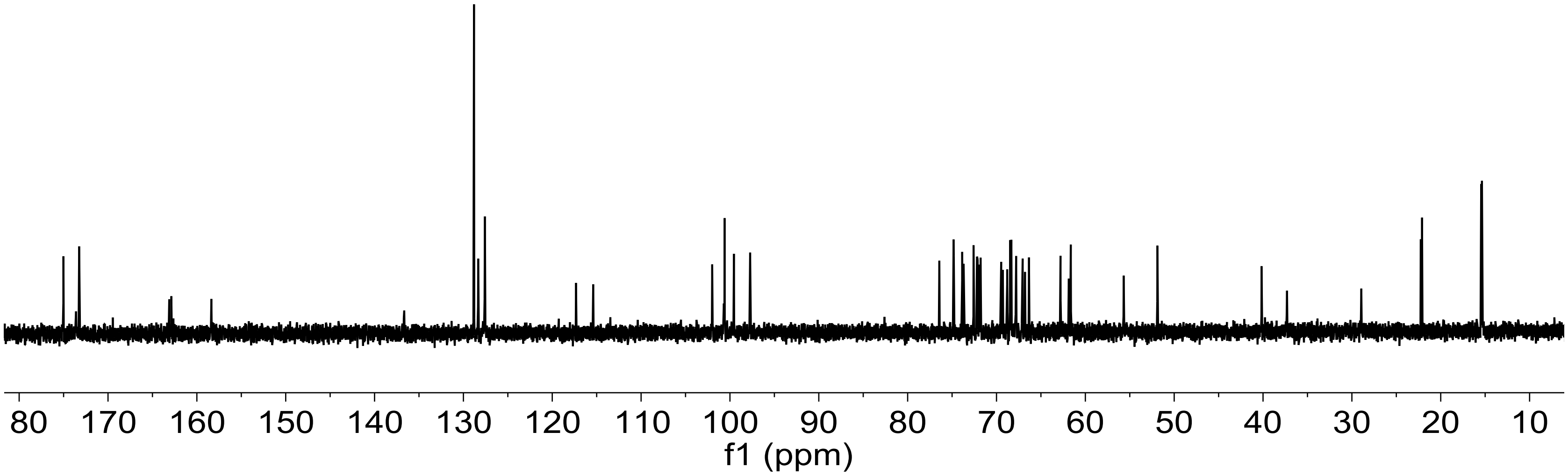

^{13}C NMR of Compound **25**

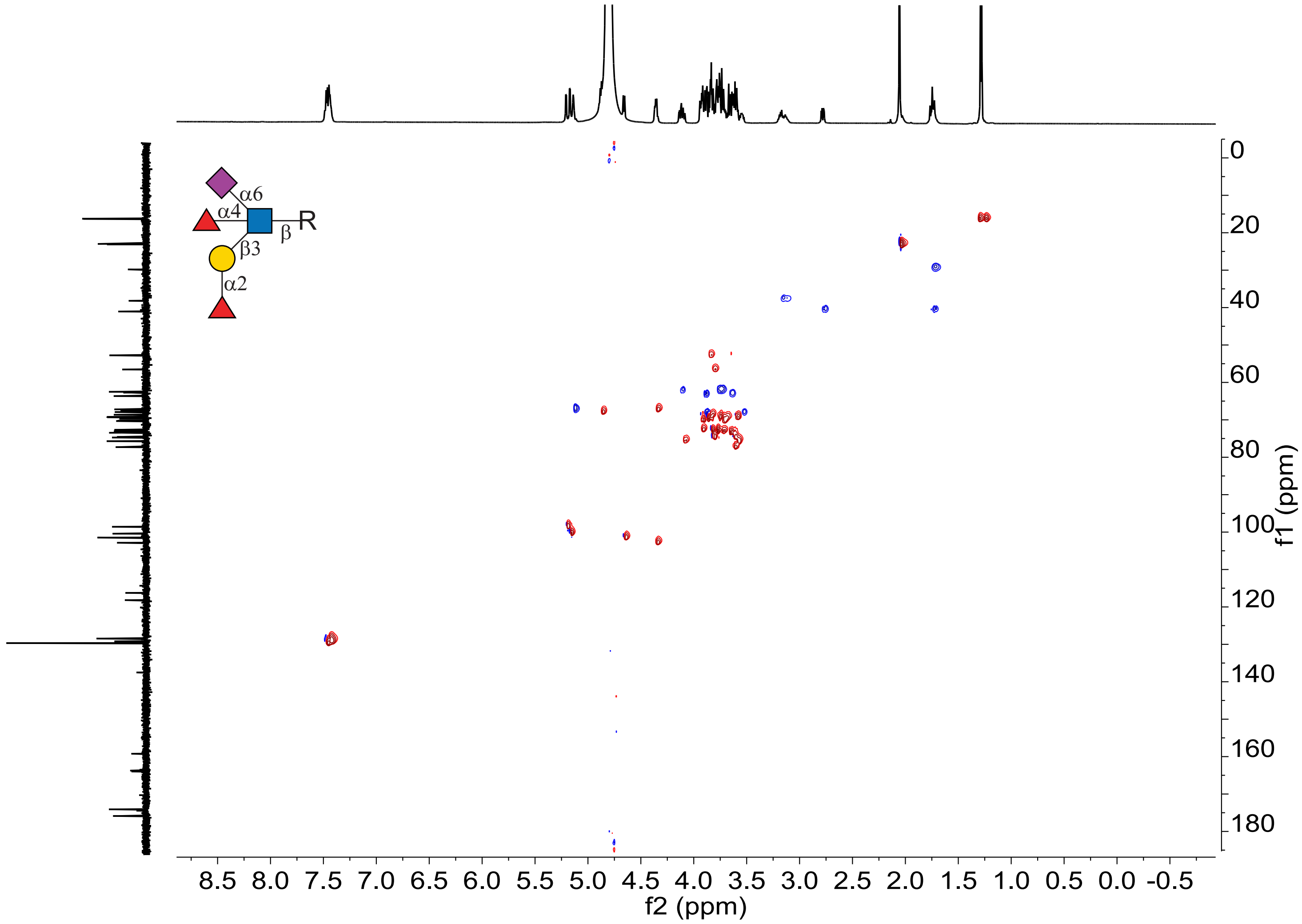

HSQC of Compound **25**

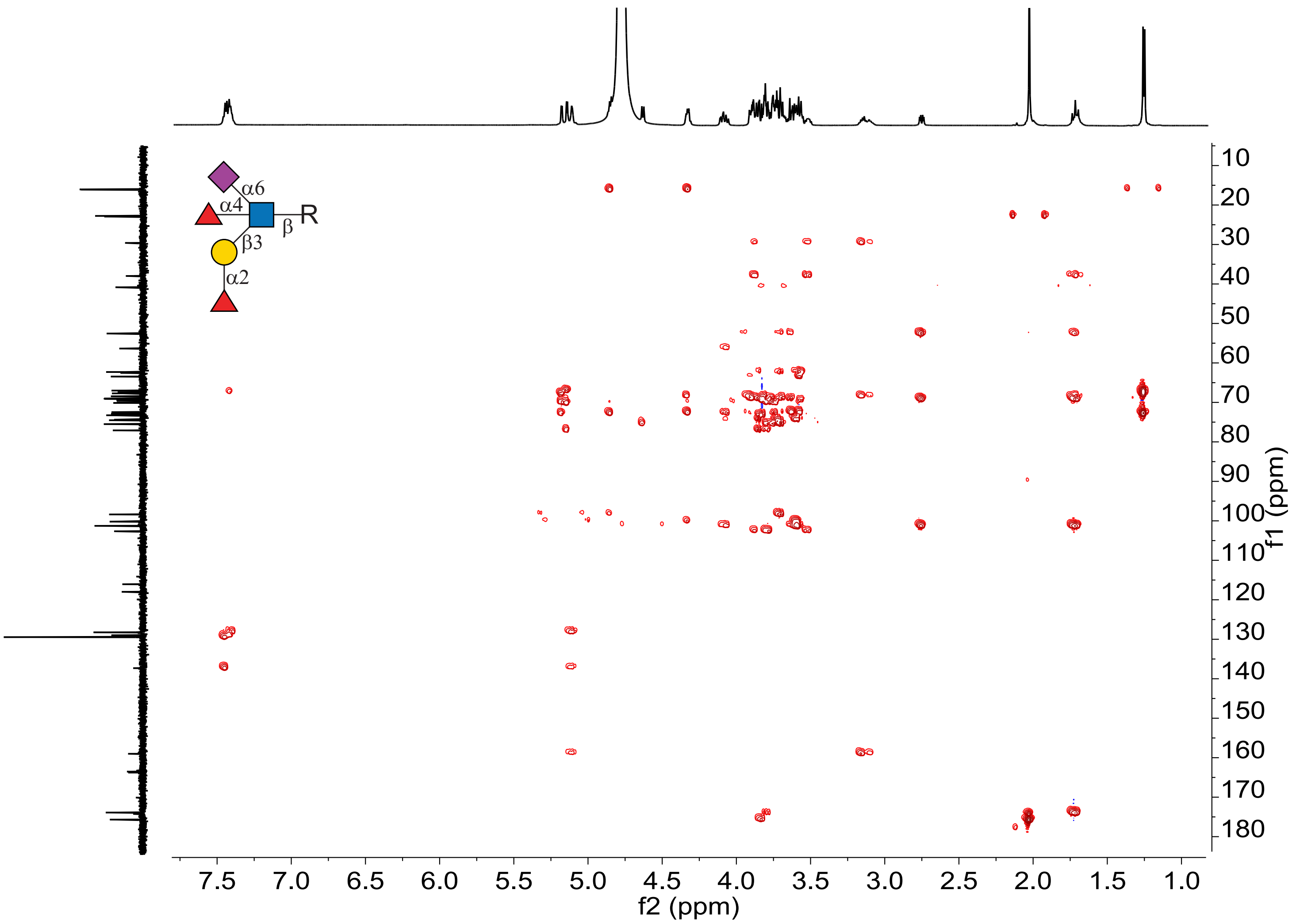

HMBC of Compound **25**

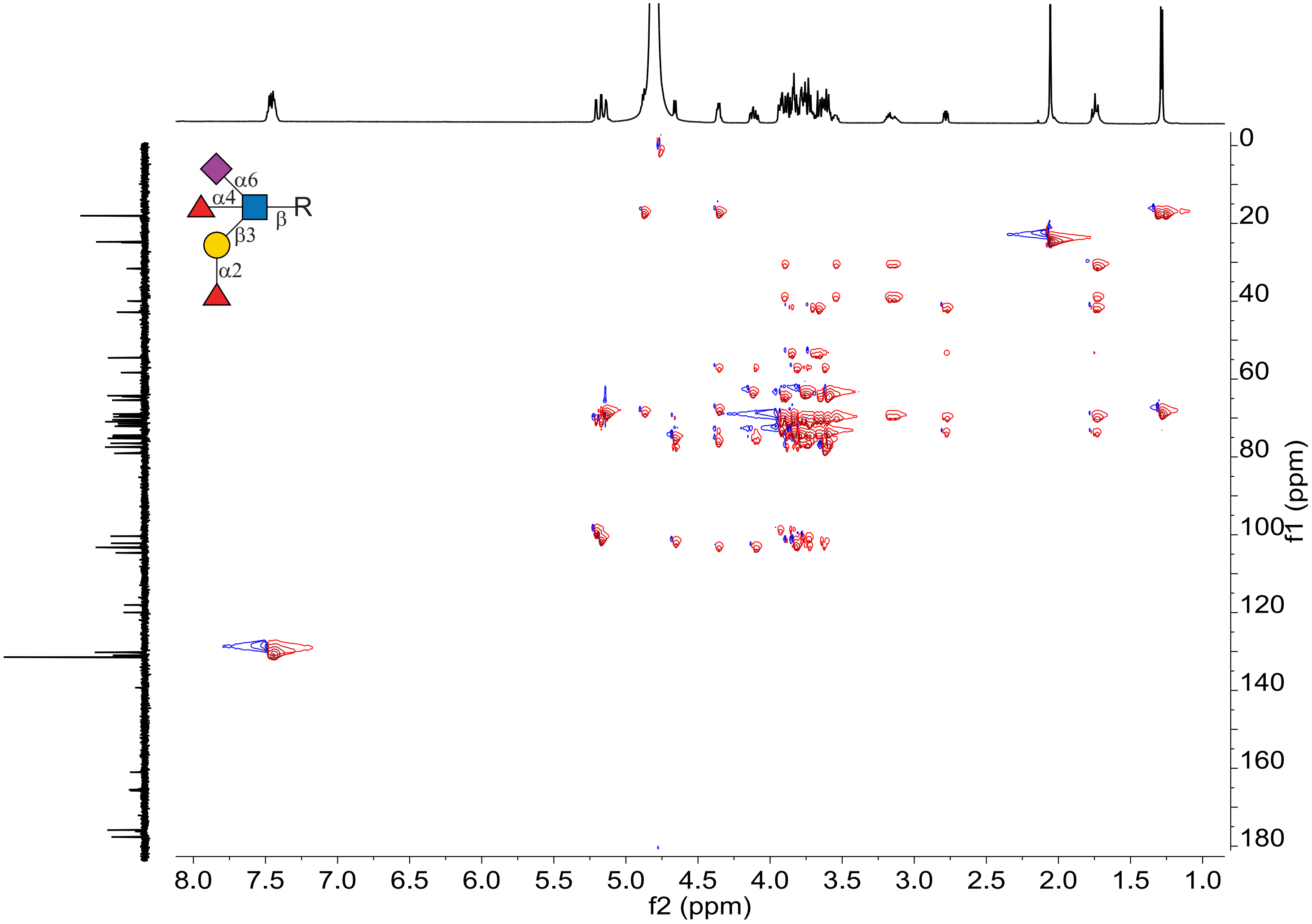

HSQC-TOCSY of Compound **25**

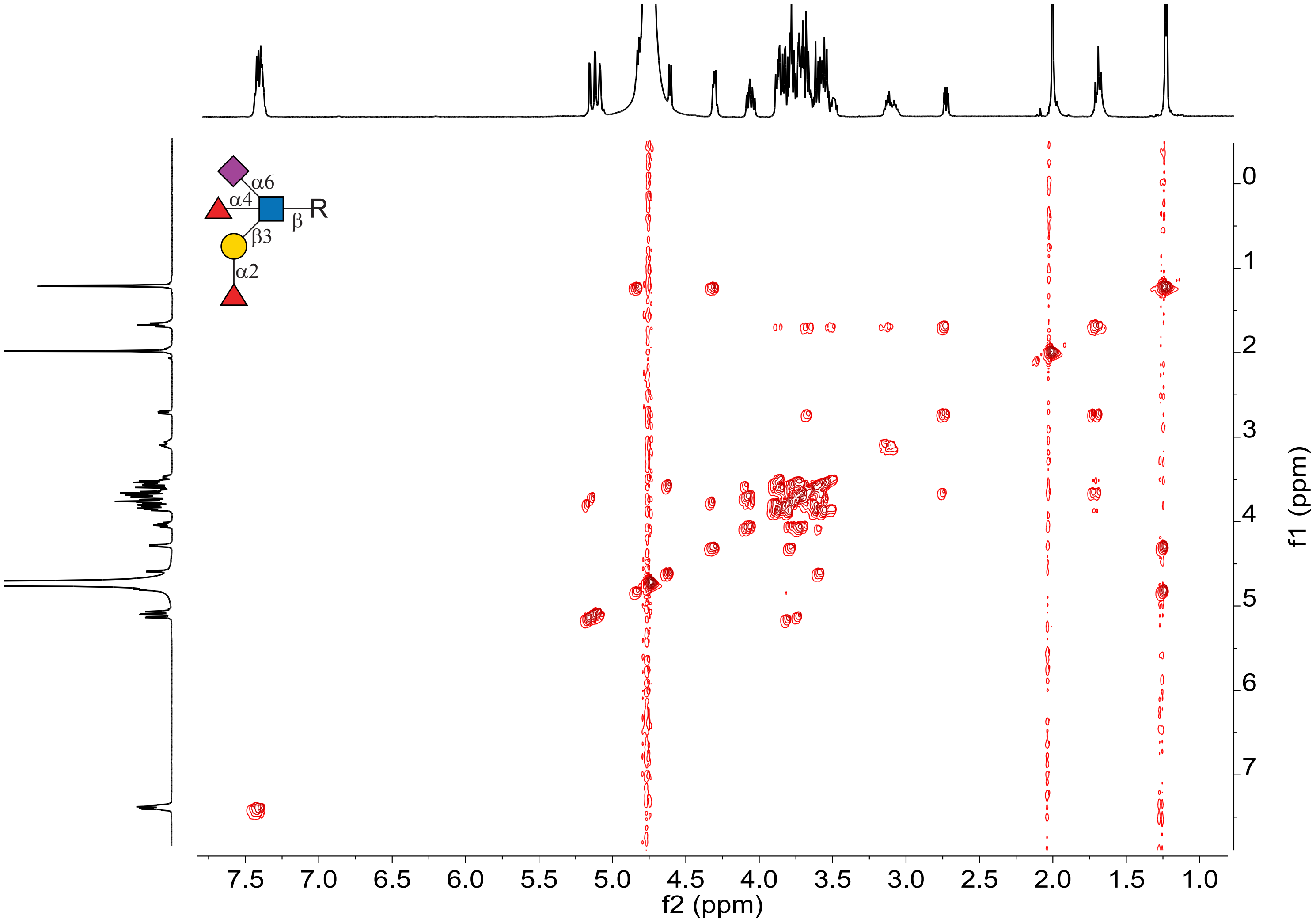

^{1}H-^{1}H COSY of Compound **25**

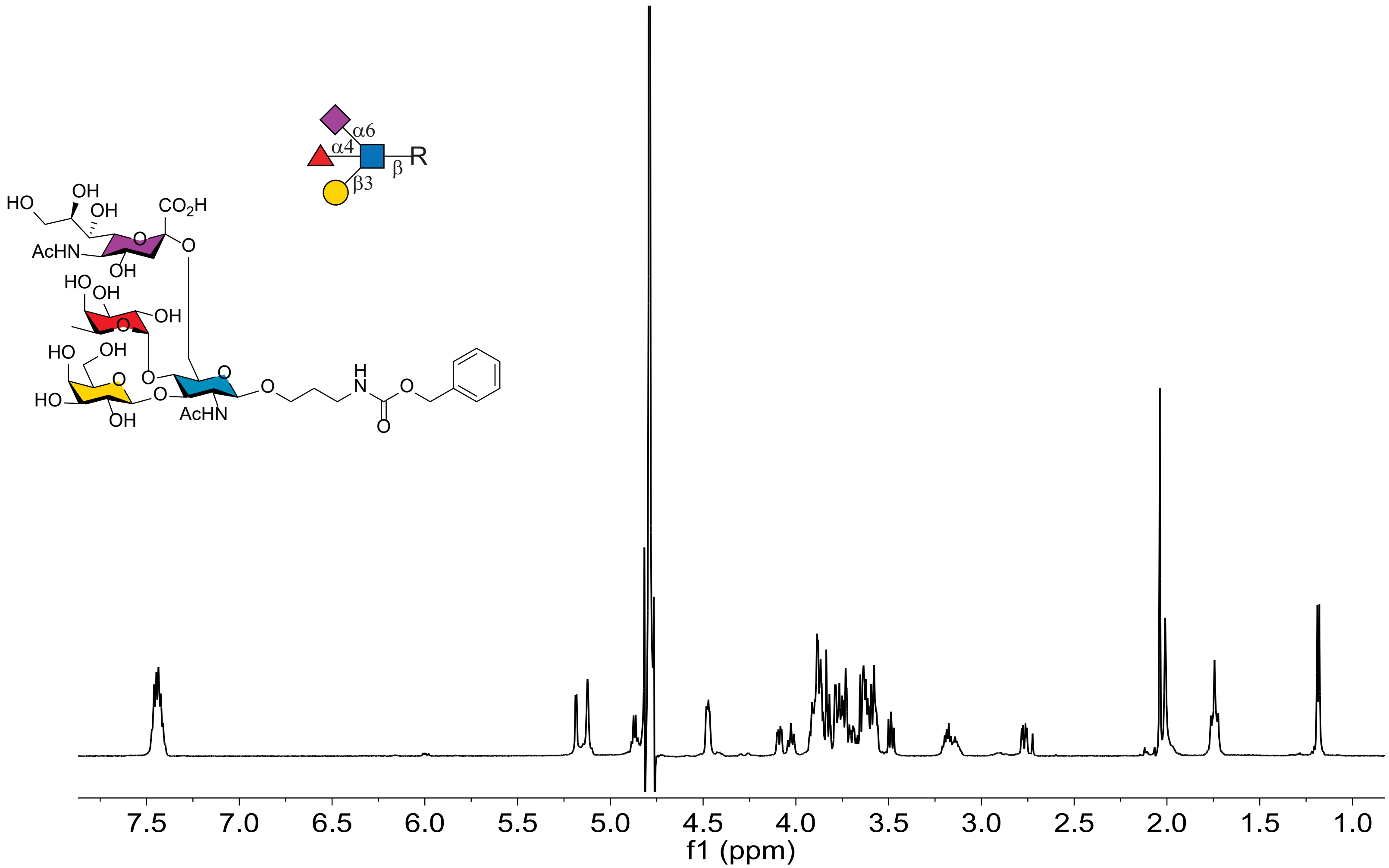

^{1}H NMR of Compound **26**

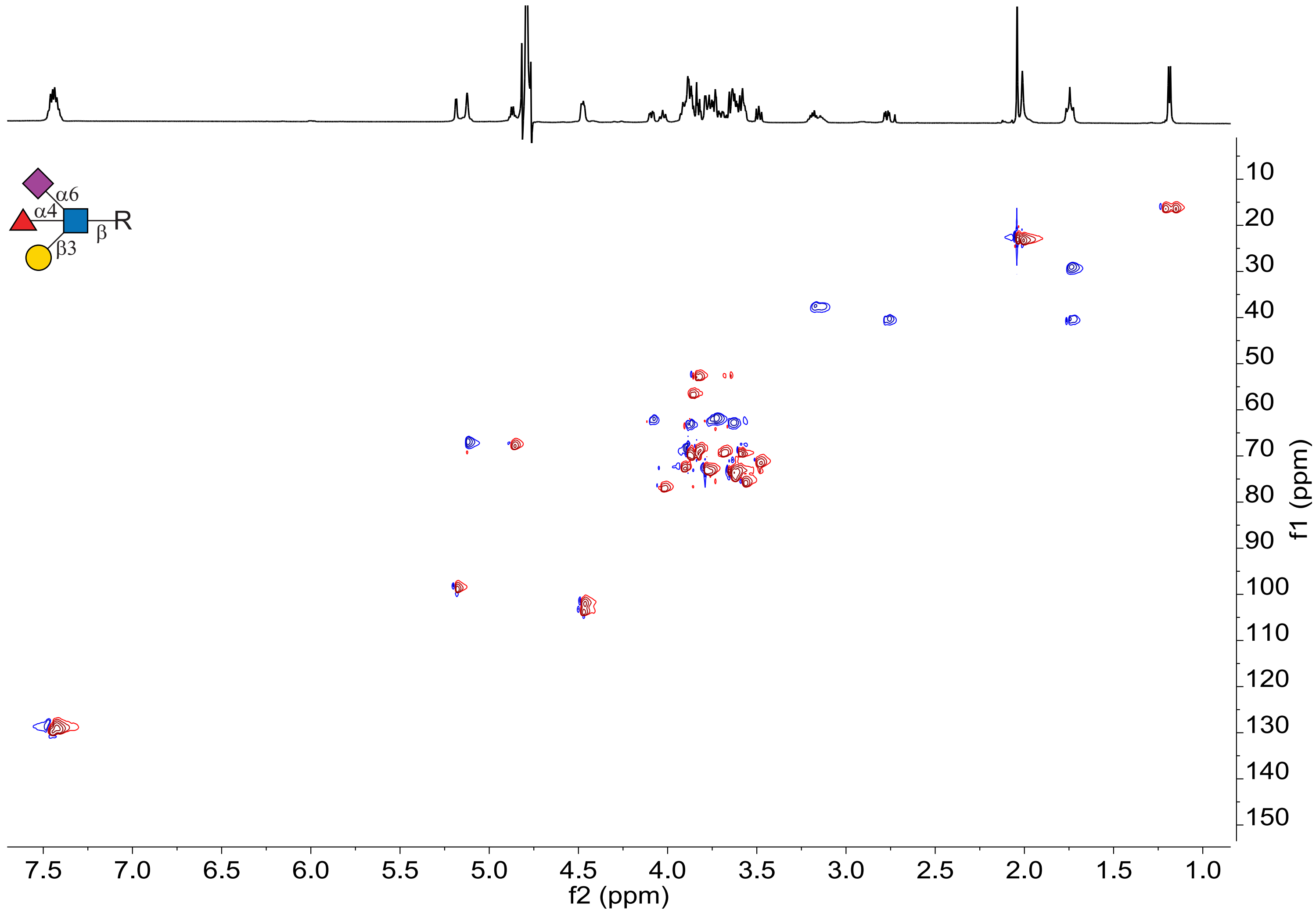

HSQC of Compound **26**

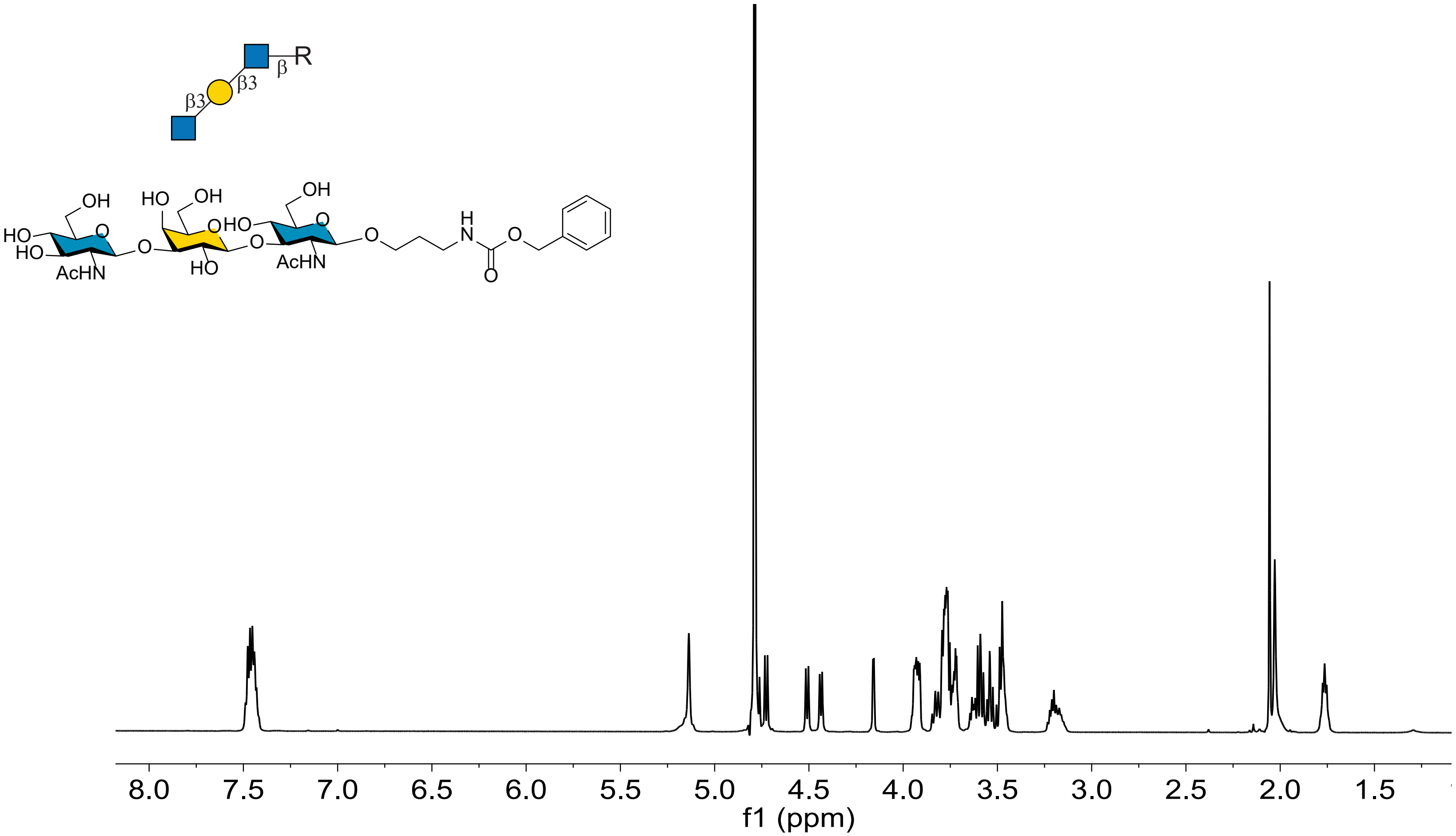

^{1}H NMR of Compound **27**

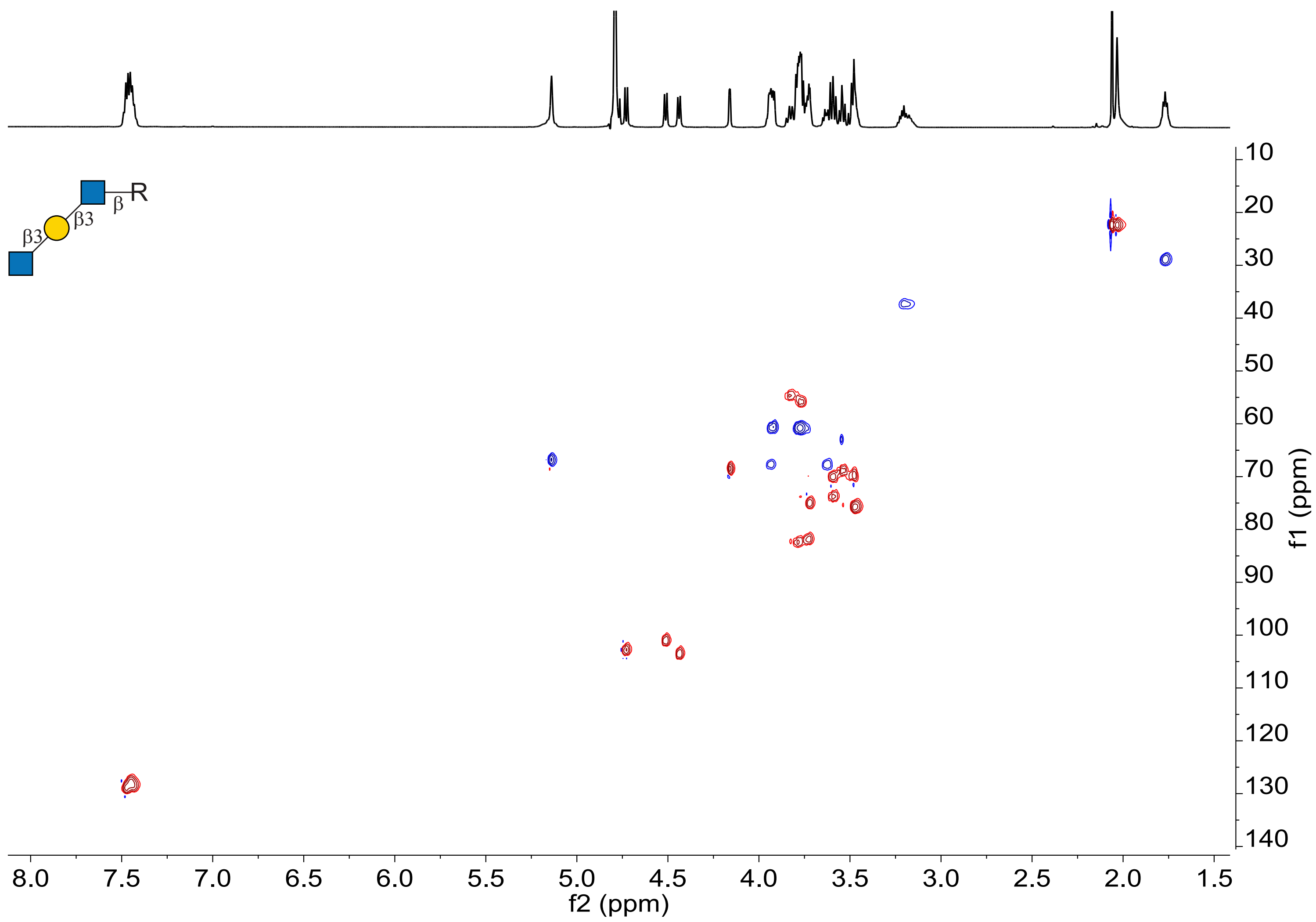

HSQC of Compound **27**

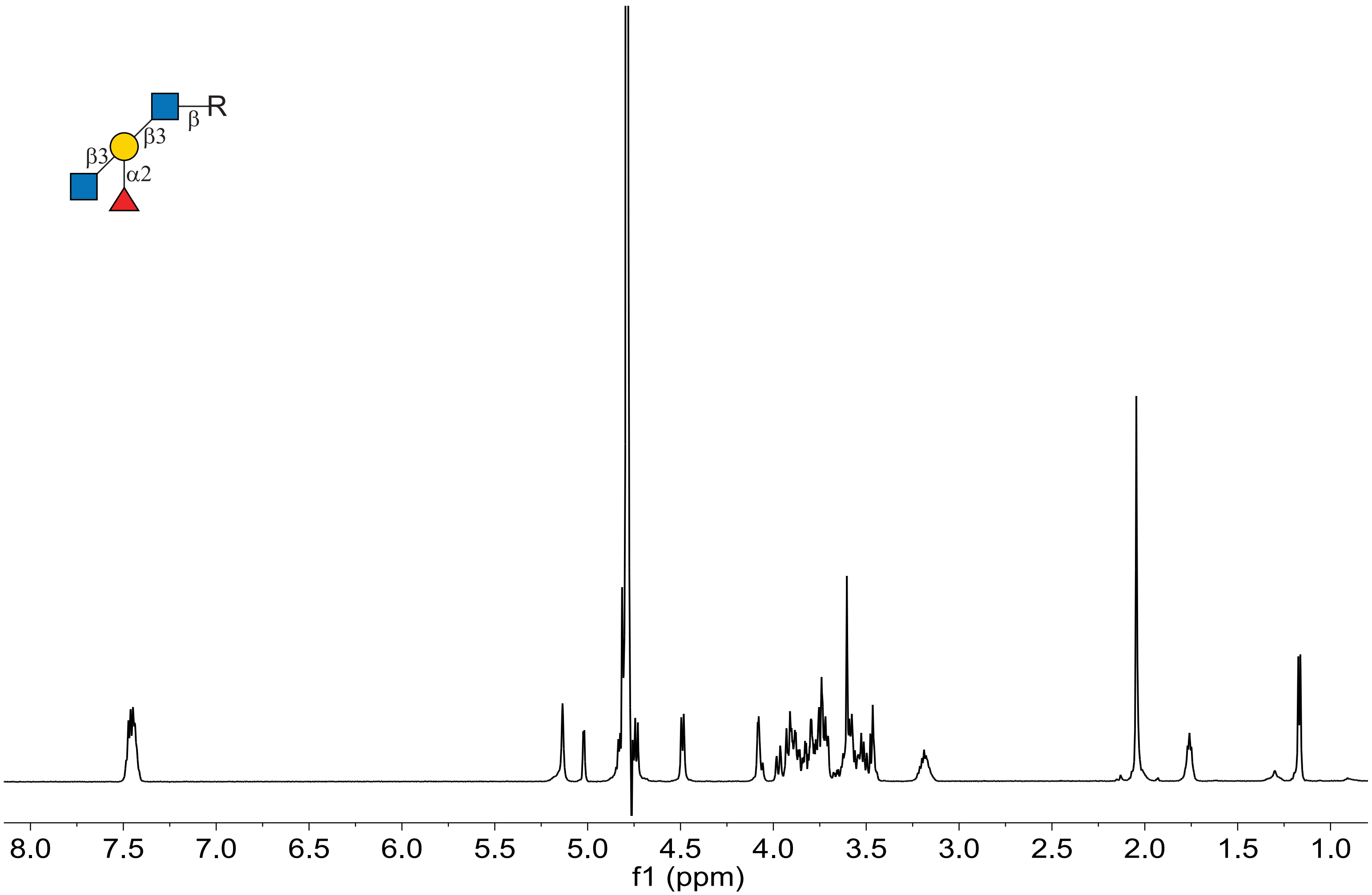

^{1}H NMR of Compound **28**

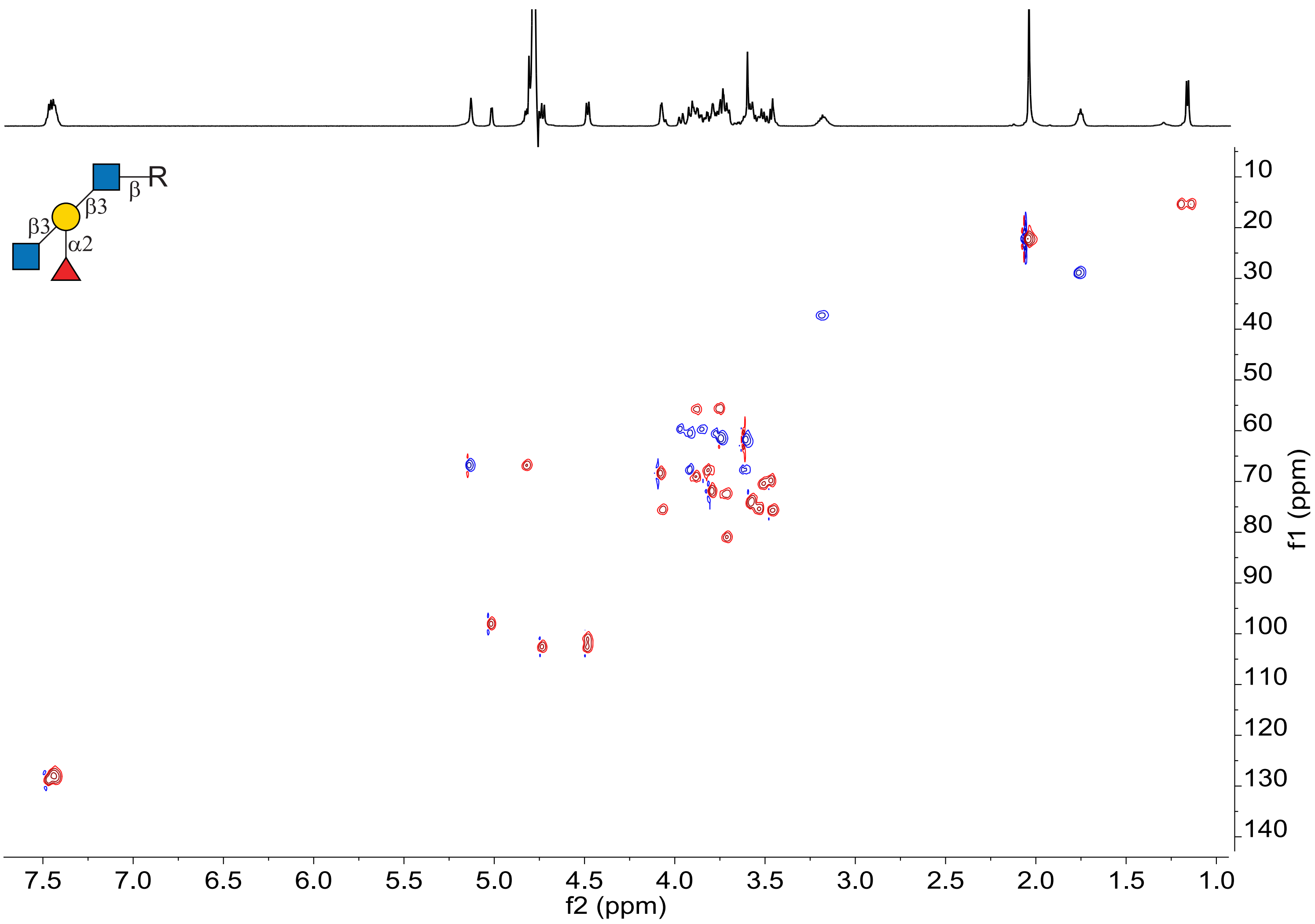

HSQC of Compound **28**

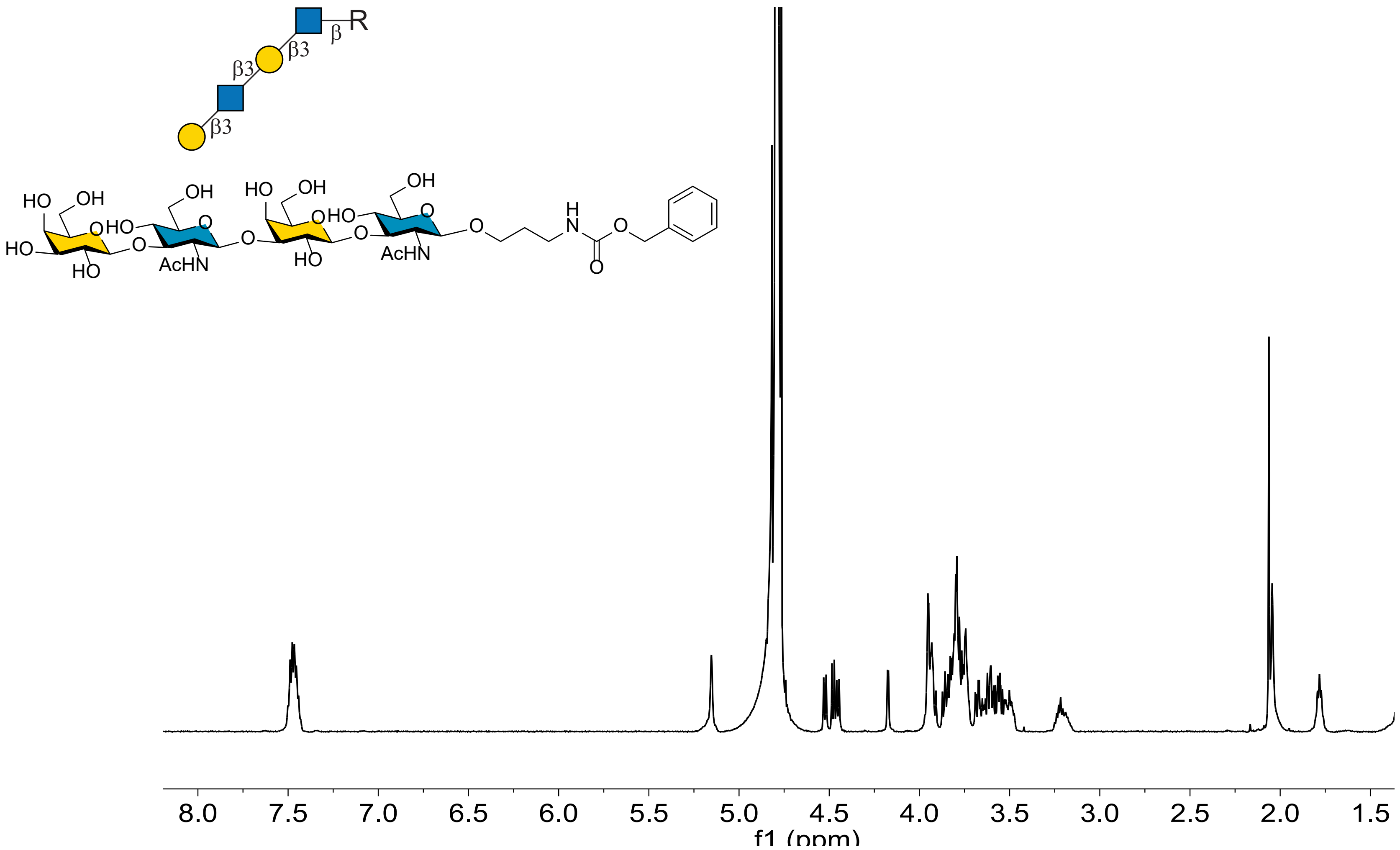

^{1}H NMR of Compound **29**

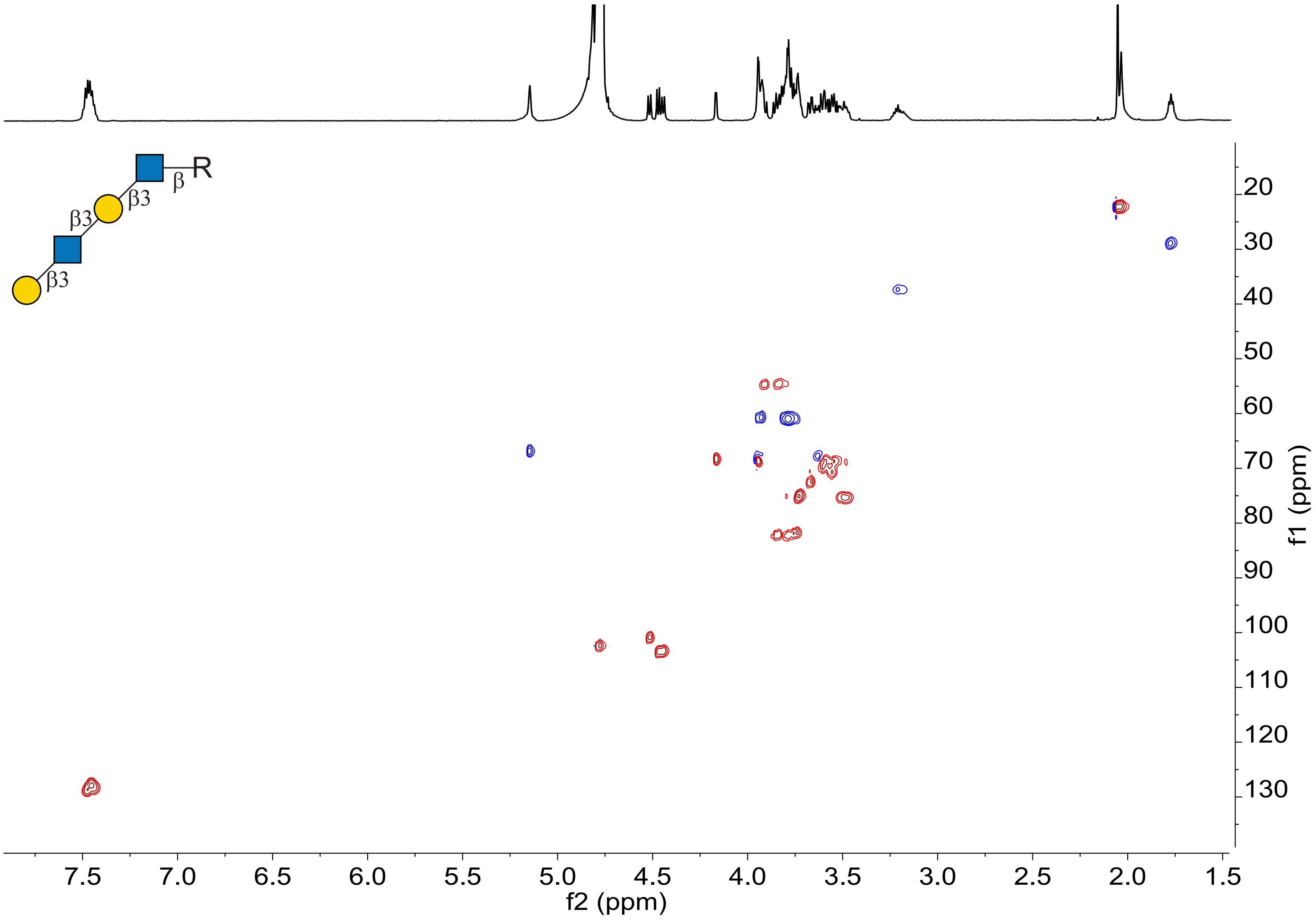

HSQC of Compound **29**

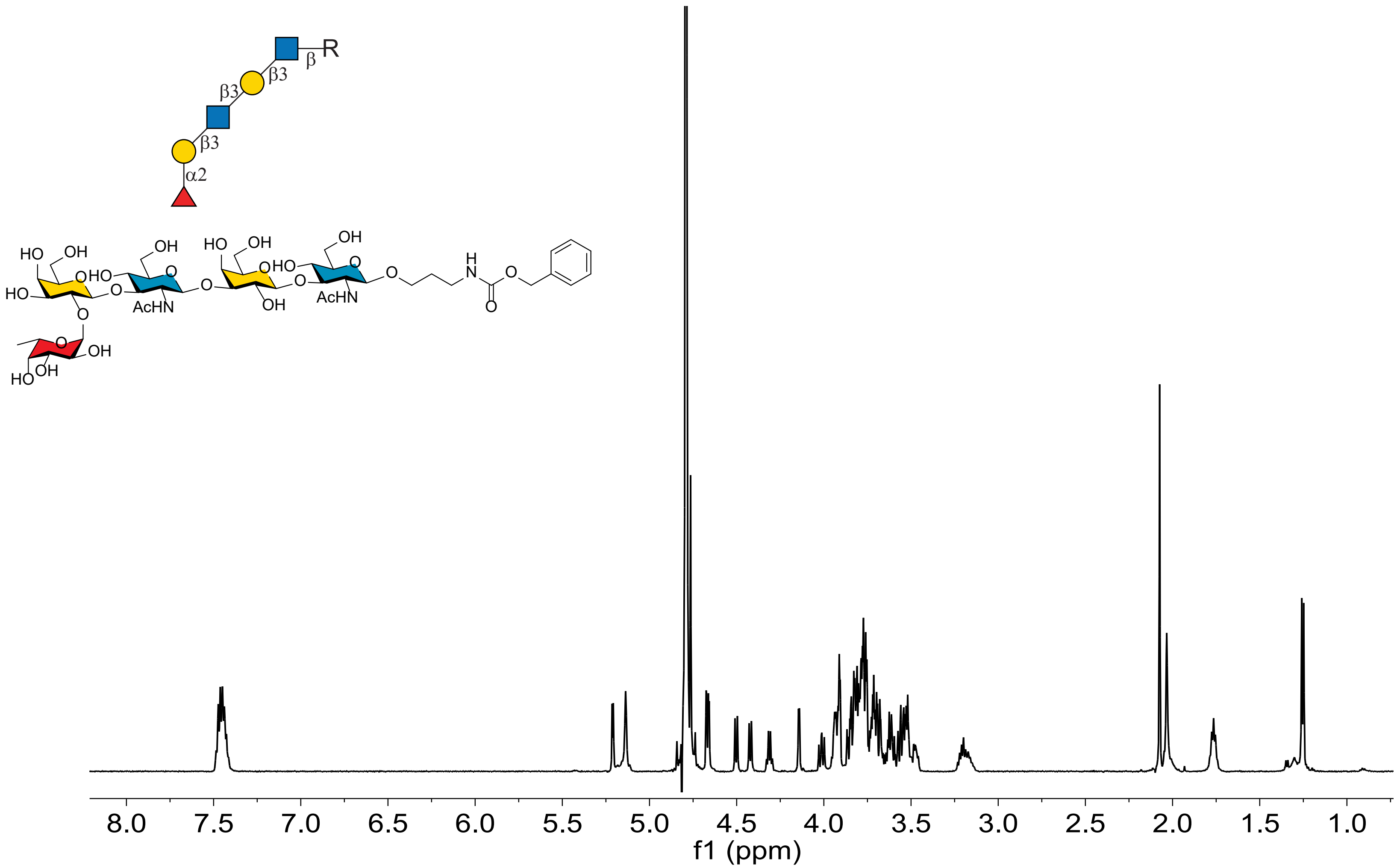

^{1}H NMR of Compound **30**

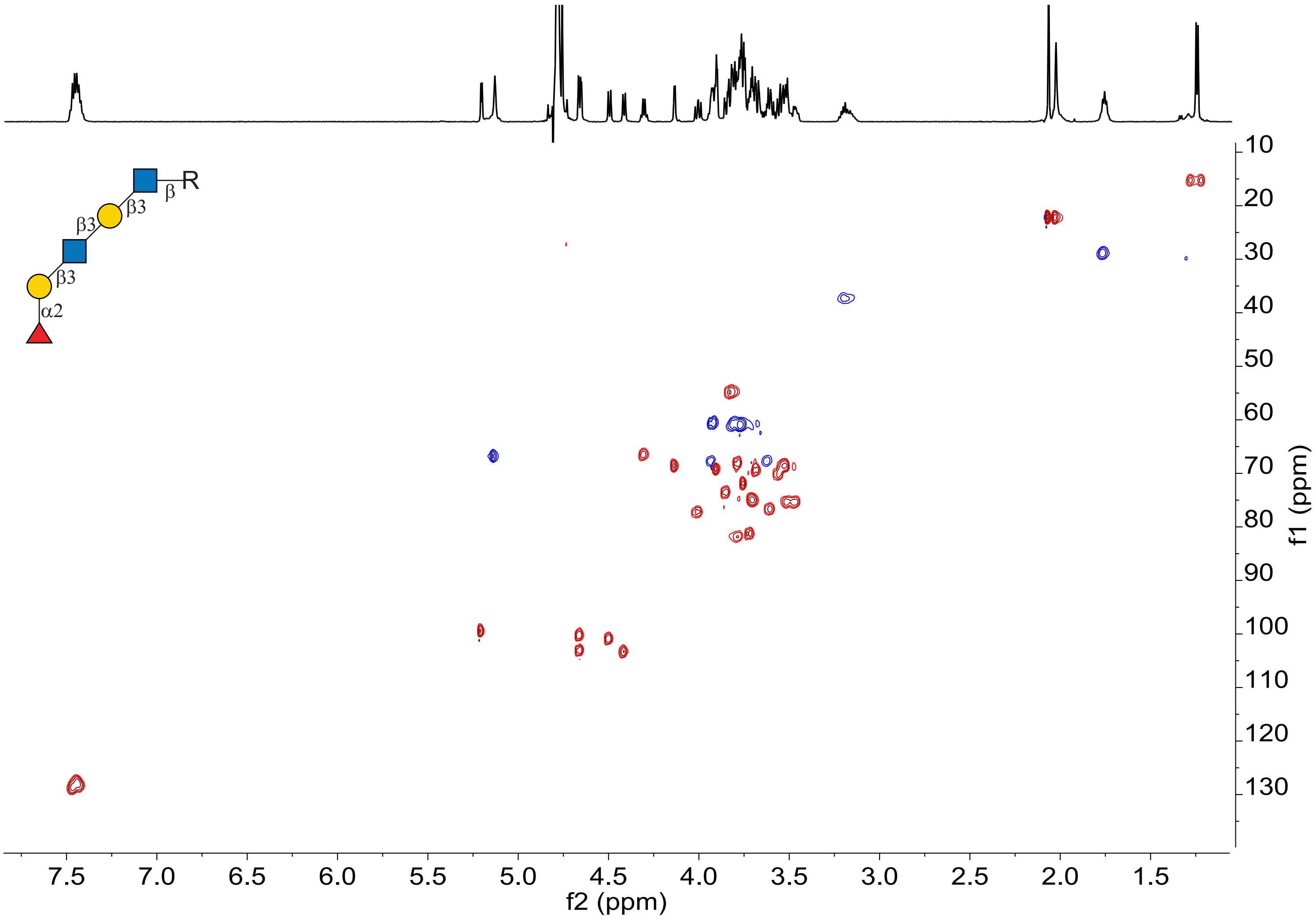

HSQC of Compound **30**

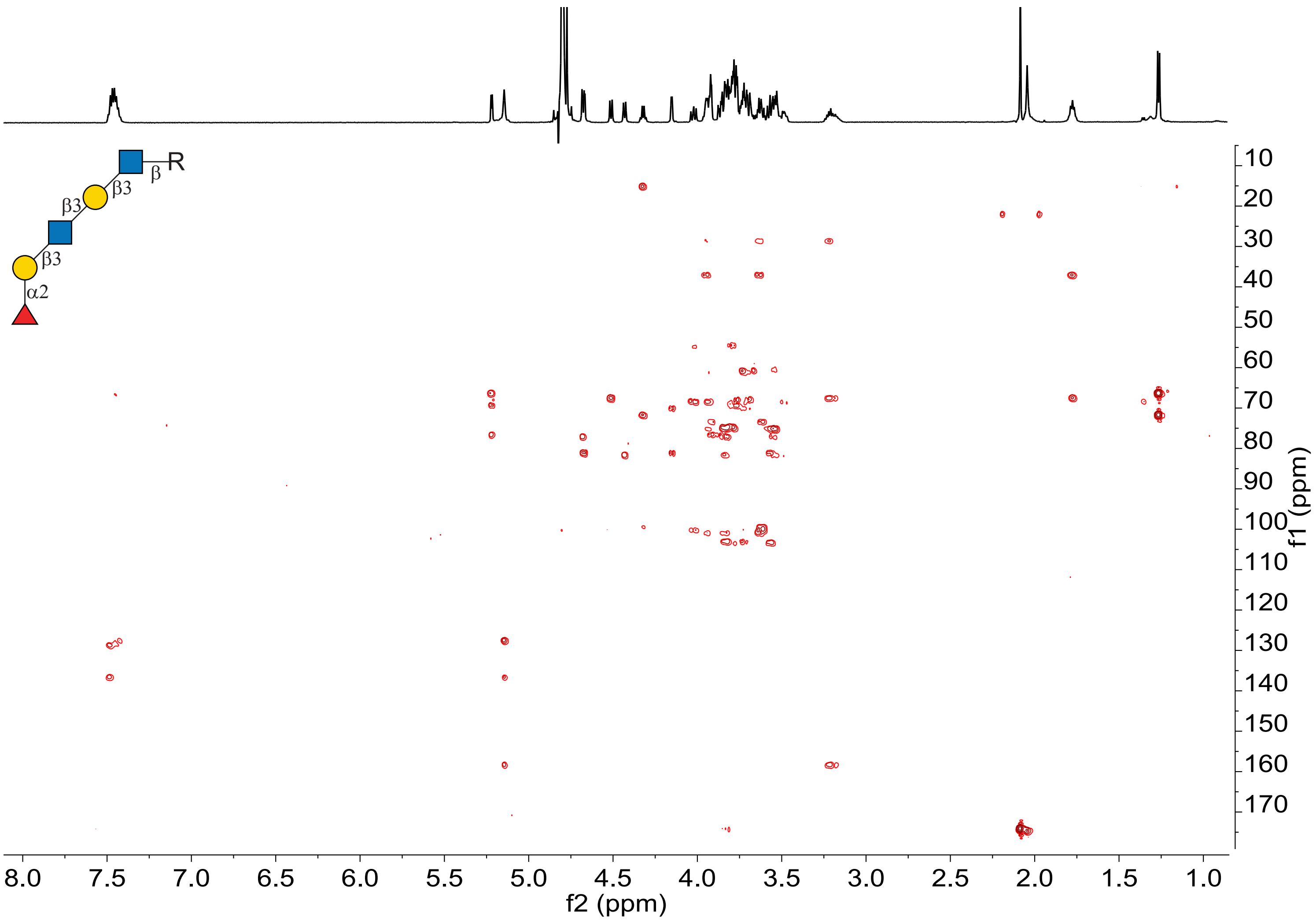

HMBC of Compound **30**

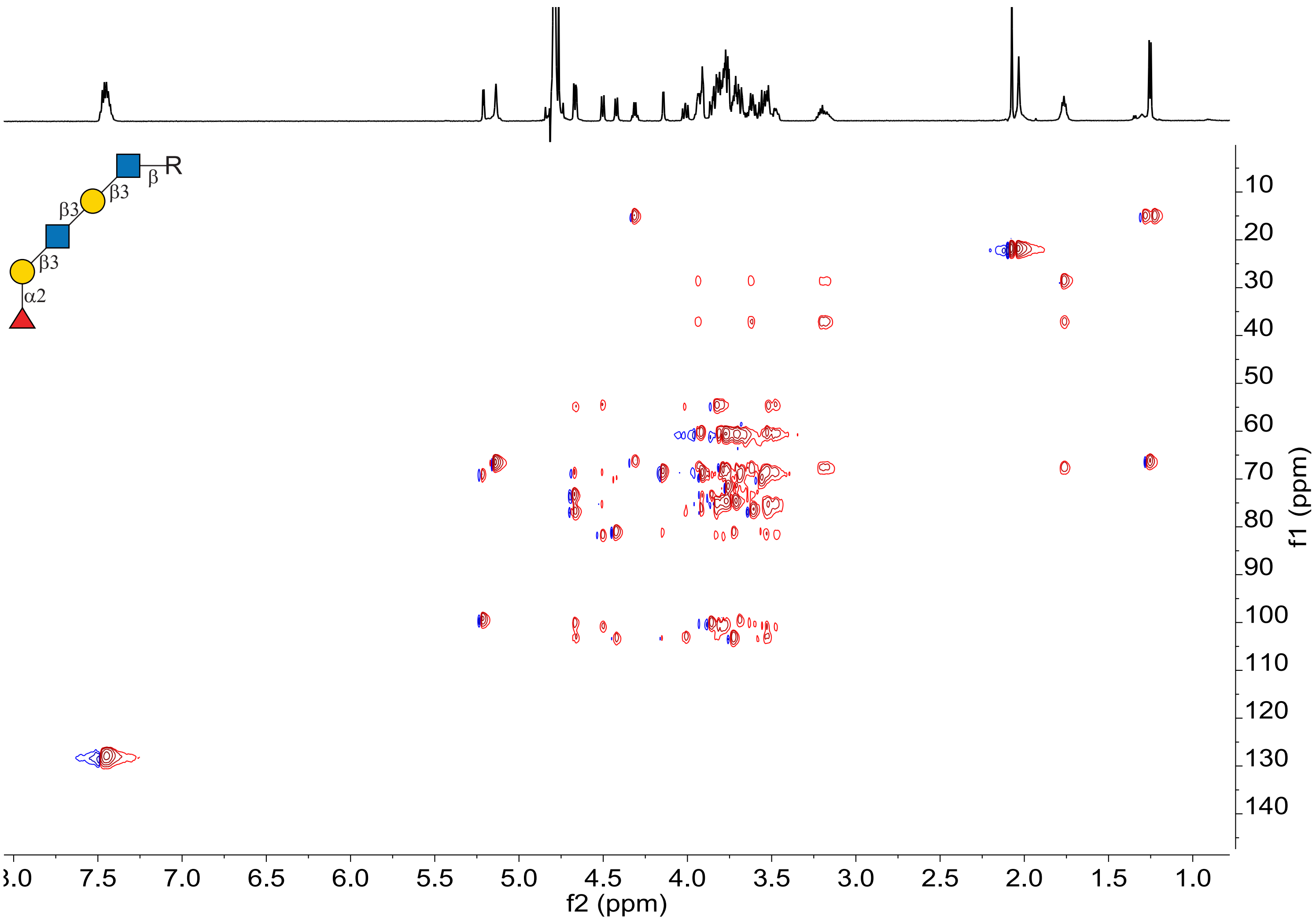

HSQC-TOCSY of Compound **30**

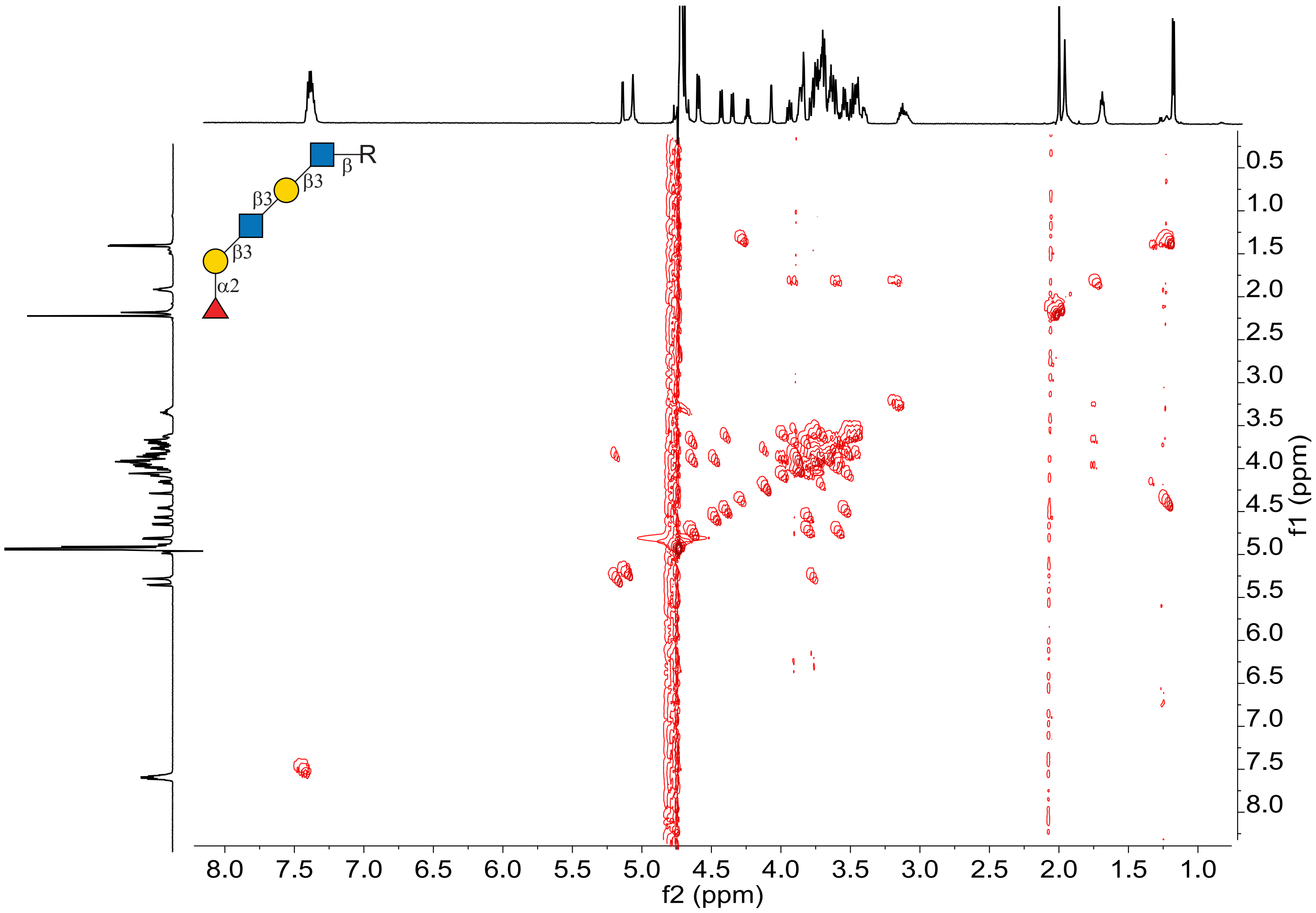

^{1}H-^{1}H COSY of Compound **30**

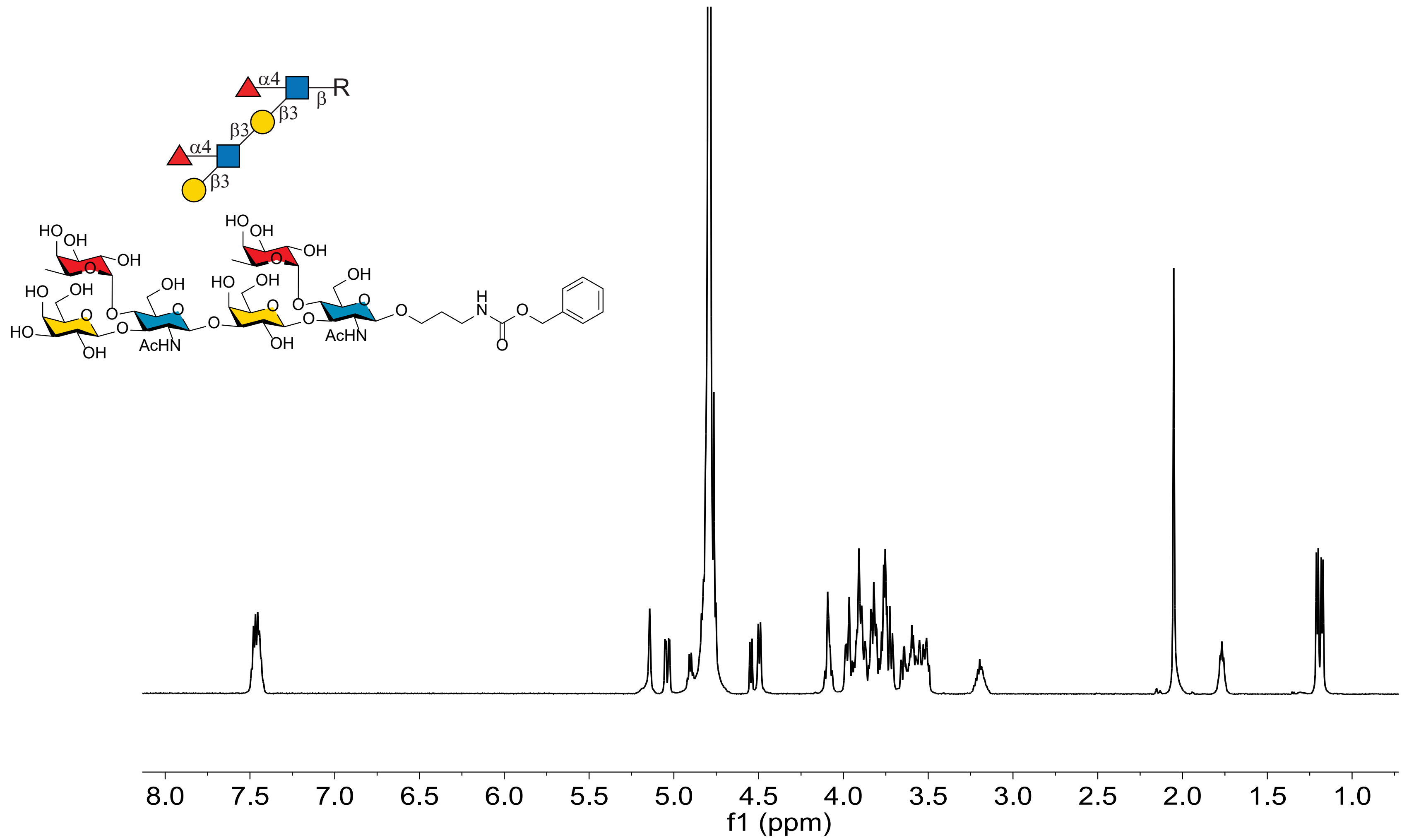

^{1}H NMR of Compound **31**

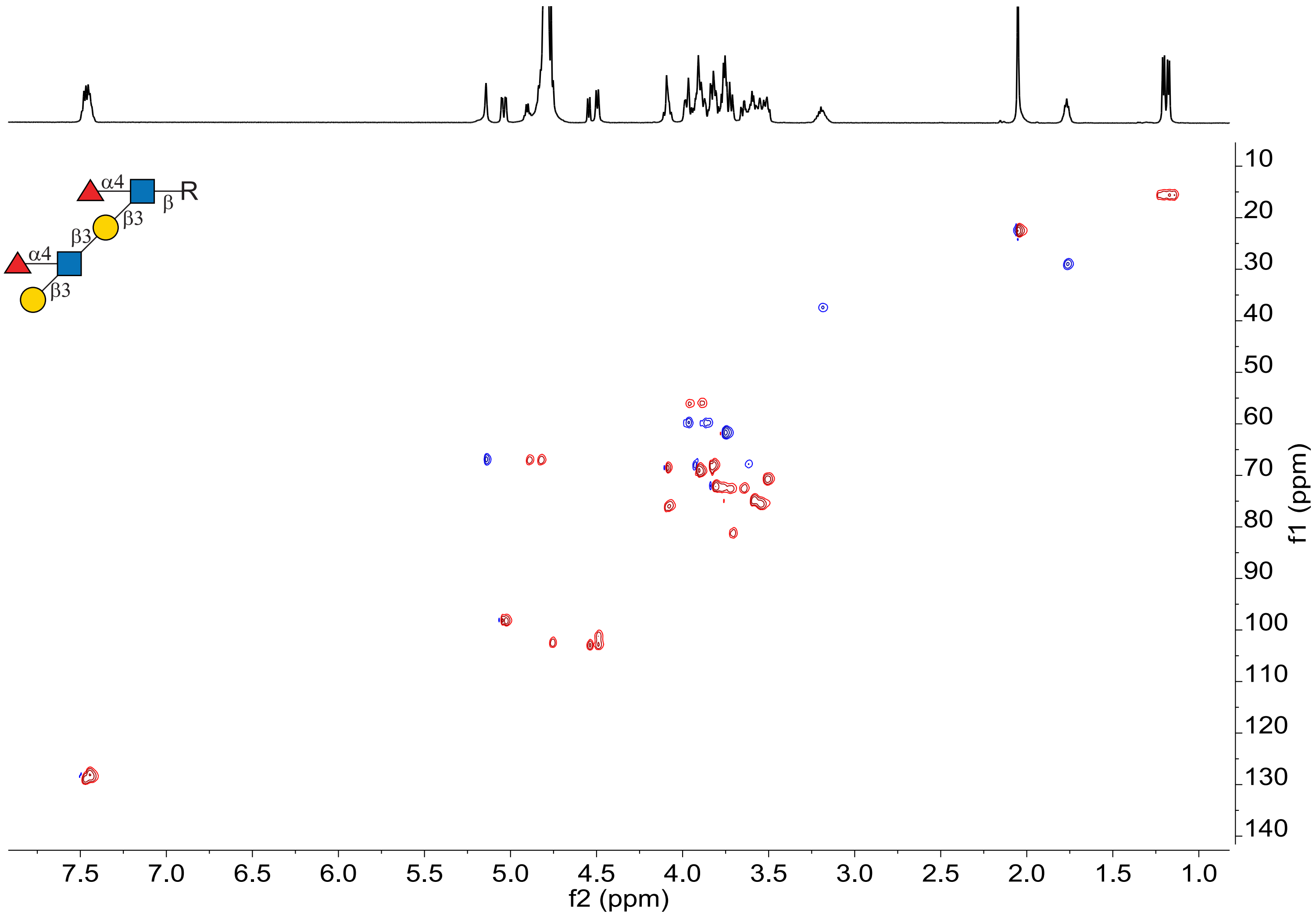

HSQC of Compound **31**

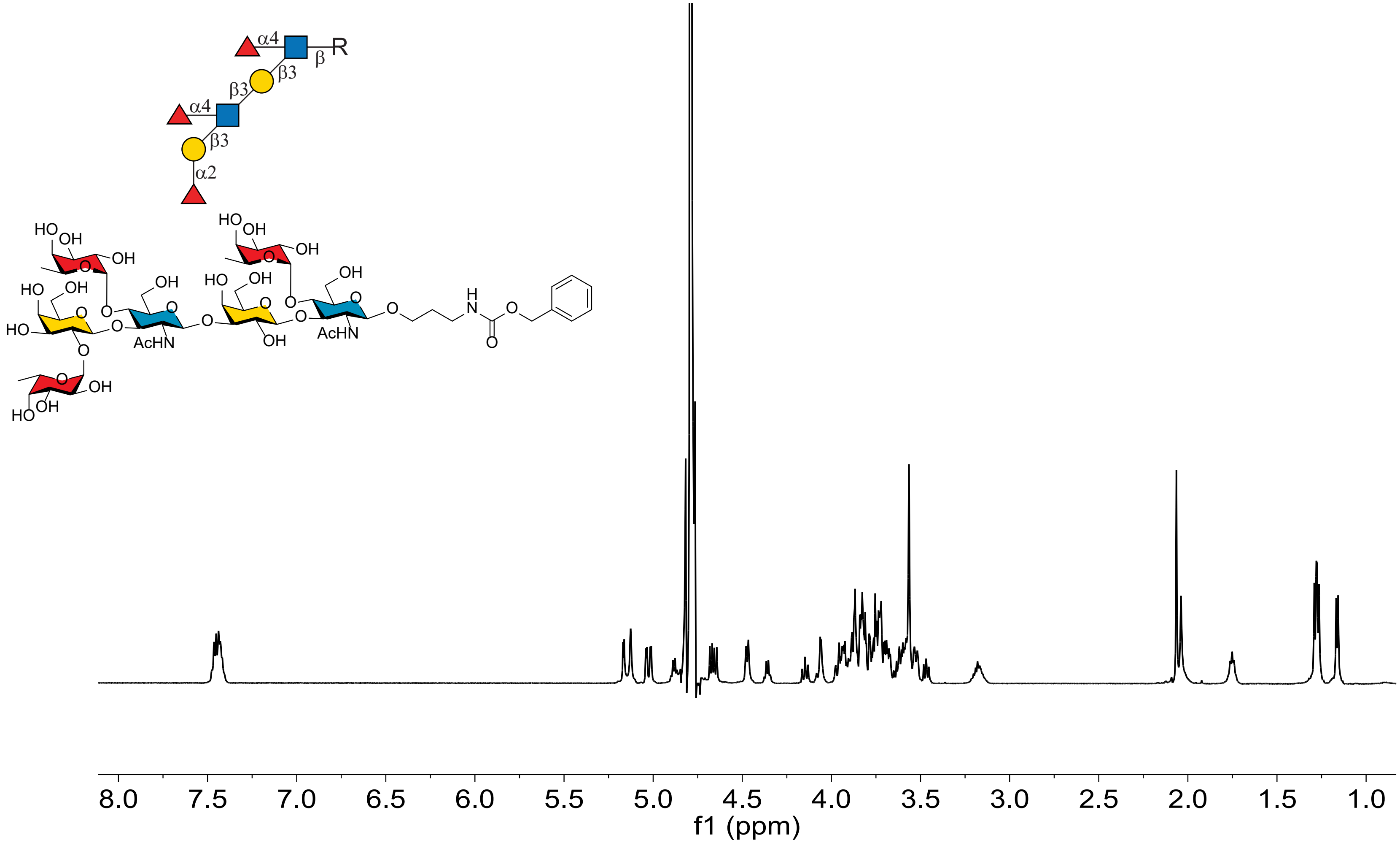

^{1}H NMR of Compound **32**

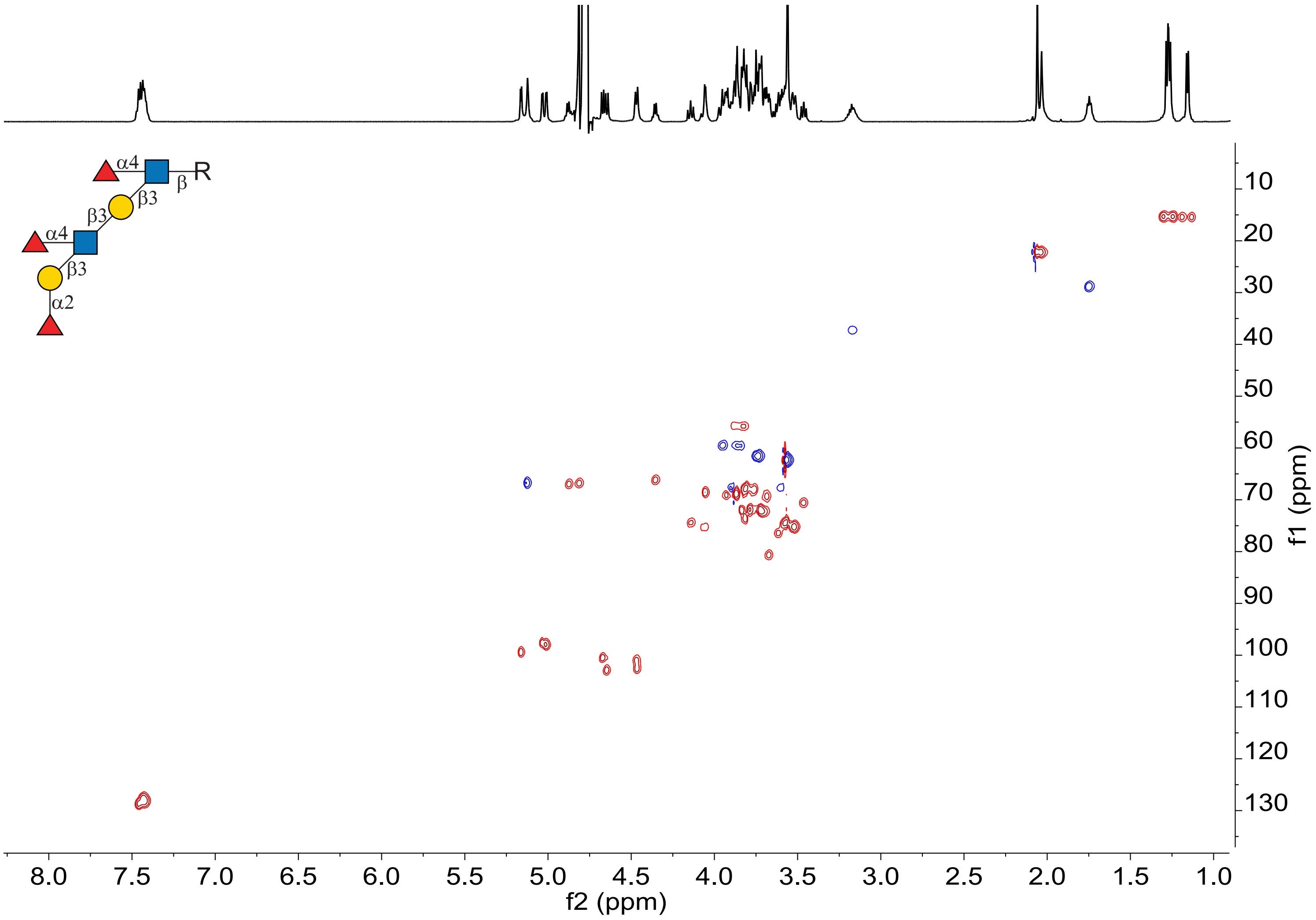

HSQC of Compound **32**

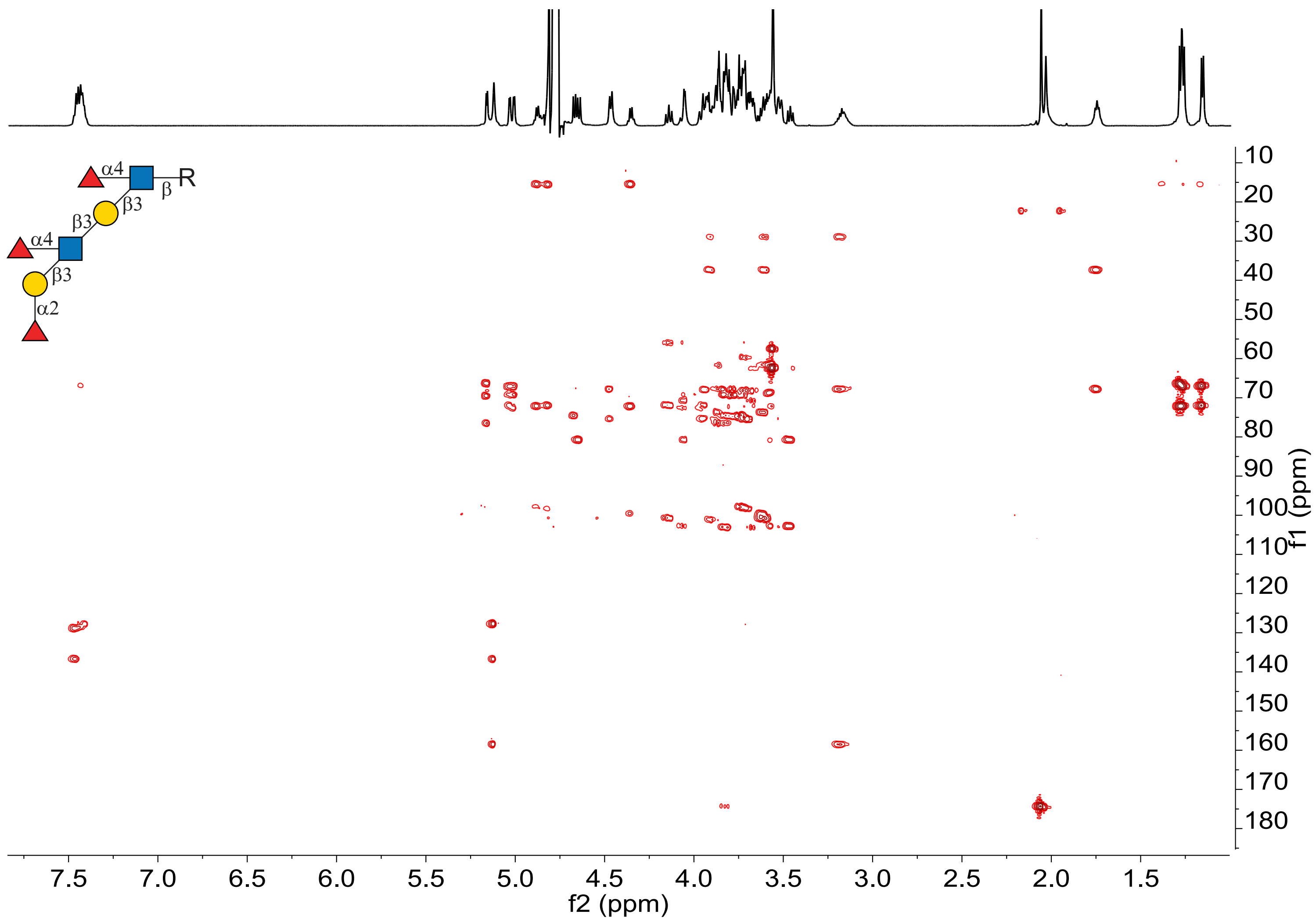

HMBC of Compound **32**

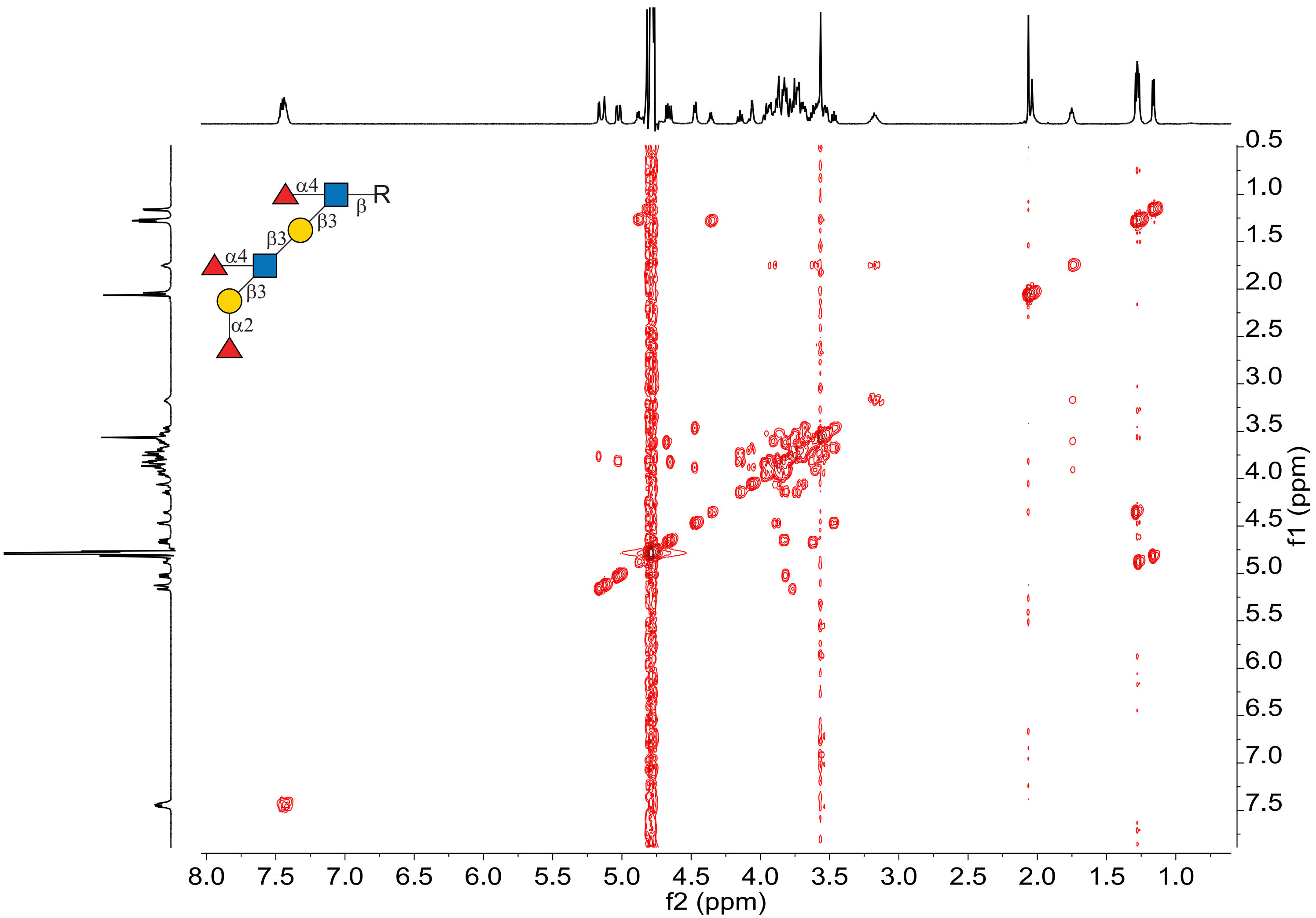

^{1}H-^{1}H COSY of Compound **32**

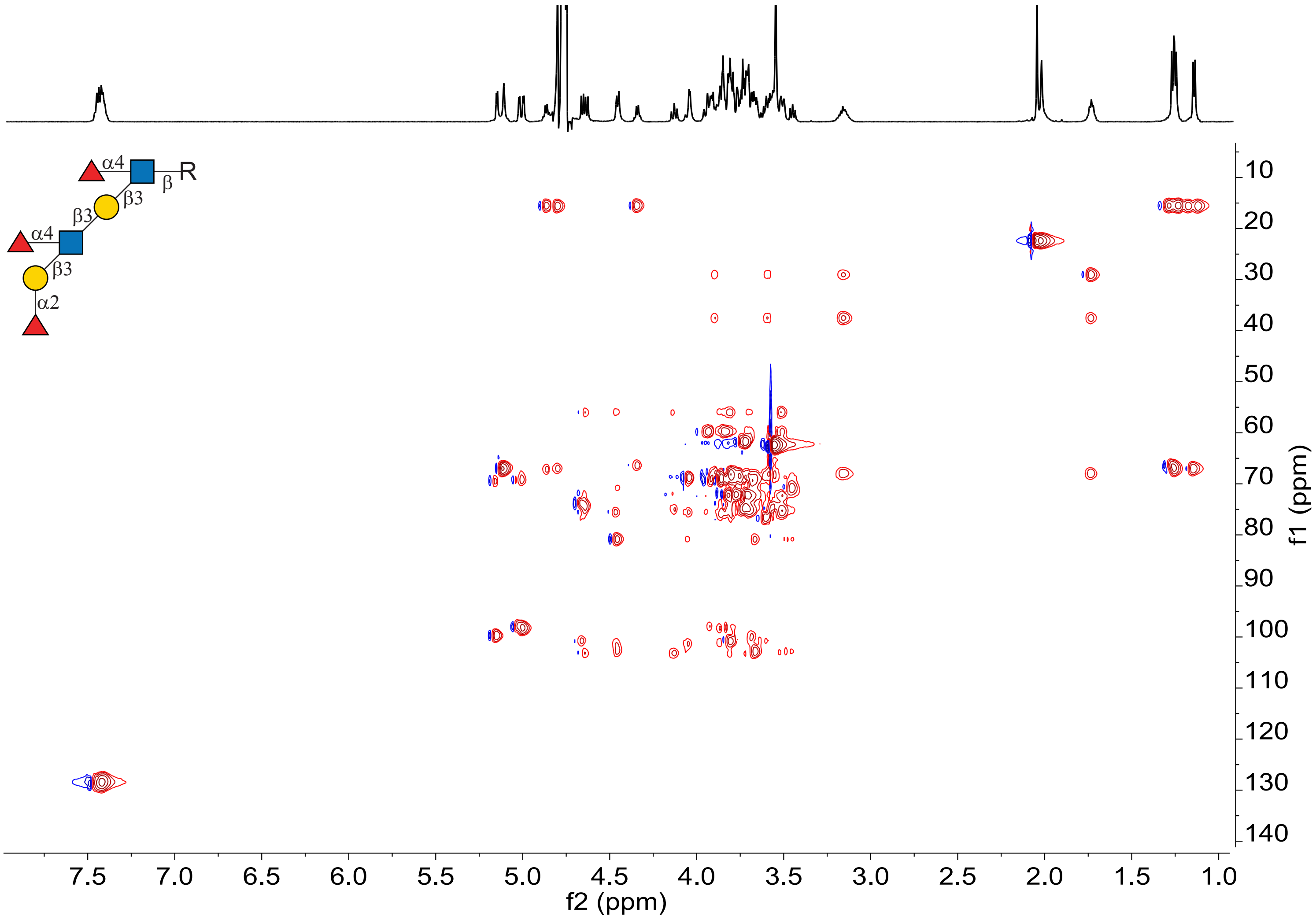

HSQC-TOCSY of Compound **32**

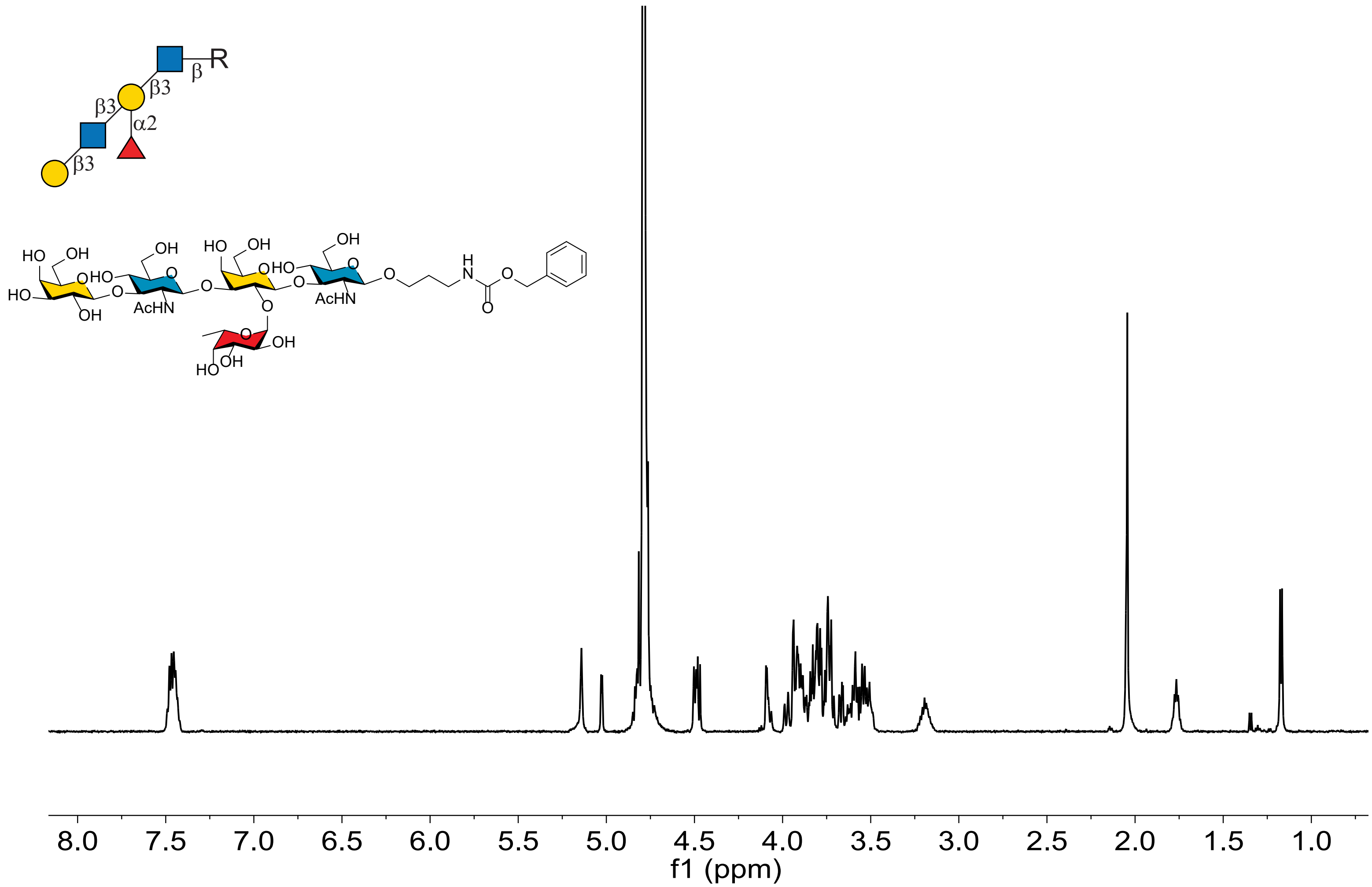

^{1}H NMR of Compound **33**

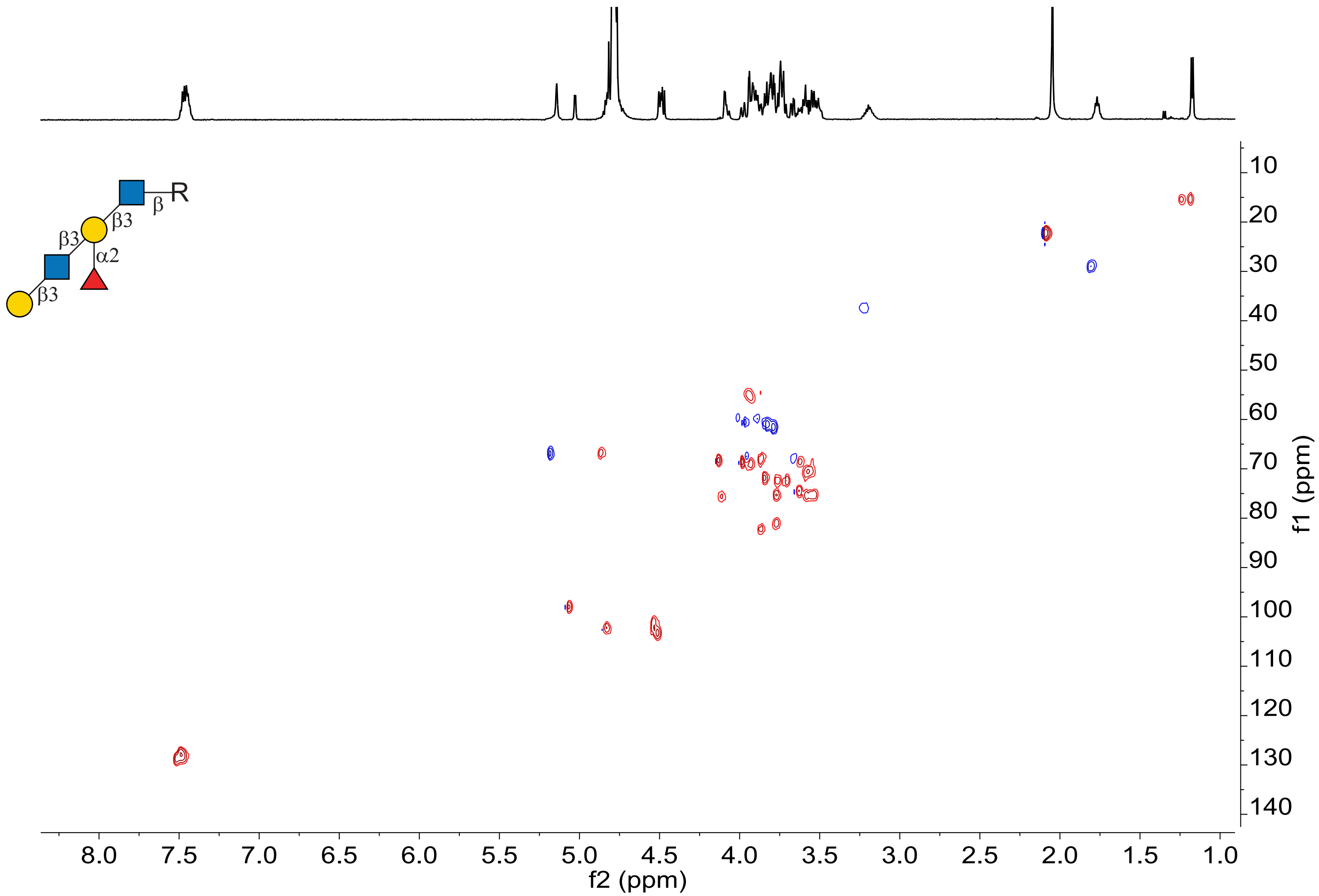

HSQC of Compound **33**

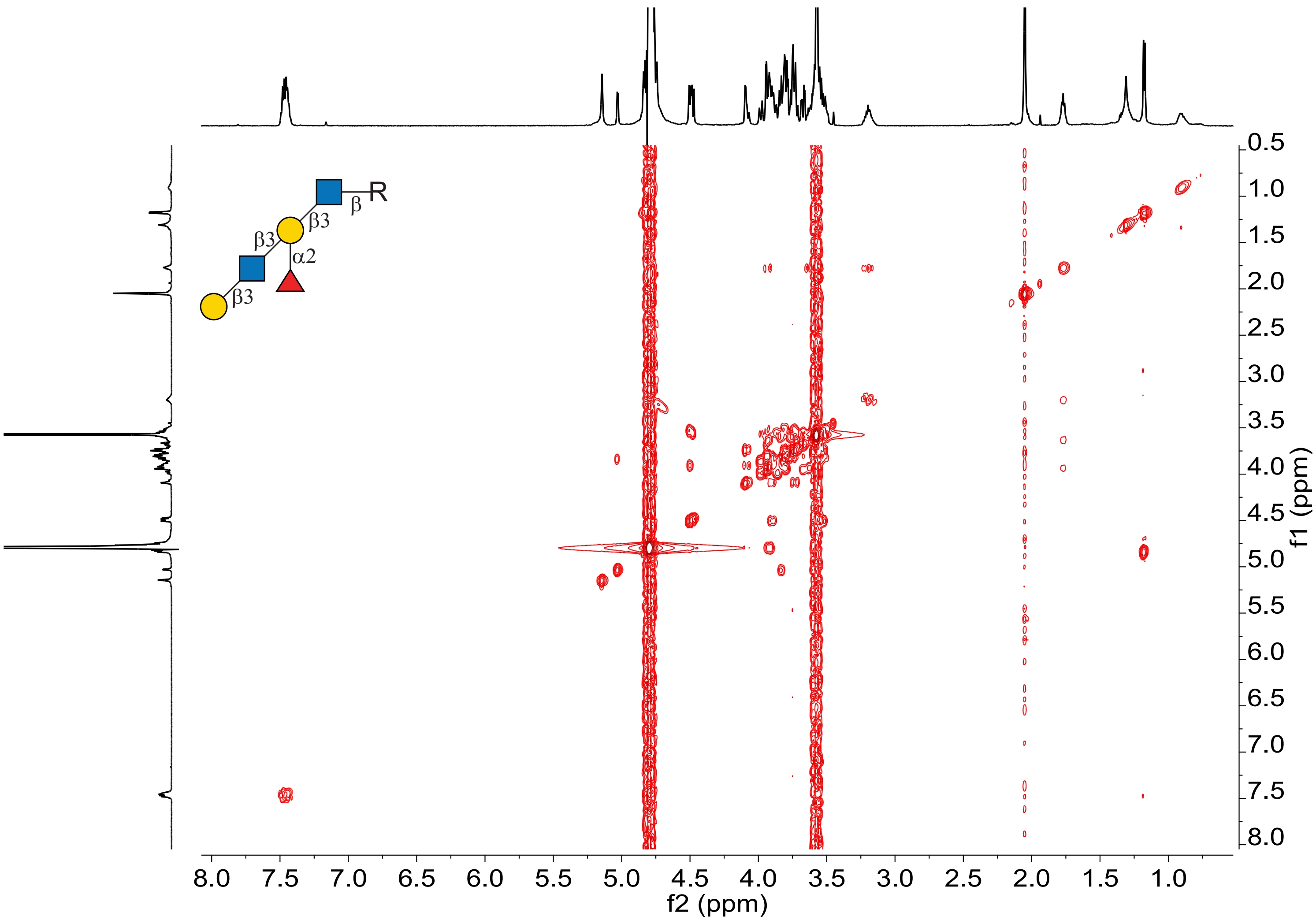

^{1}H-^{1}H COSY of Compound **33**

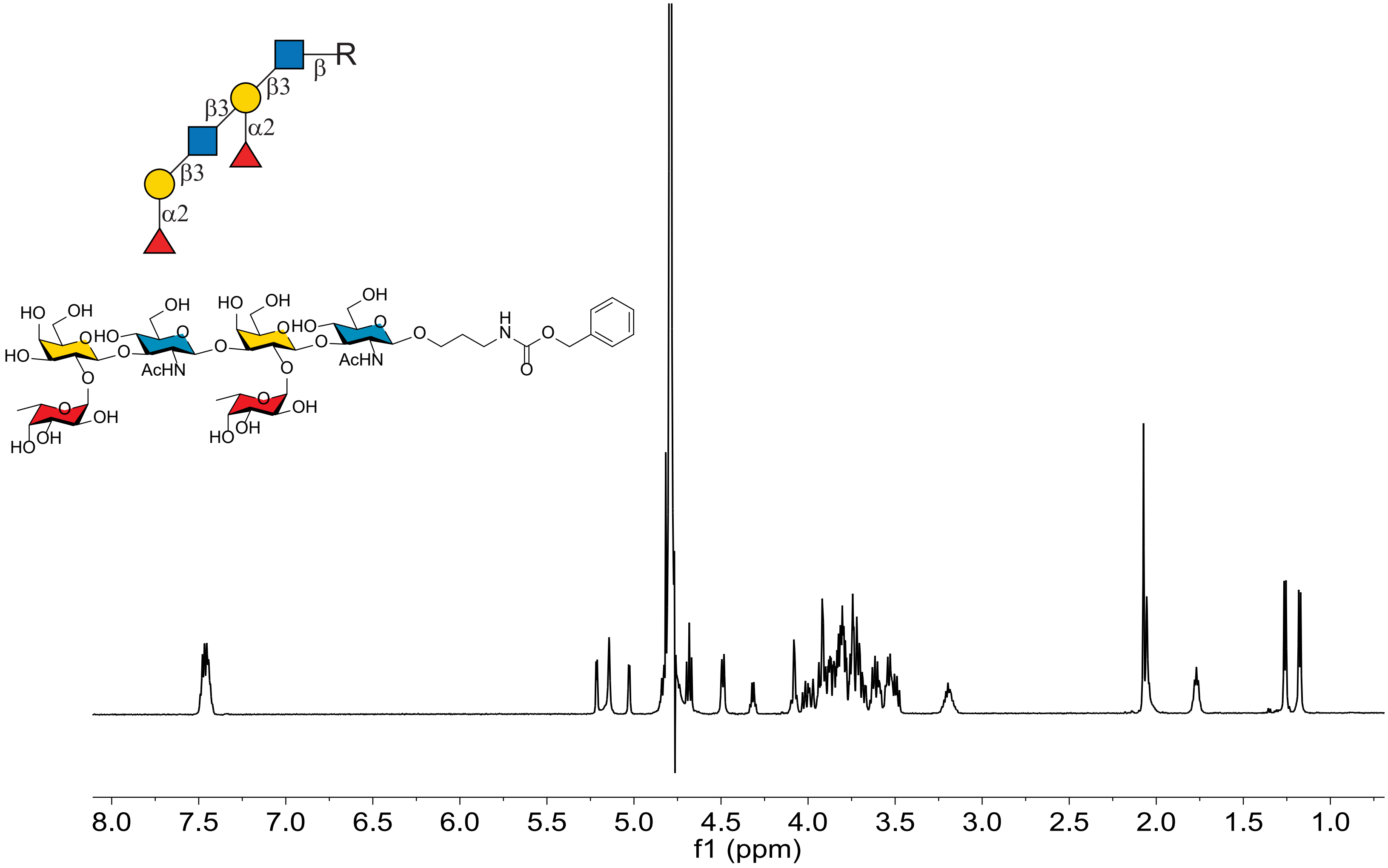

^{1}H NMR of Compound **34**

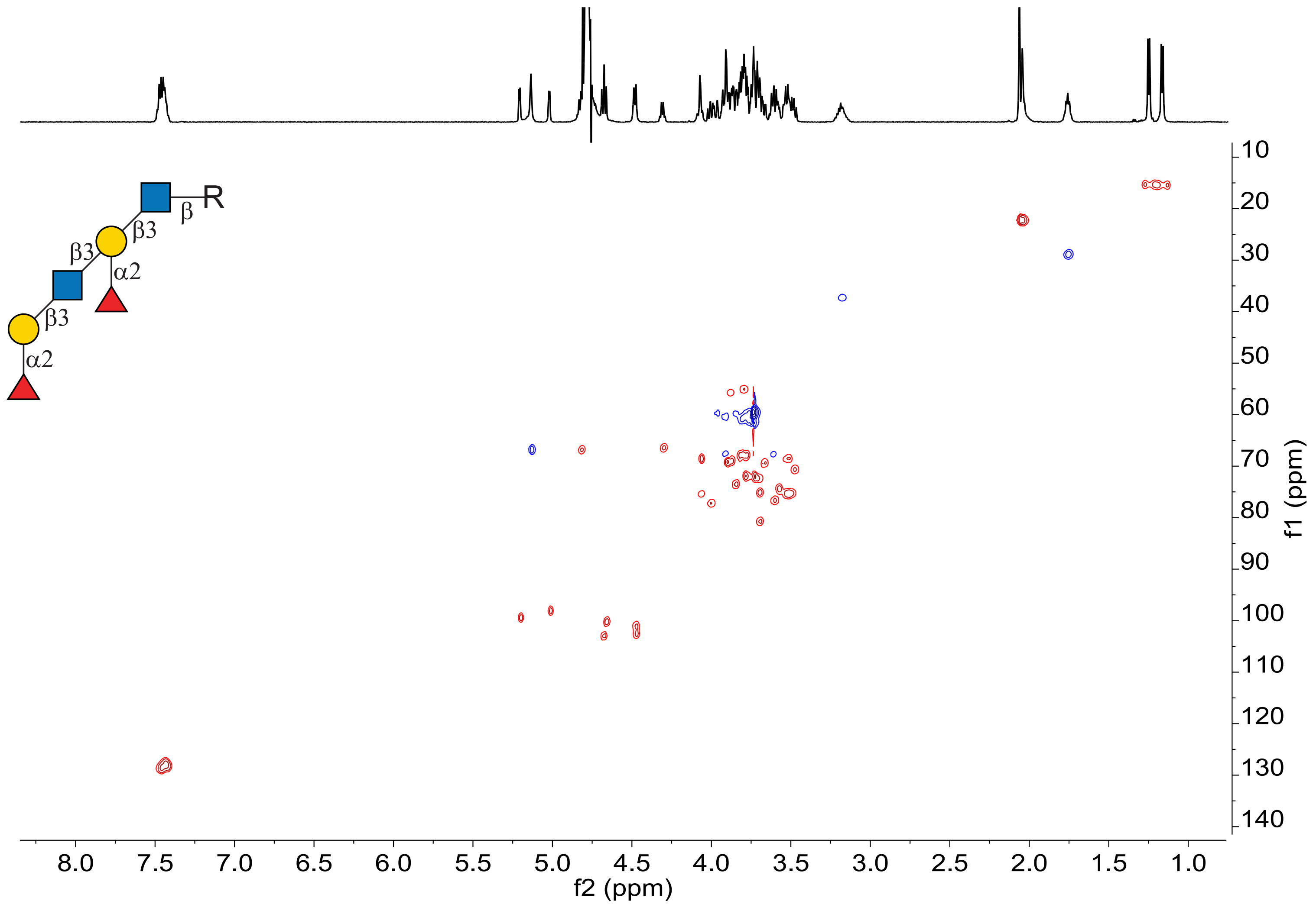

HSQC of Compound **34**

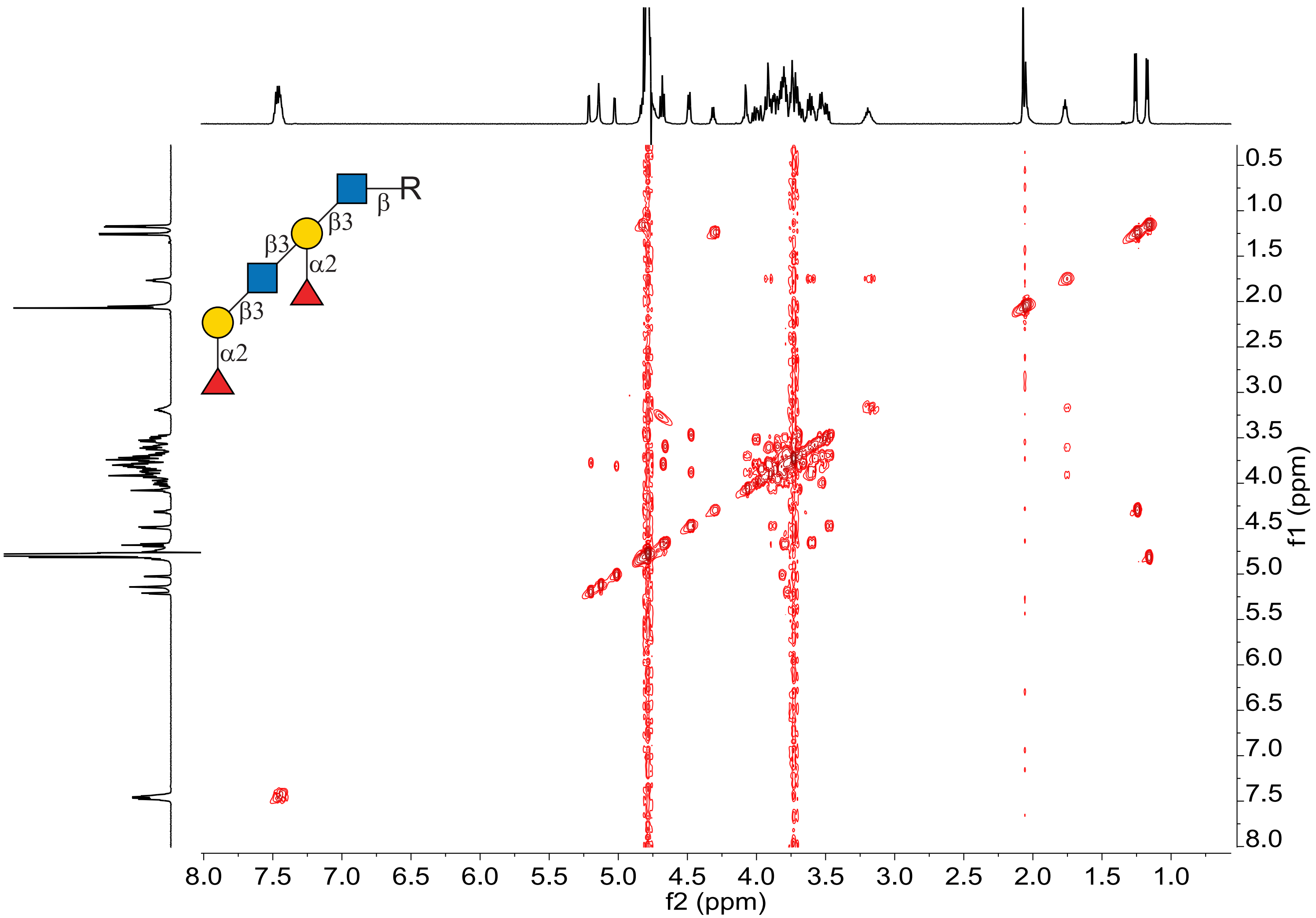

^{1}H-^{1}H COSY of Compound **34**

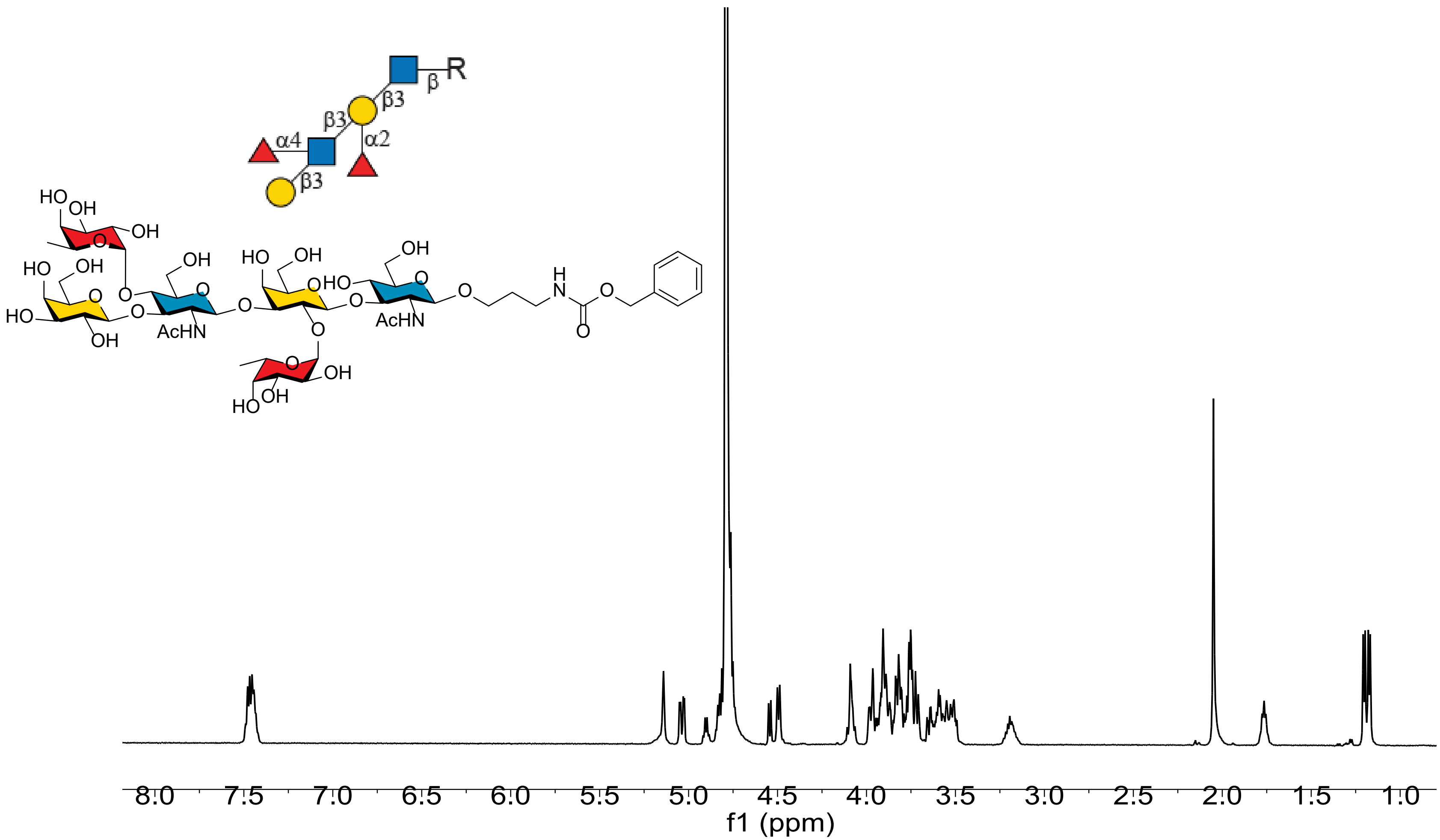

^{1}H NMR of Compound **35**

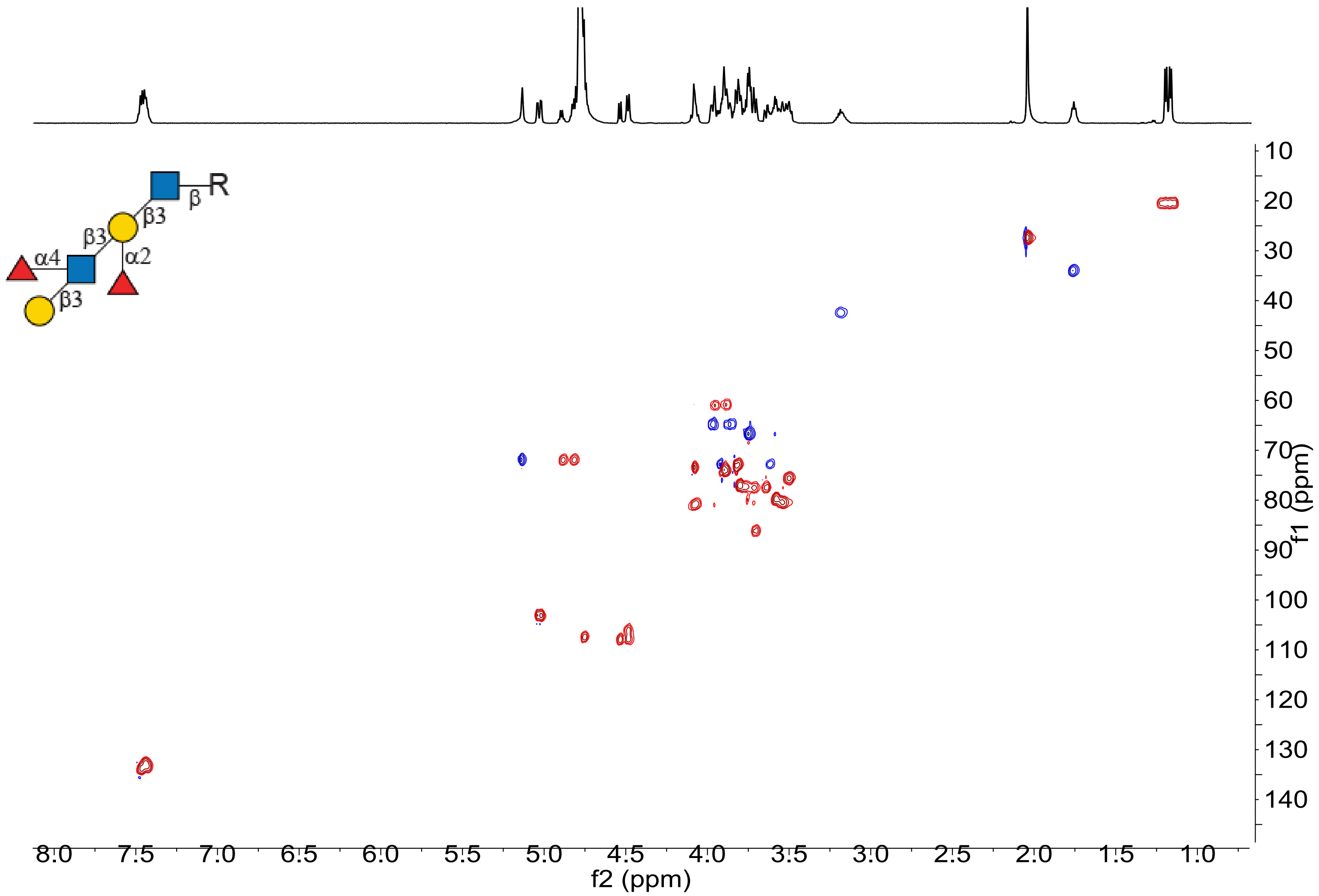

HSQC of Compound **35**

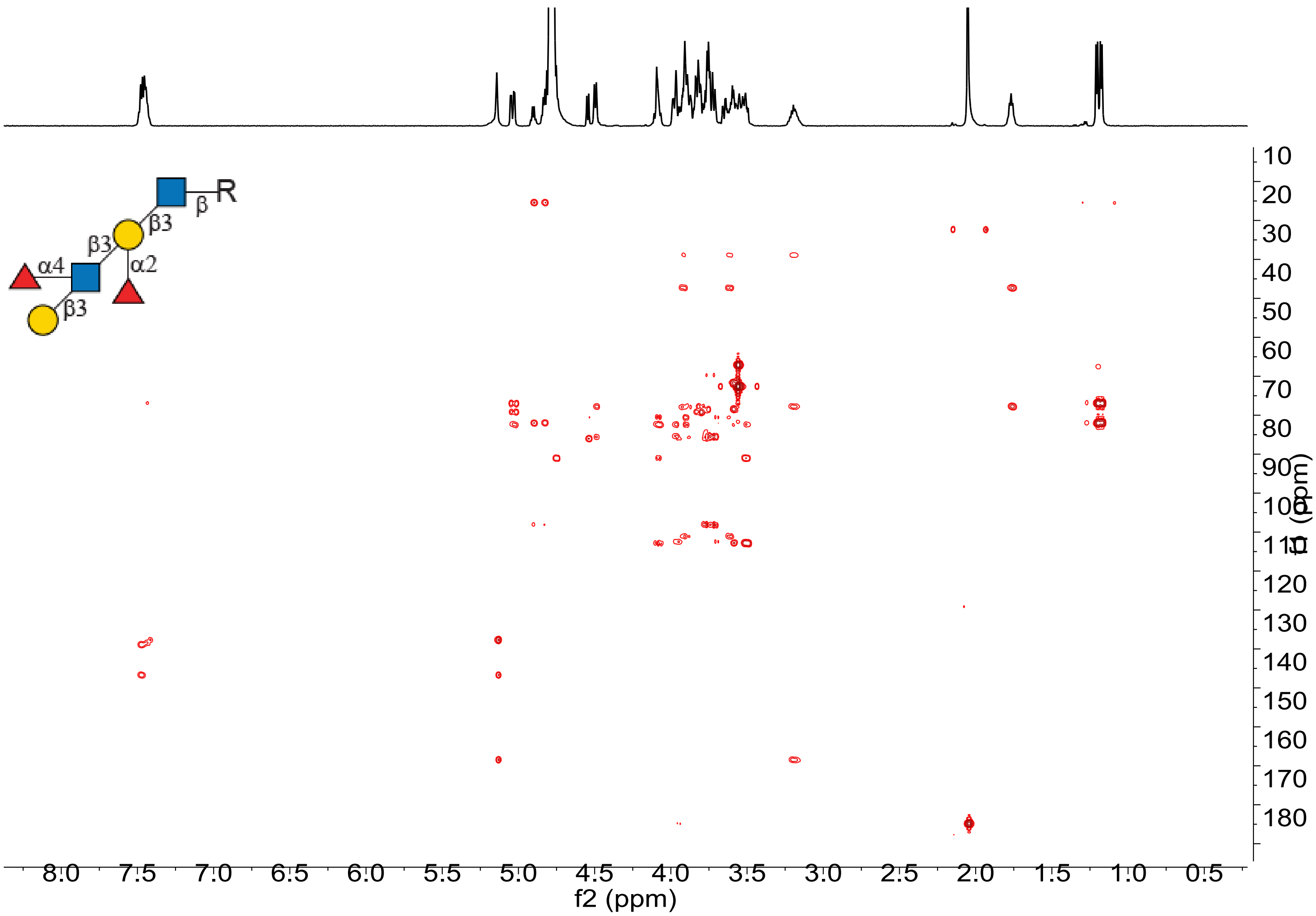

HMBC of Compound **35**

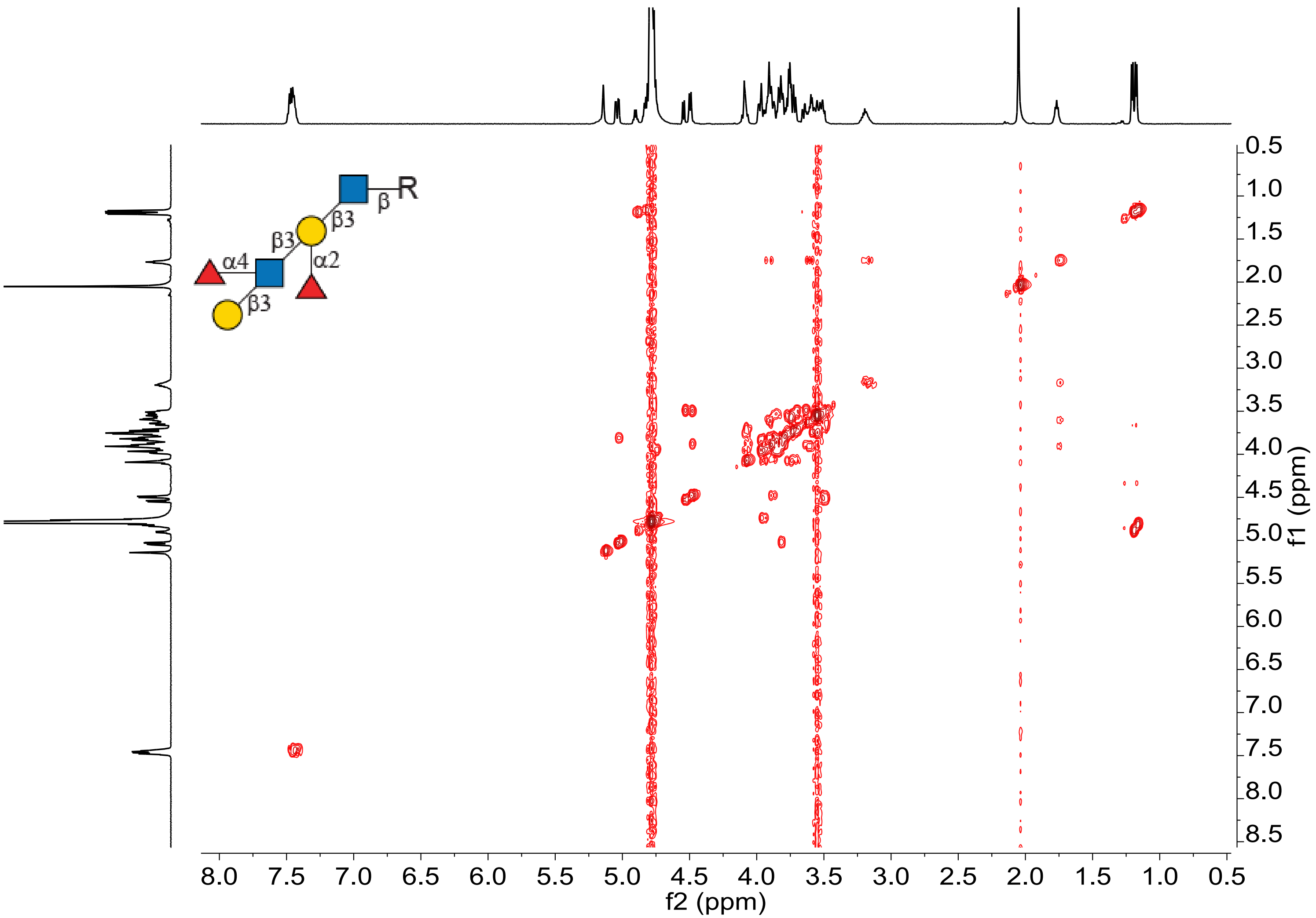

^{1}H-^{1}H COSY of Compound **35**

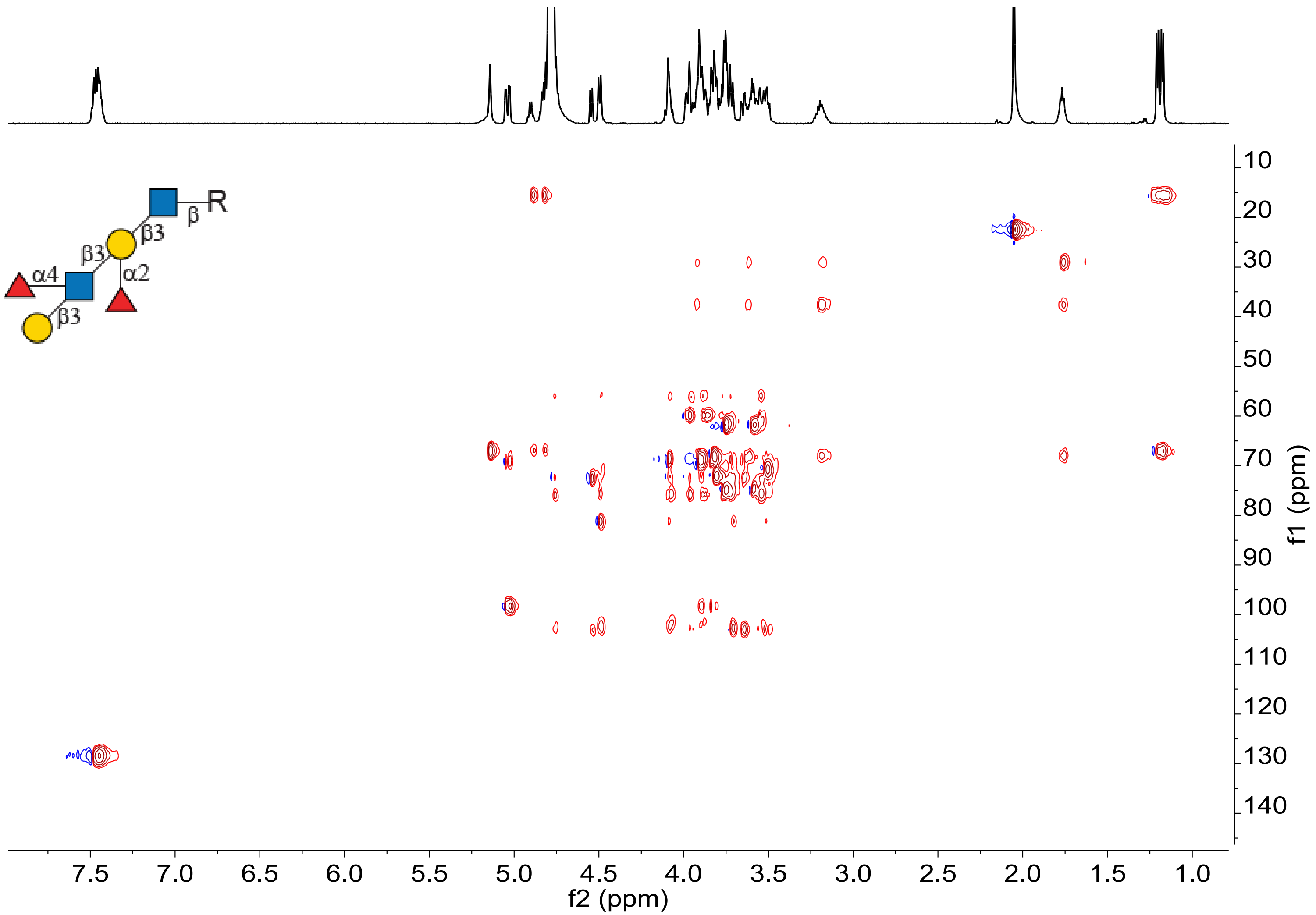

HSQC-TOCSY of Compound **35**

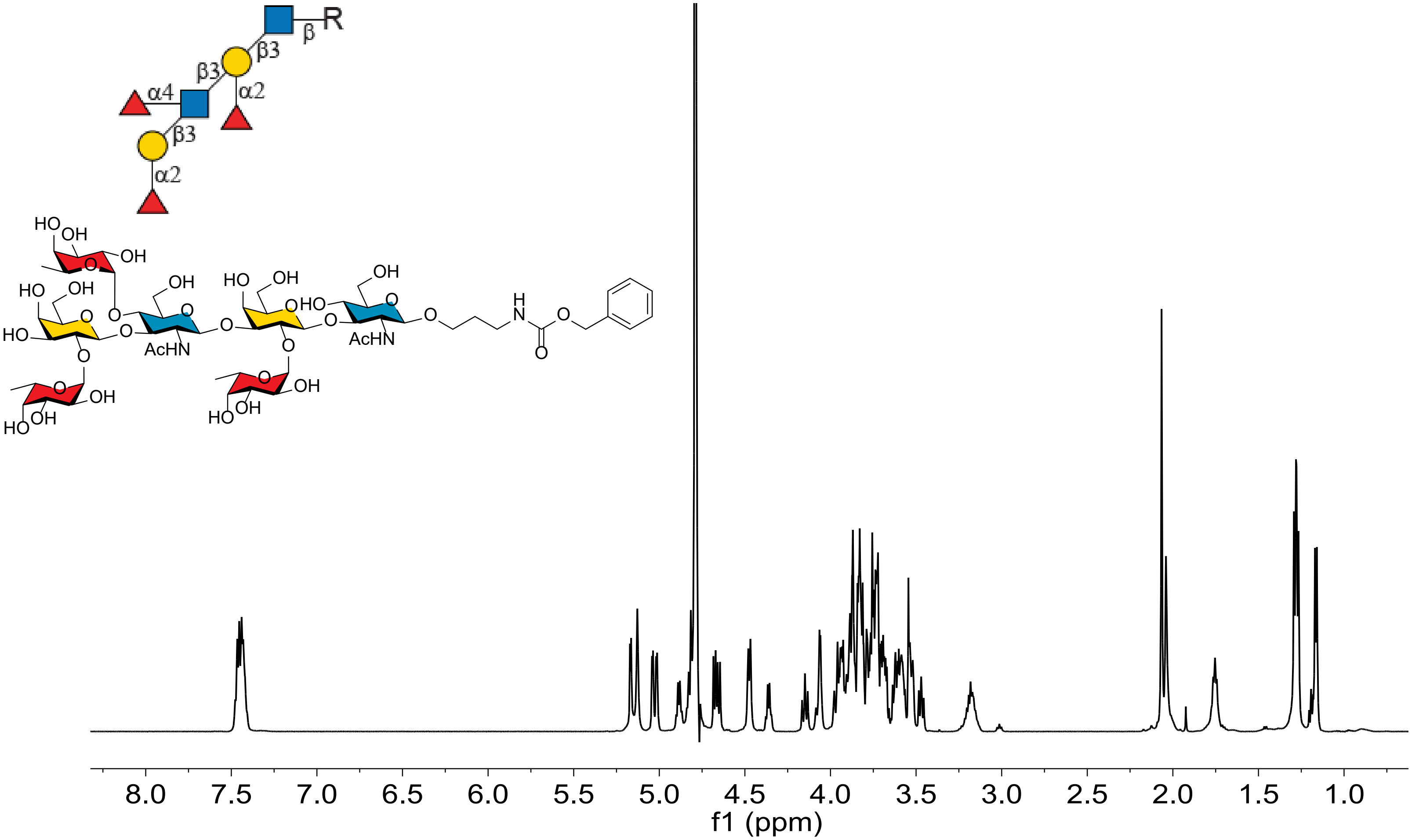

^{1}H NMR of Compound **36**

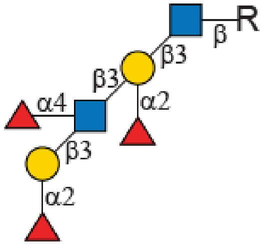

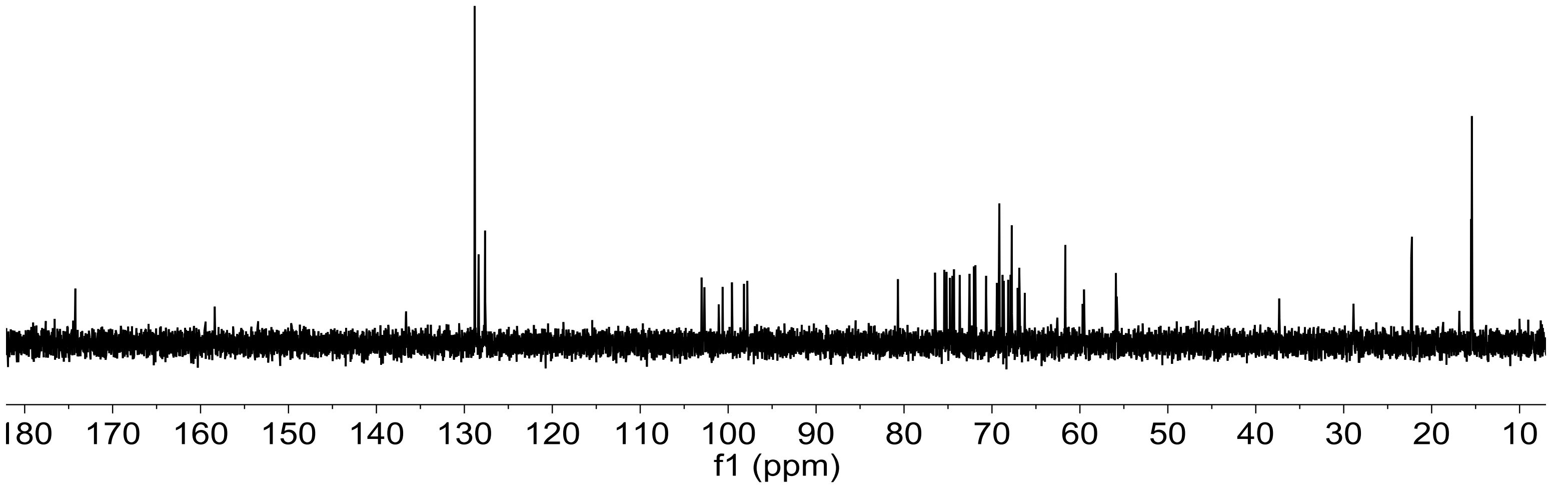

^{13}C NMR of Compound **36**

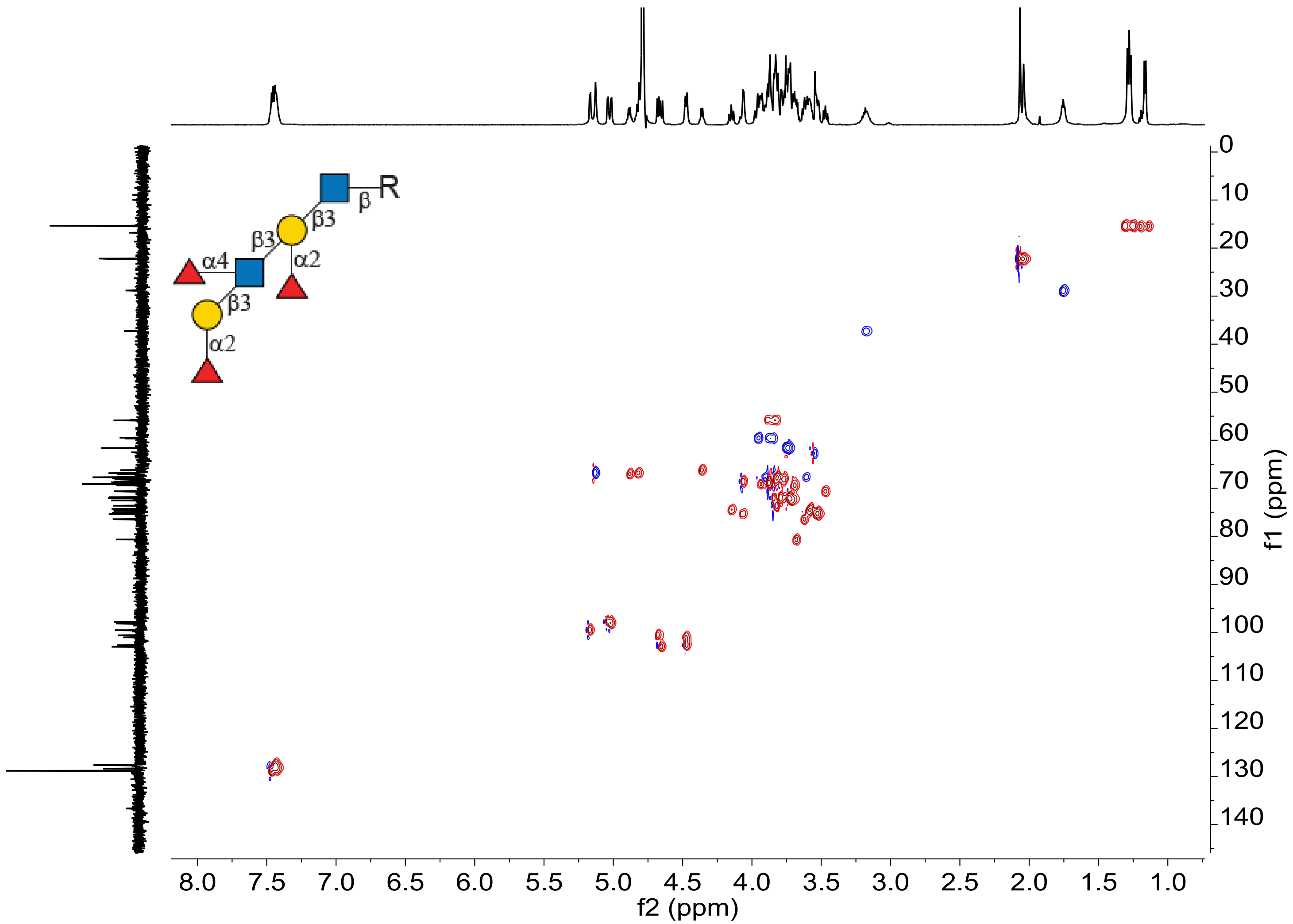

HSQC of Compound **36**

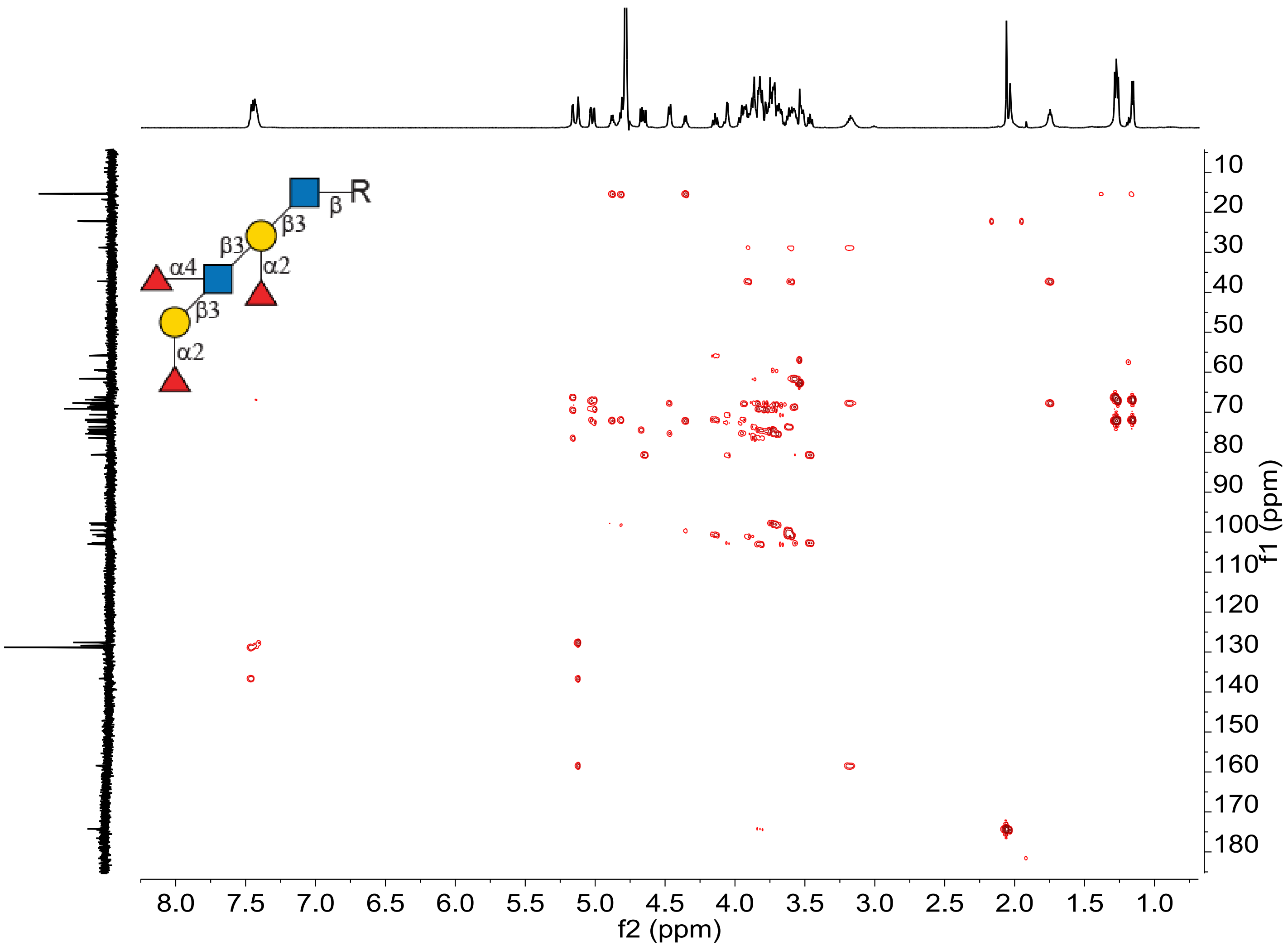

HMBC of Compound **36**

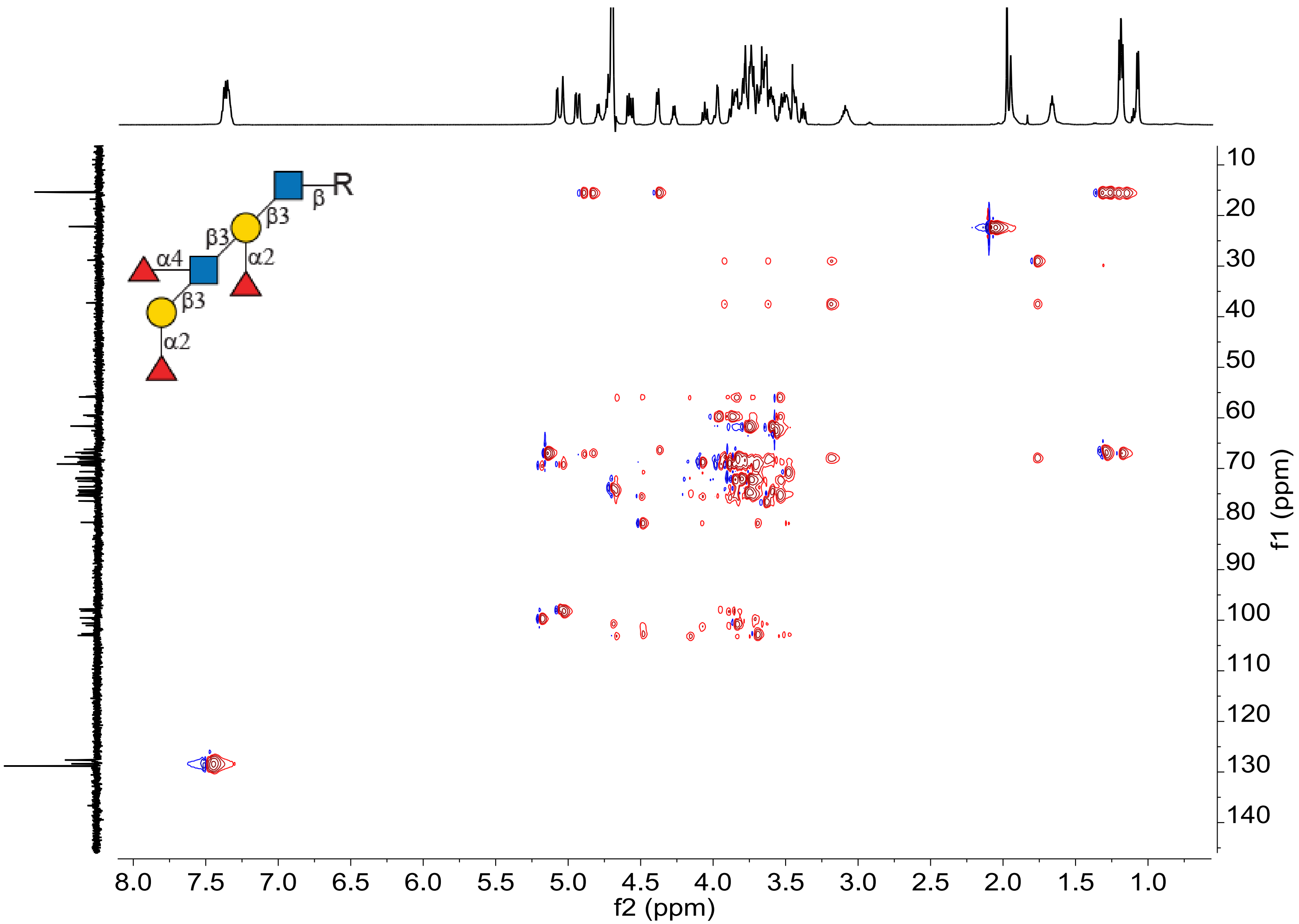

HSQC-TOCSY of Compound **36**

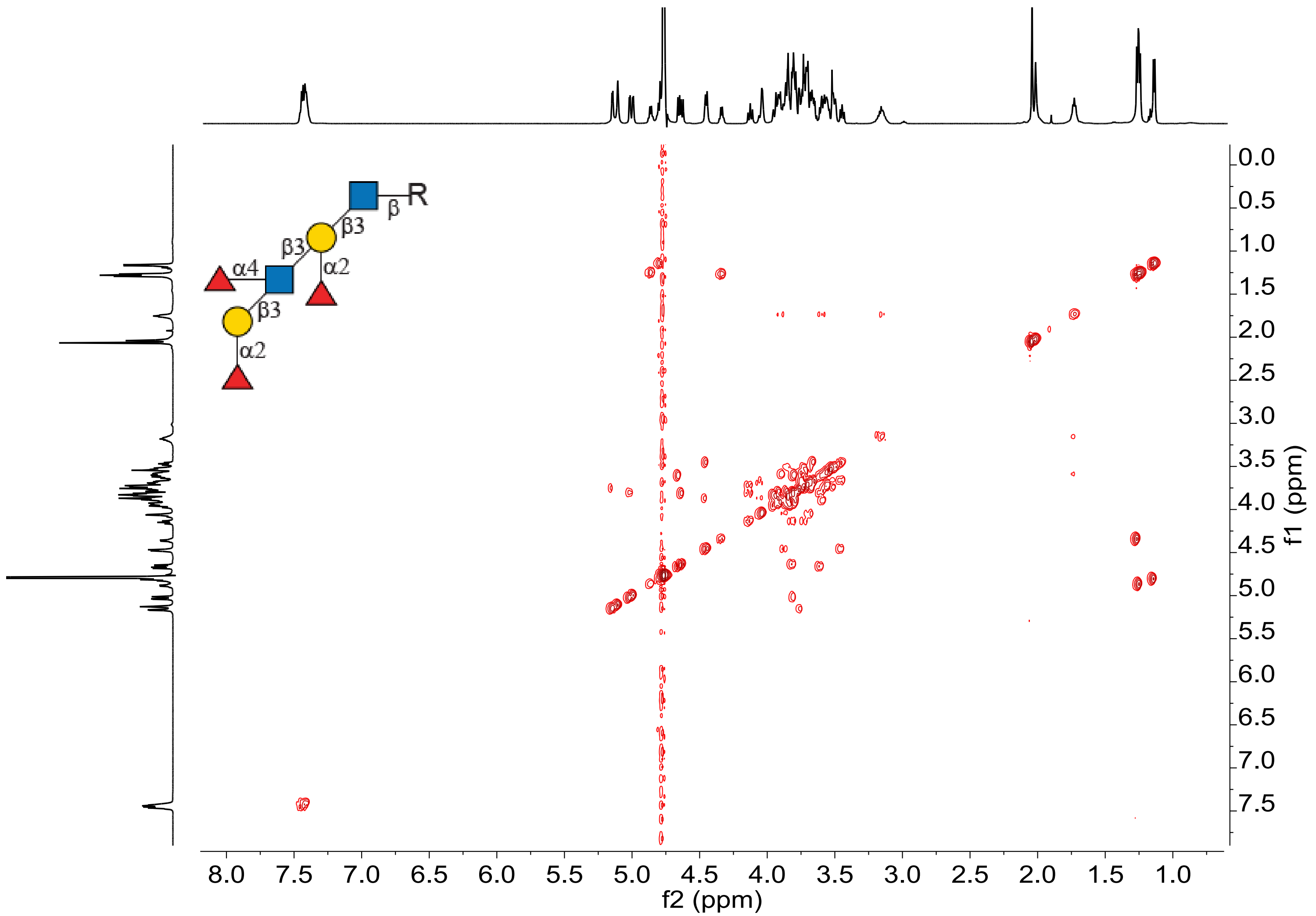

^{1}H-^{1}H COSY of Compound **36**

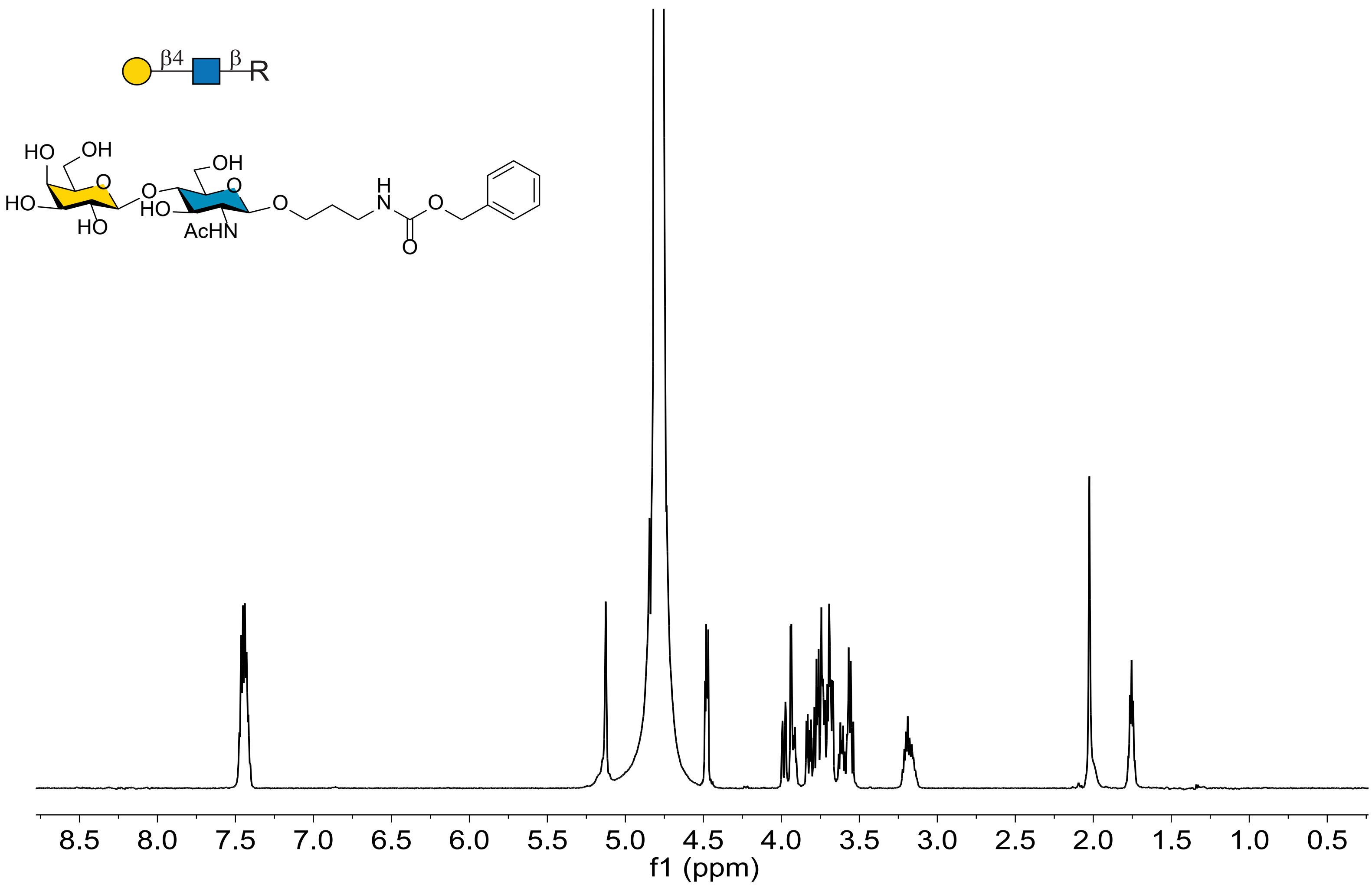

^{1}H NMR of Compound **LN**

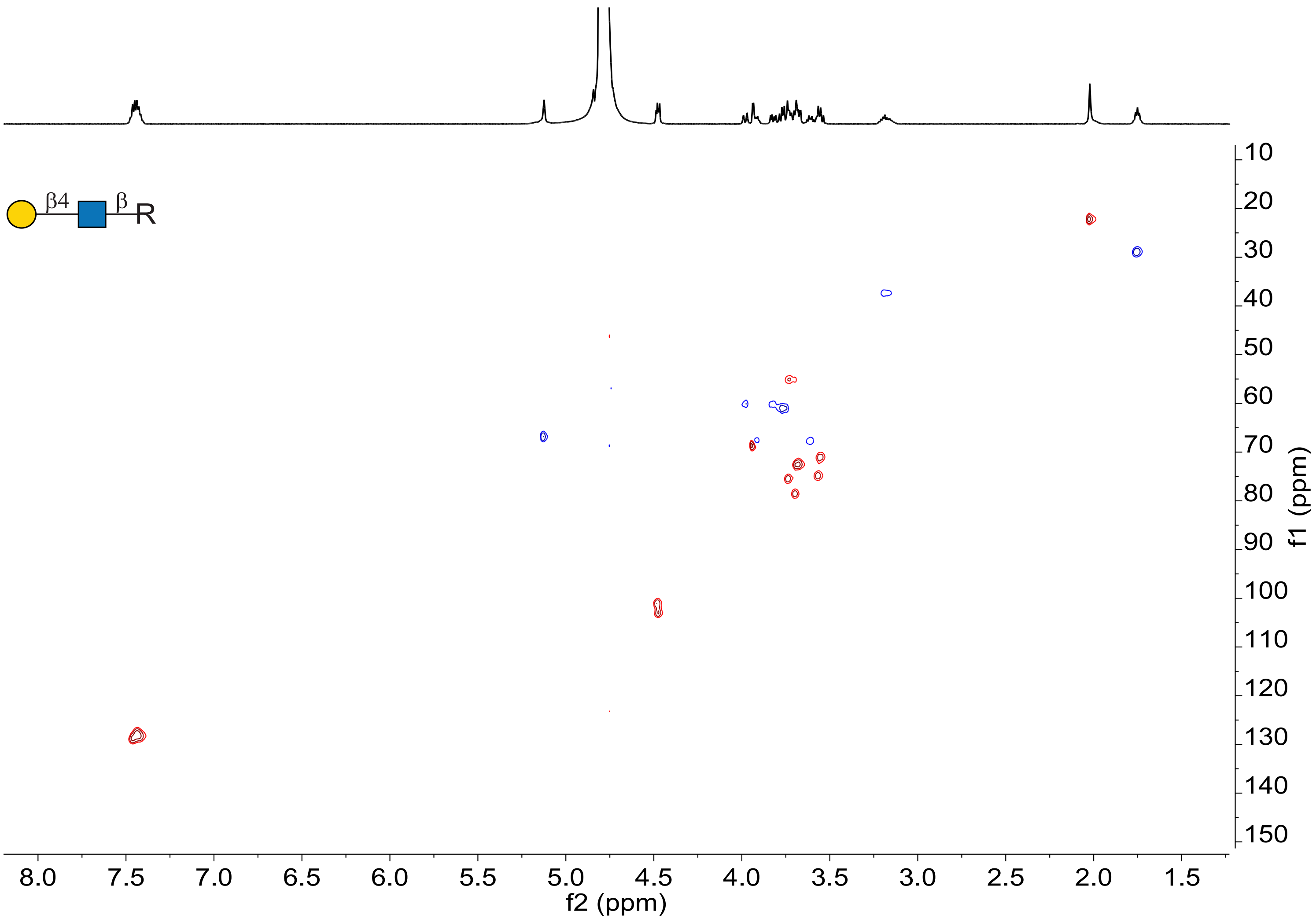

HSQC of Compound **LN**

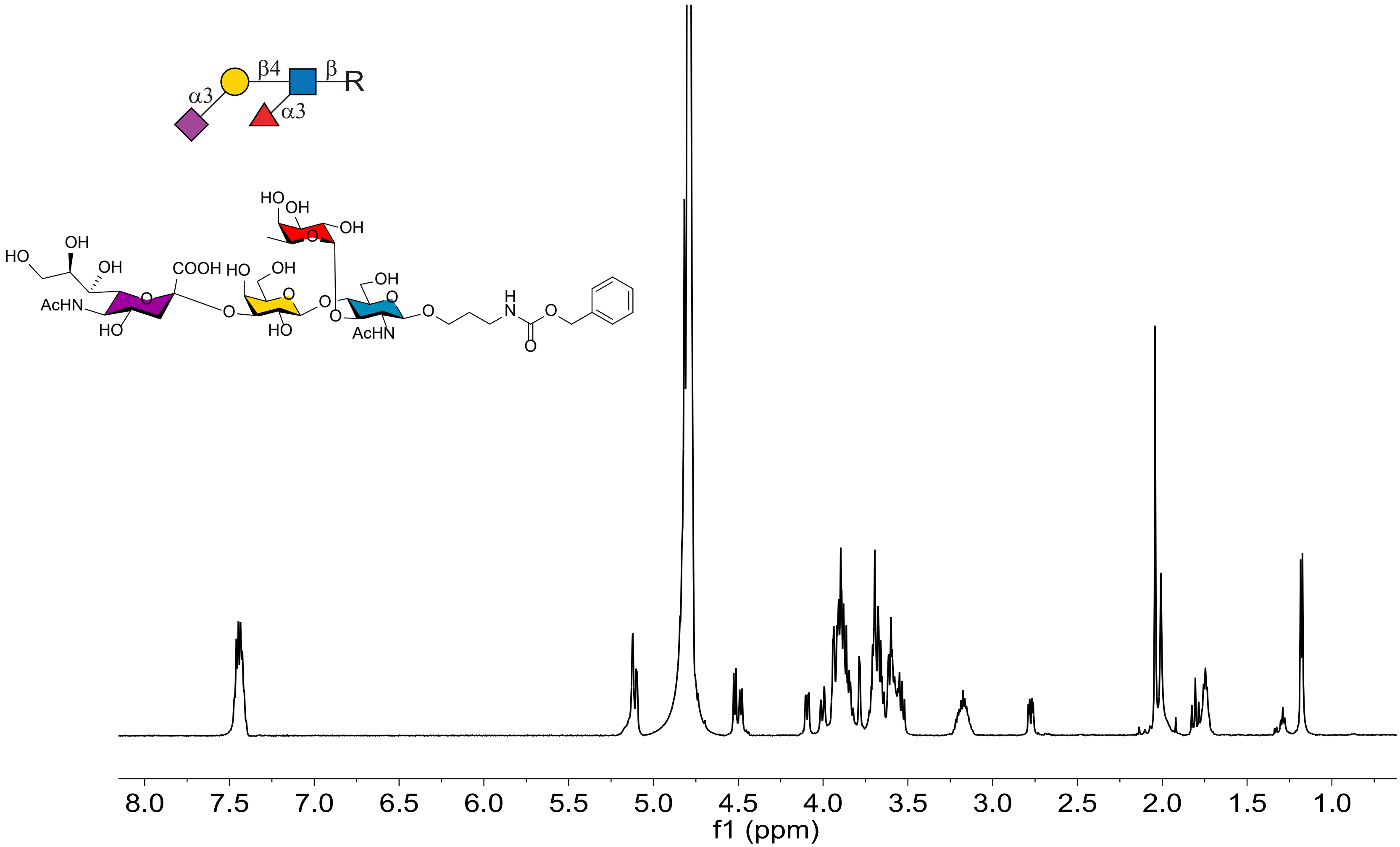

^{1}H NMR of Compound **SLe^{X}**

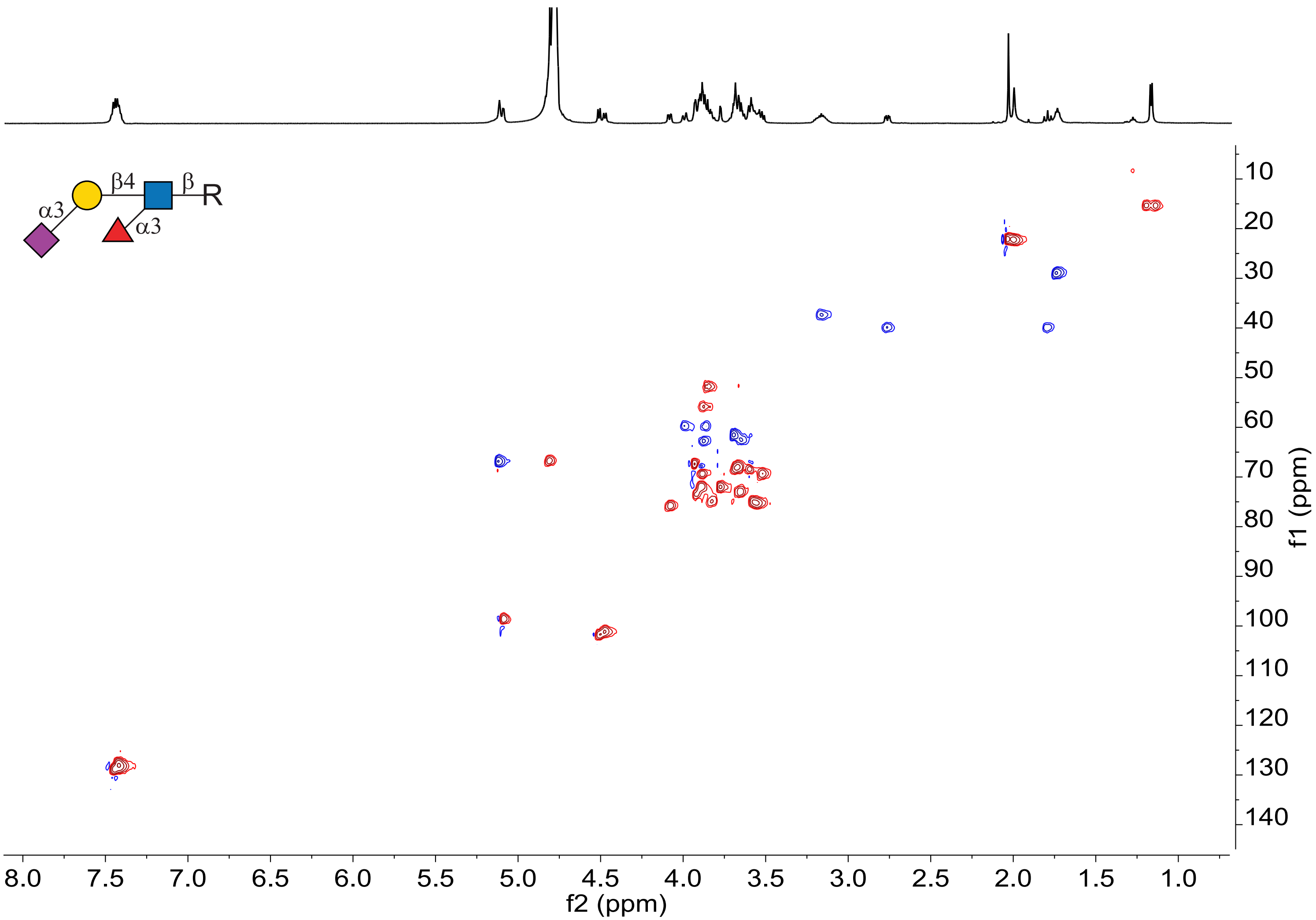

HSQC of Compound **SLe^X**
